# Neoplastic and Stromal Cells Contribute to an Extracellular Matrix Gene Expression Profile Defining a Breast Cancer Subtype Likely to Progress

**DOI:** 10.1371/journal.pone.0056761

**Published:** 2013-02-18

**Authors:** Tiziana Triulzi, Patrizia Casalini, Marco Sandri, Manuela Ratti, Maria L. Carcangiu, Mario P. Colombo, Andrea Balsari, Sylvie Ménard, Rosaria Orlandi, Elda Tagliabue

**Affiliations:** 1 Molecular Targeting Unit, Department of Experimental Oncology and Molecular Medicine, Fondazione IRCCS Istituto Nazionale dei Tumori, Milano, Italy; 2 Anatomic Pathology A Unit, Department of Pathology, Fondazione IRCCS Istituto Nazionale dei Tumori, Milano, Italy; 3 Molecular Immunology Unit, Department of Experimental Oncology and Molecular Medicine, Fondazione IRCCS Istituto Nazionale dei Tumori, Milano, Italy; 4 Dipartimento di Scienze Biomediche per la salute, Università degli Studi di Milano, Milano, Italy; Harvard School of Public Health, United States of America

## Abstract

We recently showed that differential expression of extracellular matrix (ECM) genes delineates four subgroups of breast carcinomas (ECM1, -2, -3- and -4) with different clinical outcome. To further investigate the characteristics of ECM signature and its impact on tumor progression, we conducted unsupervised clustering analyses in 6 additional independent datasets of invasive breast tumors from different platforms for a total of 643 samples. Use of four different clustering algorithms identified ECM3 tumors as an independent group in all datasets tested. ECM3 showed a homogeneous gene pattern, consisting of 58 genes encoding 43 structural ECM proteins. From 26 to 41% of the cases were ECM3-enriched, and analysis of datasets relevant to gene expression in neoplastic or corresponding stromal cells showed that both stromal and breast carcinoma cells can coordinately express ECM3 genes. In *in vitro* experiments, β-estradiol induced ECM3 gene production in ER-positive breast carcinoma cell lines, whereas TGFβ induced upregulation of the genes leading to ECM3 gene classification, especially in ER-negative breast carcinoma cells and in fibroblasts. Multivariate analysis of distant metastasis-free survival in untreated breast tumor patients revealed a significant interaction between ECM3 and histological grade (p = 0.001). Cox models, estimated separately in grade I–II and grade III tumors, indicated a highly significant association between ECM3 and worse survival probability only in grade III tumors (HR = 3.0, 95% CI = 1.3–7.0, p = 0.0098). Gene Set Enrichment analysis of ECM3 compared to non-ECM3 tumors revealed significant enrichment of epithelial-mesenchymal transition (EMT) genes in both grade I–II and grade III subsets of ECM3 tumors. Thus, ECM3 is a robust cluster that identifies breast carcinomas with EMT features but with accelerated metastatic potential only in the undifferentiated (grade III) phenotype. These findings support the key relevance of neoplastic and stroma interaction in breast cancer progression.

## Introduction

Neoplastic cells in tumors exist in a rich microenvironment composed of stromal cells, including myofibroblasts, angiogenic and inflammatory cells, and an extracellular matrix (ECM). The ECM represents a complex mixture of proteins such as proteoglycans and adhesive glycoproteins (collagens, laminins and others) that provides structural and mechanical support to cells and tissues and also influences tumor progression by architectural and signaling interaction [1]. Cell-cell and cell-matrix interactions between neoplastic cells, the surrounding stromal cells and the ECM stimulate cascades of molecular signals in and out of the cells, modulating cell behavior and contributing to tumor progression [2]–[7]. In particular, ECM remodeling is regulated jointly by stromal and epithelial cells, and the progressive change in orientation and crosslinking of collagen fibers may influence cell invasion by affecting migration along the collagen fibers or by perturbing integrin signaling [8]; [9]. Certain microenvironments can also restrict tumor progression, acting as a barrier to tumor invasion [10]. To date, several studies have emphasized the importance of interaction between neoplastic and stromal cells in *in vitro* experimental models [11]; [12].

We recently reported that breast carcinomas can be divided into four subgroups with different clinical outcome based on expression of ECM genes [13]. In the present study, we focused on ECM3, one of the subgroups that showed a highly robust cluster. We find that ECM3 genes are coordinately expressed in both neoplastic and adjacent stromal cells, and are modulated by TGFβ and hormonal stimulus. Moreover, we show that ECM3 characteristics interact with tumor grade in determining risk of distant metastases, with ECM3 grade III tumors presenting a highly significant poor prognosis in untreated patients.

## Methods

### ECM-enriched gene list

An upgraded list of ECM-enriched genes was generated essentially as described [13] using NetAffx (https://www.affymetrix.com/analysis/netaffx/). The complete list of 738 genes included genes encoding 298 membrane cell-cell matrix and cell-cell adhesion molecules, 156 extracellular molecules, 202 proteases and peptidases, 42 other enzymes (transglutaminases and enzymes involved in carbohydrate and hyaluronic acid metabolism), and 40 enzyme inhibitors (Table S1).

### Cell culture

Human breast carcinoma cell lines MDAMB231, ZR75.1 and BT474 (American Type Culture Collection) were authenticated using a panel of microsatellite markers and maintained in RPMI 1640 (Sigma-Aldrich, St. Louis, MO, USA) and DMEM (Lonza, Verviers, Belgium), respectively, supplemented with 10% (v/v) FCS (Sigma-Aldrich) and L-glutamine in a 5% CO2 humidified chamber at 37°C. ZR75.1 medium were supplemented with 1 mM sodium pyruvate, 0.1 mM non-essential amino acids, 10 µg/ml HEPES. Human GM847 fibroblasts were a kind gift from Dr. M.G. Daidone, and were maintained in DMEM 10% FCS supplemented with 2.5 mM HEPES. For estrogen deprivation, cells were grown for 72 h in the same media but containing phenol red-free RPMI and charcoal-stripped FCS; after plating in 6-well plates at an initial concentration of 6×10^5^ cells/well, cells were grown for 24 h in the presence or absence of 10 nM 17-β-estradiol (Sigma-Aldrich). Treatment with 10 ng/ml TGFβ (R&D Systems, Inc., Minneapolis, MN, USA) was performed in complete medium for 24 h.

### Immunohistochemistry

Expression of ECM3-associated SPARC and COLVI was analyzed immunohistochemically on formalin-fixed, paraffin-embedded (FFPE) tumor sections using mouse monoclonal anti-human SPARC (clone ON1-1, 10 µg/ml) (ZYMED Laboratories Inc., S. San Francisco, CA, USA) and mouse monoclonal anti-human collagen VI (clone VI-26, 5 µg/ml) (Chemicon International, Temecula, CA, USA), respectively. Antigen retrieval was carried out by heating slides for 6 min at 95°C in 5 mM citrate buffer, pH 6.0, followed by 5-min treatment at 37°C with pronase E for SPARC staining (Sigma-Aldrich) and by 1-h treatment at 37°C with hyaluronidase for collagen VI staining (Sigma-Aldrich). Immunoreactions were visualized using streptavidin-biotin-peroxidase, followed by counterstaining with Carazzi hematoxylin.

Hematoxylin and eosin (H&E)-stained FFPE sections from the ITA dataset [13] were evaluated semi-quantitatively (score 1+, 2+ and 3+) for intra-tumor fibroblast infiltration (Figure S1).

Institutional approval from our ethics committee CEI (Comitato Etico Indipendente, Fondazione IRCCS, Istituto Nazionale dei Tumori) was obtained for the conduct of the studies. Patients had agreed to the use of samples from their tumors for future investigations, although they did not provide written permission for the present study, which was performed many years after the initial diagnosis.

### Quantitative RT-PCR (qRT-PCR)

Total RNA was extracted from cell lines with Trizol ® (Invitrogen, Carlsbad, CA, USA) and from FFPE tissue with the High Pure FFPE RNA extraction kit (Roche, Basel, Switzerland) according to the manufacturers' instructions. cDNAs were reverse-transcribed from 2 ug of total RNA in a 20-µl volume with SuperScript III (Invitrogen) using oligo-dT primers for cell lines and random-hexamer primers for FFPE breast tissues. qRT-PCR was performed using Applied Biosystem Taqman assays (COL5A2: Hs00169768_m1; SPARC: Hs00277762_m1; LAMA4: Hs00158588_m1; COL1A2: Hs00164099_m1; COL6A3: Hs00915120_m1; MMP11: Hs00171829_m1) on the ABI Prism 7900HT sequence detection system (Applied Biosystems, Foster City, CA, USA). Data were normalized to GAPDH in experiments on cell lines and to the combined expression levels of 6 genes (GAPDH, ACTB, MRPL19, PUM1, PSMC4, SF3A1) [14] in experiments on FFPE breast tissues. Gene expression levels were calculated by the comparative Ct method using untreated samples as the reference in experiments on cell lines and Universal Human Reference RNA (Stratagene, La Jolla, CA, USA) in experiments on FFPE breast tissues.

### Statistical analysis

Public gene expression data from 6 previous studies based on hybridization of cDNA and oligonucleotide DNA chips [15]–[20] and the two datasets used in Bergamaschi et al [13] to define ECM classification (ITA and NOR) were used for analysis (Table S2); clinical data were accessed when available.

Strength of relationships between pairs of continuous variables (gene expression, signatures, etc.) was estimated by Pearson's correlation coefficient *r* and the null hypothesis r = 0 was tested. Absence of association between two categorical variables was tested by chi-square test. Two-sided p<0.05 was considered statistically significant.

The ECM3 sample cluster was identified using 4 unsupervised clustering algorithms: independent row-column clustering based on hierarchical clustering (IRCC-HC); independent row-column clustering based on k-means clustering (IRCC-KM); a modification of IRCC-KM based on column clustering using a selected subset (CCSS) of genes; and the Large Average Submatrix (LAS) biclustering method [21] (see Supporting Information S1). LAS biclustering of 738 ECM genes was chosen as the representative method to identify ECM3 tumors in all subsequent analyses. Agreement between ECM classification in the ITA dataset [13] using the 738 and 282 ECM-related gene lists was evaluated by the Cohen's κ coefficient [22].

The significance and stability of the ECM3 partition was evaluated: with respect to the 4 unsupervised clustering algorithms above (see Supporting Information S1); by estimating a set of internal and external cluster validation techniques (compactness, connectedness, separation of clusters); and by repeated resampling and perturbing the original datasets. Genes and samples of the ECM3 clusters identified by the 4 clustering algorithms were compared using the Jaccard similarity index [23]. Compactness and separation of cluster partitions were evaluated using the Dunn index and silhouette width [24]. Connectedness of clusters was quantified using the connectivity measure [24]. Cluster stability of ECM3 was assessed using resampling methods (bootstrapping, gene and sample subsetting, jittering, and replacement of data points by noise) [23]. Cluster stability was further investigated considering consensus clustering [25] and prediction strength [26]. Statistical significance of ECM3 clusters was estimated using the SigClust method [27].

Metagene of *COL1A2*, *COL5A2*, *SPARC* and *LAMA4* gene expression was estimated using principal component analysis (PCA). The first principal component was dichotomized using median value as cut-off. Survival functions were assessed using the Kaplan-Meier estimator, while log-rank test was used to compare survival distributions. Multivariate survival analysis was carried out using Cox proportional hazards regression models, and the effects of explanatory variables on event hazard were quantified by hazard ratios (HR) [28].

Area under the ROC curve was calculated by nonparametric ROC analysis [29] using the roctab command of Stata 11 [30].

Classification into the five molecular intrinsic subtypes (normal-like, ERBB2, lumA, lumB and basal-like) was assessed as described [13].

Gene set enrichment analysis to assess ECM and grade functional association was performed using GSEA v2.07 [31]. Genes represented by more than one probe were collapsed to the probe with the maximum value using the Collapse Dataset tool. Gene set permutation type was applied 1000 times. The 193 cancer-related gene set database reported in Table S3 is an upgrade of a database previously described [32].

## Results

### ECM3 characterizes a breast cancer molecular subtype

To test the robustness of our previously reported breast carcinoma classification according to ECM gene profile in the ITA and NOR cohorts [13], we studied the expression profile of ECM-related genes in 6 additional independent datasets of invasive breast tumors from different platforms, counting 643 samples overall (Table S2). To minimize the possibility that different platforms used in profiling invasive breast tumors are differentially efficient in analyzing ECM molecular characteristics, we analyzed an upgraded version of the ECM-related gene list which includes 738 instead of the previously used 282 ECM genes (Table S1). Agreement between ECM classification in the ITA dataset using the 738 and 282 ECM-related gene lists was highly significant (p<0.0001, Cohen's κ coefficient = 0.85).

Of the ECM1, -2, -3, and -4 breast tumor subgroups defined in our previous analysis, we focused on ECM3 because it was clearly detectable in every dataset analyzed.

Comparison of gene and sample partitions generated by the clustering algorithms LAS, IRCC-HC, IRCC-KM and CCSS indicated good agreement between clusters of ECM3 tumors (median Jaccard index = 0.76; range = 0.42–0.96) and high homogeneity in the composition of ECM3 gene clusters (median Jaccard index = 0.53; range = 0.30–0.84). Evaluation of compactness, connectedness, and separation of cluster partitions showed that the average silhouette of ECM3 clusters in the 6 datasets ranged from 0.26 to 0.38 (median = 0.36), the Dunn indices ranged from 0.19 to 0.53 (median = 0.35) and connectivity ranged from 7.4 to 53.6 (median = 17.0). The average clusterwise Jaccard index ranged from 0.57 to 0.97 for bootstrapped data, from 0.87 to 1.00 for jittered data, from 0.82 to 0.99 for subsetted data and from 0.67 to 0.97 for data partially replaced by noise. In each dataset, ECM3 clusters were highly significant (p<0.001) (see Supporting Information S1). Together, these validation results clearly indicated that ECM3 clusters are stable, since changing clustering algorithms and perturbing observed data did not significantly alter the structure of the set of ECM3 tumors and of ECM3 genes.

**Figure 1:**
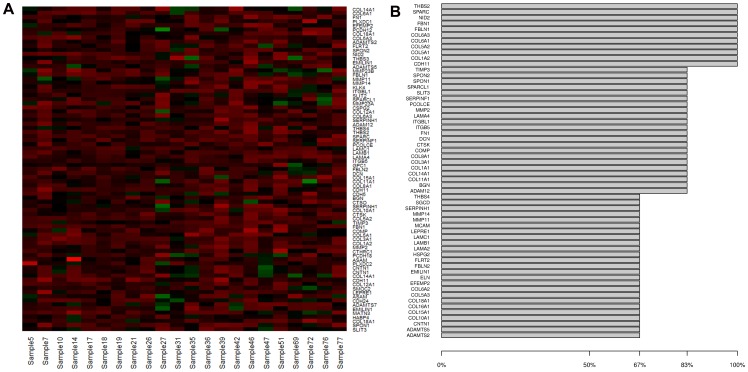
ECM3 gene pattern in breast carcinomas. (A) Expression profile of LAS biclustering in breast tumors from the Van't Veer dataset [15]. (B) Frequency distribution of most influential ECM3 genes in 6 datasets ([15]–[20]), tumor clustering and gene importance estimation.

According to the LAS clustering method, from 26 to 41% of the cases were ECM3-enriched. Figure 1A shows a representative example of the ECM3 cluster in the Van't Veer dataset [15]. Analysis of datasets for which clinical data were available (Sotiriou et al [20], Desmedt et al [19], Chin [16] datasets) revealed a consistent association between ECM3 features and ER positivity and histological grades I and II in all datasets, but no association with tumor size or age (Table 1).

**Table 1:** Frequency of clinico-pathological characteristic in patients according to ECM3 classification.

	Chin		Sotiriou		Desmedt	
	n° ECM3/tot (%)	p value*	n° ECM3/tot (%)	p value*	n° ECM3/tot (%)	p value*
Grade I–II	21/54 (39)		48/79 (61)		37/113 (33)	
Grade III	12/60 (20)	0.0381	3/28 (11)	<0.0001	14/83 (17)	0.0137
ER pos	29/75 (39)		45/84 (54)		42/134 (31)	
ER neg	6/43 (14)	0.0061	6/40 (15)	<0.0001	10/64 (16)	0.024
Size ≤2	15/47 (32)		32/74 (43)		29/102 (28)	
Size >2	20/69 (29)		19/49 (39)		23/96 (24)	
Age <50	14/50 (28)		21/53 (40)		33/142 (23)	
Age ≥50	21/67 (31)		30/71 (42)		19/56 (34)	

* Evaluated by Fisher exact test.

To identify an objective and reproducible set of overexpressing genes defining ECM3 tumors across different studies and platforms, we examined ECM3 gene clusters obtained using the LAS method and identified 58 genes most relevant in determining the ECM3 cluster (Figure 1B). Consistent with our previous study [13], *SPARC* was a prominent gene in ECM3 gene classification of breast tumors. In addition, Pearson's correlations between expression of *SPARC* and 738 ECM-enriched genes in the same datasets identified a set of ECM genes significantly correlated with *SPARC* (median r>0.5) (Figure S2) that included 89% of the 58 ECM3 genes identified by the LAS method. Most of the ECM3 genes (43/58) encode structural ECM proteins, 7 genes encode proteases (*MMP2*, *ADAM12*, *ADAMTS2*, *ADAMTS5*, *CTSK*, *MMP11*, *MMP14*), 3 encode peptidase inhibitors (*TIMP3*, *SERPINF1*, *SERPINH1*) and 5 cell adhesion molecules (*CDH11*, *SGCD*, *CNTN1*), including two integrin β chains (*ITGB5*, *ITGBL1*).

**Figure 2:**
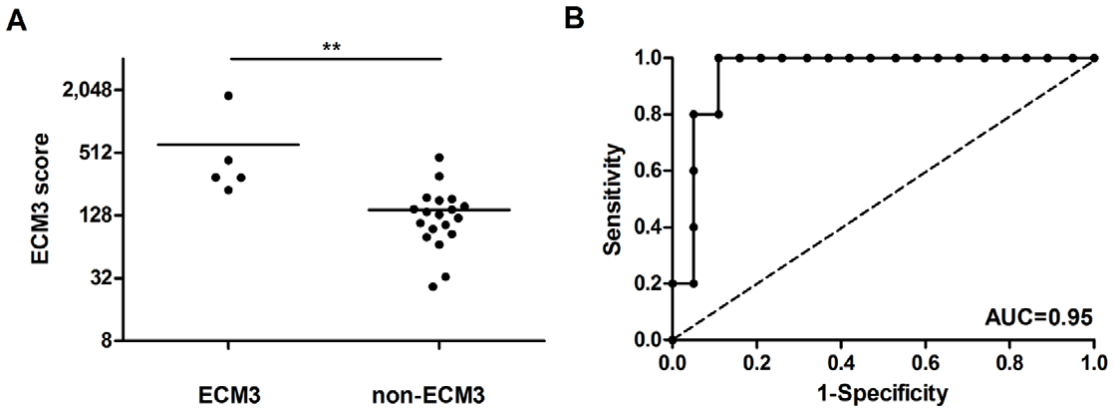
ECM3 classification of breast carcinomas by qRT-PCR. (A) qPCR analysis of ECM3 score derived from the average expression levels of *COL1A1*, *COL5A2*, *SPARC* and *LAMA4* genes on FFPE specimens of 24 breast tumors of the ITA cohort [13] classified as ECM3 or non-ECM3 by unsupervised clustering. ** p<0.01 in unpaired t-test. (B) Receiver-operator characteristics (ROC) curve for expression of ECM3 genes in the ECM3 tumor subgroup.

To further test for the coordinated expression of ECM3 genes in breast carcinomas, we randomly selected groups of 4 genes within the 58 ECM3 genes and estimated the area under the ROC curve (AUC) in all datasets. The median AUC ranged from 0.92 (IQR = 0.88–0.95) to 0.98 (IQR = 0.95–0.99), indicating a good performance of these 4-gene sets in singling out ECM3 cases. Moreover, the average expression of the gene set *COL1A1*, *COL5A2*, *SPARC* and *LAMA4*, with AUC greater than the 75th percentile of the randomly selected 4-gene AUCs in almost all datasets, was significantly higher in tumor specimens classified in microarray analysis of ITA dataset [13] as ECM3 in comparison with non-ECM3 tumors when tested by qPCR using RNA from FFPE sections (p = 0.005, Figure 2A). The AUC generated (AUC = 0.95, 95%CI = 0.86–1.00) confirmed the qPCR results, indicating the good performance of this score in discriminating ECM3 tumors (Figure 2B).

### Neoplastic cells participate in ECM3 gene expression

**Figure 3:**
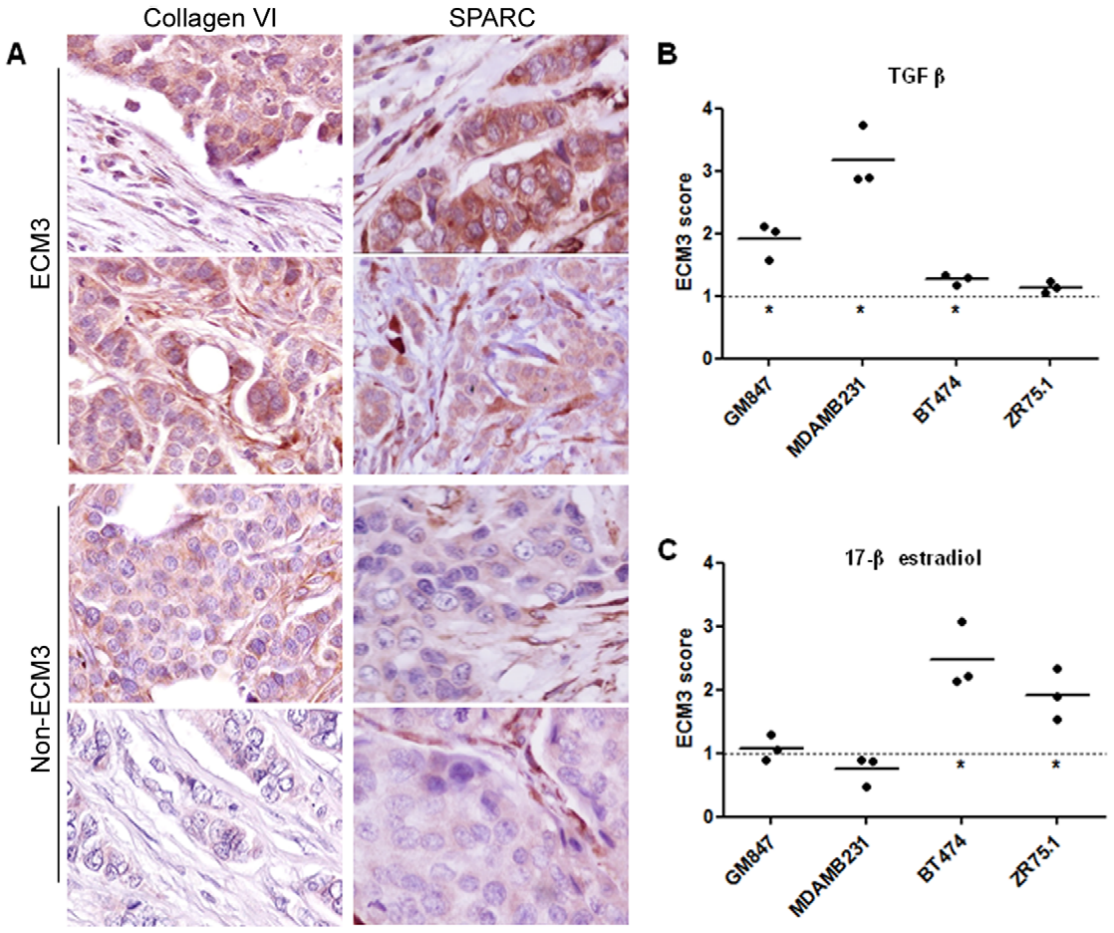
ECM3 gene expression in tumor cells. (A) Representative immunohistochemical staining for Collagen VI and SPARC in ECM3 and non-ECM3 breast tumors. Magnification: ×400. (B,C) Fold-increase in ECM3 score expression as assessed by qRT-PCR after treatment with TGFβ (B) and 17β-estradiol (C) in breast carcinoma and fibroblast cell lines. Dotted line indicates average ECM3 expression in untreated cell lines. *p<0.05 in unpaired t-test vs untreated cells.

Although previously identified stromal signatures reportedly derive from reactive stroma [33]; [34], we found no significant increase in the proportion of stromal vs epithelial cells in ECM3 compared to non-ECM3 carcinomas from 29 primary breast tumors of the ITA cohort [13] as assessed based on a semi-quantitative intra-tumor fibroblast infiltration score (score 1+: 37% vs 40%; score 2+: 44% vs 38% and score 3+: 19% vs 22%). Immunohistochemical analysis of FFPE sections from these tumors revealed the presence of the ECM3 representative markers SPARC and Collagen VI (Figure 3A; note clear cytoplasmic staining in neoplastic cells) in a significantly higher percentage of ECM3 samples as compared with non-ECM3 samples (100% vs 32%, p = 0.008 and 75% vs 35%, p = 0.050, respectively). Intra-tumoral fibroblast cells scored SPARC- and Collagen VI-positive in ECM3 and in the majority of non-ECM3 carcinomas (Figure 3A). LAS analysis of ECM3 gene expression in the Ma dataset [18], which contains expression data from whole breast tumor tissue samples as well as from the corresponding laser-captured microdissected breast tumor cells, showed that 7 of 18 (39%) tumors were classified as ECM3 in both datasets. Moreover, analysis of the Boersma dataset [35], which comprises gene expression data for both neoplastic and corresponding stromal cells, showed that the ECM3 genes were coordinately expressed in 14 of 48 (29%) microdissected neoplastic cell samples (6 ER-positive, 6 ER-negative and 2 not determined) and in 12 of 47 (25.5%) stroma samples, of which 6 are matched. Both clusters were stable according to stability parameters (see Supporting Information S1). The average expression level of ECM3 genes in microdissected neoplastic and stromal cells belonging to the ECM3 cluster was comparable, while non-ECM3 samples revealed significantly higher expression levels of ECM3 genes in stromal compared to epithelial tumor cells (Figure S3). Thus, while high expression of these genes is characteristic of stromal cells, the neoplastic cells in ECM3 tumors also coordinately express these genes and may impact on the tumor total expression levels.

Based on the high frequency of ER-positive tumors in the ECM3 cluster and on the known TGFβ induction of ECM genes in fibroblasts [36]; [37], we carried out qPCR analysis of 6 representative ECM3 genes (*COL1A1*, *COL5A2*, *SPARC*, *LAMA4*, *COL6A3* and *MMP11*) in ER-positive (ZR75.1 and BT474) and -negative (MDAMB231) breast carcinoma cell lines as well as on GM847 fibroblasts to investigate possible pathways involved in ECM3 gene expression in tumor cells. While no increase was found in *COL5A2* expression after treatment with β-estradiol or in *COL5A2* and *LAMA4* after treatment with TGFβ in ER-positive and -negative breast carcinoma cells, respectively, all other gene expression levels increased significantly after treatments (Figure S4). The ECM3 score was significantly enhanced in ER-negative and -positive breast carcinoma cells upon treatment with TGFβ (Figure 3B; MDAMB231 cells, p = 0.016; BT474 cells, p = 0.045) or β-estradiol (Figure 3C; BT474 cells, p = 0.041; ZR75.1 cells, p = 0.018), while only TGFβ stimulation led to increased ECM3 gene expression in fibroblasts (Figure 3B; GM847 cells, p = 0.034), in which basal expression levels of ECM3 genes are 30- to 300-fold higher than in breast carcinoma cell lines (data not shown).

### ECM3 impacts tumor progression according to tumor differentiation status

**Figure 4:**
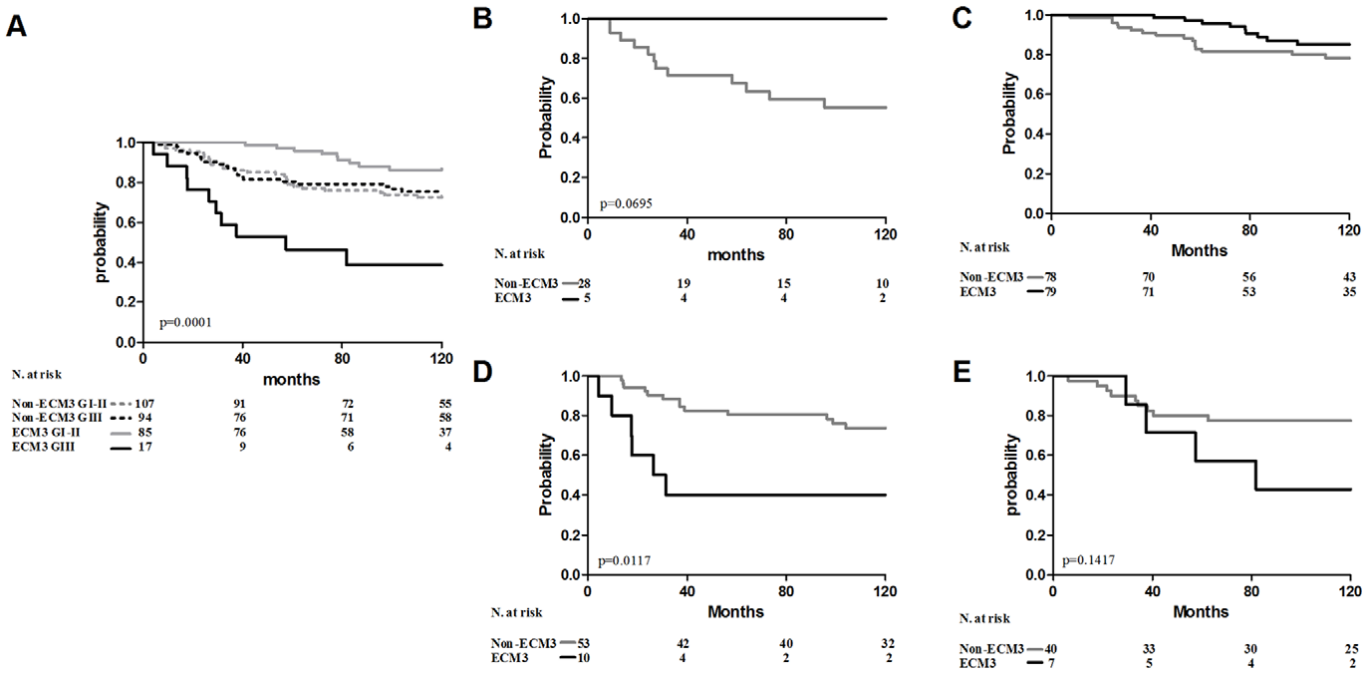
ECM3 prognostic significance in relation to differentiation status. (A) Association between ECM3 grade III (ECM3 GIII, solid black line), non-ECM3 grade III (non-ECM3 GIII, dotted black line), ECM3 grade I–II (ECM3 GI-II, solid grey line) and non-ECM3 grade I–II (non-ECM3 GI-II, dotted grey line) with DMFS in untreated patient joined datasets. (B–E) Association between ECM3 (black line) and non-ECM3 (grey line) with DMFS in ER-negative grade I–II (B), ER-positive grade I–II (C), ER-negative grade III (D) and ER-positive grade III (E) subgroups. p-values by log rank test.

Univariate analysis of metastasis-free survival (DMFS) indicated no prognostic significance for ECM3 (data not shown) in the Sotiriou [20] and Desmedt [19] datasets of node-negative, untreated patients. Multivariate analysis of the joined dataset evidenced a significant interaction between ECM3 and grade (p = 0.001) (Table 2), indicating that ECM3 has a different clinical significance in grade I–II vs grade III. The probability of developing metastases in a 10-year follow-up was 14% for ECM3 differentiated tumors (grade I–II) as compared to 61% for ECM3 grade III carcinomas and about 25% for all non-ECM3 tumors (Figure 4A). Comparable results were obtained using a metagene of the 4 genes used in qPCR (*COL1A1*, *COL5A2*, *SPARC* and *LAMA4*) (Figure S5). Cox models estimated separately in grade I–II and grade III tumors indicated the strong and significant association of ECM3 with worse survival probability in grade III tumors (HR = 3.0, 95% CI = 1.3–7.0, p = 0.0098), whereas no significant association of ECM3 with survival was found in grade I–II (HR = 1.01; p = 0.9693) (Table S4). In differentiated tumors, better survival was significantly associated with ER positivity and small tumor size (Table S4). The clinical significance of ECM3 on DMFS was investigated separately in the ER-positive and ER-negative groups of the joined dataset. In ER-negative patients, ECM3 was moderately associated with better survival in differentiated tumors (log-rank test: p = 0.0695) (Figure 4B) and was a significant predictor of distant metastasis in grade III tumors (HR = 4.12, 95%CI = 1.54–11.05, p = 0.0049) (Figure 4D and Table S5). In ER-positive patients, ECM3 and recurrence risk were modestly associated only in grade III patients (HR = 2.5, 95%CI = 0.74–8.48, p = 0.141) (Figure 4E and Table S5).

**Table 2:** Multivariate proportional hazards analysis of metastasis-free survival in untreated patients.

	Sotiriou		Desmedt		Joined	
Variable	Hazard Ratio (95% CI)*	p value	Hazard Ratio (95% CI)	p value	Hazard Ratio (95% CI)	p value
Size	2.1 (1.2–3.5)	0.0074	1.4 (1.0–1.8)	0.0376	1.5 (1.1–1.9)	0.0029
Age	1.0 (1.0–1.1)	0.0388	1.0 (1.0–1.1)	0.4059	1.0 (1.0–1.1)	0.0768
ER pos	1.0 (0.4–2.6)	0.9368	0.5 (0.3–0.9)	0.0139	0.6 (0.4–1.0)	0.0515
ECM3 Grade III	42.5 (5.2–345.1)	0.0004	3.7 (1.2–11.0)	0.0219	5.5 (2.0–15.1)	0.0010
ECM3	0.3 (0.1–0.8)	0.0219	1.0 (0.5–2.1)	0.9478	0.8 (0.4–1.7)	0.6081
Grade III	0.2 (0.1–0.7)	0.0108	0.6 (0.3–1.1)	0.1024	0.6 (0.3–1.1)	0.0792

* CI = confidence interval.

**Figure 5:**
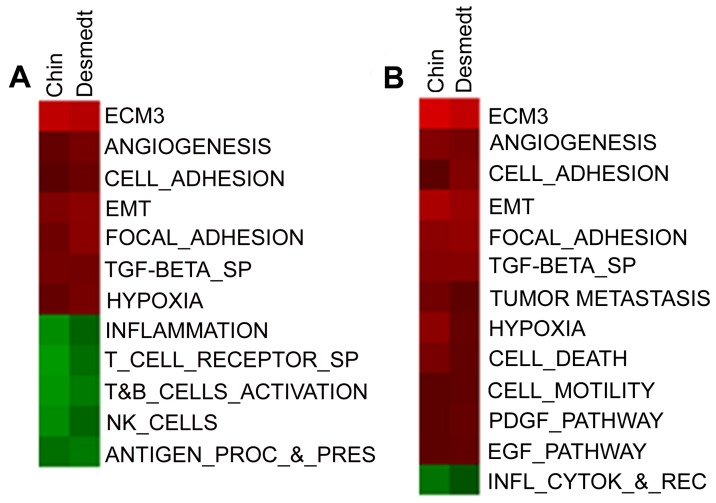
Molecular characteristics of breast carcinomas according to ECM and grade classification by Gene Set Enrichment Analysis (GSEA). Heatmap shows the normalized enrichment scores (NES) of gene sets significantly enriched at p<0.05 and FDR<25% in ECM3 grade III (A) and grade I–II (B) tumors of Desmedt [19] and Chin [16] datasets.

To explore the ability of ECM-related genes to provide biologically meaningful insights according to tumor cell differentiation grade, we performed GSEA [31] in two datasets (Desmedt [19] and Chin [16]) according to ECM and grade classification. Comparison of gene expression in ECM3 vs non-ECM3 grade III tumors revealed significant enrichment for cell adhesion, focal adhesion, EMT, TGFβ and hypoxia genes in the ECM3 group and for genes representative of NK, T or B cells in the non-ECM3 group (Figure 5A). Among grade I–II ECM3 tumors, no differences in lymphoid cell-associated pathways vs non-ECM3 tumors were detected, whereas cell and focal adhesion and EMT genes were also upregulated in this subset of ECM3 tumors (Figure 5B). Comparison between ECM3 and non-ECM3 grade III tumors in Desmedt and Sotiriou datasets with respect to clinico-pathological features and intrinsic molecular subtype, assigned as previously reported [13], revealed no significant differences. Indeed, while ECM3 grade III tumors were enriched in basal-like (5/17) and ERBB2 (6/17) subtypes, a large proportion of basal tumors (39/94) presented non-ECM3 features. Notably, ECM3 grade III patients relapsed more frequently than did non-ECM3 grade III patients independent of intrinsic molecular subtype (Figure S6). Moreover, analysis of clinico-pathological features available revealed no differences between ECM3 and non-ECM3 grade III tumors (Table S6).

## Discussion

ECM3 is one of four main groups of breast carcinomas previously identified according to the pattern of ECM gene expression in two different datasets [13]. Our present analysis of 6 independent datasets including more than 600 samples identified ECM3 as a stable tumor partition in each series analyzed. The high stability of the ECM3 cluster is supported by ECM3 tumor classification using four different unsupervised clustering algorithms and by repeated resampling and/or perturbing the original datasets, with analyses indicating that ECM3 is a robust cluster identifying a breast carcinoma molecular category with high concordance in genes defining this cluster.

The majority of the 58 genes found relevant in determining the ECM3 cluster encode macromolecules involved in the maintenance of connective tissue (collagens, laminins, and matrix-associated proteins) and proteolytic enzymes (MMPs and ADAMs). The coordinate overexpression of all of these genes strongly suggests the presence of a tumor microenvironment that undergoes extensive remodeling in the structural organization of ECM components. Moreover, enriched expression of molecules involved in focal adhesion in these tumors strongly supports interplay between tumor cells and the microenvironment.

Although stromal fibroblasts have been described as the main contributors to transcriptional signatures enriched in stroma-derived structural ECM genes [33]; [34]; [38], as supported by our *in vitro* finding of higher basal levels of ECM3 gene expression in fibroblasts than in neoplastic cells, our present data indicate that ECM3 genes are coordinately expressed by stromal as well as neoplastic cells in ECM3 tumors and that both cell types contribute in regulating the properties of their microenvironment. Furthermore, the lack of any difference between the neoplastic and stromal counterpart in ECM3 gene levels of ECM3 tumors in the microdissected tumor Boersma dataset, as well as the comparable proportion of stromal and neoplastic cells in both ECM3 and non-ECM3 tumors, point to the significant impact of neoplastic cells on total expression of these genes in ECM3 tumors. Our *in vitro* experiments with ER-positive breast carcinoma cell lines BT474 and ZR75.1 showed that β-estradiol provided a signal for induction of ECM3 gene expression. Normal human mammary cells coordinately express matrix proteins mainly belonging to the ECM3 cluster during hormone-regulated phases of mammary gland development, i.e., branching, alveologenesis and lactation [39]. While ER-positive breast carcinoma cells producing these ECM3 molecules appear to be highly hormone-dependent, as indicated by the significant correlation between ECM3, ER expression and grade I–II found in human tumor samples, the presence of ER-negative tumors in the ECM3 cluster of microdissected tumor samples indicates that breast carcinoma cells can also produce ECM3 molecules in the absence of hormonal activation. Accordingly, TGFβ induced *in vitro* upregulation of the genes leading to ECM3 gene classification, especially in ER-negative breast carcinoma cells. The different regulation of ECM3 genes in ER-positive versus ER-negative cells might reflect the dependence on diverse signals in dictating ECM3 constitution based on tumor cell molecular characteristics. Moreover, based on the lack of significantly increased expression of *COL5A2* and *LAMA4* genes in *in vitro* tumor cells after TGFβ or β-estradiol treatment, we speculate that signals other than TGFβ or β-estradiol contribute in regulating ECM3 genes. The co-regulation of ECM3 genes in both epithelial and stromal cells might rest in the fact that these genes are regulated by the same stimuli in both cell types. Indeed, TGFβ has been described to regulate some ECM3 gene expression in fibroblasts, and hormones reportedly activate fibroblasts and inflammatory cells [40]–[42], raising the possibility that they too induce ECM3 gene expression in stromal cells.

ECM3 was strongly and significantly associated with worse survival probability only in grade III tumors. In grade I–II tumors, the association of ECM3 with better prognosis in Kaplan-Meier analysis could have resulted from tumor ER positivity and small size, the two factors found significantly associated with better prognosis in this subgroup by Cox models. Thus, it is possible that in well-differentiated breast carcinoma cells, the production of ECM3 molecules driven mainly by the ER signal acts to slow tumor progression. By contrast, in undifferentiated grade III tumors, which are mainly independent of the hormone pathway, the TGFβ signal might support the ECM3 environment and contribute to tumor progression. The well-known Janus role of TGFβ in tumor progression based on tumor stage [43] might also explain the reversion of ECM3 from a pro- to an anti-tumoral effect. Indeed, some ECM3 genes are TGFβ-regulated ([44] and present results, see Figure 3), and the TGFβ pathway is enriched in ECM3 as compared with non-ECM3 tumors. In addition a strict correlation exists between SPARC, the core gene of our signature, and TGFβ, since SPARC is directly involved in the regulation of the TGFβ signaling cascade and SPARC is regulated by TGFβ [45]. In this context, expression of SPARC was found to inhibit epithelial cell proliferation, in part through stimulation of TGFβ signaling [46], while in MCF7 breast carcinoma cells expressing oncogenes, SPARC induced cell motility and aggressiveness [47]. Overall, further studies are clearly needed to determine whether ECM3 molecules simply mirror those modulated by TGFβ, or whether they actively participate in tumor progression by modulating interactions among neoplastic cells and the tumor environment. Nevertheless, the coordinated expression of ECM3 genes in breast carcinomas, confirmed by the good performance of the average expression profile of 4 genes (*COL1A1*, *COL5A2*, *SPARC* and *LAMA4*) in discriminating ECM3 tumors and evaluating their prognosis, argues for their active involvement in the behavior of breast carcinomas. Indeed, upregulation of ECM3 genes encoding macromolecules involved in the maintenance of connective tissue (e.g., collagens, laminins, etc.) and encoding matrix-associated proteins (e.g., SPARC) could result in a rigid network of stiff cross-linked matrix fibers which impedes migration of well-differentiated (grade I–II) tumor cells [48], whereas the same matrix fibers may support a valid substrate for migration and dissemination of tumor cells with aggressive features (grade III), consistent with the ability of cancer cells to migrate rapidly on collagen fibers in areas enriched in collagen [49].

Proliferation genes that dominate and integrate most prognostic information in the majority of prognostic signatures currently studied in breast cancer [50]; [51] are highly expressed in grade III tumors, but probably cannot alone explain the high aggressiveness of some breast carcinomas. Indeed, the grade III tumors with the worst prognosis were those enriched in EMT, TGFβ and hypoxia gene expression, all significantly up-modulated in ECM3 compared with non-ECM3 tumors. The enrichment in EMT gene expression was also present in ECM3 differentiated (grade I–II) carcinomas; however, in this case, there was no association with worse prognosis. In addition, both non-ECM3 grade III and grade I–II carcinomas showed comparable DMFS. This finding might rest in an accelerated acquisition of full metastatic potential only in tumors combining undifferentiated, proliferating (grade III) features with an ECM3 microenvironment and might explain why the ECM3 signature has a significant prognostic role only in progression of undifferentiated carcinomas and not in grade I–II tumors, where ECM3 enrichment is likely due to responsiveness of these breast tumors to the hormone pathway.

In addition, a prominent role for the host immune response in hampering non-ECM3 and grade III tumor progression must be considered, based on the significant enrichment for genes representative of lymphoid infiltration in these tumors. Accordingly, the lack of SPARC in the environment was found to impair collagen deposition and accelerate the onset of T-cell priming [52], whereas TGFβ was shown to induce an immunosuppressive effect [53]. Furthermore, the absence of significant differences between ECM3 and non-ECM3 grade III tumors according to clino-pathological features and intrinsic molecular subtypes suggest the value of ECM3 classification in providing additional information on the biology of tumor progression. Notwithstanding the small sample size used in our study to assess the impact of ECM3 on breast cancer progression, and the weakness of some published breast cancer outcome signatures when tested in meta-analyses [54], our results, indicating the significance of interaction between tumor and stromal features in influencing tumor progression, might provide the proof-of-concept that cross-talk between transformed cells and the microenvironment, to date extensively described in preclinical models [55], is a key force in conditioning breast carcinoma evolution in patients. Further studies are clearly needed to understand the biological implications of the ECM3 signature in differentiated versus undifferentiated breast carcinomas, and to verify the good performance of the 4-gene score (*COL1A2*, *COL5A2*, *SPARC*, *LAMA4*) in discriminating ECM3 tumors, thus validating the role of ECM3 molecular marker. If such studies confirm the relevance of ECM3 gene expression in breast carcinoma progression, new therapies that target the ECM3 microenvironment and block its communication with transformed cells could represent a promising tool to halt progression of aggressive, undifferentiated (grade III) tumors.

## Supporting Information

Figure S1 **Intra-tumor fibroblast infiltration score.** Representative H&E image of each score value is shown; magnification: ×200.
(TIF)

Figure S2 **ECM genes significantly correlated with** ***SPARC*** **expression.**
(TIF)

Figure S3 **ECM3 score expression in microdissected breast carcinomas.** Average expression level of 4 representative ECM3 genes (*COL1A1*, *COL5A2*, *SPARC* and *LAMA4*) in microdissected neoplastic cell samples (N) and in microdissected stromal cell samples (S). **p<0.01, ***p<0.001, in unpaired t-test.
(TIF)

Figure S4 **ECM3 genes expression in tumor cells.** Fold-increase in expression of representative ECM3 genes (*COL1A1*, *COL5A2*, *SPARC*, *LAMA4*, *COL6A3* and *MMP11*) as assessed by qRT-PCR after treatment with TGFβ (A) and 17β-estradiol (B) in breast carcinoma and fibroblast cell lines. Dotted line indicates ECM3 gene expression in untreated cell lines. *p<0.05, **p<0.01, ***p<0.001, in unpaired t-test vs untreated cells.
(TIF)

Figure S5 **ECM3 prognostic significance in relation to differentiation status using the ECM3 score.** Association between ECM3 (black line) and non-ECM3 (grey line) with DMFS in grade I–II (A) and grade III (B) patients of joined dataset; ECM3 was determined as higher expression level of a *COL1A2*, *COL5A2*, *SPARC* and *LAMA4* metagene with respect to metagene median expression level. p-values by log-rank test.
(TIF)

Figure S6 **Intrinsic molecular classification of grade III tumors according to ECM classification.**
(TIF)

Table S1 **ECM gene list.**
(XLS)

Table S2 **Datasets used in this study.**
(XLS)

Table S3 **Gene Set Enrichment Analysis (GSEA) of ECM3 versus non-ECM3 tumors and cancer-related gene set database.**
(XLS)

Table S4 **Multivariate proportional hazards analysis of metastasis-free survival in untreated patients according to tumor grade.**
(DOC)

Table S5 **Multivariate proportional hazards analysis of metastasis-free survival in untreated grade III patients according to ER status.**
(DOC)

Table S6 **Frequency of clinico-pathological features in patients with grade III breast carcinomas according to ECM3 classification.**
(DOC)

Supporting Information S1 **ECM3 cluster stability analysis.**
(PDF)
