# Supplementary figures and images for: Neoplastic and Stromal Cells Contribute to an Extracellular Matrix Gene Expression Profile Defining a Breast Cancer Subtype Likely to Progress
Source: PLoS One. 2013 Feb 18;8(2):e56761. doi: 10.1371/journal.pone.0056761 (PMC3575489; doi:10.1371/journal.pone.0056761)

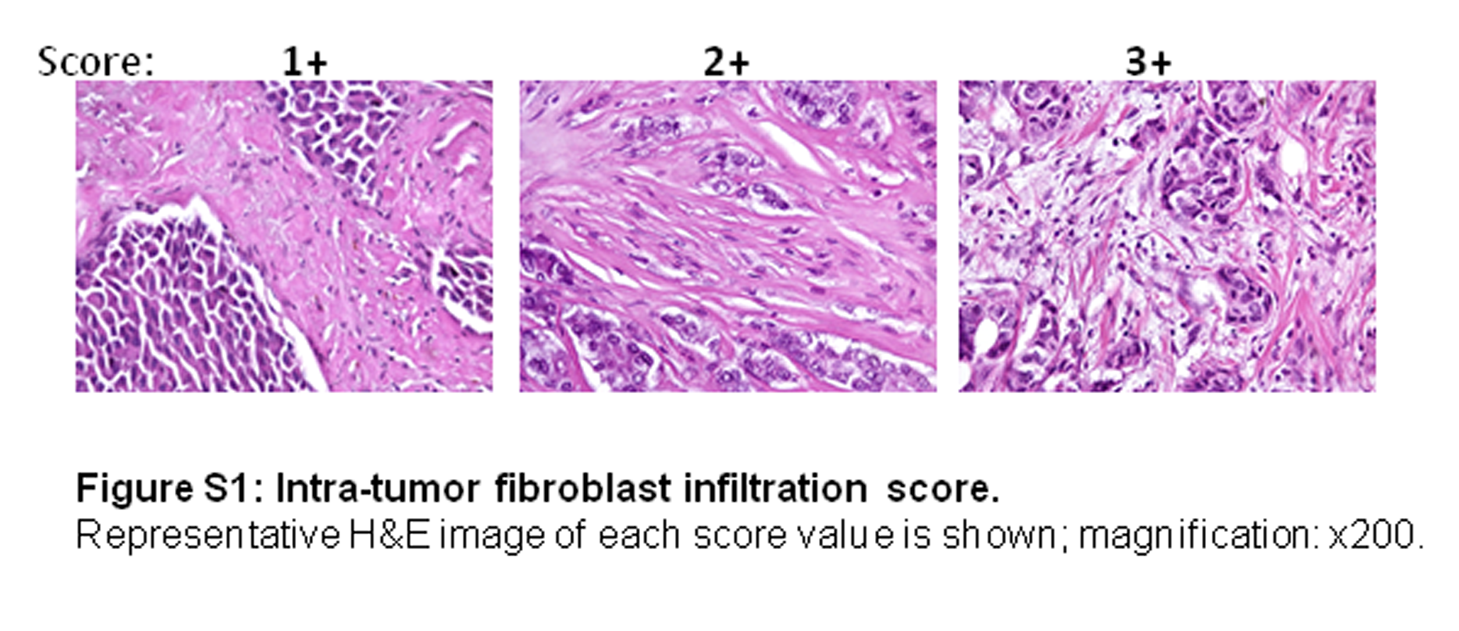

Supplement: Figure S1 — Intra-tumor fibroblast infiltration score. Representative H&E image of each score value is shown; magnification: ×200. (TIF) [file pone.0056761.s001.tif]

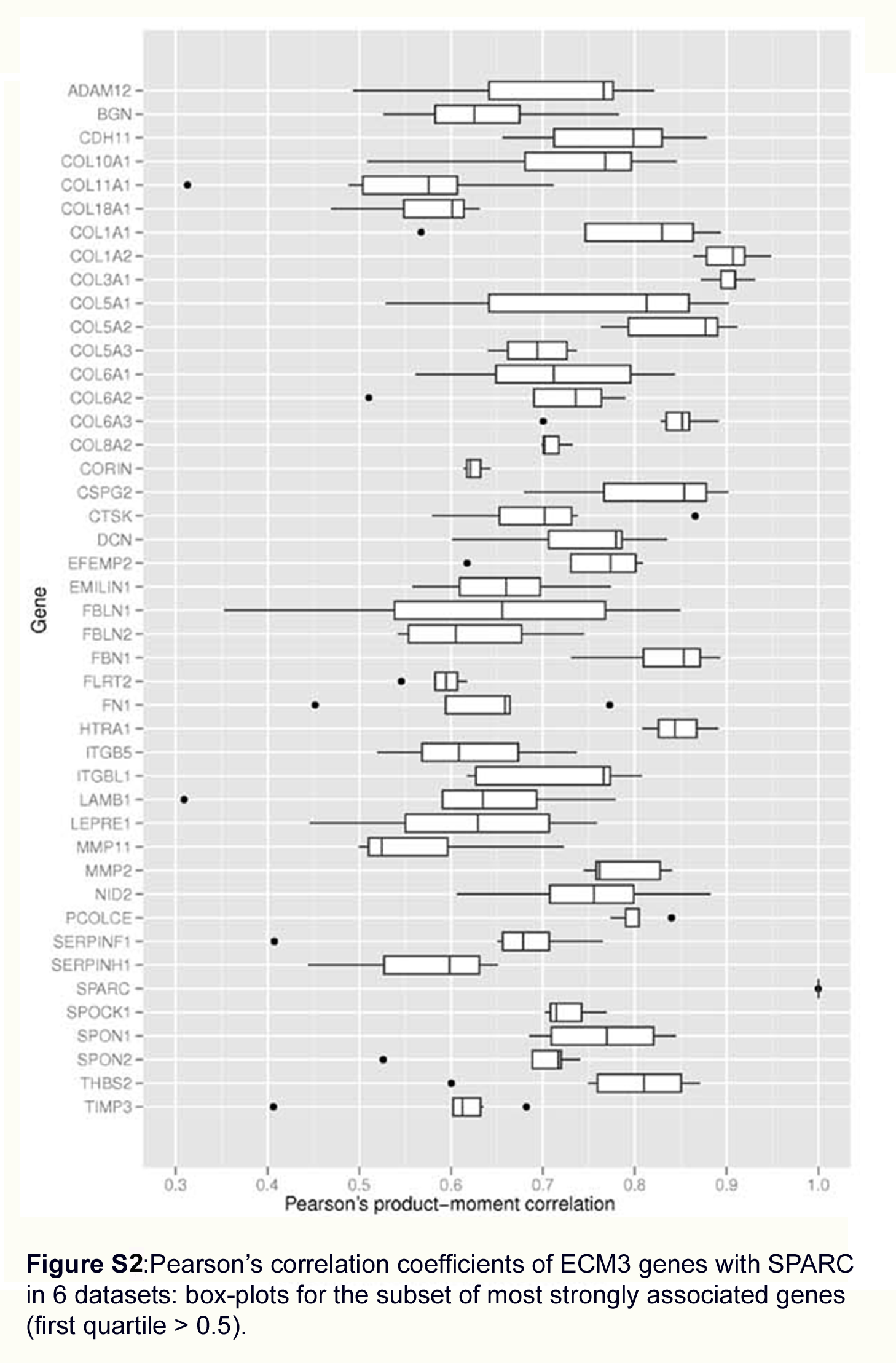

Supplement: Figure S2 — ECM genes significantly correlated with SPARC expression. (TIF) [file pone.0056761.s002.tif]

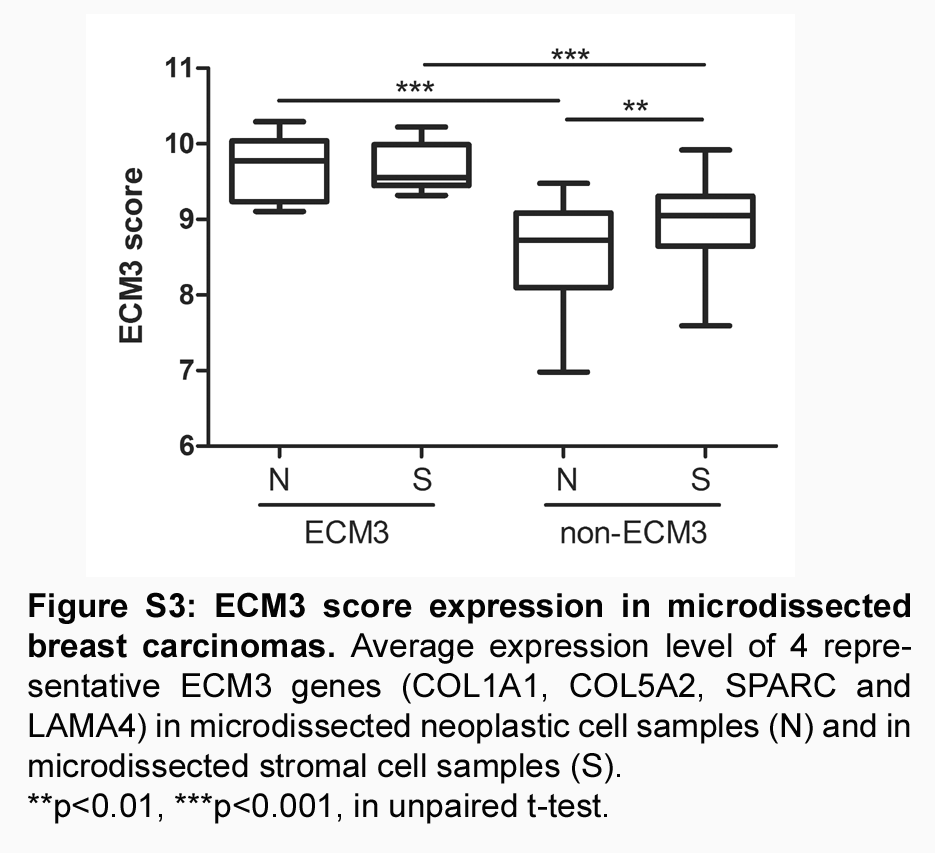

Supplement: Figure S3 — ECM3 score expression in microdissected breast carcinomas. Average expression level of 4 representative ECM3 genes (COL1A1, COL5A2, SPARC and LAMA4) in microdissected neoplastic cell samples (N) and in microdissected stromal cell samples (S). **p<0.01, ***p<0.001, in unpaired t-test. (TIF) [file pone.0056761.s003.tif]

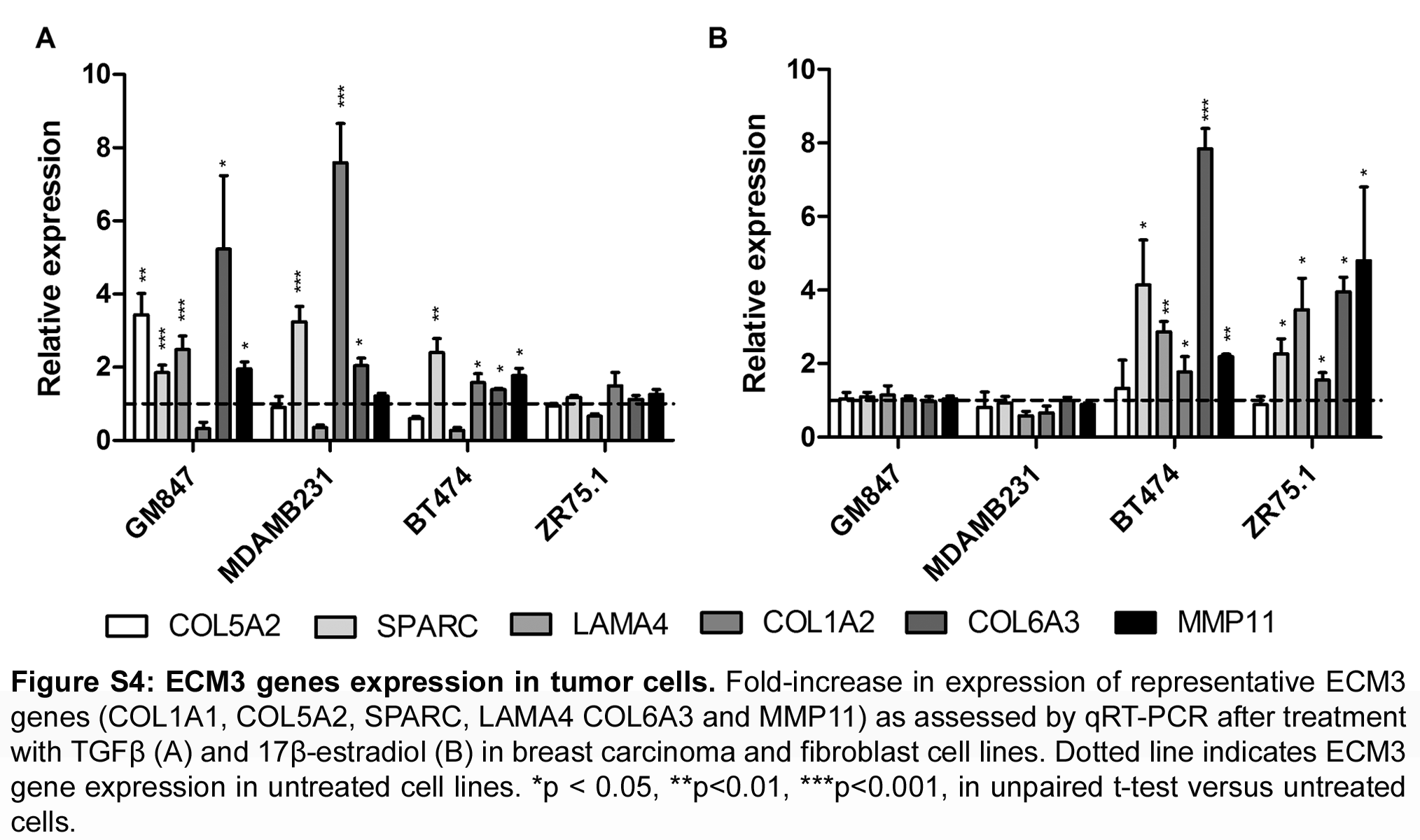

Supplement: Figure S4 — ECM3 genes expression in tumor cells. Fold-increase in expression of representative ECM3 genes (COL1A1, COL5A2, SPARC, LAMA4, COL6A3 and MMP11) as assessed by qRT-PCR after treatment with TGFβ (A) and 17β-estradiol (B) in breast carcinoma and fibroblast cell lines. Dotted line indicates ECM3 gene expression in untreated cell lines. *p<0.05, **p<0.01, ***p<0.001, in unpaired t-test vs untreated cells. (TIF) [file pone.0056761.s004.tif]

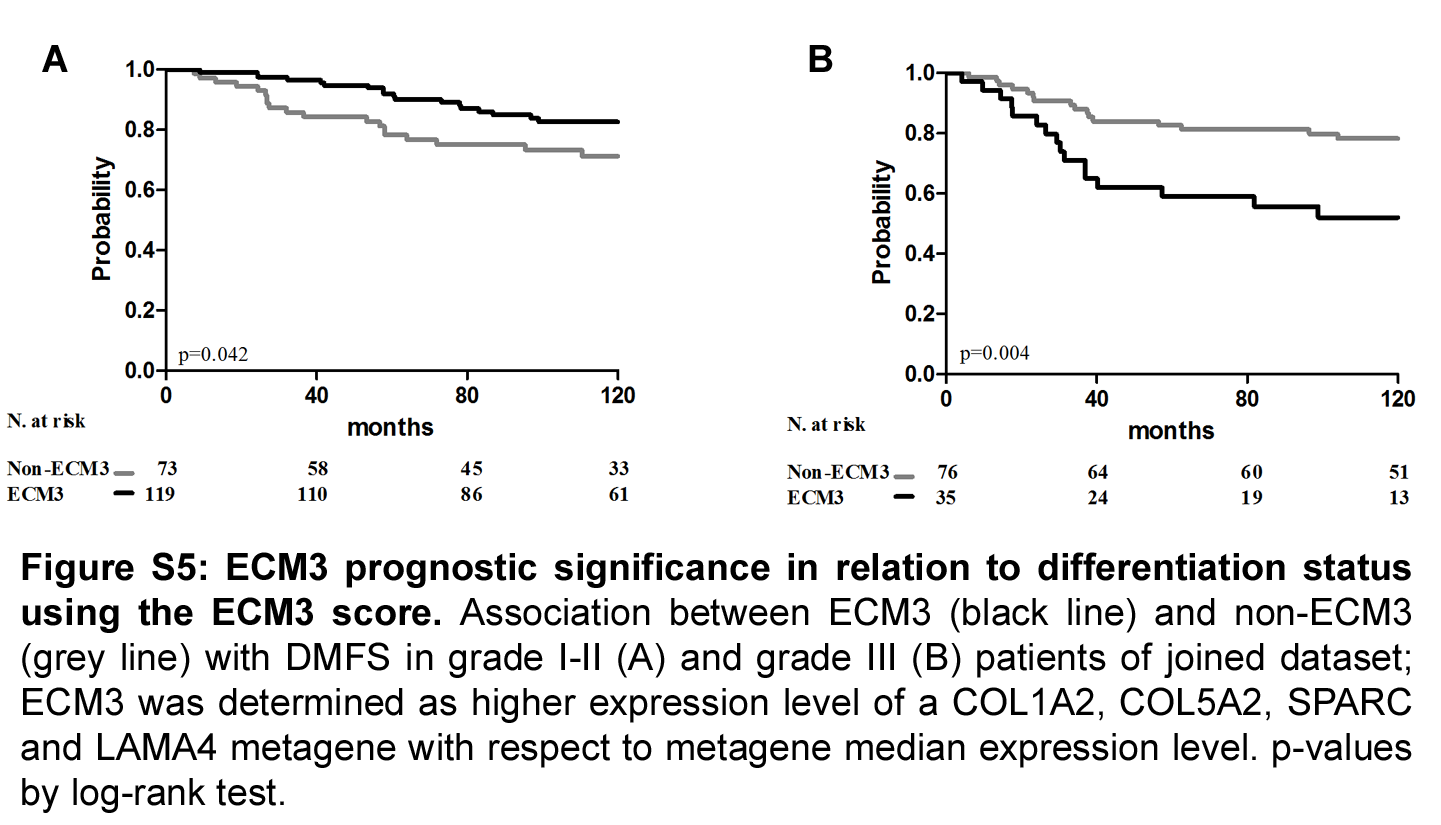

Supplement: Figure S5 — ECM3 prognostic significance in relation to differentiation status using the ECM3 score. Association between ECM3 (black line) and non-ECM3 (grey line) with DMFS in grade I–II (A) and grade III (B) patients of joined dataset; ECM3 was determined as higher expression level of a COL1A2, COL5A2, SPARC and LAMA4 metagene with respect to metagene median expression level. p-values by log-rank test. (TIF) [file pone.0056761.s005.tif]

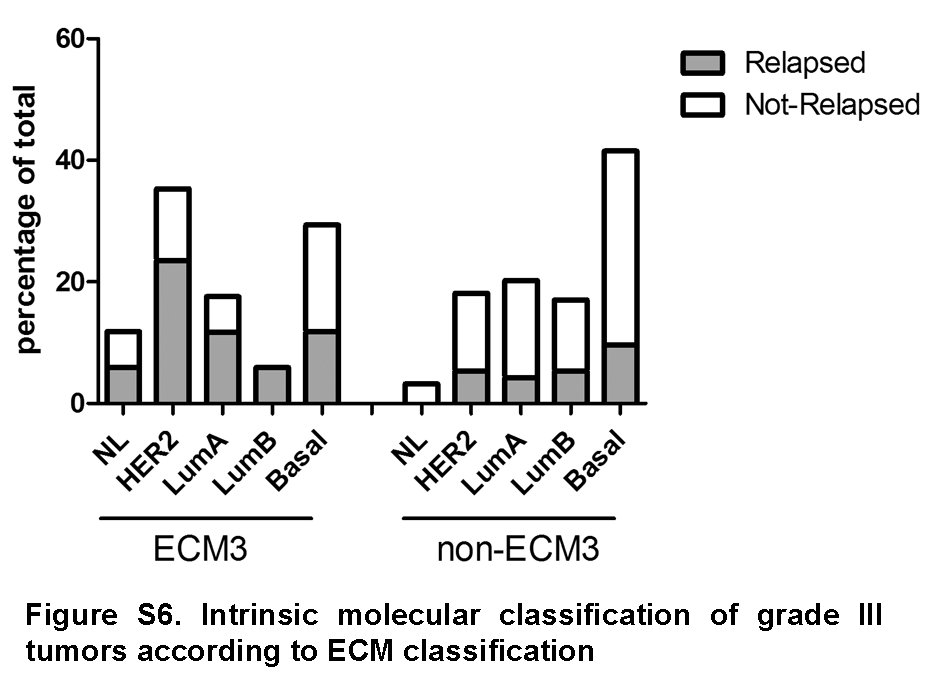

Supplement: Figure S6 — Intrinsic molecular classification of grade III tumors according to ECM classification. (TIF) [file pone.0056761.s006.tif]
