# Supplementary material for: Neoplastic and Stromal Cells Contribute to an Extracellular Matrix Gene Expression Profile Defining a Breast Cancer Subtype Likely to Progress
Source: PLoS One. 2013 Feb 18;8(2):e56761. doi: 10.1371/journal.pone.0056761 (PMC3575489; doi:10.1371/journal.pone.0056761)
Supplement: Table S4 — Multivariate proportional hazards analysis of metastasis-free survival in untreated patients according to tumor grade. (DOC) [file pone.0056761.s010.doc]

**Table S4. Multivariate proportional hazards-analysis of metastasis free survival in not treated patients according to grade status**

|  |  | |  |  | | | |
| --- | --- | --- | --- | --- | --- | --- | --- |
| **Variable** | **Hazard Ratio** | **p value** | | **Hazard Ratio** | **p value** | **Hazard Ratio** | **p value** |
|  | **(95% CI)*** |  |  | **(95% CI)*** |  | **(95% CI)*** |  |
|  | **Total** | | | **Grade I-II** | | **Grade III** | |
| Size | 1.5 (1.1-1.9) | 0.0029 | | 2.01 (1.4-2.9) | 0.0002 | 1.2 (0.8-1.7) | 0.4197 |
| ECM 3 | 0.8 (0.4-1.7) | 0.6080 | | 1.01 (0.5-2.1) | 0.9693 | 3.0 (1.3-7.0) | 0.0098 |
| Age | 1.0 (1.0-1.1) | 0.0768 | | 1.0 (1.0-1.1) | 0.0705 | 1.0 (1.0-1.1) | 0.2098 |
| dataset | 1.4 (0.7-3.0) | 0.3467 | | 1.7 (0.8-3.8) | 0.1608 | 0.6 (0.2-1.8) | 0.3304 |
| Er pos | 0.6 (0.4-1.0) | 0.0515 | | 0.4 (0.2-0.8) | 0.0113 | 0.8 (0.4-1.7) | 0.6289 |
| ECM 3*dataset  Grade III  ECM3*Grade III  Grade III*dataset | 0.6 (0.2-1.7)  0.6 (0.3-1.1)  5.5 (2.0-15.1)  0.7 (0.2-2.0) | 0.3014  0.0792  0.0010  0.4536 | | 0.4 (0.1-1.5) | 0.1768 | 2.1 (0.3-14.6) | 0.4586 |

* CI= confidence interval
