# Supplementary material for: Neoplastic and Stromal Cells Contribute to an Extracellular Matrix Gene Expression Profile Defining a Breast Cancer Subtype Likely to Progress
Source: PLoS One. 2013 Feb 18;8(2):e56761. doi: 10.1371/journal.pone.0056761 (PMC3575489; doi:10.1371/journal.pone.0056761)
Supplement: Table S5 — Multivariate proportional hazards analysis of metastasis-free survival in untreated grade III patients according to ER status. (DOC) [file pone.0056761.s011.doc]

**Table S5. Multivariate proportional hazards-analysis of metastasis free survival in grade III not treated patients according to ER status**

| **Variable** | **Hazard Ratio** | **p value** | **Hazard Ratio** | **p value** |
| --- | --- | --- | --- | --- |
|  | **(95% CI)*** |  | **(95% CI)*** |  |
|  | **ER pos** | | **ER neg** | |
| Size | 1.4 (0.7-2.7) | 0.376 | 1.1 (0.7-1.7) | 0.7320 |
| ECM 3 | 2.5 (0.7-8.5) | 0.141 | 4.1 (1.5-11.0) | 0.0049 |
| Age | 1. (0.9-1.1) | 0.900 | 1. (1.0-1.1) | 0.3328 |

* CI= confidence interval
