# Supplementary material for: Neoplastic and Stromal Cells Contribute to an Extracellular Matrix Gene Expression Profile Defining a Breast Cancer Subtype Likely to Progress
Source: PLoS One. 2013 Feb 18;8(2):e56761. doi: 10.1371/journal.pone.0056761 (PMC3575489; doi:10.1371/journal.pone.0056761)
Supplement: Table S6 — Frequency of clinico-pathological features in patients with grade III breast carcinomas according to ECM3 classification. (DOC) [file pone.0056761.s012.doc]

Table S6. Frequency of clinico-pathological features in patients with grade III breast carcinomas according to ECM3 classification

|  | **no. cases p value***  **ECM3/total (%)** | |
| --- | --- | --- |
| ER+ | 5/35 (14) |  |
| ER- | 9/48 (19) | n.s |
| Size ≤ 2 | 6/33 (18) |  |
| Size > 2 | 8/50 (16) | n.s |
| Age < 50 | 11/64 (17) |  |
| Age ≥ 50  IDC^1^  ILC^2^  Other  LVI^3^ pos  LVI neg  TIL^4^ pos  TIL neg | 3/19 (16)  9/70 (13)  2/7 (29)  1/2 (50)  1/8 (12)  3/27 (11)  2/24 (8)  3/10 (30) | n.s  n.s  n.s  n.s |

*evaluated by Fisher’s exact test, ^1^ Invasive Ductal Carcinoma, ^2^ Invasive Lobular Carcinoma, ^3^ Lymphovascular invasion, ^4^ Tumor infiltrating lymphocytes
