# Supplementary material for: Neoplastic and Stromal Cells Contribute to an Extracellular Matrix Gene Expression Profile Defining a Breast Cancer Subtype Likely to Progress
Source: PLoS One. 2013 Feb 18;8(2):e56761. doi: 10.1371/journal.pone.0056761 (PMC3575489; doi:10.1371/journal.pone.0056761)
Supplement: Supporting Information S1 — ECM3 cluster stability analysis. (PDF) [file pone.0056761.s013.pdf]

Supplementary material
Stability analysis of the ECM3 cluster

Neoplastic and stromal cells contribute to an extracellular matrix gene expression profile defining a breast cancer subtype likely to progress

Triulzi T, Casalini P, Sandri M, Ratti M, Carcangiu ML,
Colombo MP, Balsari A, Ménard S, Orlandi R and Tagliabue E.

1 Statistical methods used for ECM3 stability analysis

1.1 Missing value imputation

Missing expression values were imputed using the nearest-neighbor averaging method described in [Troyanskaya *et al.* (2001)] and implemented in the `knn.impute` command of the `impute` R package [Hastie *et al.* (2011)].
The number of neighbors used in the imputation was `k=10`, the maximum percent missing data allowed in any row was `rowmax=0.50`, the maximum percent missing data allowed in any column was `colmax=0.13` and the largest block of genes imputed using the knn algorithm was `maxp = 1500`.

1.2 Data preprocessing

Microarray data matrices were preprocessed using a method similar to the algorithm described in [Kluger *et al.* (2003)]: (a) transform data using using the tail function $\text{sign}(x)\ln(1+|x|)$, where $|x|$ is the absolute value of x, (b) perform 5 cycles of row-column standardization.

1.3 Clustering algorithms

For the identification of the ECM3 cluster, three biclustering algorithms were used: the independent row-column clustering (IRCC) based on k-means clustering, a modification of the IRCC clustering based on column clustering of a selected submatrix (CCSS) and the Large Average Submatrix (LAS) method proposed in [Shabalin *et al.* (2009)].

LAS is an iterative algorithm based on a simple significance score that trades off between the size of a submatrix and its average value. It identifies groups of genes that show similar activity patterns under a specific subset of the experimental conditions. This algorithm is implemented in the LAS program available at https://genome.unc.edu/las/. We searched bicluster with positive score setting the number of iterations to 10000, the score cut-off to 0 and the maximum number of biclusters to 30. We defined the ECM3 bicluster as the LAS submatrix with the SPARC gene in one (or more) of the rows and with the highest positive score.

The IRCC algorithm applies k-means clustering independently to the rows and the columns of the data matrix X, finds the row clusters rC_i, $i = 1, 2, \ldots, k_r$ and the column clusters cC_j, $j = 1, 2, \ldots, k_c$, then reorders rows and columns so that each cluster forms a contiguous group and finally builds biclusters at the intersection of the row clusters with the column clusters. The ECM3 bicluster is identified as follows: (a) find the row cluster rC_i that contains the SPARC gene; (b) consider the biclusters $B_1, \ldots, B_{k_c}$ defined by the intersection of rC_i with the column clusters $\text{cC}_1, \ldots, \text{cC}_{k_c}$; (c) ECM3 is the bicluster B_j with the highest positive mean expression value.

The CCSS algorithm starts applying k-means clustering to the rows of X (genes), finds the row clusters rC_i, $i = 1, 2, \ldots, k_r$, selects the row cluster rC_i that contains the SPARC gene and finally applies k-means column clustering to the submatrix X_{rC_i} of X whose rows belong to rC_i. In other words, CCSS does not apply k-means clustering independently to the rows and the columns of the data matrix but applies column clustering to a subset of rows that has been selected by a previous row clustering. The column cluster with the highest positive mean expression value identifies the ECM3 bicluster.

The number of row and column clusters in IRCC were set to 4 for all the analyzed datasets, with the only exception of the Ma et al. (2004) dataset (GDS806) where the rows and the columns were partitioned in 5 and 3 groups, respectively. k-means clustering was performed using the `kmeans` R command . The maximum number of iterations allowed was `iter.max=30`, the number of random starts was `nstart=20` and the algorithm of Hartigan and Wong was selected (`algorithm = "Hartigan-Wong"`). Using these settings we were able to find IRCC biclusters that are very similar to LAS biclusters.

In CCSS the number of row clusters was set to 4 (with the exception of Ma et al. (2004) dataset (GDS806) where k=5) and the number of column clusters were selected adaptively using two approaches: the consensus clustering of [Monti *et al.* (2003)] and the prediction strength of [Tibshirani *et al.* (2005)]. The two methods gives similar results for all the analyzed datasets.

Prediction strength was calculated using the `prediction.strength` command of the `fpc` R package [Hennig (2010)], applying the k-means clustering algorithm (`method="kmeans"`), with k ranging from 2 to 7 (`Gmin=2, Gmax=7`) and with a number of times the dataset is divided into two halves equal to `M=200`.

Consensus clustering is a resampling-based approach to cluster stability implemented in the R package `ConsensusClusterPlus` (see [Wilkerson (2011)] and [Wilkerson and Hayes (2010)]). The adopted clustering algorithm was k-means (`clusterAlg="km"`), with `distance = "euclidean"`. The maximum cluster number was set to `maxK = 7` and the number of subsamples to `reps = 500`. The proportion of items to sample was `pItem=0.5` and the proportion of features to sample was `pFeature = 1`. See the next section for a brief description of the method.

1.4 Stability analysis

1.5 Using different clustering methods

The stability of the ECM3 partition was first evaluated with respect to the use of different clustering algorithms. Row and column elements of the ECM3 bicluster obtained from LAS were compared to row and column elements of IRCC and CCSS. We assessed the similarity between these partitions of the same dataset using the Jaccard index defined as:

$$\gamma(C_1, C_2) = \frac{|C_1 \cap C_2|}{|C_1 \cup C_2|}, \tag{1}$$

where C_1 and C_2 are two (row or column) partitions and $|C|$ is the number of elements in the C partition. This index ranges from 0 (no common elements in the C_1 and C_2 partitions) to 1 (the elements in the C_1 and C_2 partitions are exactly the same). For details see [Hennig (2007)]. The Jaccard index was calculated using the `clujaccard` command of the `fpc` R packages.

1.6 Removing genes or samples

The stability of the ECM3 cluster was also evaluated comparing the results from clustering on the full data to clustering based on:

- removing one gene at a time;
- removing one sample at a time;
- repeatedly and randomly removing B sets of k genes, where $k = 2/3 \cdot n_r$, n_r is the number of rows (genes) and $B = 1000$.

At each step, after removing one sample or one gene or a set of genes, the partitioning calculated on the full dataset was compared to the partitioning on the reduced dataset and the Jaccard index was calculated. Finally, the histogram and some descriptive statistics about the distribution of the B Jaccard indexes were estimated.

When removing one gene at a time, four additional stability measures were estimated using the `stability` command of the `clValid` R package: average proportion of non-overlap (APN), average distance (AD), average distance between means (ADM) and figure of merit (FOM). The APN measures the average proportion of observations not placed in the same cluster under both cases (i.e. before

and after gene removal). The APN is in the interval $[0, 1]$. Values close to 0 indicate highly consistent clustering results. The AD measures the average distance between observations placed in the same cluster under both cases and the ADM measures the average distance between cluster centers for observations placed in the same cluster under both cases. These measures have values between zero and ∞. Smaller values are preferred. The FOM measures the average intra-cluster variance of the observations in the deleted columns (averaged over all removed columns), where the clustering is based on the remaining (undeleted) columns. FOM has values between zero and ∞ and should be minimized.

Using the `stability` command of the `clValid` R package with `clMethods = "kmeans"`, `metric = "euclidean"`, `iter.max = 30` and `nstart = 10` we estimated the above stability measures varying the number of cluster from 2 to 6 (`nClust = 2:6`) and removing one column at a time.

1.7 Bootstrapping, jittering, adding noise points

In addition, following [Hennig (2007)], we investigated whether the ECM3 cluster remains stable:

- for different data sets drawn from the same underlying distribution (bootstrapping);
- under the addition of a random error to every point of the data set (jittering);
- under the replacement of some point in the data set by "noise points" or outliers.

Data were bootstrapped, jittered and noise points were added producing modified datasets for B times. At each step, the partitioning calculated on the full dataset was compared to the partitioning on the modified dataset and the Jaccard index was calculated. Finally, the histogram and some descriptive statistics about the distribution of the B Jaccard indexes were estimated.

This analysis was performed on the submatrix of X identified by the ECM3 genes. The `clusterboot` command of the `fpc` R package was used with the following parameters: `B = 1000`, `bootmethod = c("boot", "jitter", "noise")`, `clustermethod = kmeansCBI`, `runs = 10`, `jittertuning = 0.1`, `noisetuning = c(0.1, 4)`. The number of cluster `k` was set equal to the number of clusters of the CCSS algorithm.

The stability of the discovered ECM3 cluster with respect to sampling variability was also assessed using consensus clustering [Monti *et al.* (2003)]. Working in conjunction with resampling techniques for simulating perturbations of the original data, it provides for a method to represent the consensus across multiple runs of a clustering algorithm. Consensus clustering is based on the notions of connectivity matrix and consensus matrix. Let $X^{(1)}, \ldots, X^{(B)}$ be a collection of perturbed data set obtained by resampling the original X matrix. The connectivity matrix $M^{(b)}$ corresponding to the bth dataset is a $N \times N$ matrix whose entries are defined as follows: $M^{(b)}(i, j) = 1$ if items i and j belong to the same cluster and $M^{(b)}(i, j) = 0$ otherwise. The consensus matrix $\mathcal{M}$ is a $N \times N$ matrix is a properly normalized sum of the connectivity matrices of all the perturbed data sets $\{X^{(b)} : b = 1, 2, \ldots, H\}$. Its entries are defined as follows: $\mathcal{M}(i, j) = \sum_b M^{(b)}(i, j) / \sum_b I^{(b)}(i, j)$, where $I^{(b)}(i, j)$ is an $(N \times N)$ indicator matrix such that its (i, j)-th entry is equal to 1 if both items i and j are present in the dataset $X^{(b)}$, and 0 otherwise.

In other words, a consensus matrix is a matrix that stores, for each pair of items, the proportion of clustering runs in which two items are clustered together. The consensus matrix is used as a visualization tool to help assess the clusters' composition and the number of clusters. Associating a color gradient to the 0-1 range of real numbers of $\mathcal{M}$, so that white corresponds to 0, and dark blue corresponds to 1, and arranging $\mathcal{M}$ so that items belonging to the same cluster are adjacent to each other (with the same item order used to index both the rows and the columns of the matrix), a matrix corresponding to perfect consensus will be displayed as a color-coded heat map characterized by blue blocks along the diagonal, on a white background.

Plotting a histogram of a consensus matrix entries (i.e., a histogram of the $N(N-1)/2$ entries $\mathcal{M}(i,j)$'s for $i<j$), perfect consensus would translate into two bins centered at 0 and 1. Plotting the corresponding empirical cumulative distribution (CDF) defined over the range $[0,1]$, perfect consensus would translate into a step function with a step around 0 (the magnitude of which is equivalent to the proportion of 0's in the matrix), a flat line reaching across the 0-1 range, and a second step around 1. For more details, see [Handl *et al.* (2005)].

1.8 Internal validation

The internal validation of the ECM3 cluster was performed evaluating measures that reflect the compactness, connectedness, and separation of the cluster partitions.

The Dunn index and silhouette width are measures that combine compactness and separation. The Dunn index is the ratio of the smallest distance between observations not in the same cluster to the largest intra-cluster distance. It has a value between zero and ∞ and should be maximized. The silhouette value measures the degree of confidence in the assignment of a particular observation to a cluster. If silhouette value is close to 1, it means that sample is 'well-clustered' and it was assigned to a very appropriate cluster. If silhouette value is about zero, it means that that sample could be assign to another closest cluster as well, and the sample lies equally far away from both clusters. If silhouette value is close to -1, it means that sample is 'misclassified' and is merely somewhere in between the clusters. The silhouette width of a cluster is the average of the silhouette values of each observation in the cluster. It lies in the interval $[-1,1]$ and should be maximixed. For more details see [Brock *et al.* (2008)].

Connectedness measures to what extent a partitioning places observations in the same cluster as their nearest neighbors in the data space, and is measured by connectivity. Connectivity has a value between zero and ∞ and should be minimized [Brock *et al.* (2008)].

The Dunn index and connectivity of ECM3 were calculated using the `dunn` and `connectivity` commands of the `clValid` R package [Brock *et al.* (2011)]. The size of the neighborhood was `neighbSize = 10` and the metric used to determine the distance matrix was `method = "euclidean"`. The silhouette widths were calculated and plotted using the `silhouette` command of the `cluster` R package [Maechler *et al.* (2005)].

Using the `stability` command of the `clValid` R package with `clMethods = "kmeans"`, `metric = "euclidean"`, `iter.max = 30` and `nstart = 10` we estimated the above internal measures varying the number of cluster from 2 to 6 (`nClust=2:6`) and removing one column at a time.

1.9 Statistical significance

An important question about clustering is the need to decide if a given cluster is actually present in the data or if it is an artifact of the natural sampling variation. In our work the statistical significance of the ECM3 cluster was assessed using the SigClust method proposed in [Liu *et al.* (2008)] and implemented in the `sigclust` R package [Huang *et al.* (2010)]. This approach is based on the two-means cluster index (CI), a measure of data non-Gaussianity, defined as the ratio between the within-group sum of squared distances to group means and the overall sum of squared distances to the overall mean:

$$\text{CI} = \frac{\sum_{j\in C_1}||\mathbf{x}_j - \bar{\mathbf{x}}^{(1)}||^2 + \sum_{j\in C_2}||\mathbf{x}_j - \bar{\mathbf{x}}^{(2)}||^2}{\sum_{j=1}^{n}||\mathbf{x}_j - \bar{\mathbf{x}}||^2}, \qquad (2)$$

where C_1 and C_2 denote the two clusters, $\bar{\mathbf{x}}^{(1)}$ and $\bar{\mathbf{x}}^{(2)}$ represent the mean of the C_1 and C_2 clusters and $\bar{\mathbf{x}}$ is the overall mean. The smaller the CI, the larger the proportion of the overall variance that is explained by clustering.

Using a large number of simulations, the SigClust method obtain an empirical distribution of the CI based on the null hypothesis of absence of clusters and calculate a p-value for the value of CI calculated on the original data set.

In the present study the number of simulations was `nsim = 1000` and covariance was estimated by the original background noise thresholded estimate ('hard thresholding', `icovest=3`).

1.10 Informative genes

The importance of genes in predicting the ECM3 partitioning was estimated using the method based on random forests [Breiman (2001)] and implemented in the `party` R package [Hothorn *et al.* (2006)].

For each dataset we started fitting a conditional random forests with `ntree = 5000` trees using the `cforest` command. The outcome was the ECM3 sample partitioning found by LAS and the covariates are the genes. The number of randomly preselected variables was set equal to the square root of the number of genes, i.e. `mtry = round(sqrt(nrow(X)))`, as suggested in [Genuer *et al.* (2008)] for high dimensional classification problems. Other settings for the random forests were: `teststat = "quad"`, `testtype = "Bonferroni"`, `mincriterion = 0.90`, `replace = F`, `fraction = 0.632`, `minsplit=5`, `maxsurrogate = 0` and `maxdepth = 0`.

After fitting random forests, the unconditional 'mean decrease in accuracy' importance was estimated using the `varimp` command of `party`. Negative importances were set to zero. A bar plot of the first 50 most informative genes were plotted.

It is interesting to compare this list of informative genes with the consensus list that we found using the COSA algorithm of [Friedman and Meulman (2004)]. A good agreement between the two lists suggests a good stability of the proposed ECM3 consensus list.

References

[Breiman (2001)] Breiman (2001), Random Forests, *Machine Learning*, 45(1), 5–32

[Brock *et al.* (2008)] Brock G., Pihur V., Datta S. and Datta S. (2005), clValid , an R package for cluster validation, *Journal of Statistical Software*, 25(4). URL http://www.jstatsoft.org/v25/i04

[Brock *et al.* (2011)] Brock G., Pihur V., Datta S. and Datta S. (2005), clValid: Validation of Clustering Results, *R package version 0.6-2*, 25(4). URL http://CRAN.R-project.org/package=clValid

[Friedman and Meulman (2004)] Friedman J. and Meulman J.J. (2004), Clustering objects on subsets of attributes, *Journal of the Royal Statistical Society B*, 66(4), 815–849

[Genuer *et al.* (2008)] Genuer R., Poggi J-M. and Tuleau C. (2005), Random Forests: some methodological insights, arXiv:0811.3619v1

[Handl *et al.* (2005)] Handl J., Knowles J. and Kell D.B (2005), Computational cluster validation in post-genomic data analysis, *Bioinformatics*, 21(15), 3201–3212

[Hastie *et al.* (2011)] Hastie T., Tibshirani R., Narasimhan B. and Chu G. (2011), impute: Imputation for microarray data, *R package version 1.26.0*, URL http://CRAN.R-project.org/package=impute

[Hennig (2007)] Henning C. (2007), Cluster-wise assessment of cluster stability, *Computational Statistics & Data Analysis*, 52, 258–271

[Hennig (2010)] Hennig C. (2010), fpc: Flexible procedures for clustering, *R package version 2.0-3*, URL http://CRAN.R-project.org/package=fpc

[Hothorn *et al.* (2006)] Hothorn T., Buehlmann P., Dudoit S., Molinaro A. and Van Der Laan M. (2006), Survival Ensembles, *Biostatistics*, 7(3), 355–373

[Huang *et al.* (2010)] Huang H., Liu Y. and Marron J.S. (2010), sigclust: Statistical Significance of Clustering, *R package version 1.0.0*, URL http://CRAN.R-project.org/package=sigclust

[Kluger *et al.* (2003)] Kluger Y., Basri R., Chang J.T., and Gerstein M. (2003), Spectral Biclustering of Microarray Data: Coclustering Genes and Conditions, *Genome Research*, 13, 703–716

[Liu *et al.* (2008)] Liu Y, Hayes D.N., Nobel A. and Marron J.S. (2008), Statistical Significance of Clustering for High-Dimension, Low-Sample Size Data, *Journal of the American Statistical Association*, 103(483), 1281–1293

[Madeira and Oliveria (2004)] Madeira S.C.and Oliveria A.L. (2004), Biclustering Algorithms for Biological Data Analysis: A Survey, *IEEE Transactions on Computational Biology and Bioinformatics*, 1(1), 24–45

[Maechler *et al.* (2005)] Maechler M., Rousseeuw P., Struyf A. and Hubert M. (2005). Cluster Analysis Basics and Extensions, *Unpublished*

[Monti *et al.* (2003)] Monti S., Tamayo P., Jill M. and Golub T. (2003), Consensus Clustering: A Resampling-Based Method for Class Discovery and Visualization of Gene Expression Microarray Data, *Machine Learning*, 52(1-2), 91–118

[Shabalin *et al.* (2009)] Shabalin A.A., Weigman V.J., Perou C.M. and Nobel A.B. (2009), Finding Large Average Submatrices in high dimensional data, *The Annals of Applied Statistics*, 3(3), 985-1012

[Tibshirani *et al.* (2005)] Tibshirani, R. and Walther, G. (2005), Cluster Validation by Prediction Strength, *Journal of Computational and Graphical Statistics*, 14, 511–528

[Troyanskaya *et al.* (2001)] Troyanskaya O., Cantor M., Sherlock G., Brown P., Hastie T., Tibshirani R., Botstein D. and Altman R.B. (2001), Missing value estimation methods for DNA microarrays, *Bioinformatics*, 17(6), 520–525

[Wilkerson (2011)] Wilkerson M.D. (2011), ConsensusClusterPlus, *R package version 1.5.1*, URL http://www.bioconductor.org/packages/ 2.6/bioc/html/ConsensusClusterPlus.html

[Wilkerson and Hayes (2010)] Wilkerson M.D. and Hayes D.N. (2003), ConsensusClusterPlus: a class discovery tool with confidence assessments and item tracking, *Bioinformatics*, 26(12), 1572–1573

2 Van'T Veer et al (2002) dataset

2.1 LAS bicluster

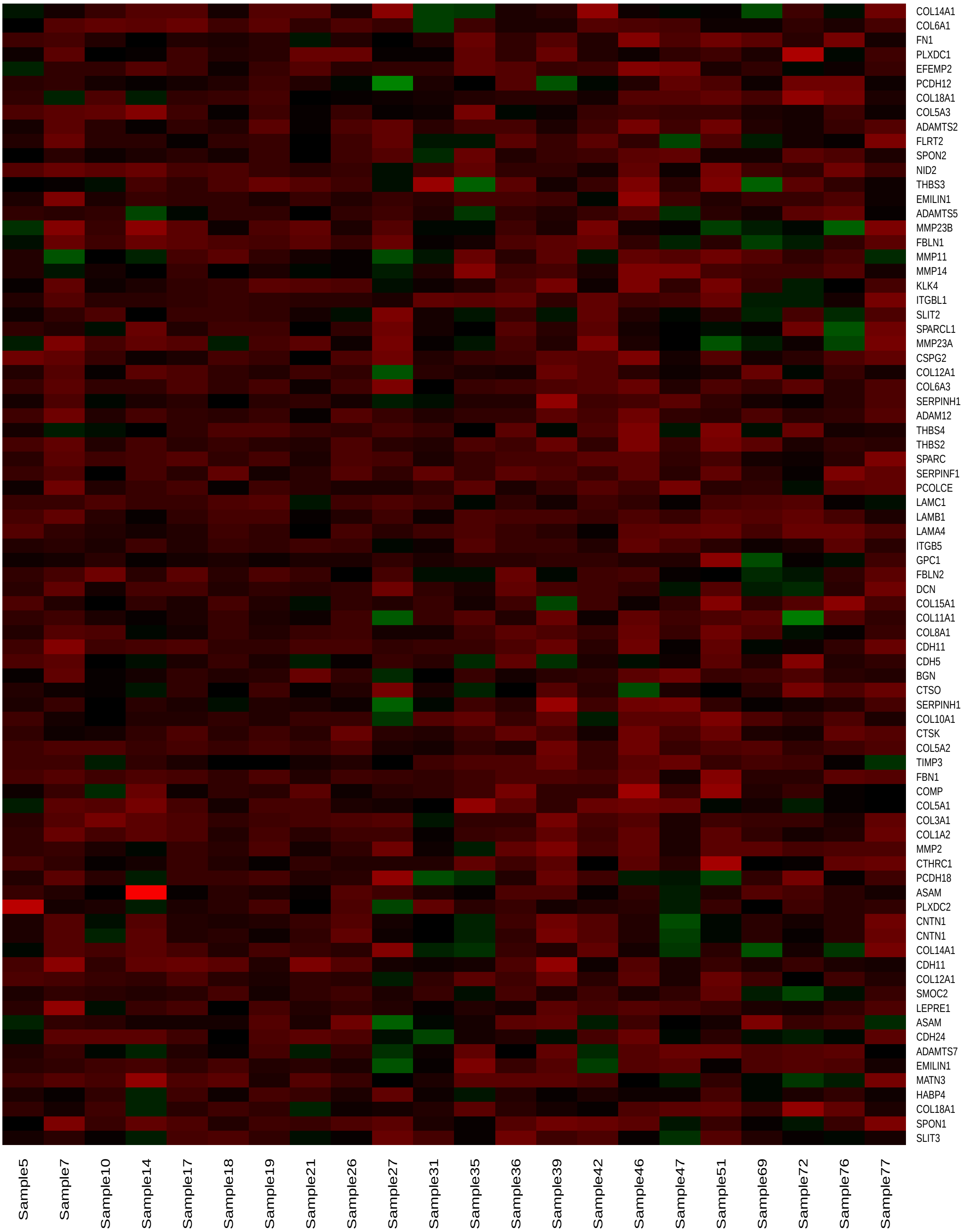

Figure 1: Heatmap of the LAS bicluster

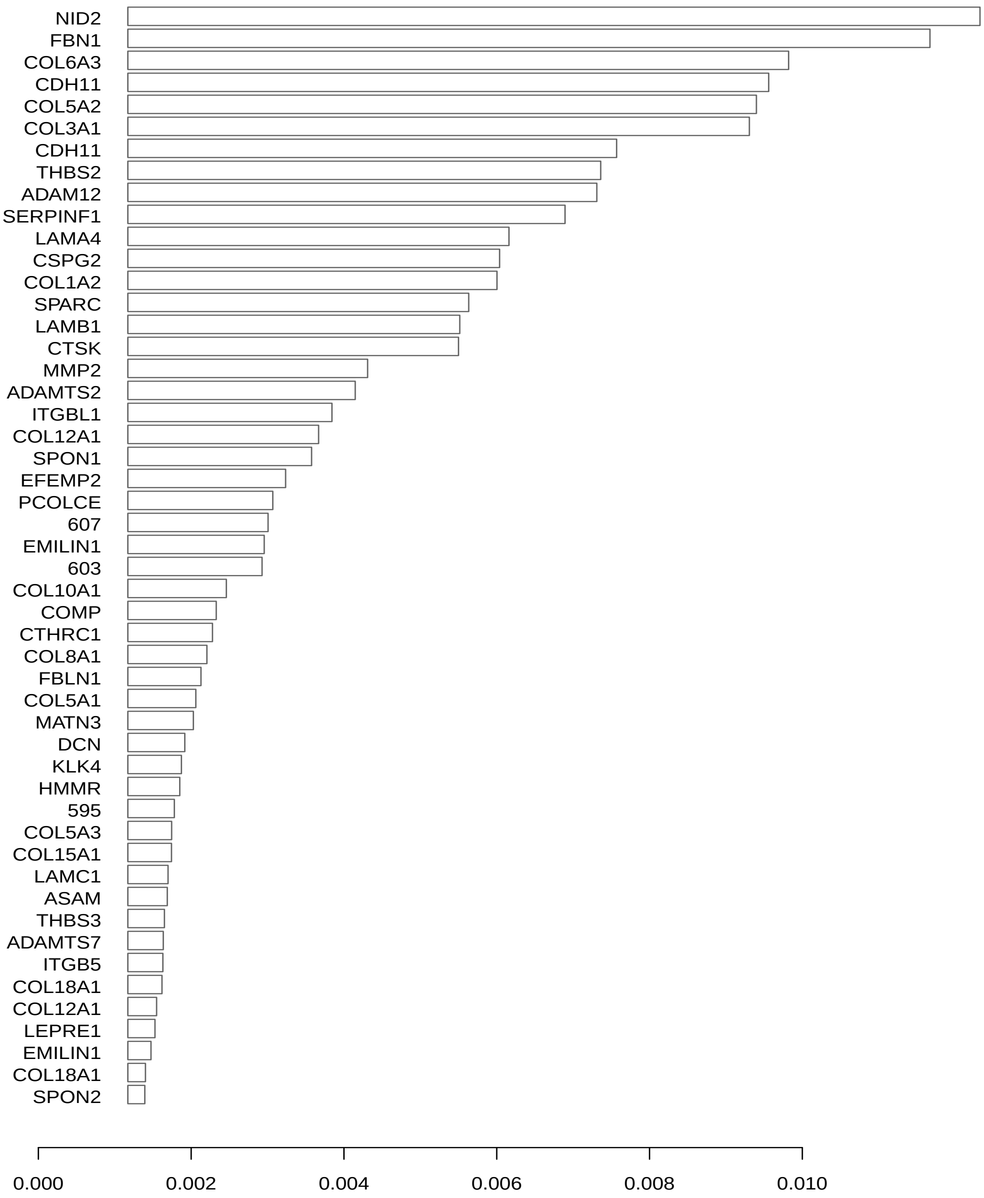

Figure 2: Most informative genes for the LAS bicluster

```
Number of informative genes of the LAS bicluster that also belong
to the ECM3 consensus list: 28 of 34 (82%)
```

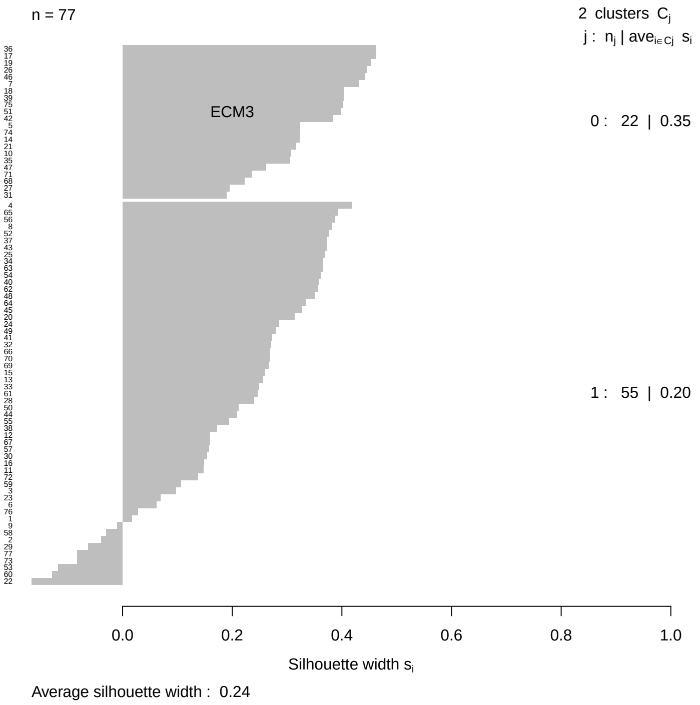

Figure 3: Silhoutte plot for the LAS bicluster

Connectivity	Dunn Index
15.99	0.32

Table 1: Connectivity validation measure and Dunn Index of LAS partitioning

2.2 IRCC-KM bicluster

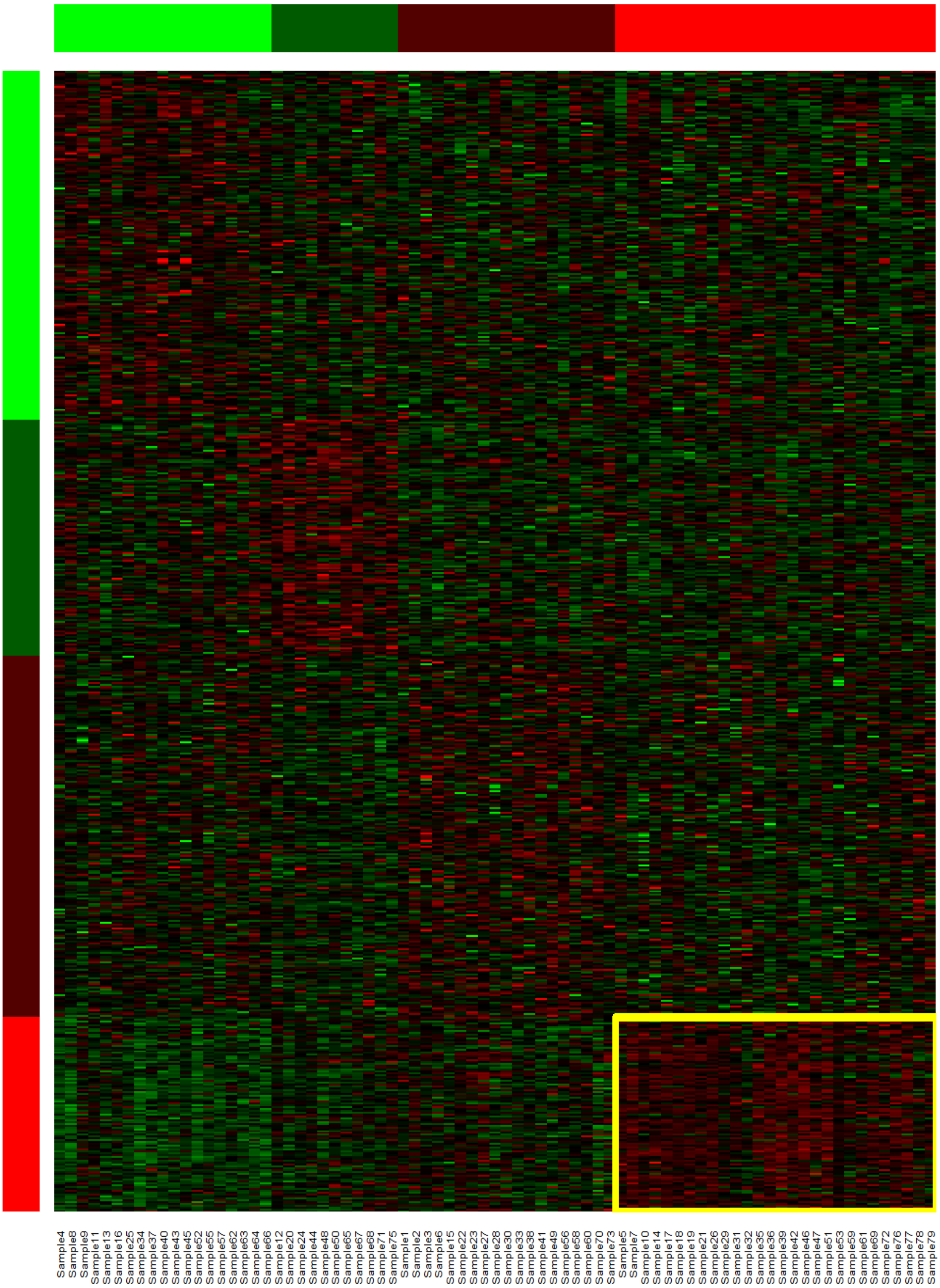

Figure 4: Heatmap of the IRCC-KM bicluster

IRCC-KM genes

ADAM12	ADAMTS10	ADAMTS2	ADAMTS5	ADAMTS7	APP	ASAM	ASAM
BGN	CDH11	CDH11	CDH13	CDH24	CDH5	CEECAM1	CHPF
CNTN1	CNTN1	COL10A1	COL11A1	COL12A1	COL12A1	COL14A1	COL14A1
COL15A1	COL18A1	COL18A1	COL1A2	COL3A1	COL5A1	COL5A2	COL5A3
COL6A1	COL6A3	COL8A1	COMP	CSGlcA_T	CSPG2	CTHRC1	CTSK
DCN	EFEMP2	EMILIN1	EMILIN1	ESAM	FBLN1	FBLN1	FBLN2
FBLN5	FBN1	FLRT2	FN1	GPC1	GPC6	HABP4	ITGA11
ITGA5	ITGB1	ITGB1	ITGB5	ITGBL1	KLK4	LAMA4	LAMB1
LAMB2	LAMC1	LEPRE1	MATN3	MMP11	MMP13	MMP14	MMP2
MMP23A	MMP23B	NID2	NRP2	NTN4	PCDH12	PCDH18	PCDH18
PCDH7	PCOLCE	PGCP	PLXDC1	PLXDC2	PLXND1	SDC2	SEMA5A
SERPINF1	SERPINH1	SERPINH1	SLIT2	SLIT3	SMOC2	SPARC	SPARCL1
SPG20	SPG3A	SPON1	SPON2	THBS1	THBS2	THBS3	THBS4
TIMP3							

IRCC-KM samples

Sample5	Sample7	Sample10	Sample14	Sample17	Sample18	Sample19	Sample21
Sample26	Sample29	Sample31	Sample32	Sample35	Sample36	Sample39	Sample42
Sample46	Sample47	Sample51	Sample53	Sample59	Sample61	Sample69	Sample72
Sample76	Sample77	Sample78	Sample79				

2.2.1 Comparing LAS and IRCC-KM biclusters

	No ECM	ECM3
No ECM3	508	1
ECM3	27	78
Jaccard similarity	0.74	

Table 2: Comparing gene lists: LAS (column) vs. IRCC-KM bicluster (row)

	No ECM	ECM3
No ECM3	48	1
ECM3	7	21
Jaccard similarity	0.72	

Table 3: Comparing sample lists: LAS (column) vs. IRCC-KM bicluster (row)

2.3 IRCC-HC bicluster

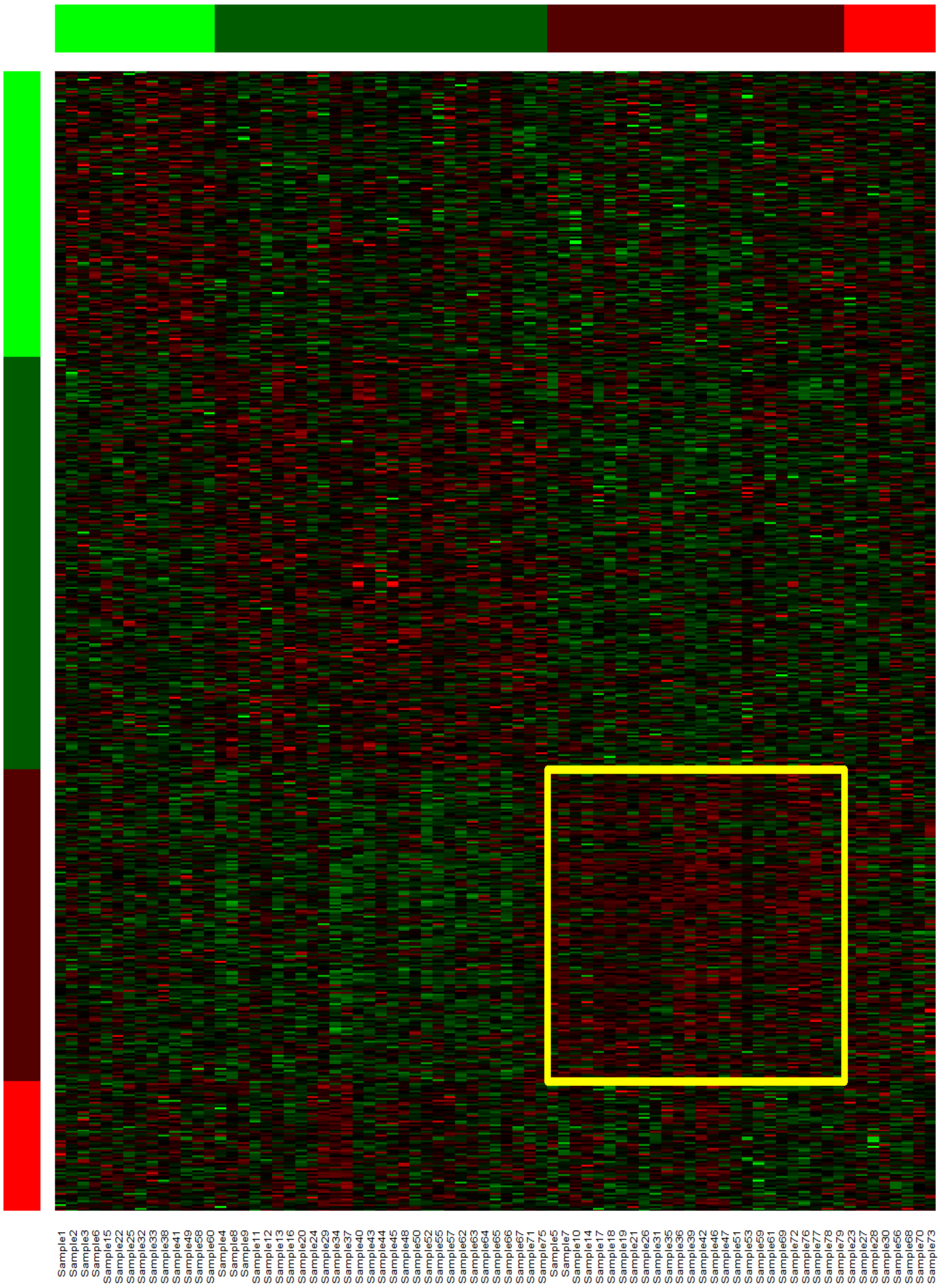

Figure 5: Heatmap of the IRCC-HC bicluster

IRCC-HC genes

ADAM10	ADAM12	ADAM19	ADAM22	ADAM9	ADAMTS1
ADAMTS10	ADAMTS2	ADAMTS5	ADAMTS7	APP	ASAM
ASAM	BGN	BST1	CD36	CD47	CDH11
CDH11	CDH13	CDH15	CDH23	CDH24	CDH5
CHL1	CHPF	CHSY1	CNTN1	CNTN1	CNTN1
CNTN3	COL10A1	COL11A1	COL12A1	COL12A1	COL14A1
COL14A1	COL15A1	COL17A1	COL18A1	COL18A1	COL1A2
COL3A1	COL4A1	COL4A2	COL5A2	COL5A3	COL6A1
COL6A3	COL8A1	COMP	CSGlcA_T	CSPG2	CSPG6
CTHRC1	CTSK	CTSO	DCN	DKFZP586H212	DPP4
EFEMP2	EMILIN1	EMILIN1	ENPEP	ESAM	FBLN1
FBLN1	FBLN1	FBLN2	FBLN5	FBN1	FBN2
FLJ25084	FLRT2	FN1	GALNACT-2	GPC1	GPC6
HABP4	HAS1	HAS2	IBSP	ITGA5	ITGA8
ITGAV	ITGB1	ITGB1	ITGB1BP1	ITGB5	ITGBL1
KLK11	KLK12	KLK4	LAMA4	LAMB1	LAMC1
LEPRE1	MASP1	MATN2	MATN3	MCAM	MGP
MME	MME	MME	MMP11	MMP13	MMP14
MMP2	MMP23A	MMP23B	MMP3	NID2	NTN4
NTN4	PAPLN	PCDH12	PCDH17	PCDH18	PCDH18
PCDH21	PCDH7	PCDHB6	PCOLCE	PECAM1	PGCP
PGCP	PLXDC1	PLXDC2	PLXNA2	PLXNC1	PRG1
SDC2	SELE	SEMA3E	SEMA5A	SEMA6D	SERPINE1
SERPINE1	SERPINF1	SERPINF2	SERPING1	SERPINH1	SERPINH1
SGCA	SGCB	SGCE	SLIT2	SLIT3	SLIT3
SLITRK3	SMOC2	SPARC	SPARCL1	SPG20	SPG3A
SPON1	SPON2	STIM2	THBS1	THBS2	THBS3
THBS4	TIMP1	TIMP3	TIMP4	TNC	VWF

IRCC-HC samples

Sample5	Sample7	Sample10	Sample14	Sample17	Sample18	Sample19	Sample21
Sample26	Sample31	Sample35	Sample36	Sample39	Sample42	Sample46	Sample47
Sample51	Sample53	Sample59	Sample61	Sample69	Sample72	Sample76	Sample77
Sample78	Sample79						

2.3.1 Comparing LAS and IRCC-HC biclusters

	No ECM	ECM3
No ECM3	445	1
ECM3	90	78
Jaccard similarity	0.46	

Table 4: Comparing gene lists: LAS (column) vs. IRCC-HC bicluster (row)

	No ECM	ECM3
No ECM3	50	1
ECM3	5	21
Jaccard similarity	0.78	

Table 5: Comparing sample lists: LAS (column) vs. IRCC-HC bicluster (row)

2.4 CCSS bicluster

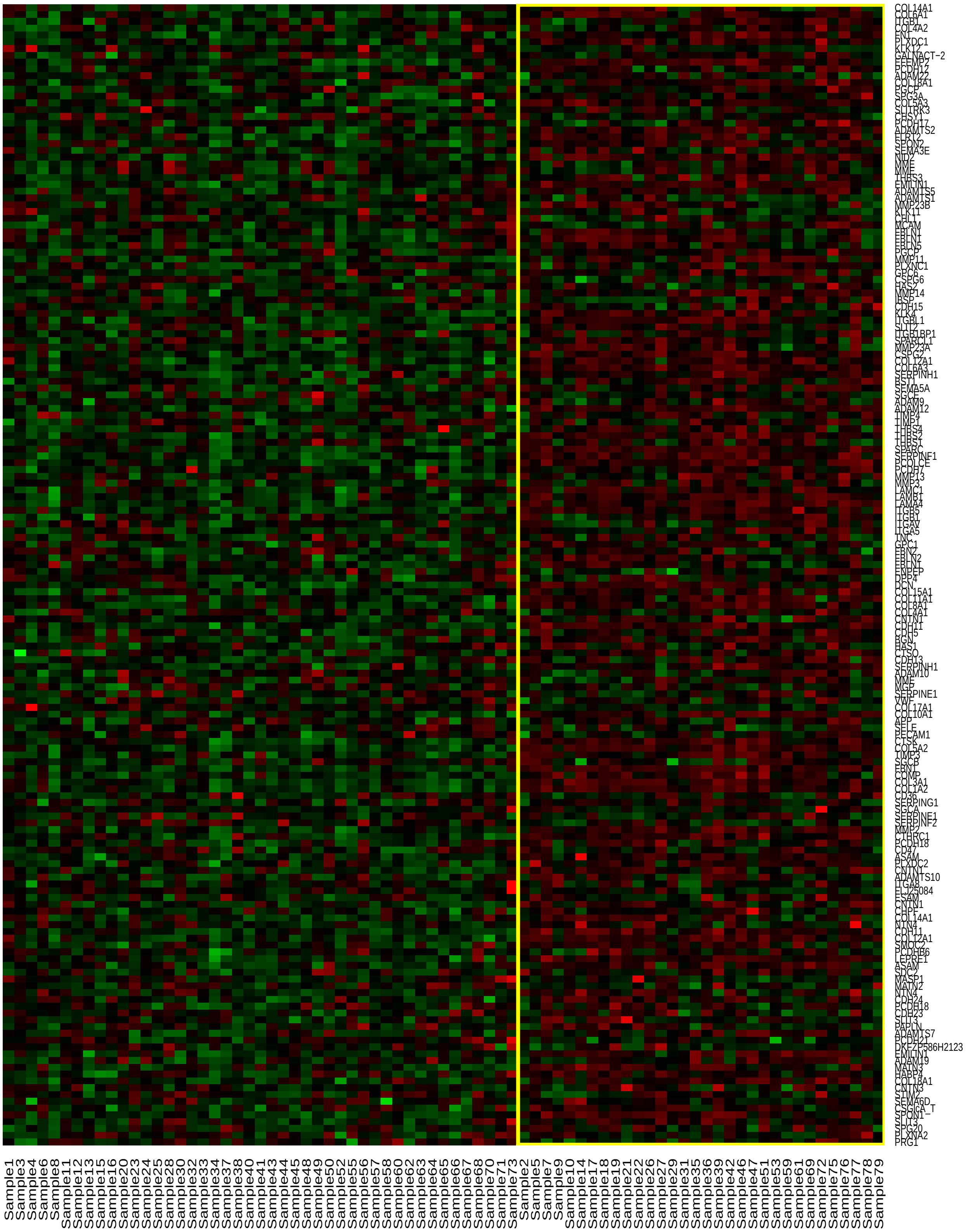

Figure 6: Heatmap of the CCSS bicluster

2.4.1 Comparing LAS and CCSS biclusters

	No ECM	ECM3
No ECM3	45	0
ECM3	10	22
Jaccard similarity	0.69	

Table 6: Comparing sample lists: LAS (column) vs. CCSS (row) biclusters

2.4.2 Prediction strength for CCSS

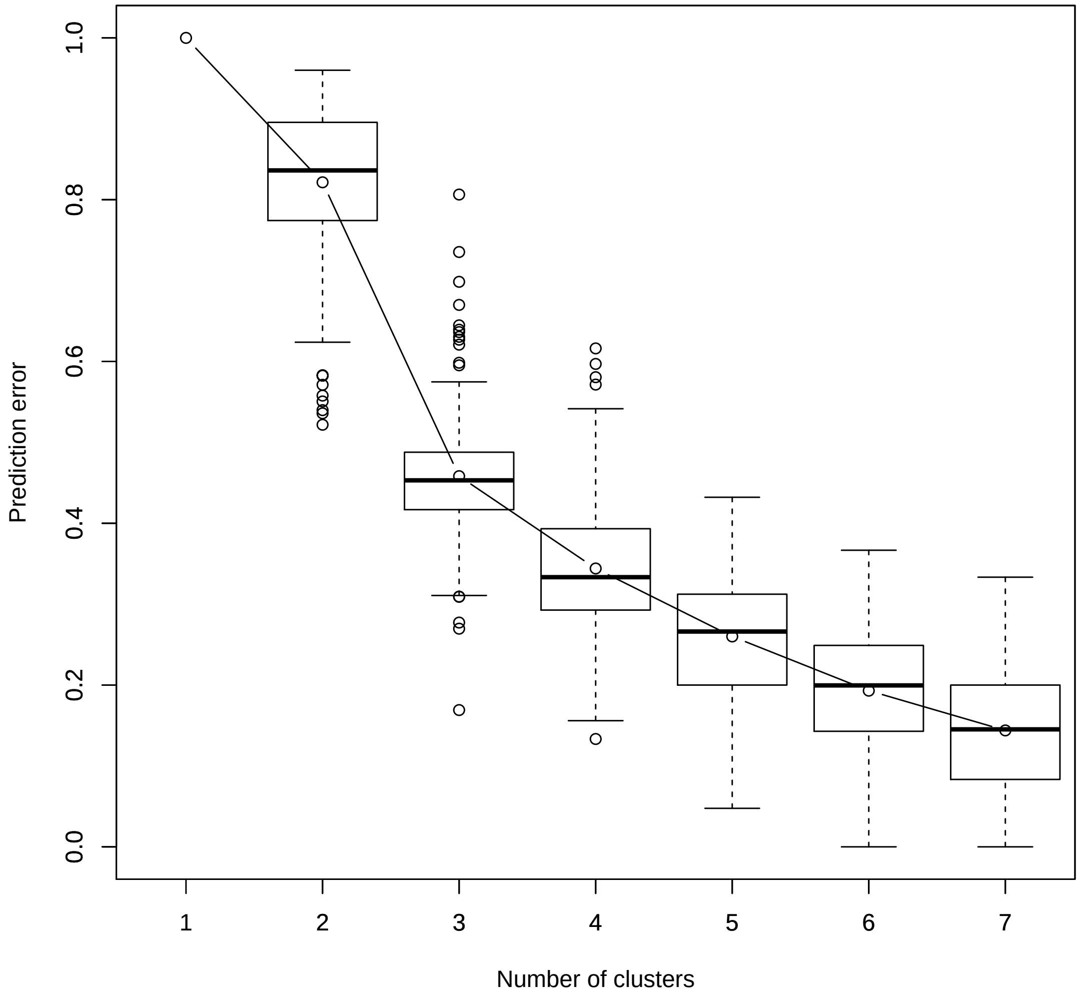

2.4.3 Consensus clustering

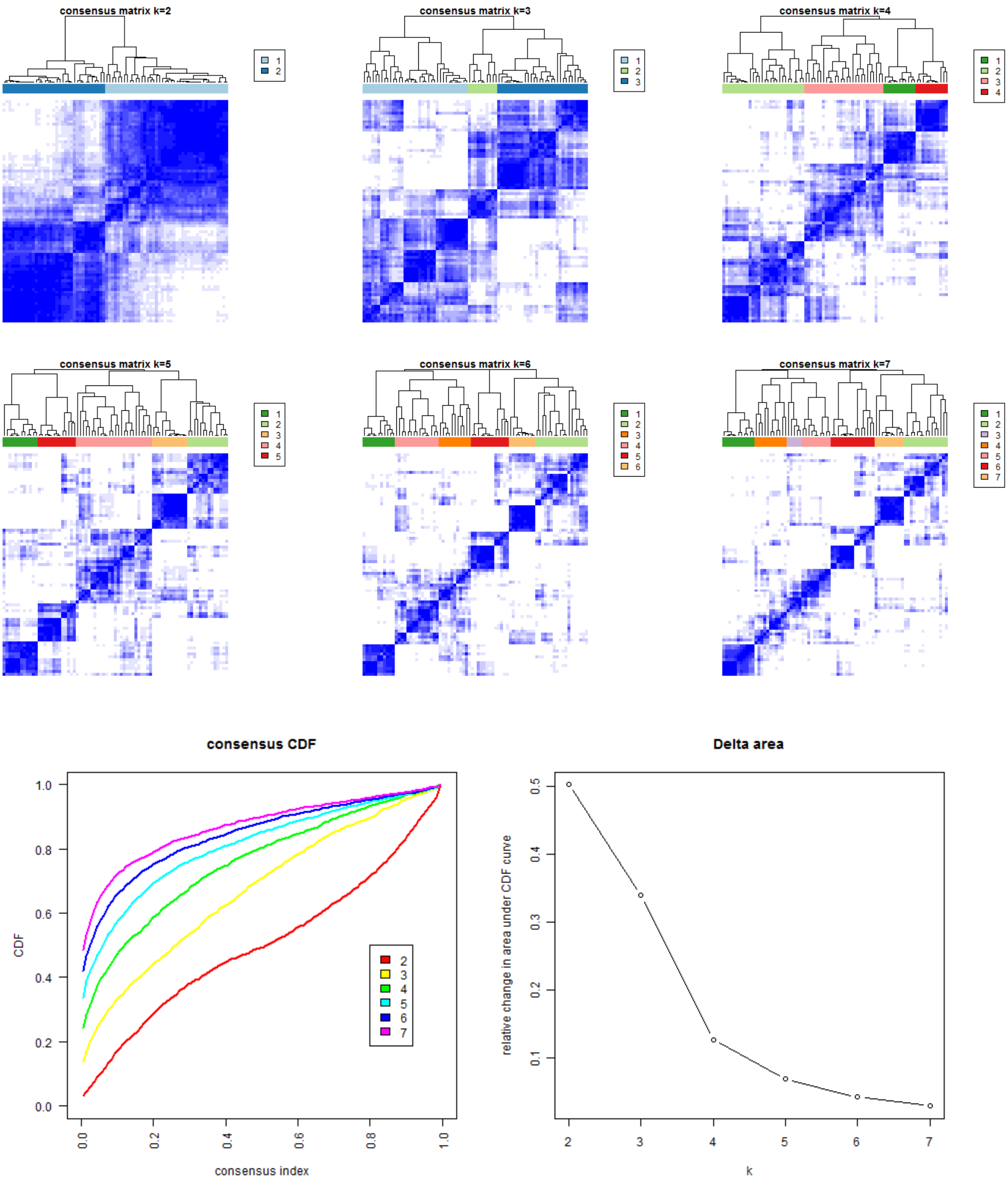

Figure 7: Statistical significance of CCSS clustering (Consensus clustering)

2.4.4 Statistical significance

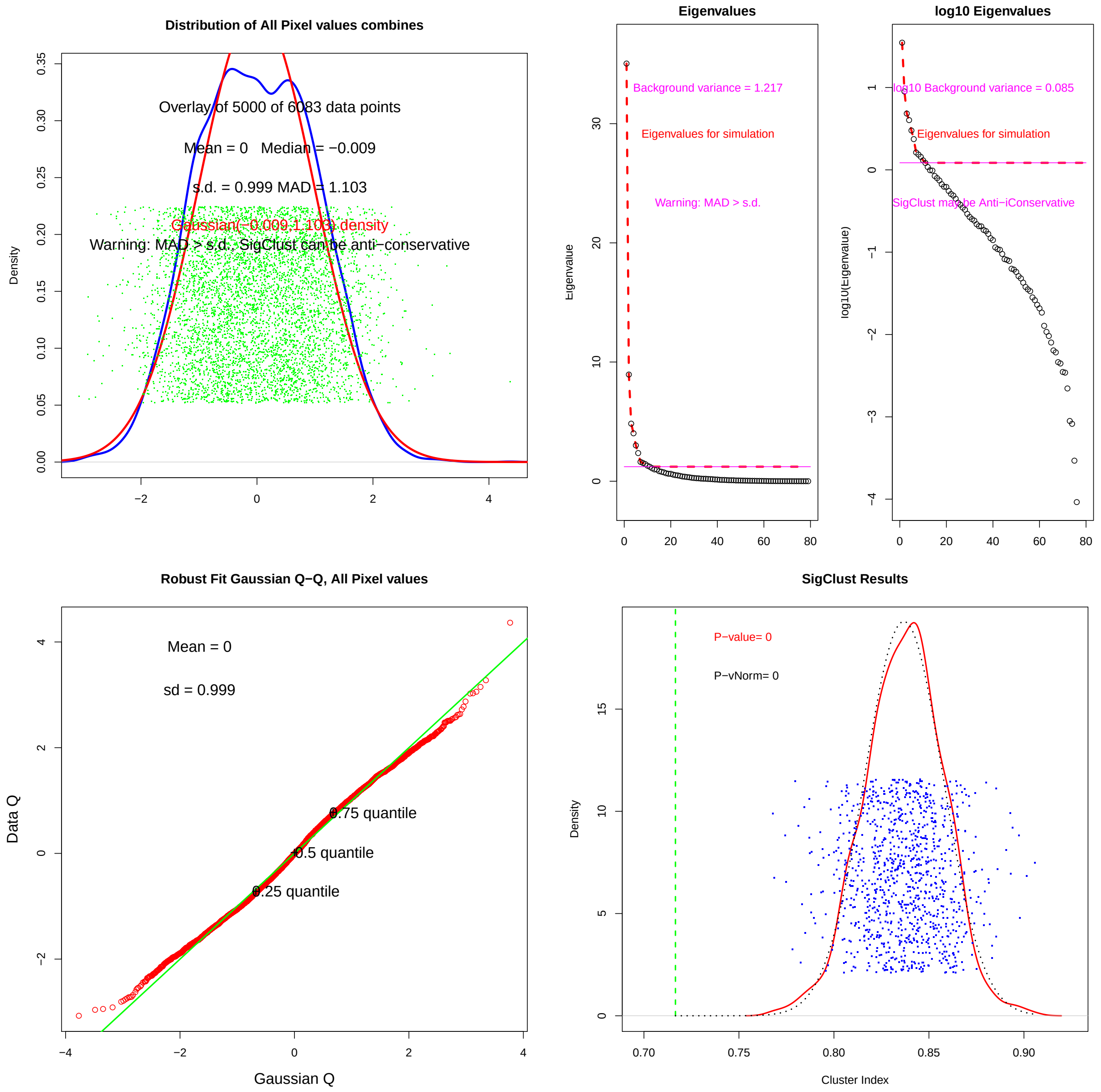

P-value	P-vNorm
0.0E+00	3.1E-09

Table 7: SigClust p-values

2.4.5 Cluster-wise assessment of cluster stability

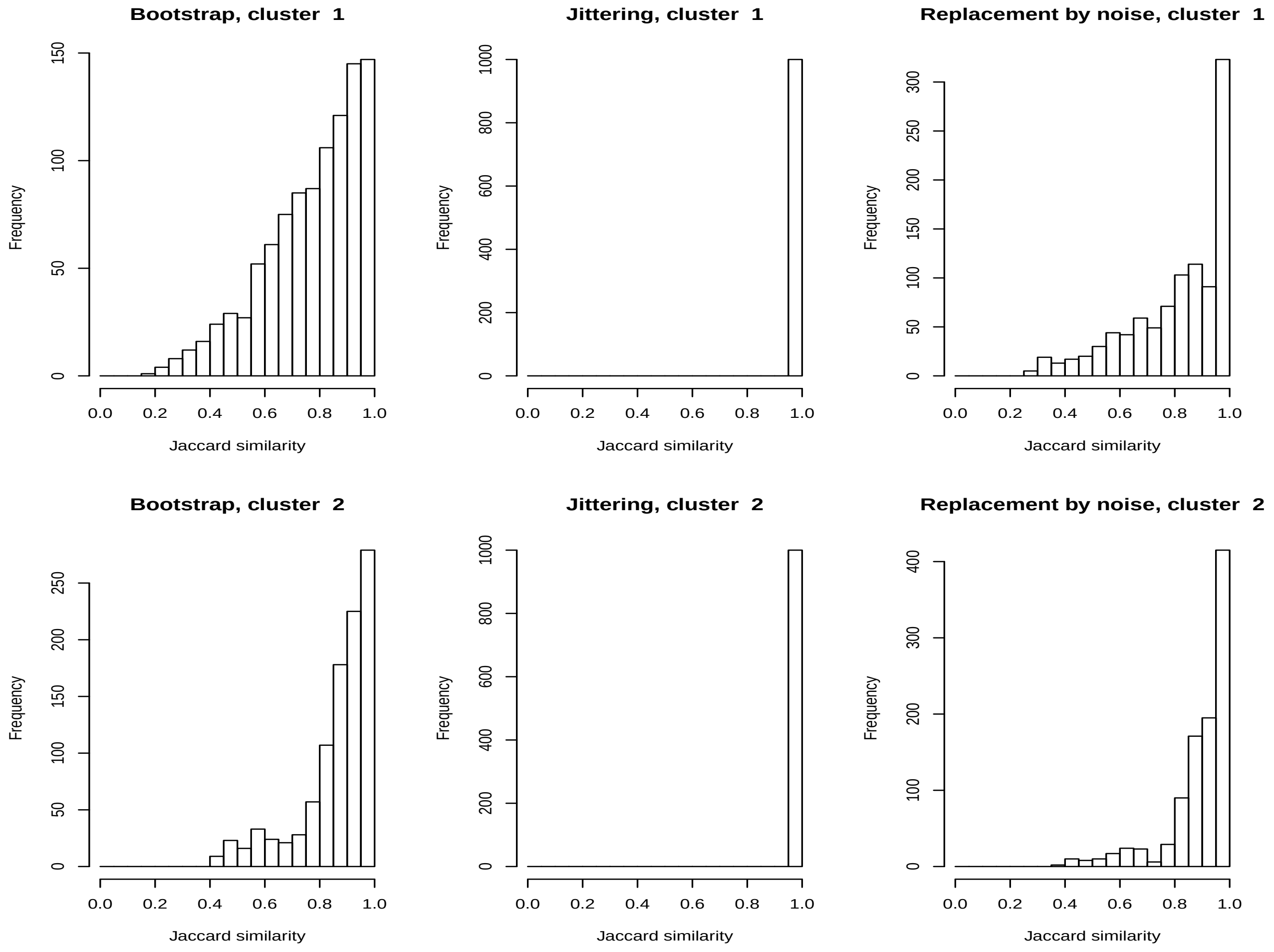


```
* Cluster stability assessment *
Cluster method:  kmeans
Full clustering results are given as parameter result
of the clusterboot object, which also provides further statistics
of the resampling results.
Number of resampling runs:  1000

Number of clusters found in data:  2

 Clusterwise Jaccard bootstrap mean:
[1] 0.7787438 0.8649353
dissolved:
[1] 94 32
recovered:
[1] 606 846
 Clusterwise Jaccard jittering mean:
[1] 1 1
dissolved:
[1] 0 0
recovered:
```

```
[1] 1000 1000
 Clusterwise Jaccard replacement by noise mean:
[1] 0.8150483 0.8962224
dissolved:
[1] 74 20
recovered:
[1] 702 900
```

Removing one sample

```
   Min. 1st Qu.  Median    Mean 3rd Qu.    Max.
 0.9697  1.0000  1.0000  0.9953  1.0000  1.0000
```

Removing one gene

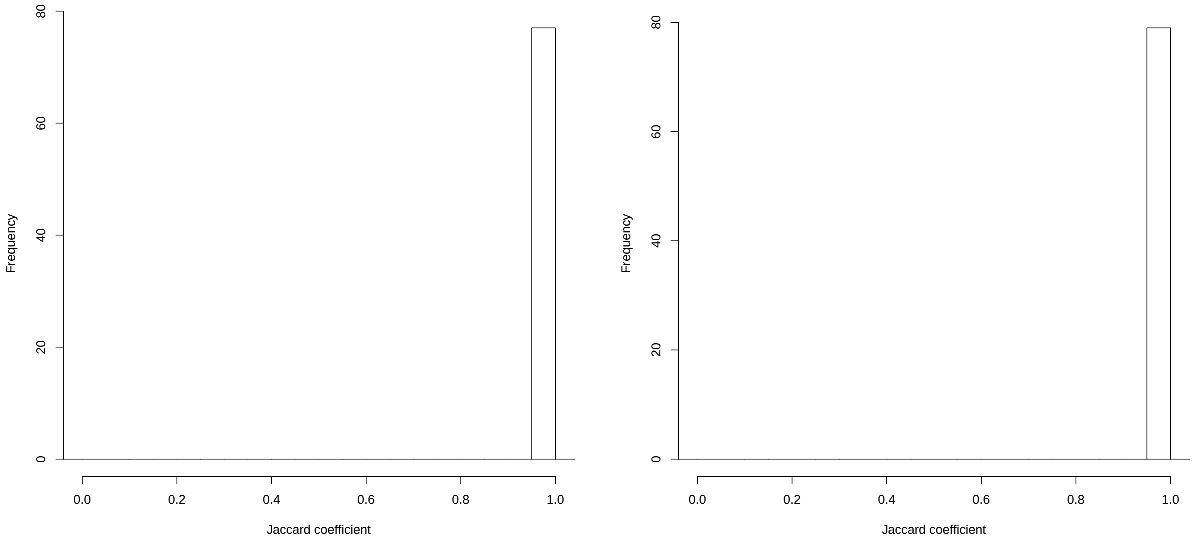

Figure 8: Removing one sample (left) and one gene (right) at a time: distributions of Jaccard coefficients

```
   Min. 1st Qu.  Median    Mean 3rd Qu.    Max.
 0.9697  0.9706  1.0000  0.9907  1.0000  1.0000
```

```
      APN                AD               ADM               FOM
 Min.   :0.2125   Min.   :10.68   Min.   :2.375   Min.   :0.6294
 1st Qu.:0.2286   1st Qu.:10.72   1st Qu.:2.556   1st Qu.:0.7733
 Median :0.2286   Median :10.72   Median :2.556   Median :0.8604
 Mean   :0.2330   Mean   :10.73   Mean   :2.611   Mean   :0.8427
 3rd Qu.:0.2437   3rd Qu.:10.76   3rd Qu.:2.742   3rd Qu.:0.9247
 Max.   :0.2437   Max.   :10.76   Max.   :2.742   Max.   :0.9812
```

Removing sets of k genes

```
   Min. 1st Qu.  Median    Mean 3rd Qu.    Max.
 0.7045  0.8611  0.8889  0.8889  0.9167  1.0000
```

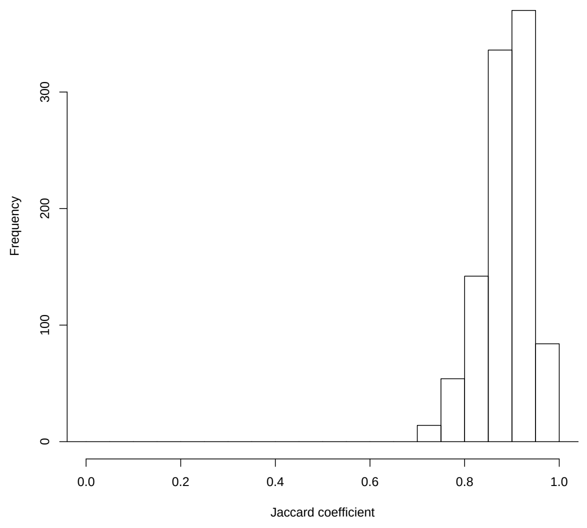

Figure 9: Removing two thirds of the genes: distribution of Jaccard coefficients

2.4.6 Validation measures

```
Clustering Methods:
 kmeans

Cluster sizes:
 2 3 4 5 6

Validation Measures:
                           2       3       4       5       6

kmeans APN            0.0080  0.1198  0.0320  0.1266  0.1599
       AD            10.1230  9.7911  9.1010  8.9703  8.6735
       ADM            0.1152  1.3278  0.3305  1.1478  1.3488
       FOM            0.8427  0.8114  0.7739  0.7688  0.7420
       Connectivity  23.9448 41.5837 44.7028 66.4540 70.1206
       Dunn           0.3521  0.3714  0.4027  0.4198  0.4184
       Silhouette     0.2444  0.1501  0.1594  0.1158  0.1209

Optimal Scores:

             Score   Method Clusters
APN           0.0080 kmeans 2
AD            8.6735 kmeans 6
ADM           0.1152 kmeans 2
FOM           0.7420 kmeans 6
Connectivity 23.9448 kmeans 2
Dunn          0.4198 kmeans 5
Silhouette    0.2444 kmeans 2
```

3 Chin et al. (2006) dataset

3.1 LAS bicluster

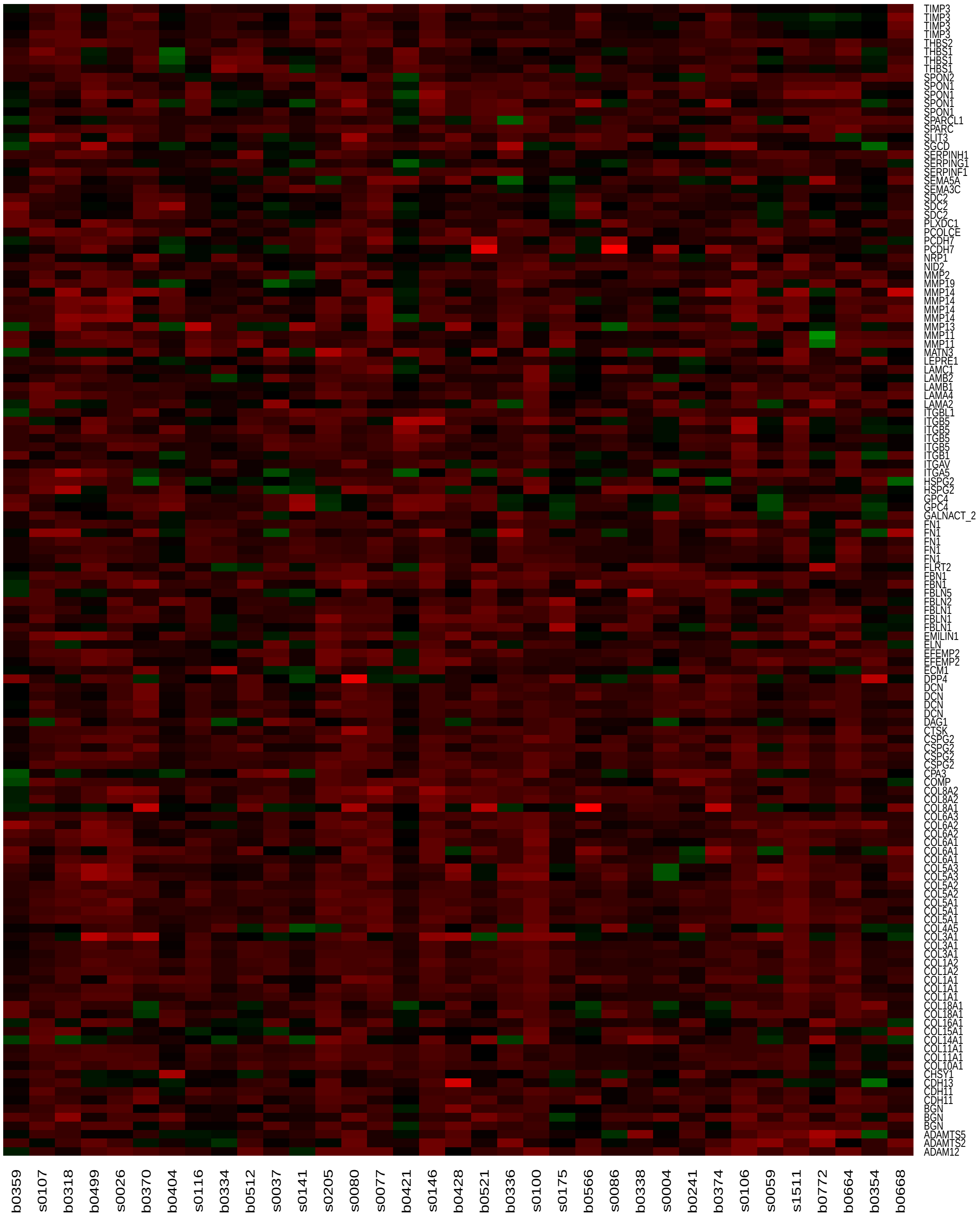

Figure 10: Heatmap of the LAS bicluster

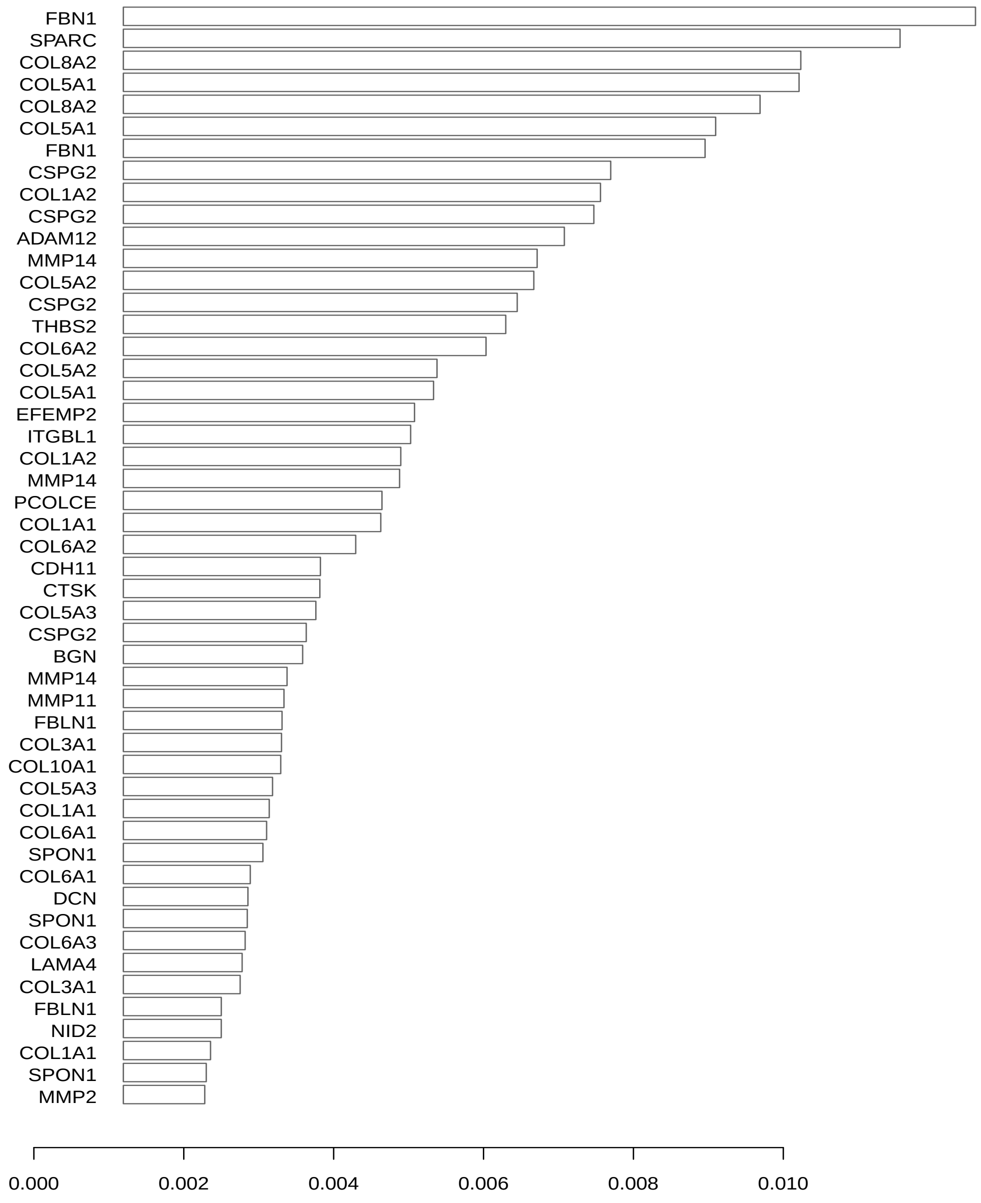

Figure 11: Most informative genes for the LAS bicluster

```
Number of informative genes of the LAS bicluster that also belong
to the ECM3 consensus list: 32 of 34 (94%)
```

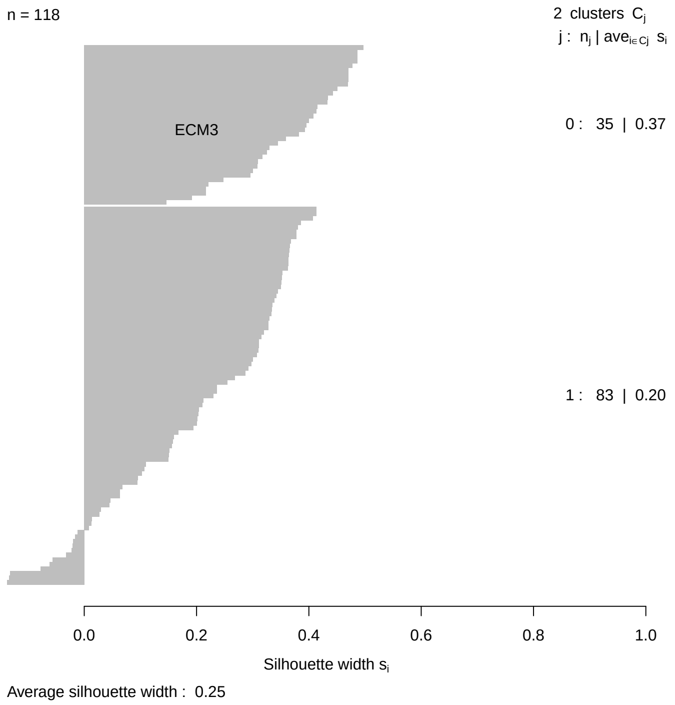

Figure 12: Silhoutte plot for the LAS bicluster

Connectivity	Dunn Index
17.90	0.38

Table 8: Connectivity validation measure and Dunn Index of LAS partitioning

3.2 IRCC-KM bicluster

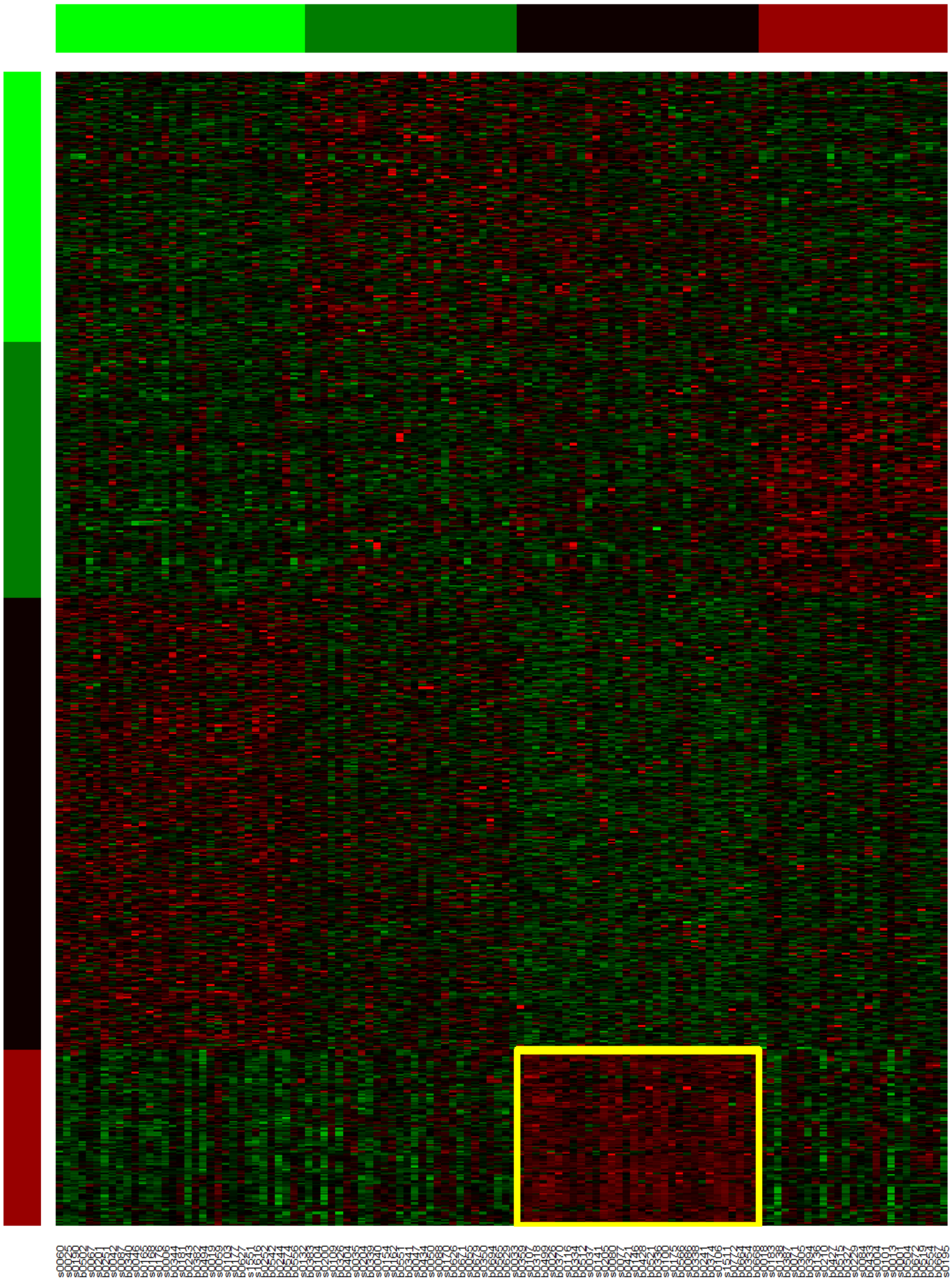

Figure 13: Heatmap of the IRCC-KM bicluster

IRCC-KM genes

ADAM12	ADAMTS2	ADAMTS5	BGN	BGN	BGN	CDH11	CDH11
COL10A1	COL11A1	COL11A1	COL14A1	COL15A1	COL16A1	COL18A1	COL18A1
COL1A1	COL1A1	COL1A1	COL1A2	COL1A2	COL3A1	COL3A1	COL3A1
COL5A1	COL5A1	COL5A1	COL5A2	COL5A2	COL5A3	COL5A3	COL6A1
COL6A1	COL6A1	COL6A1	COL6A2	COL6A2	COL6A3	COL8A1	COL8A2
COL8A2	COMP	CPA3	CSPG2	CSPG2	CSPG2	CSPG2	CTSK
DCN	DCN	DCN	DCN	DPP4	DSPG3	EFEMP1	EFEMP2
EFEMP2	ELN	EMILIN1	FBLN1	FBLN1	FBLN1	FBLN2	FBN1
FBN1	FLRT2	FN1	FN1	FN1	FN1	FN1	FN1
GALNACT_2	HSPG2	ITGA5	ITGAV	ITGB1	ITGB5	ITGB5	ITGB5
ITGB5	ITGBL1	LAMA4	LAMA4	LAMB1	LAMC1	LEPRE1	MATN3
MMP11	MMP11	MMP13	MMP14	MMP14	MMP14	MMP14	MMP19
MMP2	NID2	NRP1	NRP1	PCDH7	PCDH7	PCDH7	PCOLCE
PLXDC1	SDC2	SDC2	SDC2	SERPINE1	SERPINE1	SERPINF1	SERPINH1
SGCD	SLIT3	SPARC	SPARCL1	SPON1	SPON1	SPON1	SPON1
SPON2	THBS1	THBS1	THBS1	THBS2	TIMP3	TIMP3	TIMP3
TIMP3							

IRCC-KM samples

b0359 s0107 b0318 b0499 s0026 b0370 s0116 b0334
b0512 s0037 s0141 s0205 s0080 s0077 b0421 s0146
b0428 b0521 b0336 s0100 s0175 b0566 s0086 b0338
b0241 b0374 s0106 s1511 b0772 b0664 b0354 b0668

3.2.1 Comparing LAS and IRCC-KM biclusters

	No ECM	ECM3
No ECM3	702	14
ECM3	9	120
Jaccard similarity	0.84	

Table 9: Comparing gene lists: LAS (column) vs. IRCC-KM bicluster (row)

	No ECM	ECM3
No ECM3	83	3
ECM3	0	32
Jaccard similarity	0.91	

Table 10: Comparing sample lists: LAS (column) vs. IRCC-KM bicluster (row)

3.3 IRCC-HC bicluster

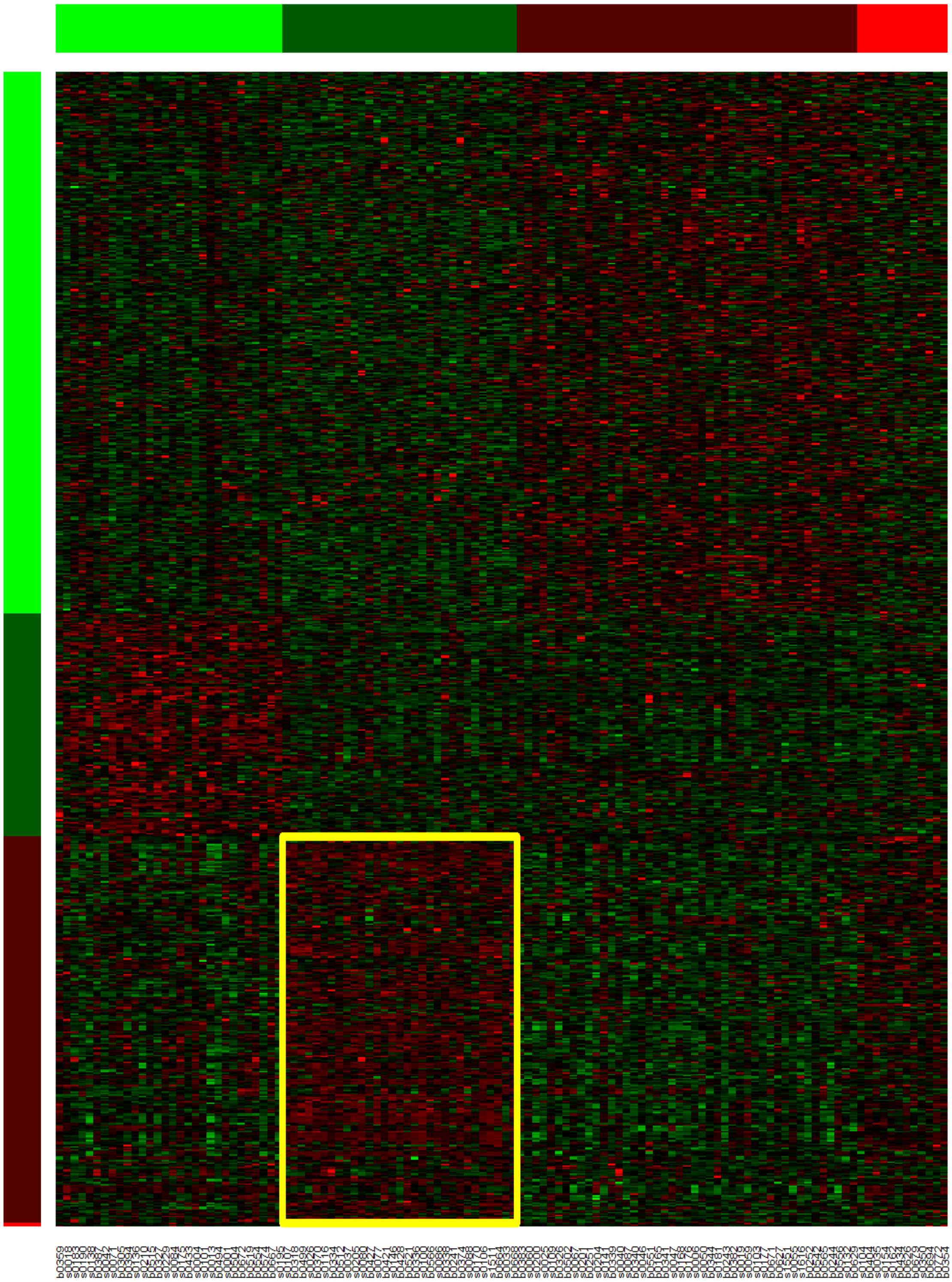

Figure 14: Heatmap of the IRCC-HC bicluster

IRCC-HC genes

ADAM10	ADAM10	ADAM10	ADAM12	ADAM15	ADAM2
ADAM9	ADAMTS2	ADAMTS5	AGT	ALCAM	ALCAM
BGN	BGN	BGN	BST1	BST2	CD164
CD164	CD164	CD36	CD36	CD44	CD44
CD44	CD44	CD44	CD9	CDH1	CDH1
CDH11	CDH11	CDH13	CDH2	CEACAM1	CEACAM1
CEACAM1	CEACAM1	CEACAM1	CEACAM5	CEACAM6	CEACAM7
CHL1	CHPF	CHSY1	CIB1	CLU	CLU
CLU	COL10A1	COL11A1	COL11A1	COL13A1	COL14A1
COL15A1	COL16A1	COL17A1	COL18A1	COL18A1	COL1A1
COL1A1	COL1A1	COL1A2	COL1A2	COL3A1	COL3A1
COL3A1	COL4A1	COL4A1	COL4A2	COL4A2	COL4A3BP
COL4A5	COL5A1	COL5A1	COL5A1	COL5A2	COL5A2
COL5A3	COL5A3	COL6A1	COL6A1	COL6A1	COL6A1
COL6A1	COL6A2	COL6A2	COL6A3	COL7A1	COL8A1
COL8A2	COL8A2	COMP	CPA3	CPD	CPD
CPD	CPD	CPE	CPE	CPM	CSPG2
CSPG2	CSPG2	CSPG2	CSPG6	CSPG6	CSPG6
CTSK	CTSO	DAG1	DAG1	DCN	DCN
DCN	DCN	DCN	DKFZP586H212	DPP4	DPP4
DPP4	DSPG3	ECM1	EFEMP1	EFEMP1	EFEMP2
EFEMP2	ELN	ELN	EMILIN1	ENPEP	ENPEP
FBLN1	FBLN1	FBLN1	FBLN2	FBLN5	FBN1
FBN1	FBN2	FLRT2	FN1	FN1	FN1
FN1	FN1	FN1	GALNACT_2	GPC3	GPC4
GPC4	HSPG2	HSPG2	IBSP	ITGA2	ITGA5
ITGA6	ITGA6	ITGA7	ITGA7	ITGAV	ITGB1
ITGB1	ITGB3	ITGB3BP	ITGB5	ITGB5	ITGB5
ITGB5	ITGB6	ITGB6	ITGBL1	LAMA2	LAMA4
LAMA4	LAMA4	LAMA4	LAMB1	LAMB2	LAMC1
LAMC1	LAMC3	LEPRE1	MATN2	MATN3	MCAM
MCAM	MCAM	MCAM	MGEA5	MGEA5	MGP
MME	MME	MMP10	MMP11	MMP11	MMP13
MMP14	MMP14	MMP14	MMP14	MMP19	MMP2
MMP23B	MMP28	NID2	NRP1	NRP1	NRXN2
OSGEPL1	PCDH12	PCDH17	PCDH7	PCDH7	PCDH7
PCDH9	PCDHA12	PCDHGA1	PCDHGA11	PCDHGA3	PCDHGB7
PCDHGC3	PCDHGC3	PCOLCE	PGCP	PGCP	PLXDC1
PLXDC1	PLXNC1	PLXND1	PLXND1	PRG1	RNPEP
SDC2	SDC2	SDC2	SDF2	SEMA3C	SEMA3C
SEMA5A	SERPINA3	SERPINA5	SERPINB1	SERPINB1	SERPINE1
SERPINE1	SERPINF1	SERPINH1	SGCB	SGCD	SGCD
SGCD	SGCE	SLIT2	SLIT3	SLIT3	SPARC
SPARCL1	SPG20	SPON1	SPON1	SPON1	SPON1
SPON2	STAG1	STAG1	THBS1	THBS1	THBS1
THBS2	THBS4	TIMP1	TIMP3	TIMP3	TIMP3
TIMP3	TIMP4	TNN	TNXB	TNXB	TNXB
TNXB					

IRCC-HC samples

```
s0107 b0318 b0499 s0026 b0370 s0116 b0334 b0512
s0037 s0205 s0080 b0427 s0077 b0421 s0146 b0428
b0521 b0336 s0100 b0566 s0086 b0338 b0241 b0374
s0088 s0170 s0106 s1511 b0664 s0033 b0668
```

3.3.1 Comparing LAS and IRCC-HC biclusters

	No ECM	ECM3
No ECM3	561	1
ECM3	150	133
Jaccard similarity	0.47	

Table 11: Comparing gene lists: LAS (column) vs. IRCC-HC bicluster (row)

	No ECM	ECM3
No ECM3	79	8
ECM3	4	27
Jaccard similarity	0.69	

Table 12: Comparing sample lists: LAS (column) vs. IRCC-HC bicluster (row)

3.4 CCSS bicluster

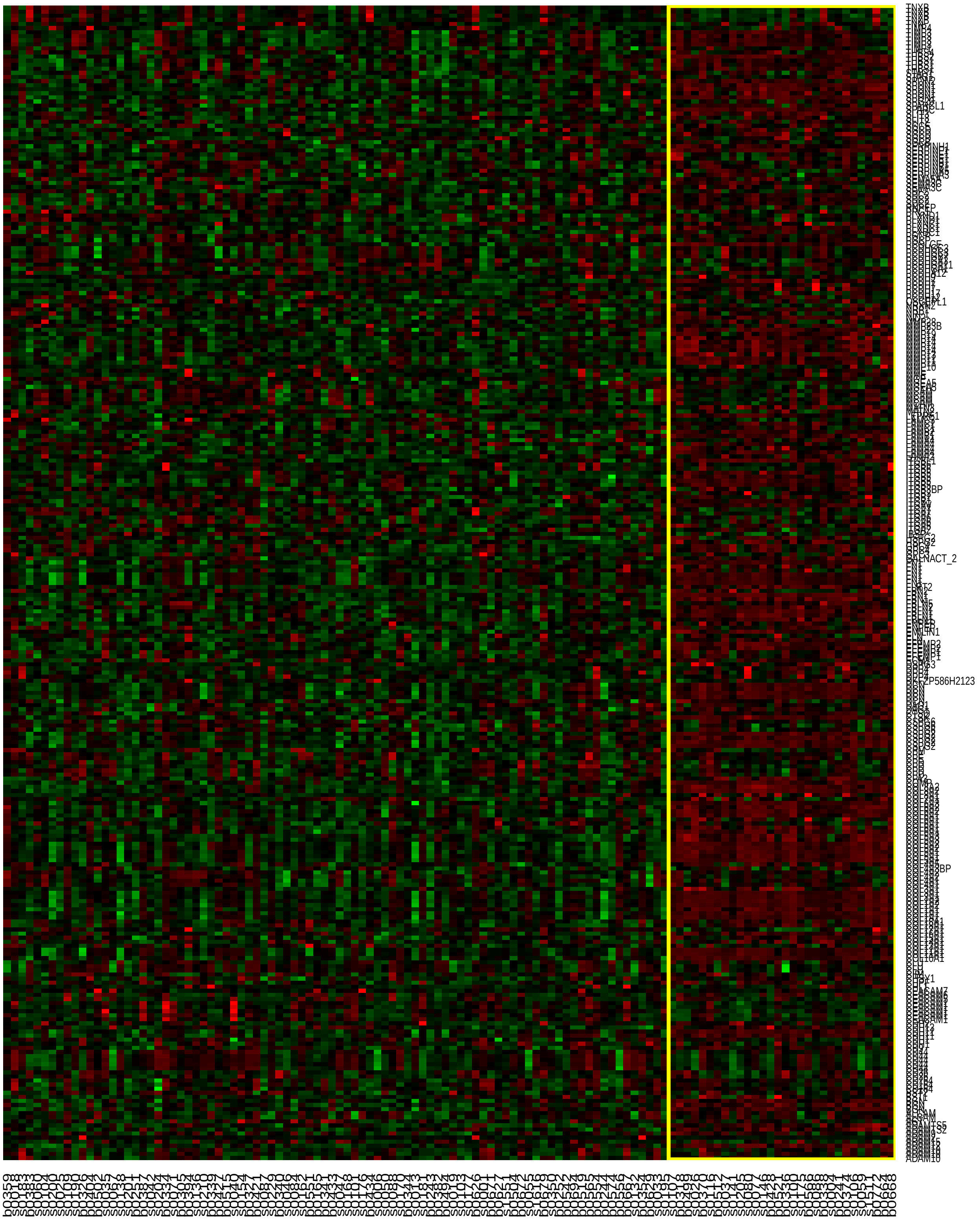

Figure 15: Heatmap of the CCSS bicluster

3.4.1 Comparing LAS and CCSS biclusters

	No ECM	ECM3
No ECM3	83	5
ECM3	0	30
Jaccard similarity	0.86	

Table 13: Comparing sample lists: LAS (column) vs. IRCC-2 (row) biclusters

3.4.2 Prediction strength for CCSS

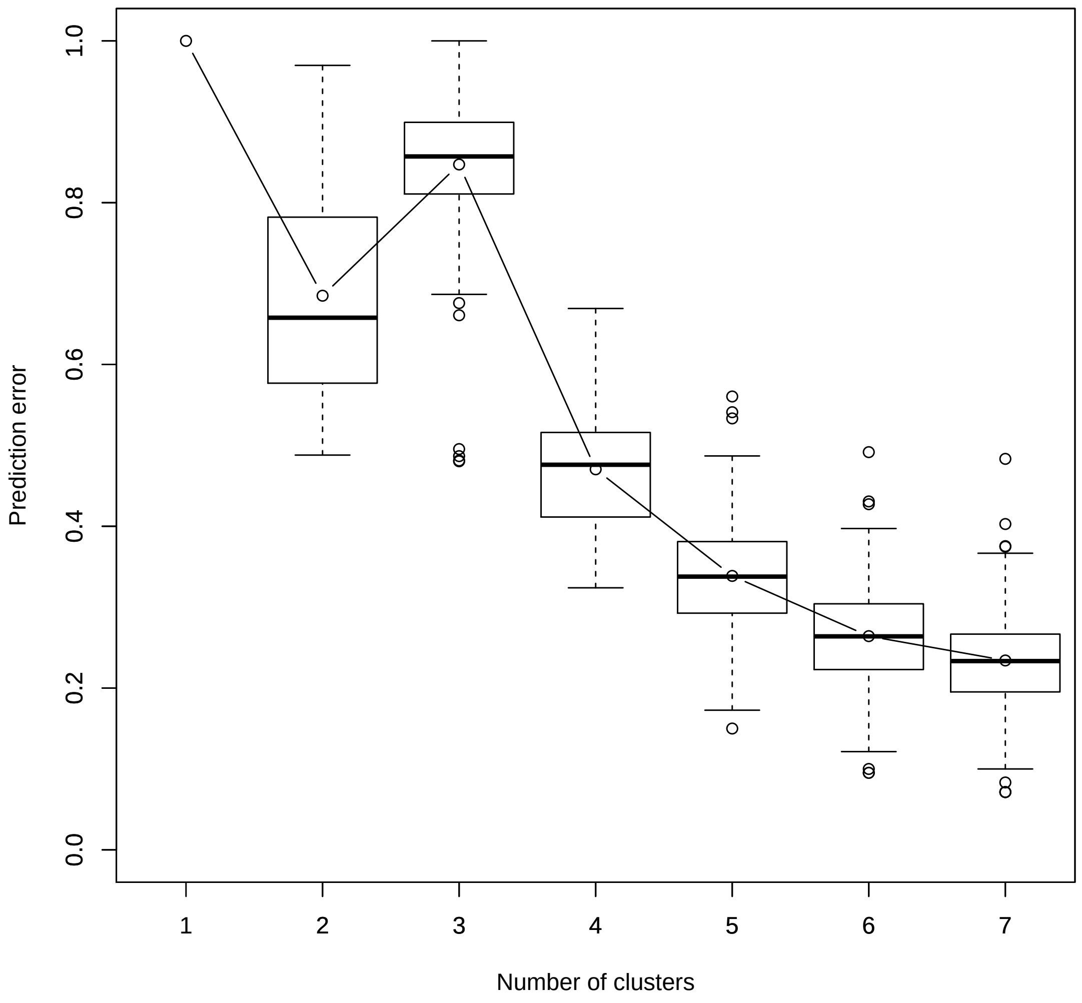

3.4.3 Consensus clustering

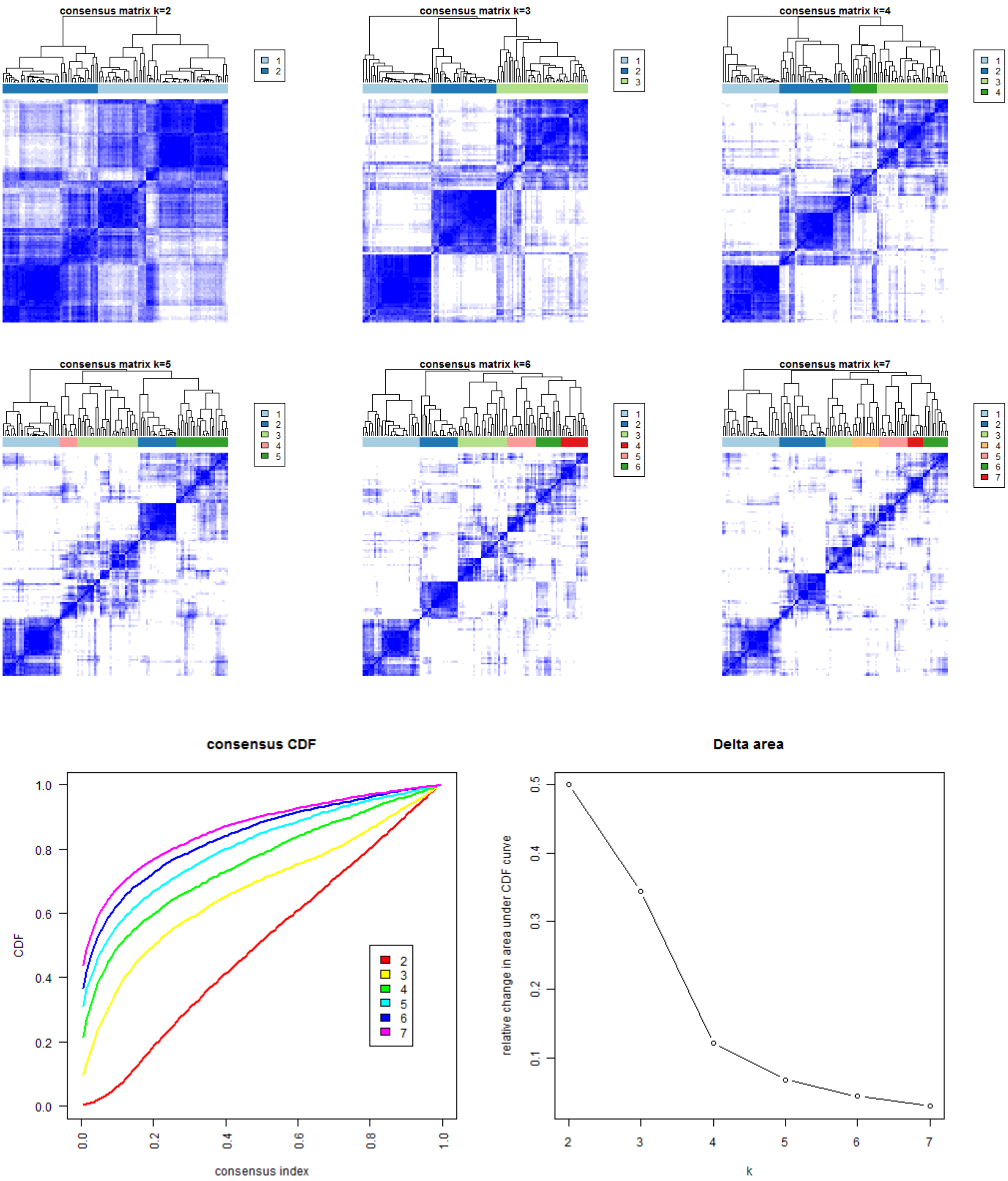

Figure 16: Statistical significance of CCSS clustering (Consensus clustering)

3.4.4 Statistical significance

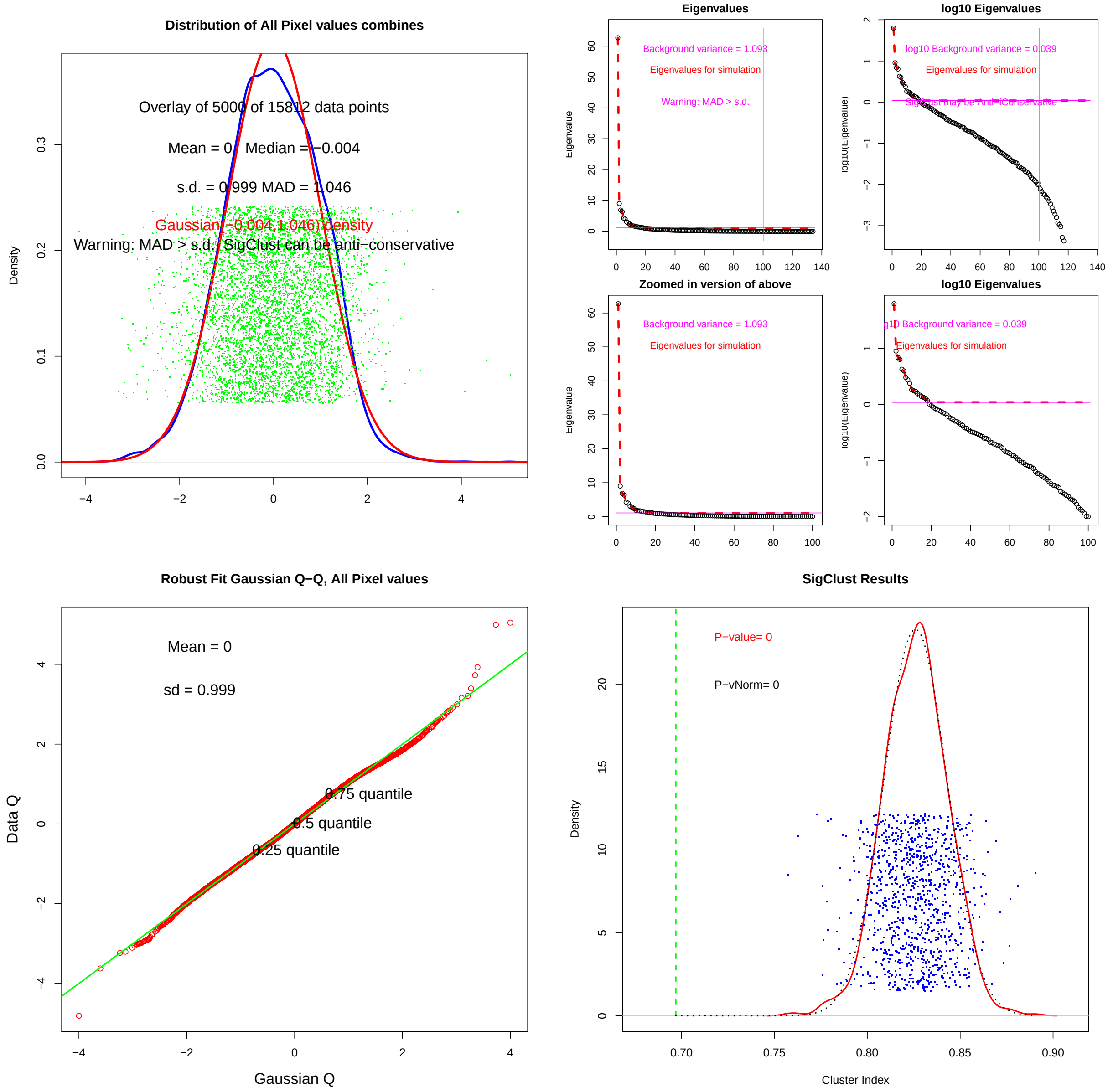

P-value	P-vNorm
0.0E+00	2.4E-14

Table 14: SigClust p-values

3.4.5 Cluster-wise assessment of cluster stability

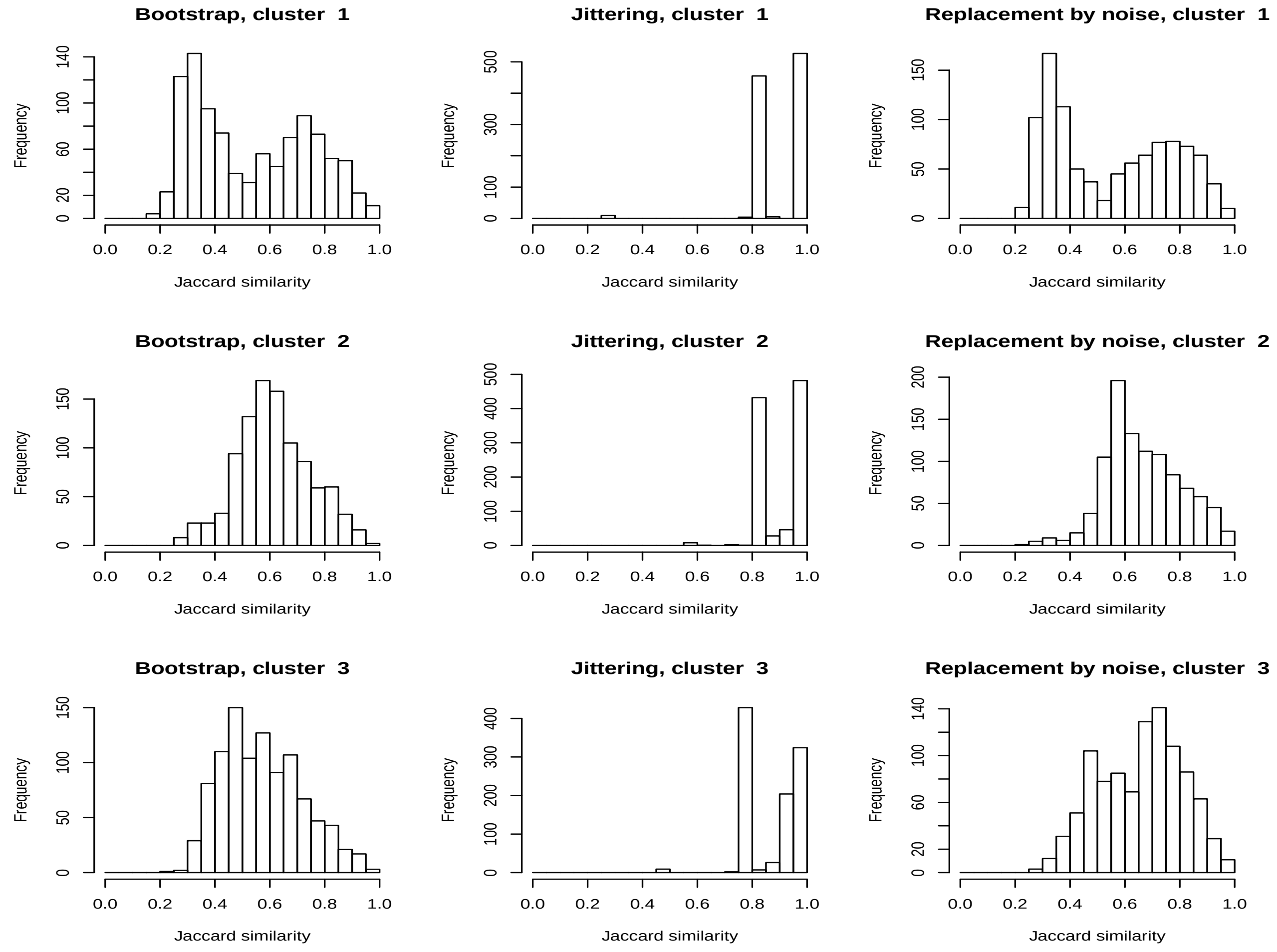


```
* Cluster stability assessment *
Cluster method:  kmeans
Full clustering results are given as parameter result
of the clusterboot object, which also provides further statistics
of the resampling results.
Number of resampling runs:  1000

Number of clusters found in data:  3

 Clusterwise Jaccard bootstrap mean:
[1] 0.5352050 0.6171207 0.5754993
dissolved:
[1] 501 181 373
recovered:
[1] 208 169 131
 Clusterwise Jaccard jittering mean:
[1] 0.9151124 0.8961530 0.8692155
dissolved:
[1] 9 0 9
recovered:
```

```
[1] 991 989 989
 Clusterwise Jaccard replacement by noise mean:
[1] 0.5563464 0.6676355 0.6586267
dissolved:
[1] 480  74 201
recovered:
[1] 260 272 297
```

Removing one sample

```
   Min. 1st Qu.  Median    Mean 3rd Qu.    Max.
 0.8750  1.0000  1.0000  0.9924  1.0000  1.0000
```

Removing one gene

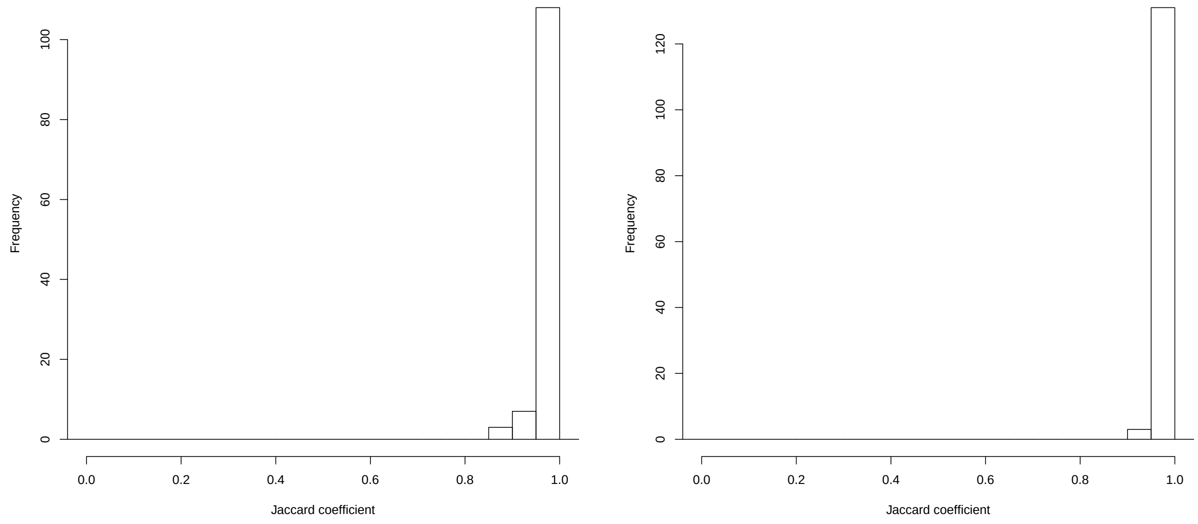

Figure 17: Removing one sample (left) and one gene (right) at a time: distributions of Jaccard coefficients

```
   Min. 1st Qu.  Median    Mean 3rd Qu.    Max.
 0.9394  1.0000  1.0000  0.9951  1.0000  1.0000
```

```
      APN                AD                ADM                FOM
 Min.   :0.3492   Min.   :14.74   Min.   :6.578   Min.   :0.6077
 1st Qu.:0.3492   1st Qu.:14.74   1st Qu.:6.578   1st Qu.:0.7276
 Median :0.3492   Median :14.74   Median :6.578   Median :0.8593
 Mean   :0.3492   Mean   :14.74   Mean   :6.578   Mean   :0.8351
 3rd Qu.:0.3492   3rd Qu.:14.74   3rd Qu.:6.578   3rd Qu.:0.9301
 Max.   :0.3492   Max.   :14.74   Max.   :6.578   Max.   :0.9893
```

Removing sets of k genes

```
   Min. 1st Qu.  Median    Mean 3rd Qu.    Max.
 0.7941  0.8857  0.9118  0.9166  0.9412  1.0000
```

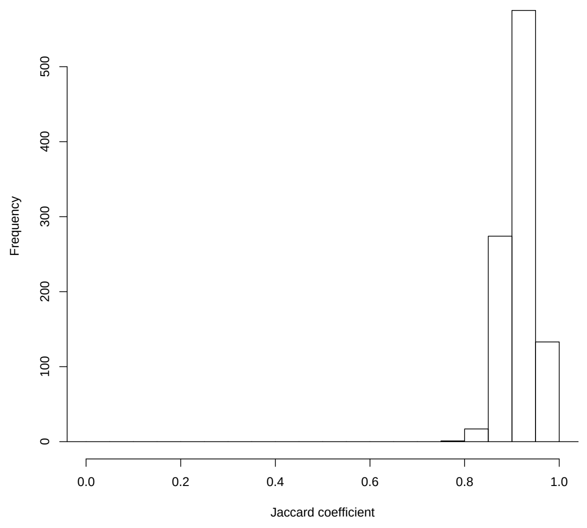

Figure 18: Removing two thirds of the genes: distribution of Jaccard coefficients

3.4.6 Validation measures

```
Clustering Methods:
 kmeans

Cluster sizes:
 2 3 4 5 6

Validation Measures:
                           2        3        4        5        6

kmeans APN            0.0415   0.0042   0.0656   0.0866   0.2005
       AD             0.9580   0.9338   0.9060   0.8707   0.8668
       ADM            0.6142   0.0585   0.7094   0.9381   1.8927
       FOM            0.8307   0.7572   0.7383   0.7278   0.7187
       Connectivity  88.5385 155.1917 166.7317 172.5540 182.4409
       Dunn           0.2086   0.2214   0.2086   0.2421   0.2437
       Silhouette     0.0686   0.0370   0.0356   0.0461   0.0373

Optimal Scores:

             Score   Method Clusters
APN           0.0042 kmeans 3
AD            0.8668 kmeans 6
ADM           0.0585 kmeans 3
FOM           0.7187 kmeans 6
Connectivity 88.5385 kmeans 2
Dunn          0.2437 kmeans 6
Silhouette    0.0686 kmeans 2
```

4 Perou et al. (2000) dataset

4.1 LAS bicluster

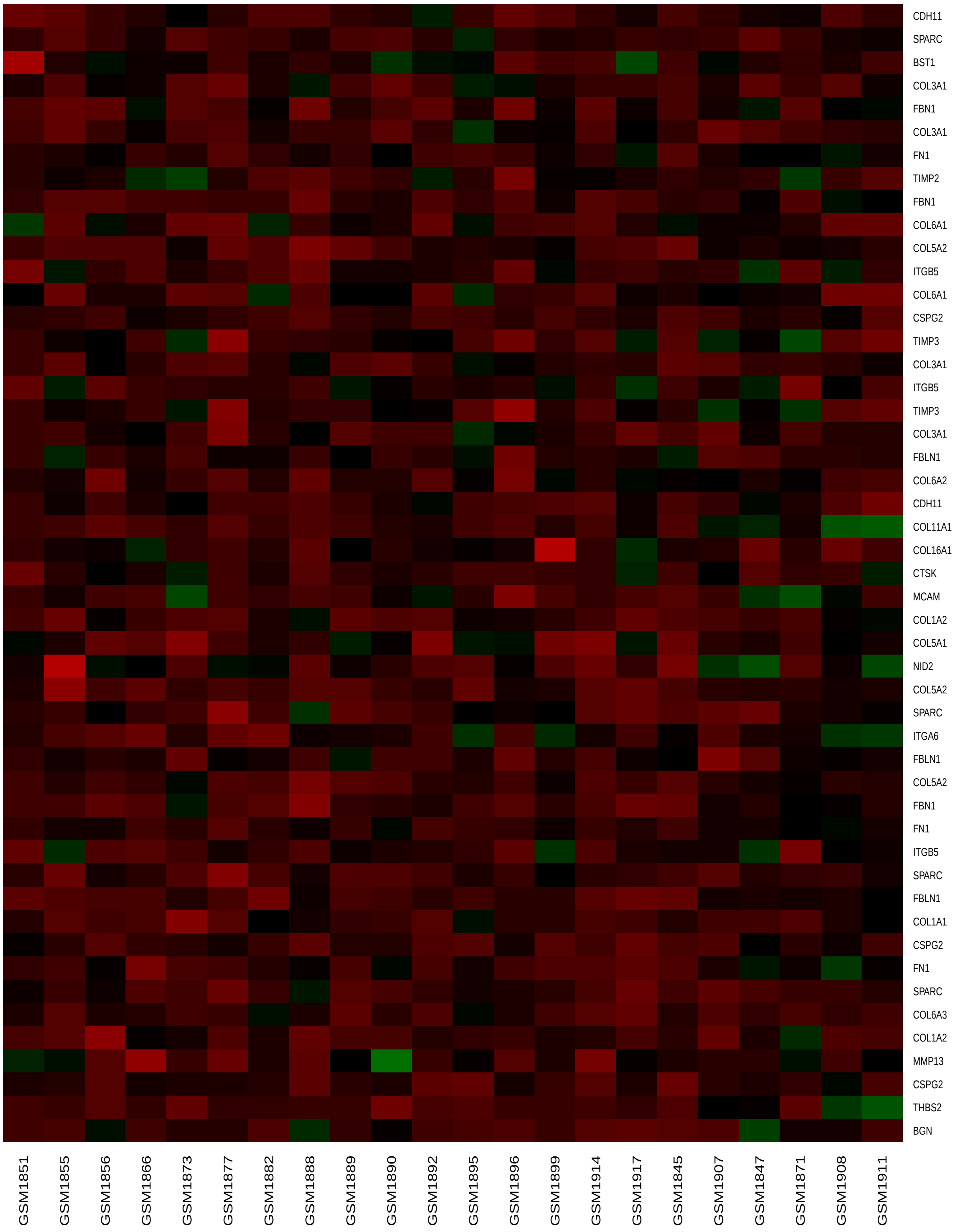

Figure 19: Heatmap of the LAS bicluster

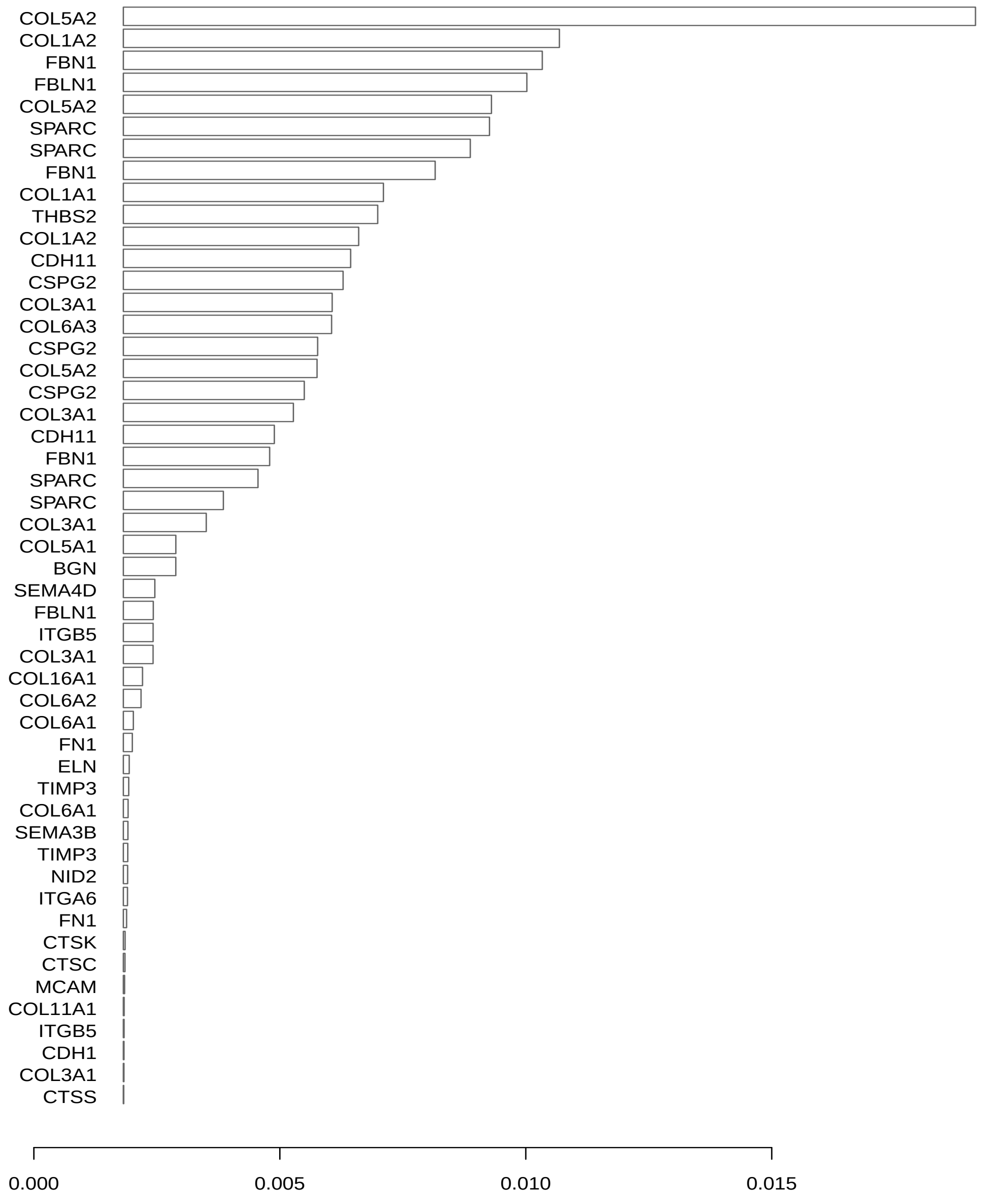

Figure 20: Most informative genes for the LAS bicluster

```
Number of informative genes of the LAS bicluster that also belong
to the ECM3 consensus list: 19 of 34 (56%)
```

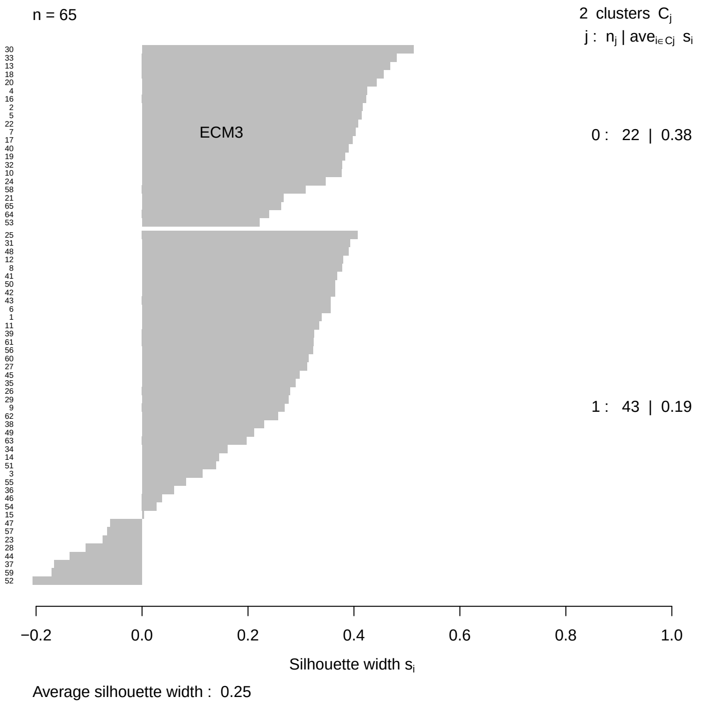

Figure 21: Silhoutte plot for the LAS bicluster

Connectivity	Dunn Index
17.95	0.19

Table 15: Connectivity validation measure and Dunn Index of LAS partitioning

4.2 IRCC-KM bicluster

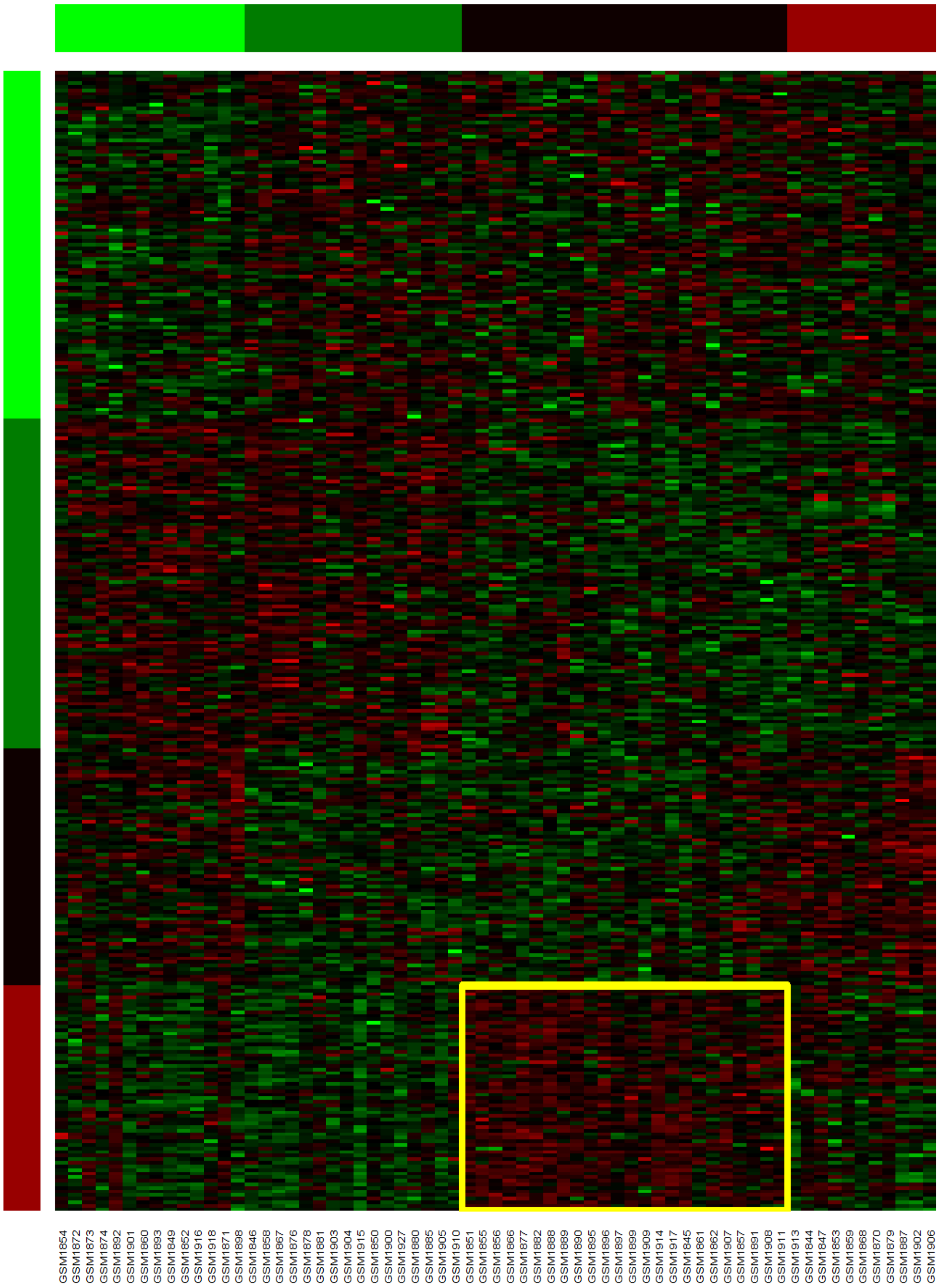

Figure 22: Heatmap of the IRCC-KM bicluster

IRCC-KM genes

```
BGN      THBS2    CSPG2    MMP13    COL1A2   COL6A3   SERPING1 SPARC
FN1      CSPG2    COL1A1   FBLN1    SPARC    ITGB5    FN1      FN1
FBN1     COL3A1   COL5A2   FBLN1    ITGA6    ITGA6    SPARC    COL5A2
FN1      NID2     COL5A1   COL6A2   COL1A2   MCAM     CTSK     COL16A1
THBS1    COL11A1  CDH11    COL6A2   FBLN1    ITGAV    COL3A1   ITGAV
SPON1    MMP3     TIMP3    ITGB5    FBLN2    COL3A1   TIMP3    CSPG2
COL6A1   ITGB5    COL5A2   COL6A1   FBN1     TIMP2    SERPING1 FN1
COL3A1   FBN1     COL3A1   MMP9     BST1     SPARC    CDH11
```

IRCC-KM samples

```
GSM1851 GSM1855 GSM1856 GSM1866 GSM1877 GSM1882 GSM1888 GSM1889
GSM1890 GSM1895 GSM1896 GSM1897 GSM1899 GSM1909 GSM1914 GSM1917
GSM1845 GSM1861 GSM1862 GSM1907 GSM1857 GSM1891 GSM1908 GSM1911
```

4.2.1 Comparing LAS and IRCC-KM biclusters

	No ECM	ECM3
No ECM3	255	0
ECM3	14	49
Jaccard similarity	0.78	

Table 16: Comparing gene lists: LAS (column) vs. IRCC-KM bicluster (row)

	No ECM	ECM3
No ECM3	37	4
ECM3	6	18
Jaccard similarity	0.64	

Table 17: Comparing sample lists: LAS (column) vs. IRCC-KM bicluster (row)

4.3 IRCC-HC bicluster

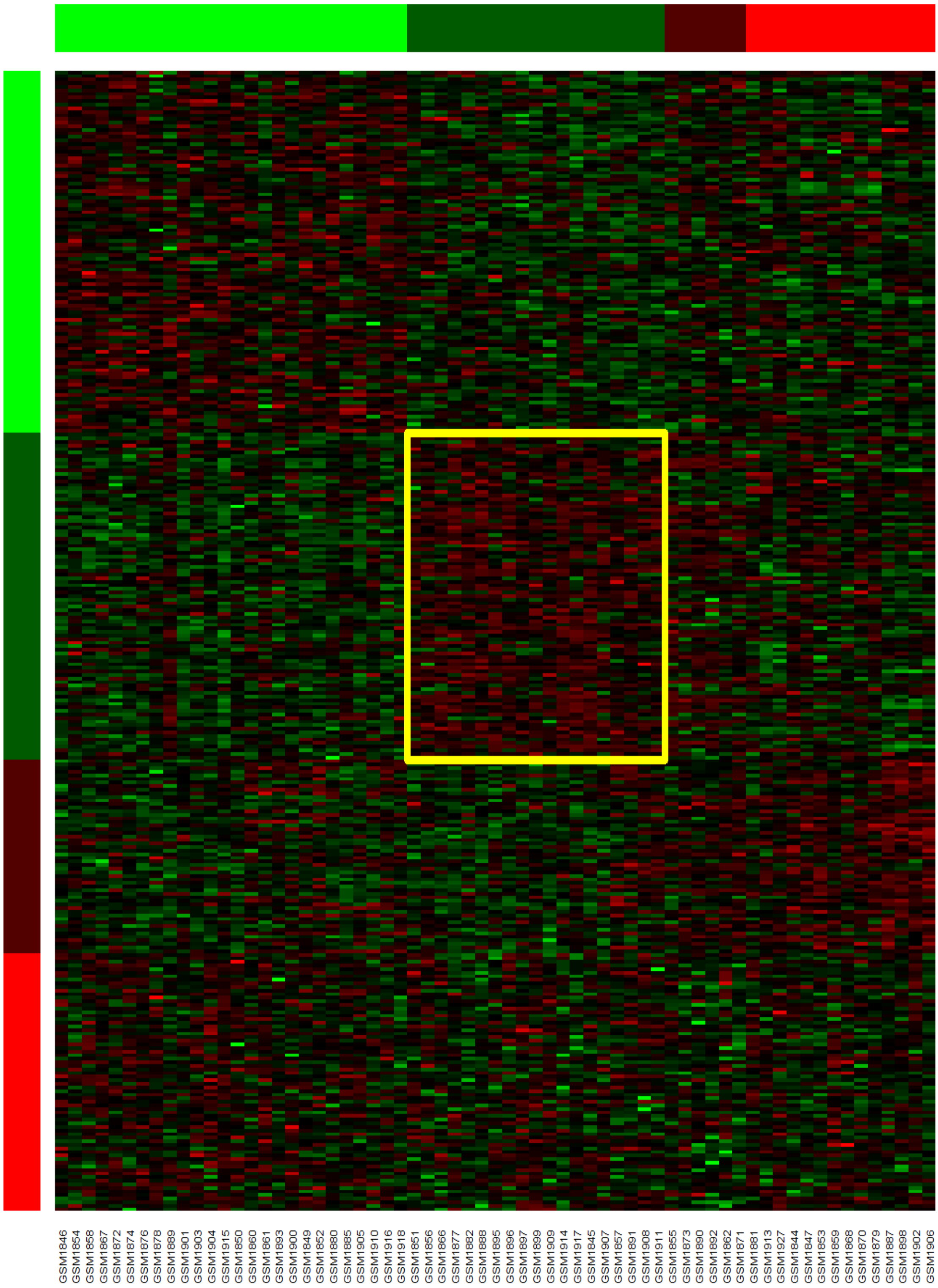

Figure 23: Heatmap of the IRCC-HC bicluster

IRCC-HC genes

APP	BGN	BST1	CDH11	CDH11	CDH13	CDH13	CDH8
CNTN1	COL11A1	COL16A1	COL1A1	COL1A2	COL1A2	COL3A1	COL3A1
COL3A1	COL3A1	COL3A1	COL4A1	COL4A1	COL4A1	COL4A1	COL4A2
COL5A1	COL5A2	COL5A2	COL5A2	COL6A1	COL6A1	COL6A2	COL6A3
CSPG2	CSPG2	CSPG2	CSPG4	CTSD	CTSE	CTSK	ECM1
FBLN1	FBLN1	FBLN1	FBLN2	FBN1	FBN1	FBN1	FN1
FN1	FN1	FN1	FN1	ITGA2	ITGA2	ITGA3	ITGA3
ITGA6	ITGA6	ITGAV	ITGAV	ITGB1	ITGB1	ITGB5	ITGB5
ITGB5	LAMC1	MCAM	MMP13	MMP3	MMP9	NID2	NRP2
PCDHGC3	PLXNA2	SDC2	SERPINA7	SERPINE1	SERPINF1	SGCB	SPARC
SPARC	SPARC	SPARC	SPON1	THBS1	THBS1	THBS2	TIMP2
TIMP3	TIMP3	TNXB					

IRCC-HC samples

GSM1851 GSM1856 GSM1866 GSM1877 GSM1882 GSM1888 GSM1895 GSM1896
GSM1897 GSM1899 GSM1909 GSM1914 GSM1917 GSM1845 GSM1907 GSM1857
GSM1891 GSM1908 GSM1911

4.3.1 Comparing LAS and IRCC-HC biclusters

	No ECM	ECM3
No ECM3	227	0
ECM3	42	49
Jaccard similarity	0.54	

Table 18: Comparing gene lists: LAS (column) vs. IRCC-HC bicluster (row)

	No ECM	ECM3
No ECM3	39	7
ECM3	4	15
Jaccard similarity	0.58	

Table 19: Comparing sample lists: LAS (column) vs. IRCC-HC bicluster (row)

4.4 CCSS bicluster

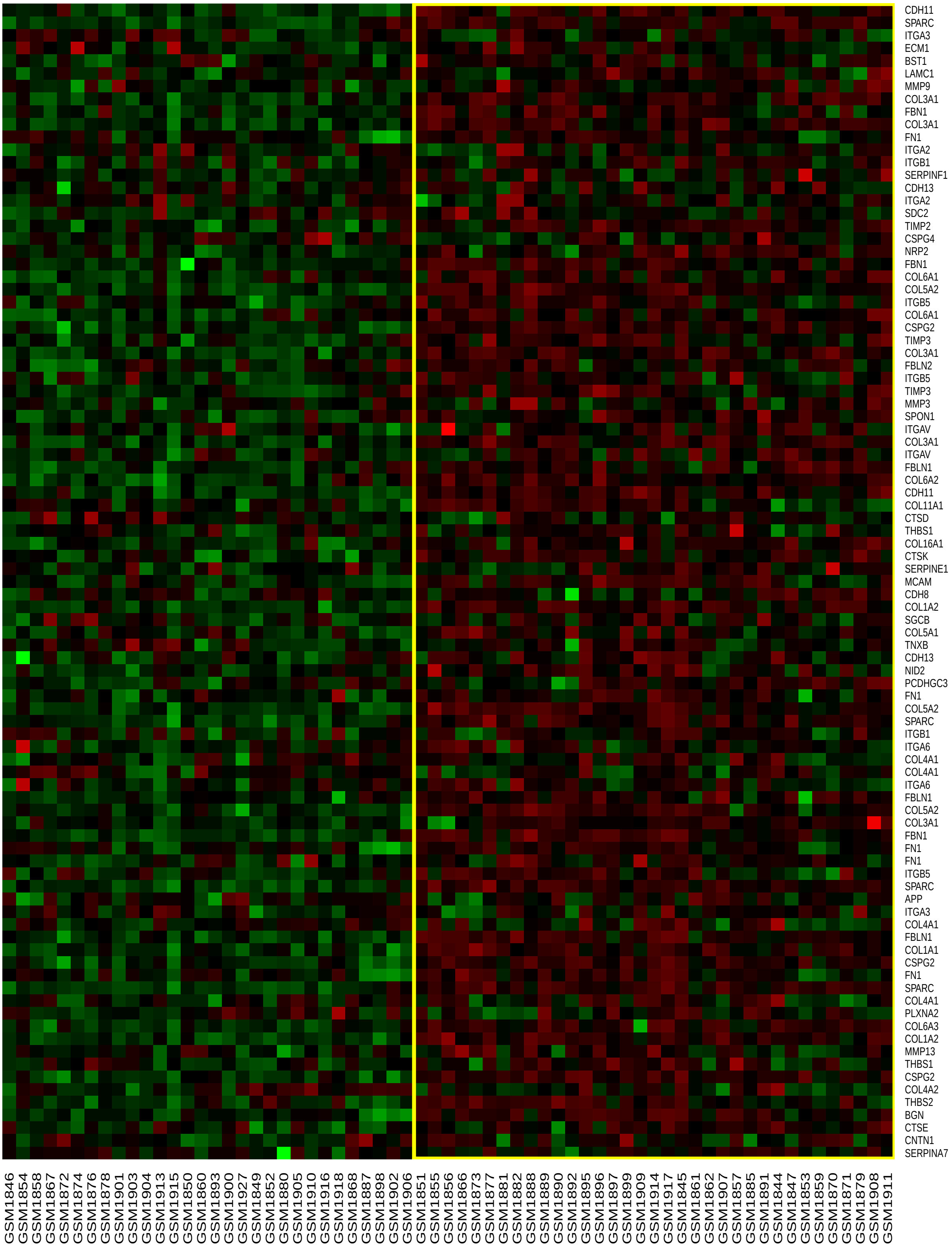

Figure 24: Heatmap of the CCSS bicluster

4.4.1 Comparing LAS and CCSS biclusters

	No ECM	ECM3
No ECM3	30	0
ECM3	13	22
Jaccard similarity	0.63	

Table 20: Comparing sample lists: LAS (column) vs. CCSS (row) biclusters

4.4.2 Prediction strength for CCSS

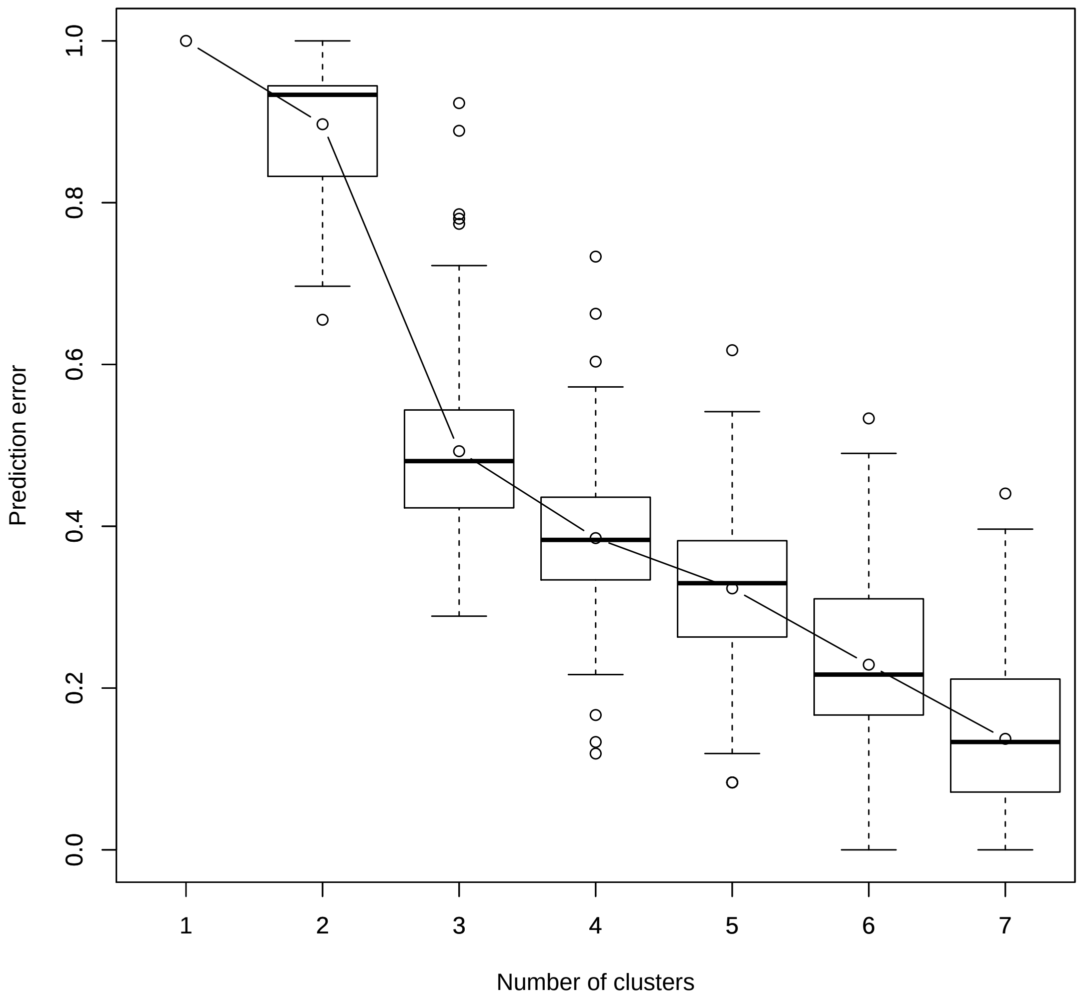

4.4.3 Consensus clustering

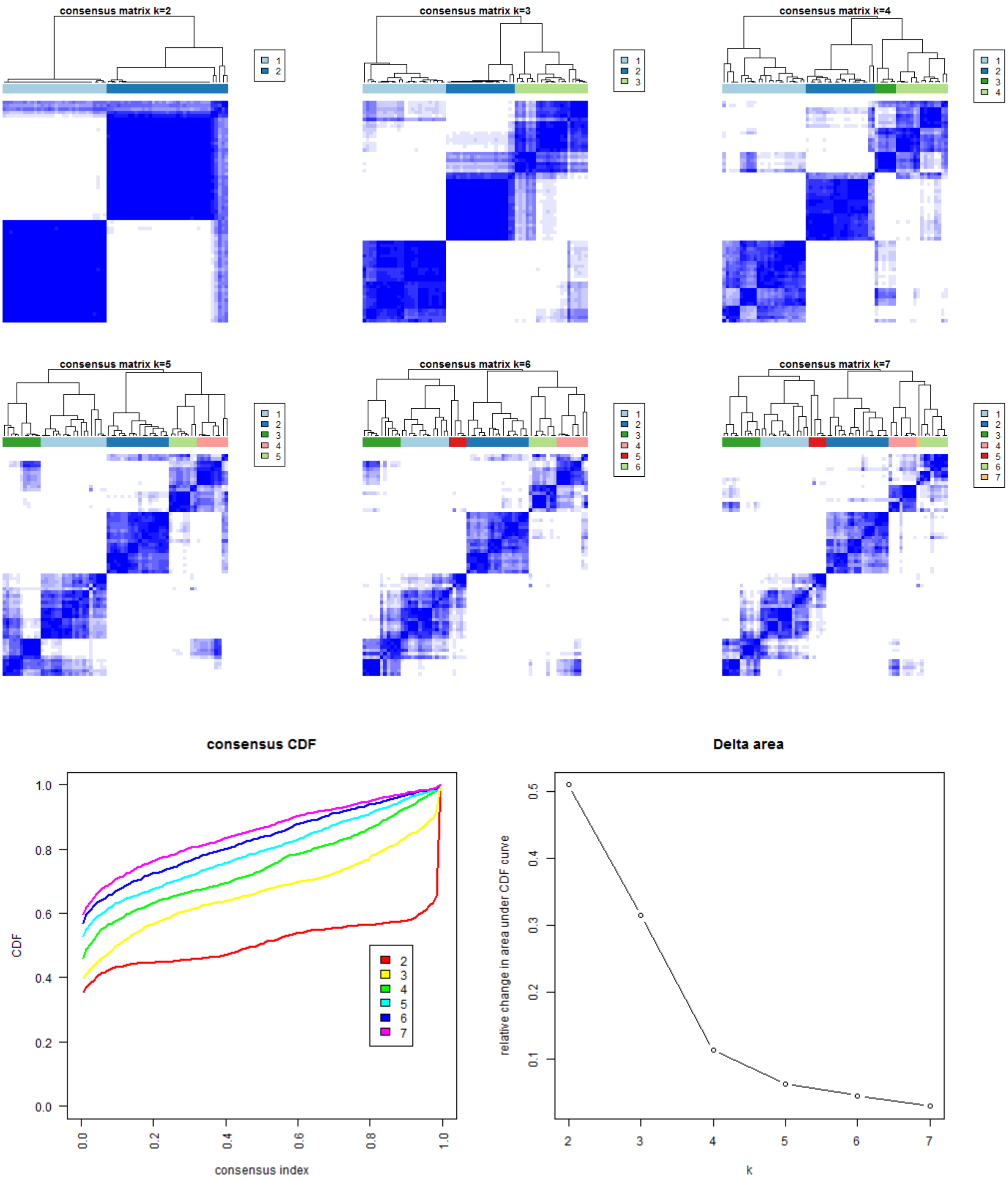

Figure 25: Statistical significance of CCSS clustering (Consensus clustering)

4.4.4 Statistical significance

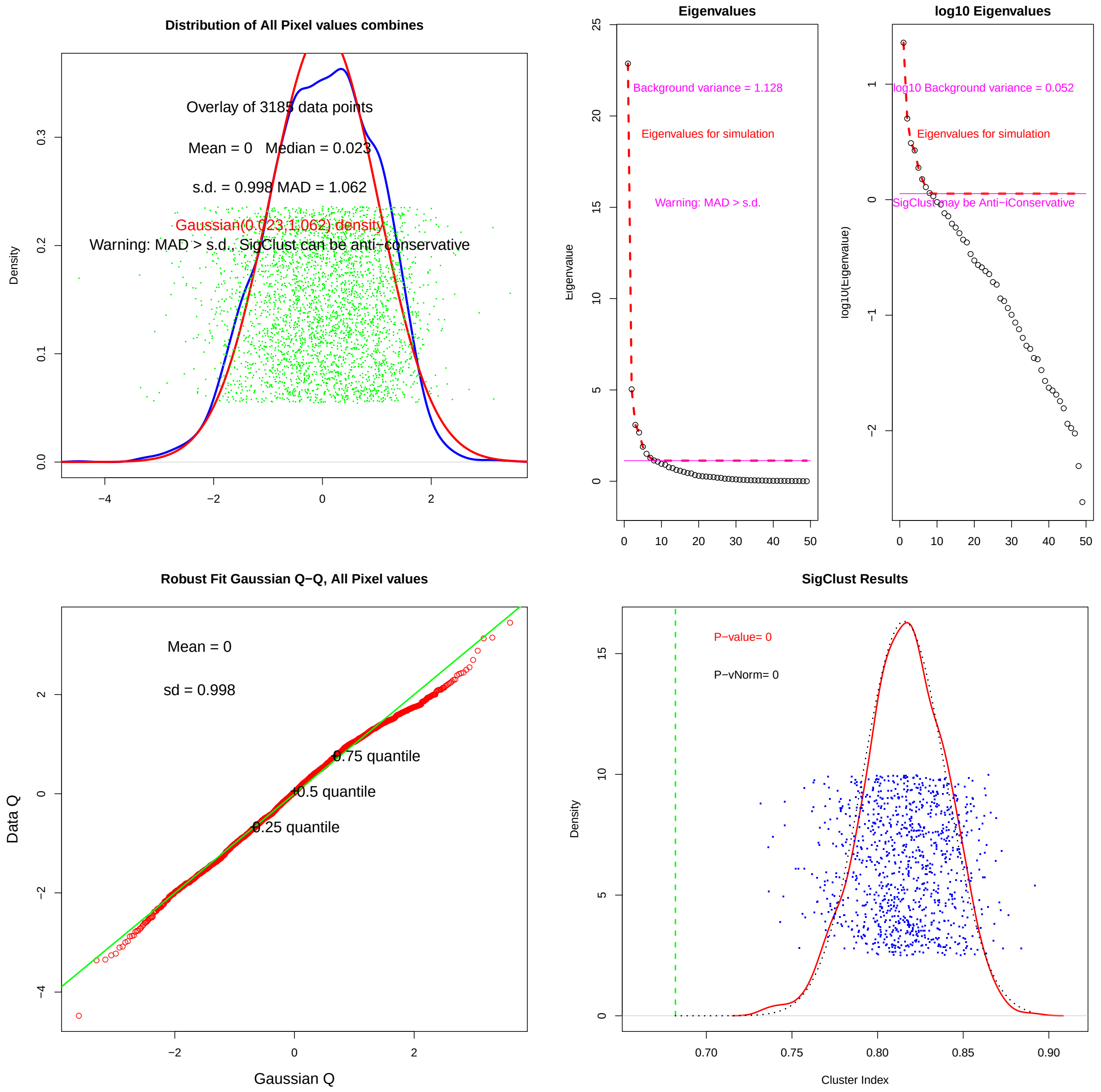

P-value	P-vNorm
0.0E+00	2.3E-08

Table 21: SigClust p-values

4.4.5 Cluster-wise assessment of cluster stability

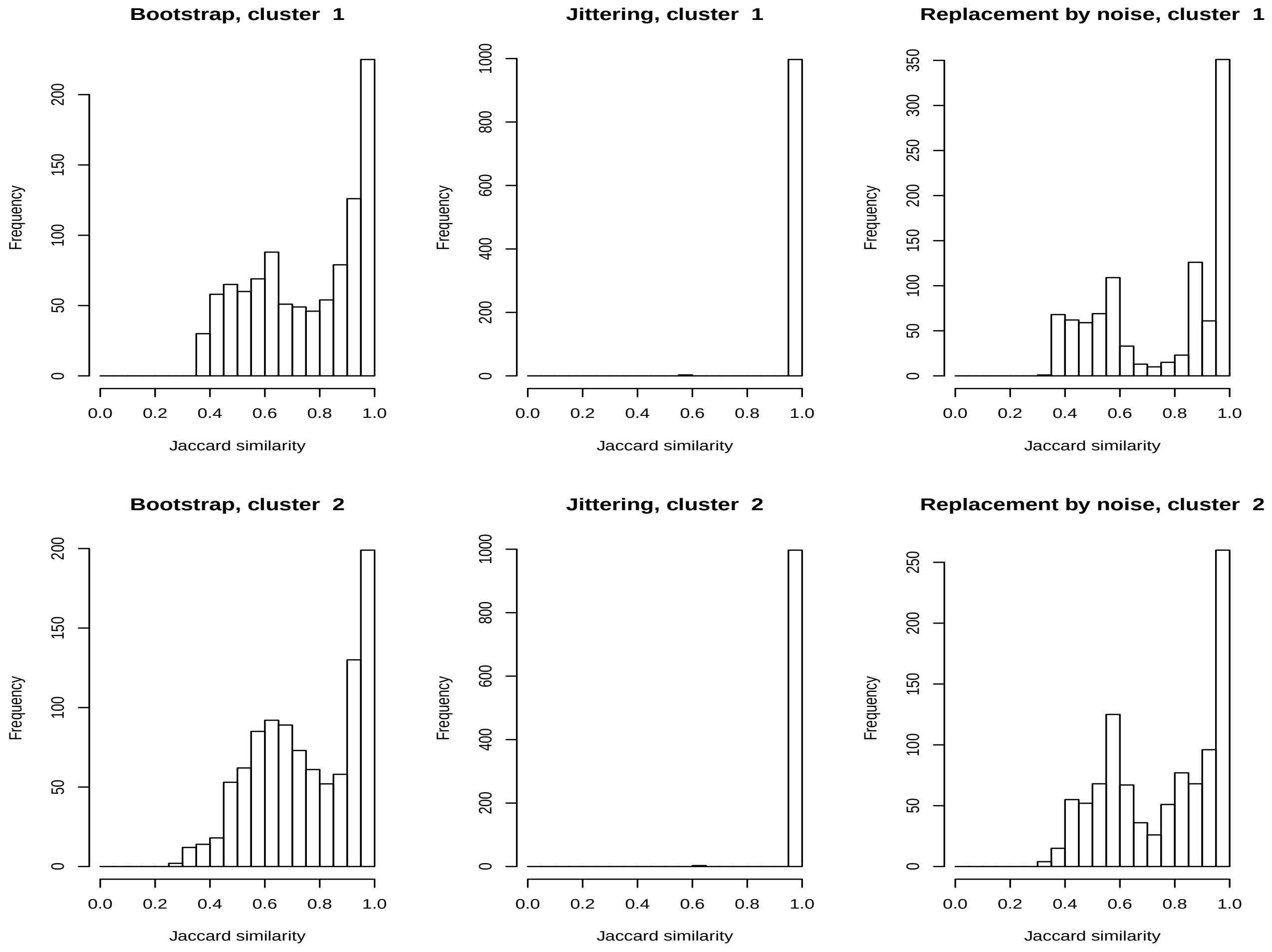


```
* Cluster stability assessment *
Cluster method:  kmeans
Full clustering results are given as parameter result
of the clusterboot object, which also provides further statistics
of the resampling results.
Number of resampling runs:  1000

Number of clusters found in data:  2

 Clusterwise Jaccard bootstrap mean:
[1] 0.7579851 0.7579400
dissolved:
[1] 153  99
recovered:
[1] 530 500
 Clusterwise Jaccard jittering mean:
[1] 0.9987586 0.9988750
dissolved:
[1] 0 0
recovered:
```

```
[1] 997 997
 Clusterwise Jaccard replacement by noise mean:
[1] 0.7614243 0.7630827
dissolved:
[1] 190 126
recovered:
[1] 576 552
```

Removing one sample

```
   Min. 1st Qu.  Median    Mean 3rd Qu.    Max.
 0.9697  1.0000  1.0000  0.9941  1.0000  1.0000
```

Removing one gene

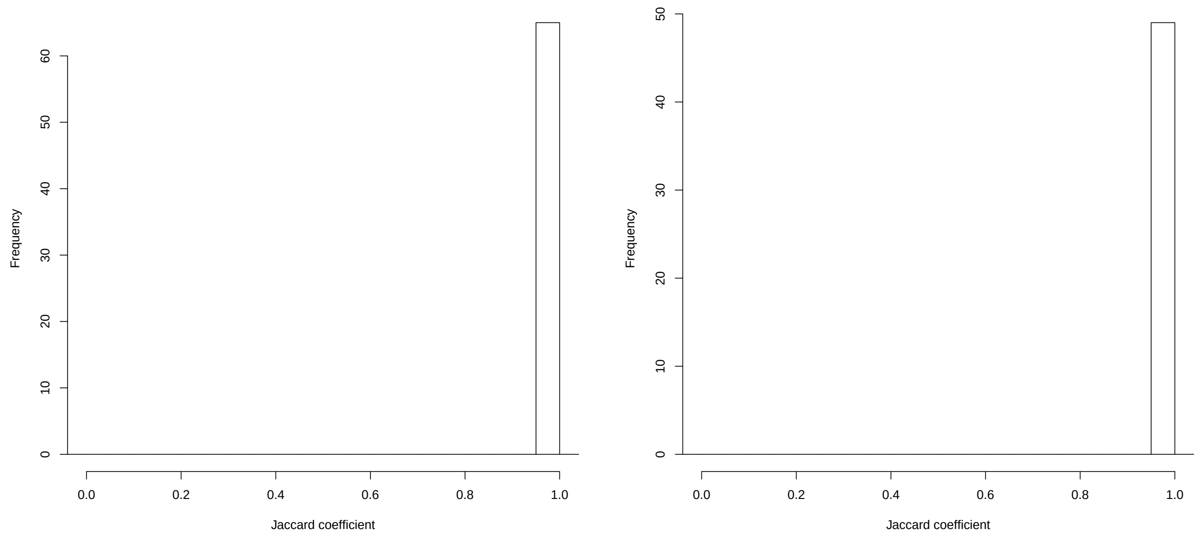

Figure 26: Removing one sample (left) and one gene (right) at a time: distributions of Jaccard coefficients

```
   Min. 1st Qu.  Median    Mean 3rd Qu.    Max.
 0.9706  0.9714  1.0000  0.9865  1.0000  1.0000
```

```
      APN                 AD                ADM                FOM
 Min.   :0.2519   Min.   :8.306   Min.   :2.478   Min.   :0.5847
 1st Qu.:0.2662   1st Qu.:8.349   1st Qu.:2.665   1st Qu.:0.7072
 Median :0.2662   Median :8.349   Median :2.665   Median :0.8328
 Mean   :0.2684   Mean   :8.355   Mean   :2.684   Mean   :0.8130
 3rd Qu.:0.2791   3rd Qu.:8.387   3rd Qu.:2.807   3rd Qu.:0.8998
 Max.   :0.2791   Max.   :8.387   Max.   :2.807   Max.   :0.9886
```

Removing sets of k genes

```
   Min. 1st Qu.  Median    Mean 3rd Qu.    Max.
 0.5714  0.8684  0.9118  0.8981  0.9412  1.0000
```

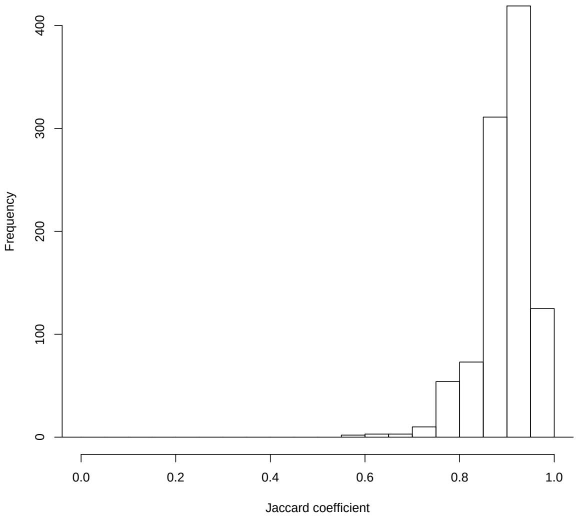

Figure 27: Removing two thirds of the genes: distribution of Jaccard coefficients

4.4.6 Validation measures

```
Clustering Methods:
 kmeans

Cluster sizes:
 2 3 4 5 6

Validation Measures:
                              2       3       4       5       6

kmeans APN             0.0140  0.0272  0.1325  0.1264  0.1242
       AD              7.6754  7.1799  7.0374  6.6685  6.5174
       ADM             0.1559  0.2487  1.0008  0.9022  0.8359
       FOM             0.8130  0.7705  0.7644  0.7333  0.7242
       Connectivity   10.2766 24.3758 28.1075 35.2214 38.0075
       Dunn            0.3951  0.4836  0.4418  0.5190  0.5306
       Silhouette      0.2902  0.1801  0.1582  0.1697  0.1666

Optimal Scores:

             Score   Method Clusters
APN           0.0140 kmeans 2
AD            6.5174 kmeans 6
ADM           0.1559 kmeans 2
FOM           0.7242 kmeans 6
Connectivity 10.2766 kmeans 2
Dunn          0.5306 kmeans 6
Silhouette    0.2902 kmeans 2
```

5 Ma et al. (2004) dataset (GDS806)

5.1 LAS bicluster

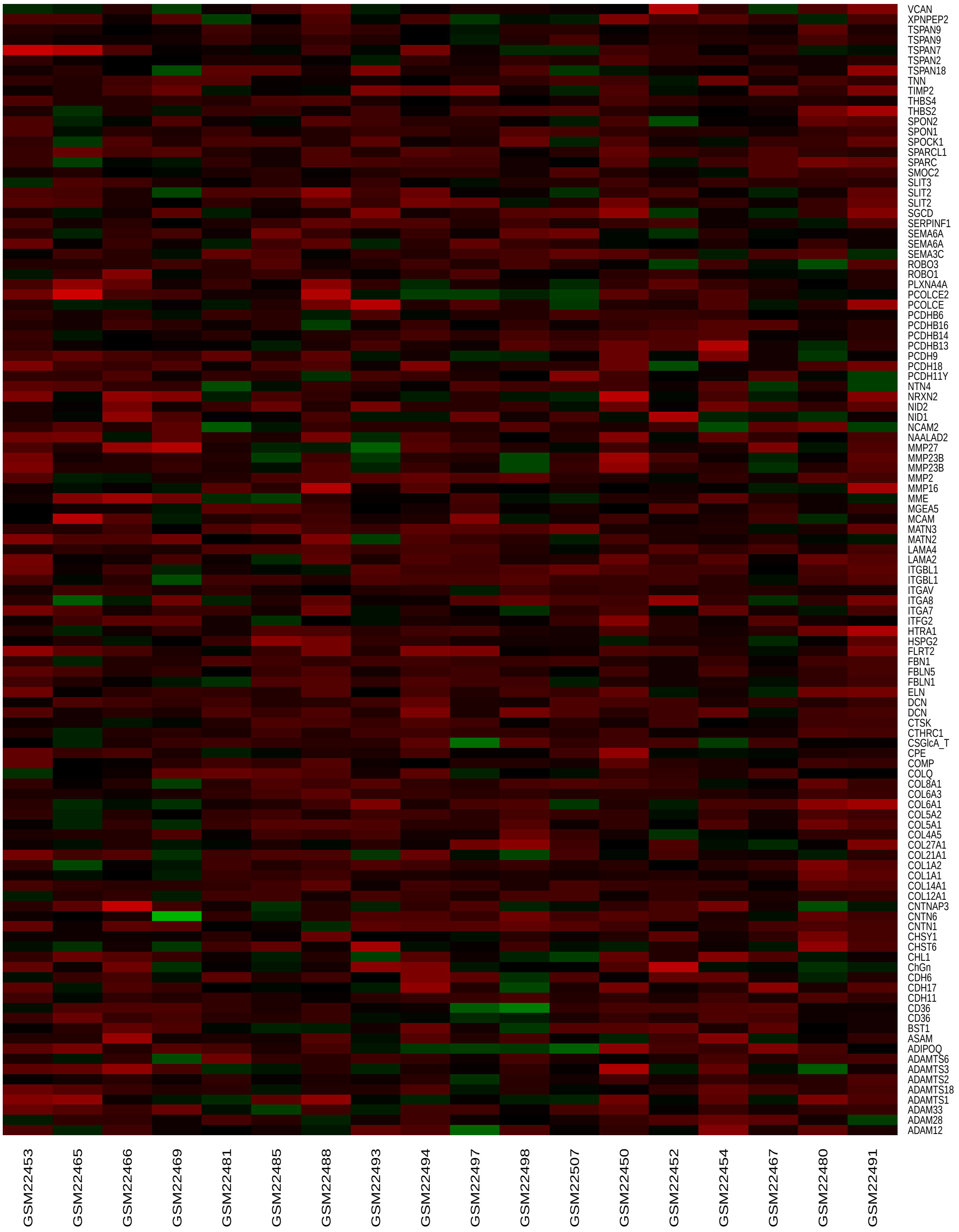

Figure 28: Heatmap of the LAS bicluster

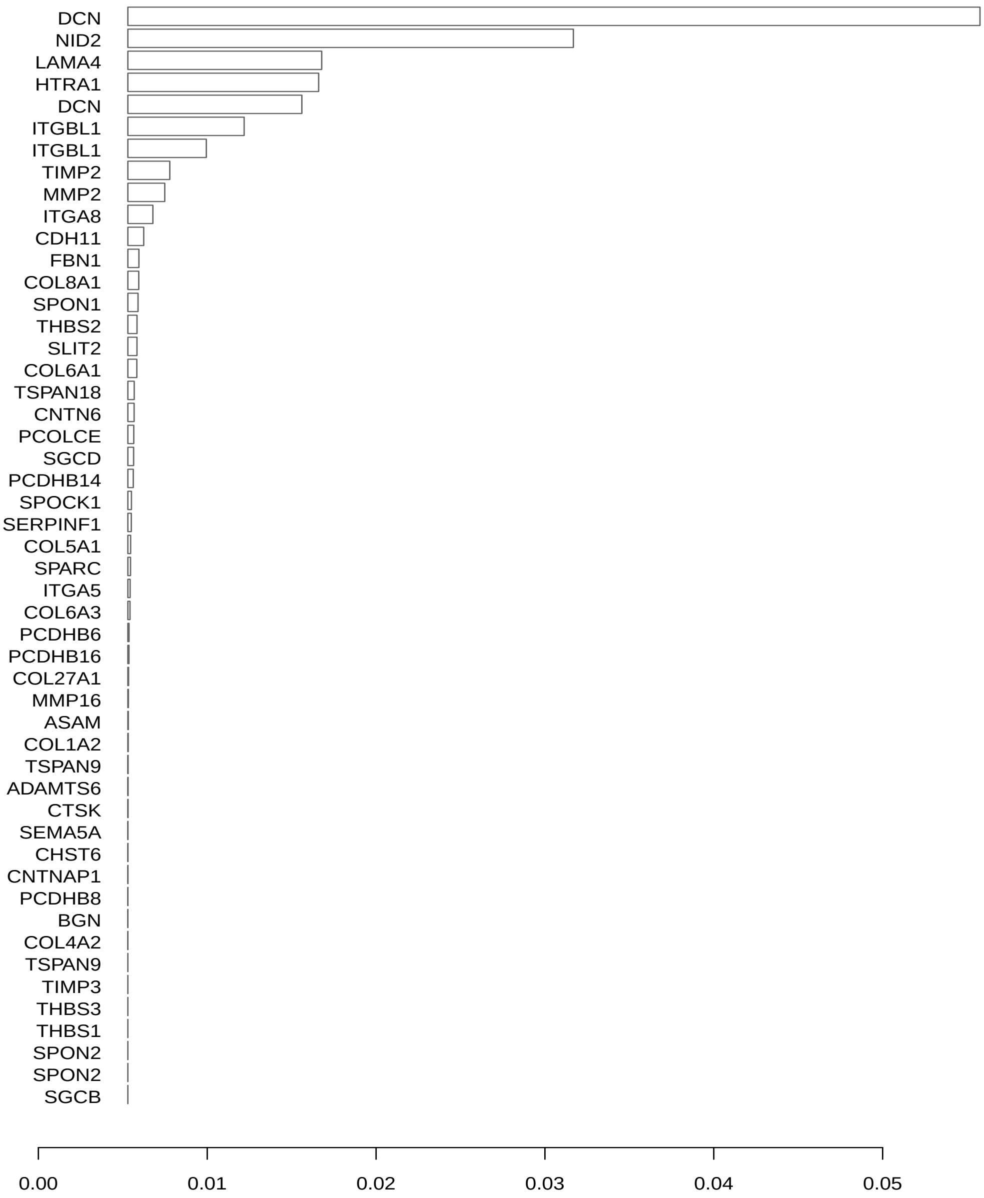

Figure 29: Most informative genes for the LAS bicluster

```
Number of informative genes of the LAS bicluster that also belong
to the ECM3 consensus list: 21 of 34 (62%)
```

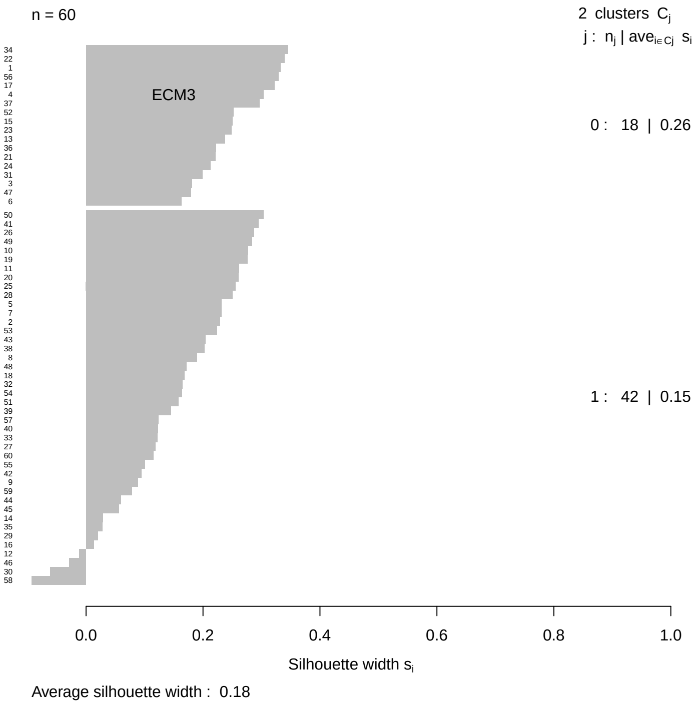

Figure 30: Silhoutte plot for the LAS bicluster

Connectivity	Dunn Index
7.42	0.53

Table 22: Connectivity validation measure and Dunn Index of LAS partitioning

5.2 IRCC-KM bicluster

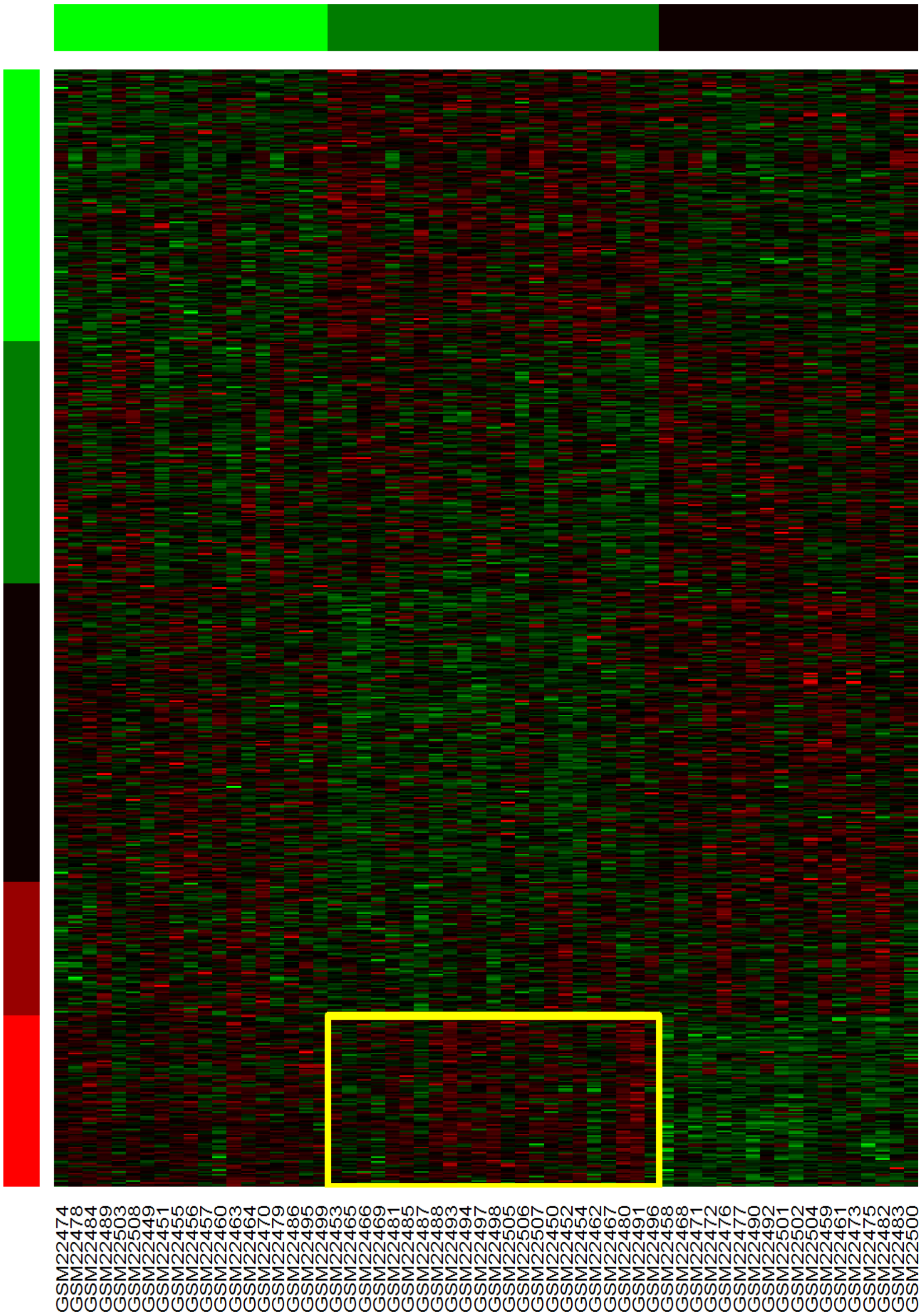

Figure 31: Heatmap of the IRCC-KM bicluster

IRCC-KM genes

ADAM12	ADAM19	ADAM23	ADAM7	ADAMTS12	ADAMTS2	ADAMTS4	ADAMTS6
ASAM	BGN	CDH11	CDH2	CDH3	CHST3	CHST6	CNTN6
CNTNAP1	COL10A1	COL11A1	COL12A1	COL13A1	COL18A1	COL1A1	COL1A2
COL27A1	COL4A1	COL4A2	COL5A1	COL5A2	COL5A3	COL6A1	COL6A3
COL7A1	COL8A1	CORIN	CSGlcA_T	CTHRC1	CTSK	DCN	DCN
EFEMP2	EMILIN1	EMILIN1	FBLN1	FBLN2	FBN1	GPC6	HABP4
HAS2	HSPG2	HTRA1	ITGA11	ITGA5	ITGA8	ITGB5	ITGBL1
ITGBL1	LAMA4	LEPRE1	MMP11	MMP11	MMP13	MMP14	MMP16
MMP2	MMP28	NID2	NRP1	NRP2	PCDH7	PCDHB14	PCDHB16
PCDHB2	PCDHB6	PCDHB7	PCDHB8	PCDHGA3	PCOLCE	PLXDC2	PLXNA1
PLXNA2	PLXNB3	SEMA3A	SEMA5A	SERPINF1	SERPINH1	SGCB	SGCD
SLIT2	SPARC	SPG21	SPOCK1	SPON1	SPON2	SPON2	THBS1
THBS2	THBS3	TIMP2	TIMP3	TNC	TSPAN18	TSPAN2	TSPAN9
TSPAN9	VCAN						

IRCC-KM samples

GSM22453	GSM22465	GSM22466	GSM22469	GSM22481	GSM22485	GSM22487	GSM22488
GSM22493	GSM22494	GSM22497	GSM22498	GSM22505	GSM22506	GSM22507	GSM22450
GSM22452	GSM22454	GSM22462	GSM22467	GSM22480	GSM22491	GSM22496	

5.2.1 Comparing LAS and IRCC-KM biclusters

	No ECM	ECM3
No ECM3	524	61
ECM3	56	50
Jaccard similarity	0.30	

Table 23: Comparing gene lists: LAS (column) vs. IRCC-KM bicluster (row)

	No ECM	ECM3
No ECM3	37	0
ECM3	5	18
Jaccard similarity	0.78	

Table 24: Comparing sample lists: LAS (column) vs. IRCC-KM bicluster (row)

5.3 IRCC-HC bicluster

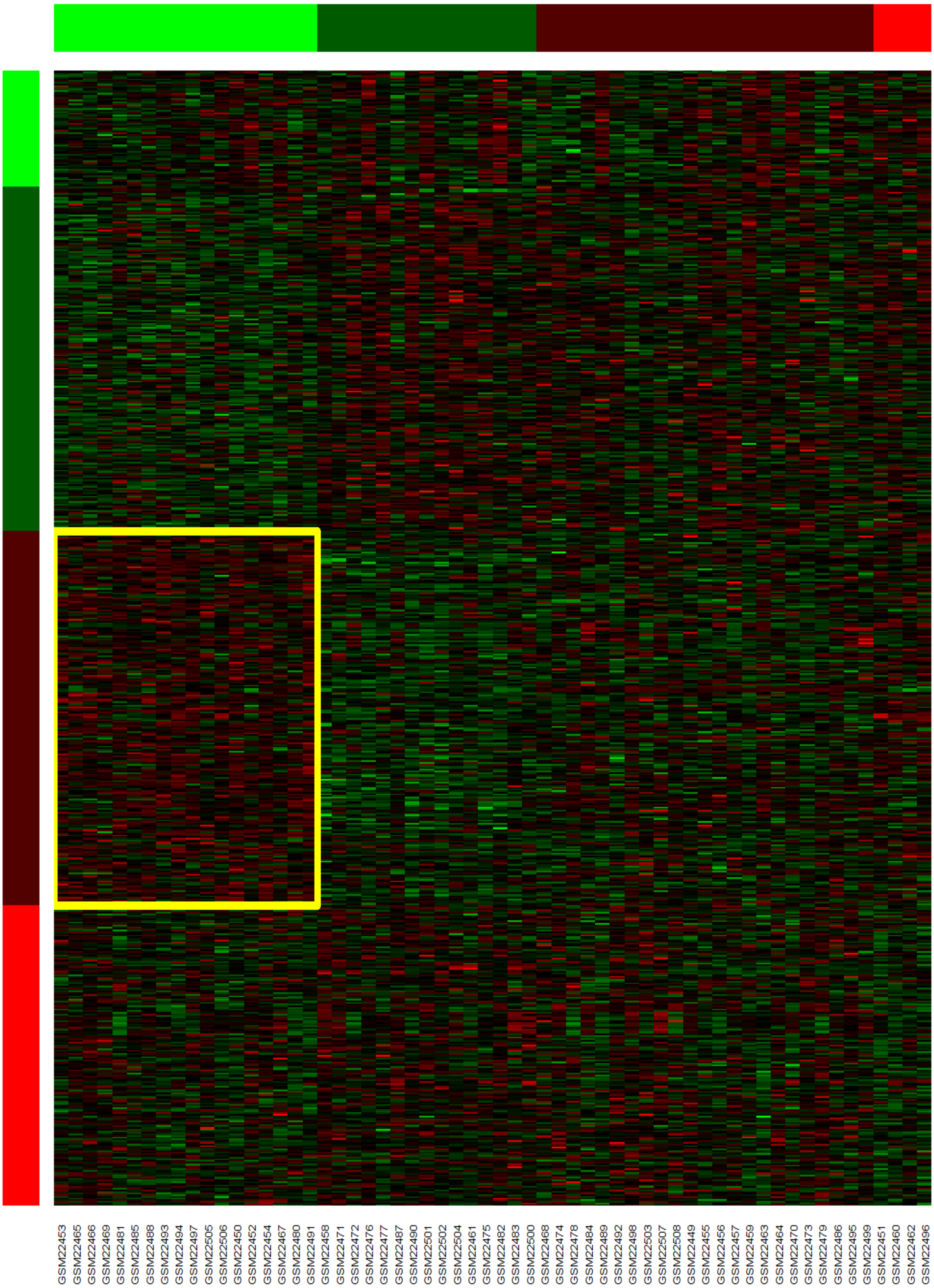

Figure 32: Heatmap of the IRCC-HC bicluster

IRCC-HC genes

ADAM12	ADAM23	ADAM30	ADAM33	ADAMTS1	ADAMTS12
ADAMTS2	ADAMTS3	ADAMTS4	ADAMTS6	ADAMTS8	ADAMTS9
ADAMTSL1	ADIPOQ	AGT	APP	APP	ASAM
BCAN	BGN	BST1	BST2	CD36	CD36
CD47	CD47	CDH11	CDH17	CDH18	CDH22
CDH3	CDH5	CDH6	CEACAM1	ChGn	CHL1
CHST1	CHST3	CHST6	CHST7	CHST9	CHSY1
CIB2	CNTN1	CNTN6	CNTNAP3	COL10A1	COL11A1
COL11A2	COL12A1	COL14A1	COL15A1	COL17A1	COL18A1
COL1A1	COL1A2	COL21A1	COL23A1	COL4A1	COL4A2
COL5A1	COL5A2	COL5A3	COL6A1	COL6A2	COL6A3
COL7A1	COL8A1	COLQ	COMP	CORIN	CPA3
CPE	CSGlcA_T	CTHRC1	CTSG	CTSK	CTSO
DCN	DCN	DKFZP586H212	EFEMP2	ELN	EMILIN1
EMILIN1	EMILIN2	ESAM	FBLN1	FBLN2	FBLN5
FBN1	FLRT2	GPC3	GPC6	HABP4	HAS1
HAS2	HSPG2	HTRA1	ITFG2	ITGA10	ITGA11
ITGA5	ITGA7	ITGA8	ITGA9	ITGAV	ITGAX
ITGB6	ITGBL1	ITGBL1	KLK1	KLK3	KLK5
KLK6	KLK7	KLK7	LAMA2	LAMA4	LAMB3
LAMC1	LAMC2	LEPRE1	MASP1	MATN2	MATN2
MATN3	MCAM	MME	MMP10	MMP11	MMP11
MMP13	MMP14	MMP16	MMP19	MMP2	MMP23B
MMP23B	MMP27	MMP28	MMP3	MMP7	NAALAD2
NAALADL1	NID2	NRP1	NRXN2	P11	PAPLN
PCDH12	PCDH17	PCDH17	PCDH18	PCDH19	PCDH7
PCDH9	PCDHB13	PCDHB14	PCDHB15	PCDHB16	PCDHB17
PCDHB18	PCDHB2	PCDHB4	PCDHB5	PCDHB6	PCDHB7
PCDHB8	PCDHGA12	PCDHGA12	PCDHGA3	PCOLCE	PCOLCE2
PECAM1	PGCP	PLXNA1	PLXNA2	PLXNA4A	PLXNC1
ROBO1	ROBO3	ROBO4	SDC2	SELE	SELP
SEMA3C	SERPINA2	SERPINB1	SERPINE1	SERPINE2	SERPINF1
SERPING1	SERPINH1	SGCB	SGCD	SGCG	SLIT2
SLIT2	SLIT3	SLIT3	SMOC2	SNED1	SPARC
SPARCL1	SPG21	SPG3A	SPOCK1	SPON1	SPON2
SPON2	THBS1	THBS2	THBS3	THBS4	TIMP2
TIMP3	TIMP4	TMPRSS3	TNC	TNN	TSPAN18
TSPAN2	TSPAN7	TSPAN8	VCAN	VWF	XPNPEP2

IRCC-HC samples

GSM22453 GSM22465 GSM22466 GSM22469 GSM22481 GSM22485 GSM22488 GSM22493 GSM22494 GSM22497 GSM22505 GSM22506 GSM22450 GSM22452 GSM22454 GSM22467 GSM22480 GSM22491

5.3.1 Comparing LAS and IRCC-HC biclusters

	No ECM	ECM3
No ECM3	450	13
ECM3	130	98
Jaccard similarity	0.41	

Table 25: Comparing gene lists: LAS (column) vs. IRCC-HC bicluster (row)

	No ECM	ECM3
No ECM3	40	2
ECM3	2	16
Jaccard similarity	0.80	

Table 26: Comparing sample lists: LAS (column) vs. IRCC-HC bicluster (row)

5.4 CCSS bicluster

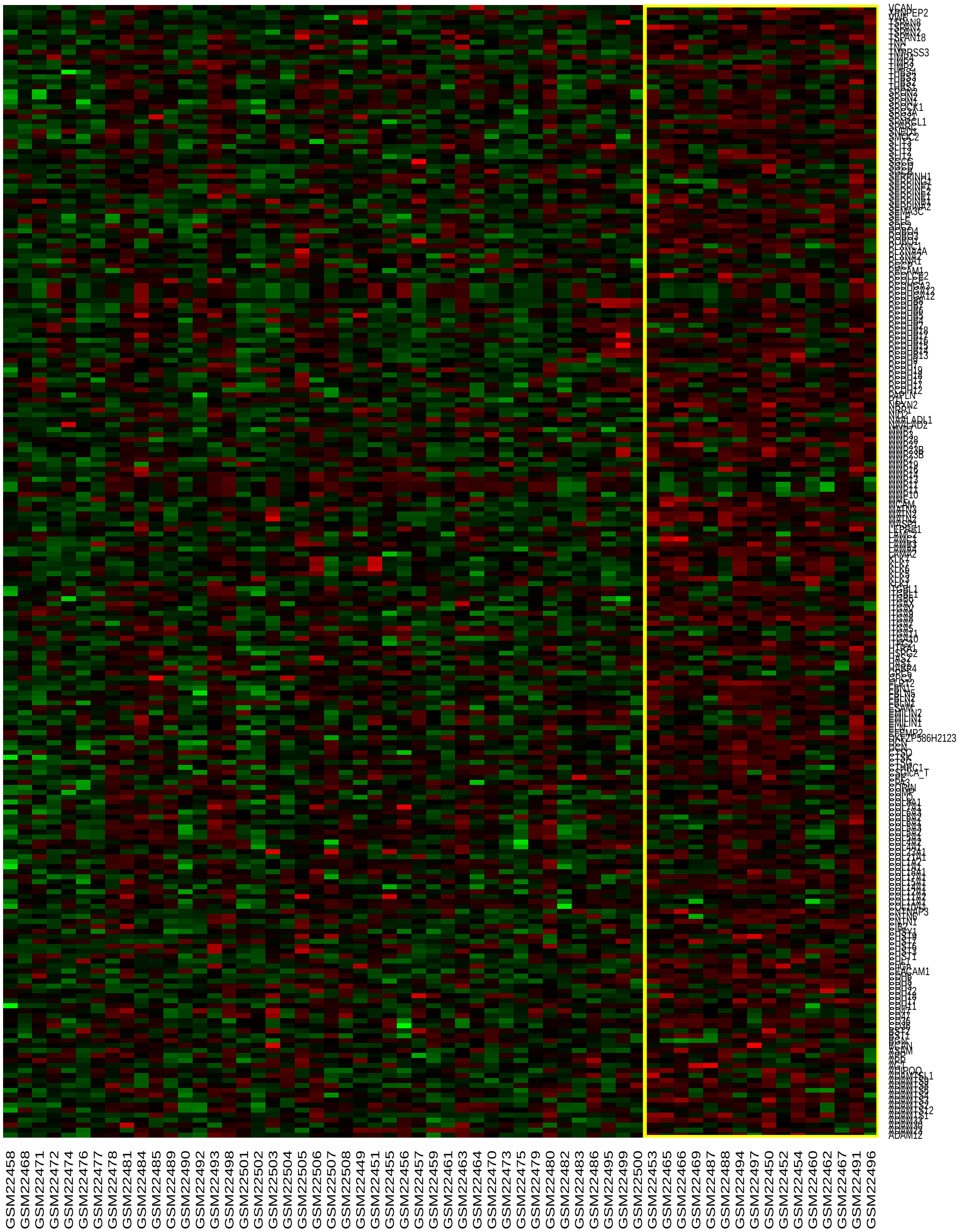

Figure 33: Heatmap of the CCSS bicluster

5.4.1 Comparing LAS and CCSS biclusters

	No ECM	ECM3
No ECM3	38	6
ECM3	4	12
Jaccard similarity	0.55	

Table 27: Comparing sample lists: LAS (column) vs. CCSS (row) biclusters

5.4.2 Prediction strength for CCSS

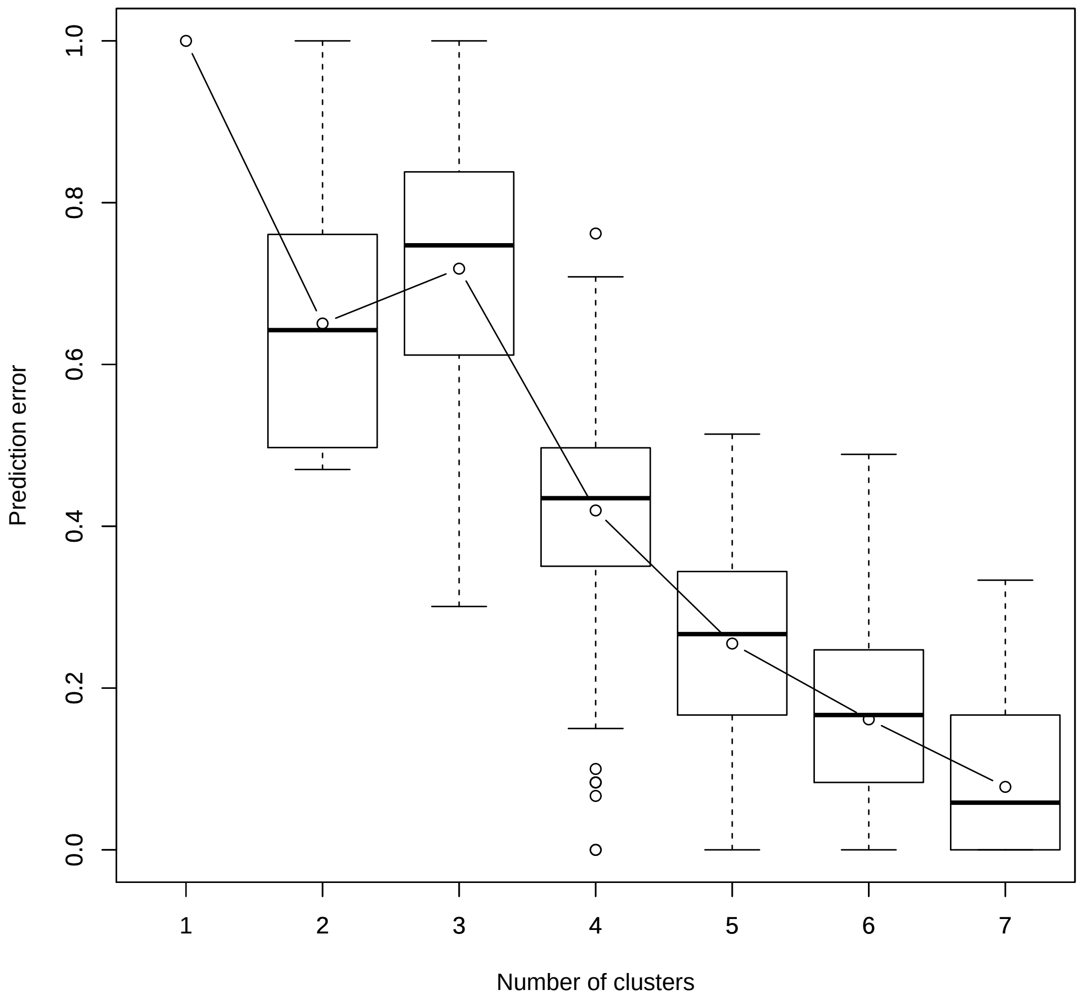

5.4.3 Consensus clustering

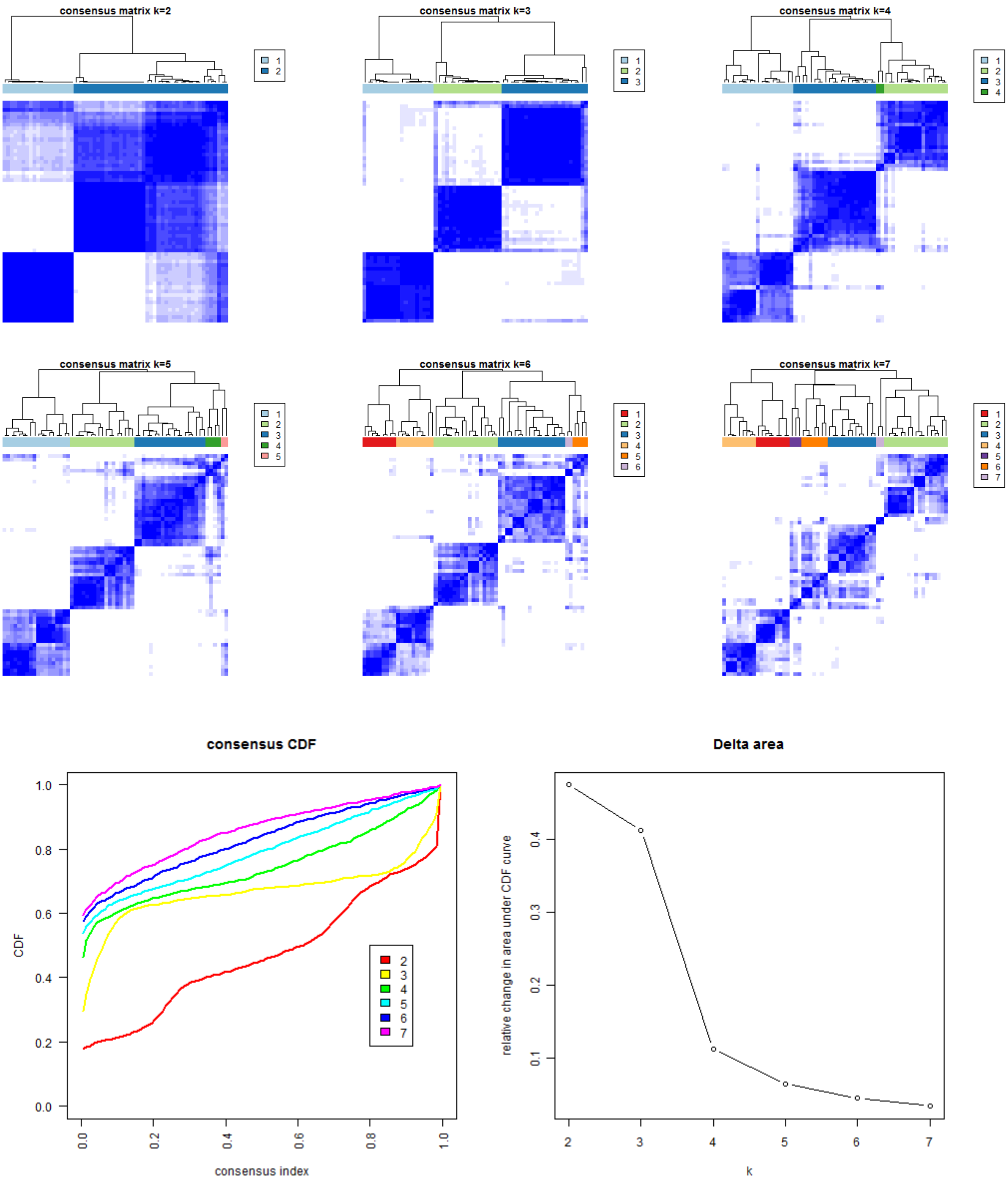

Figure 34: Statistical significance of CCSS clustering (Consensus clustering)

5.4.4 Statistical significance

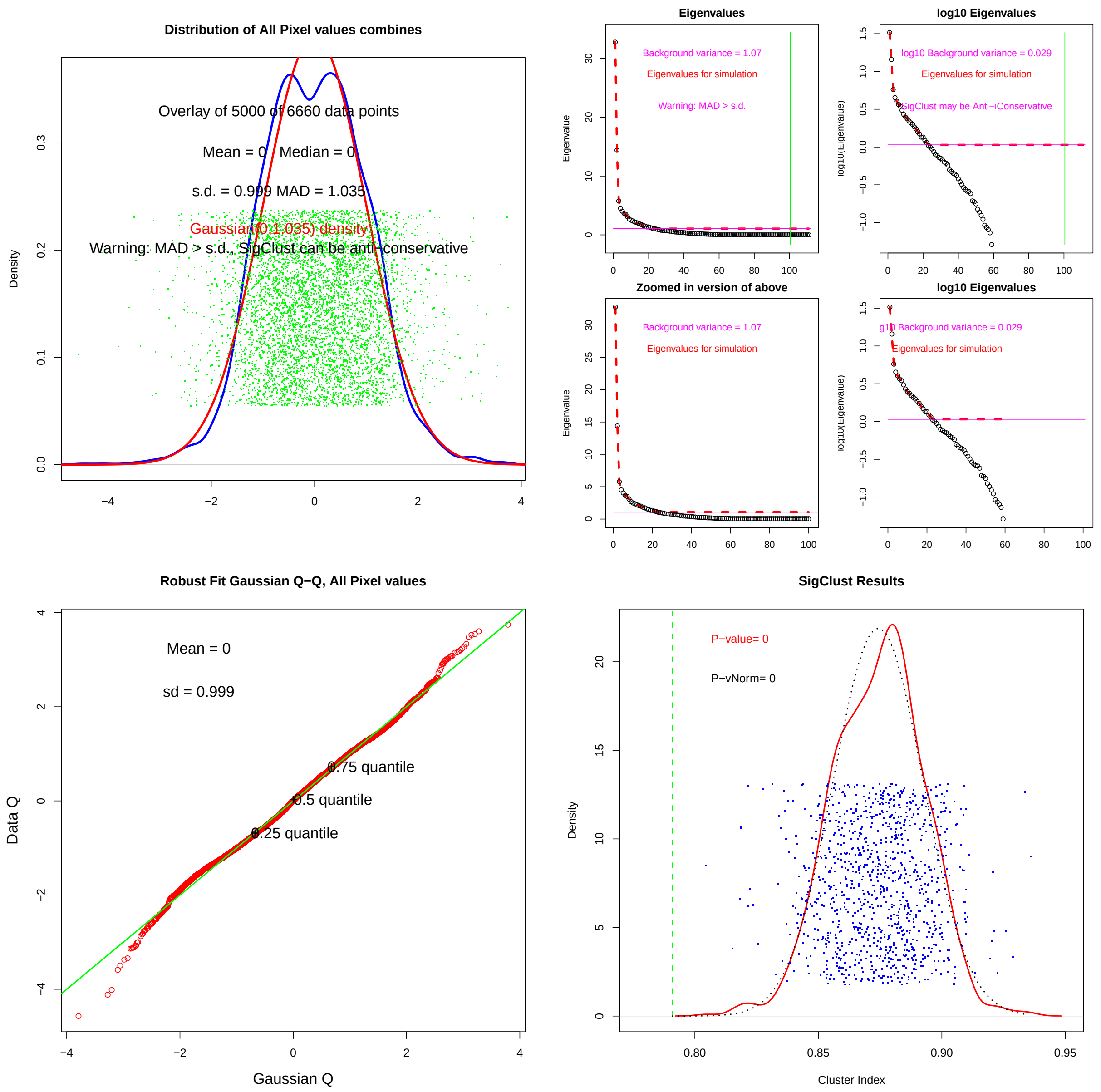

P-value	P-vNorm
0.0E+00	2.5E-06

Table 28: SigClust p-values

5.4.5 Cluster-wise assessment of cluster stability

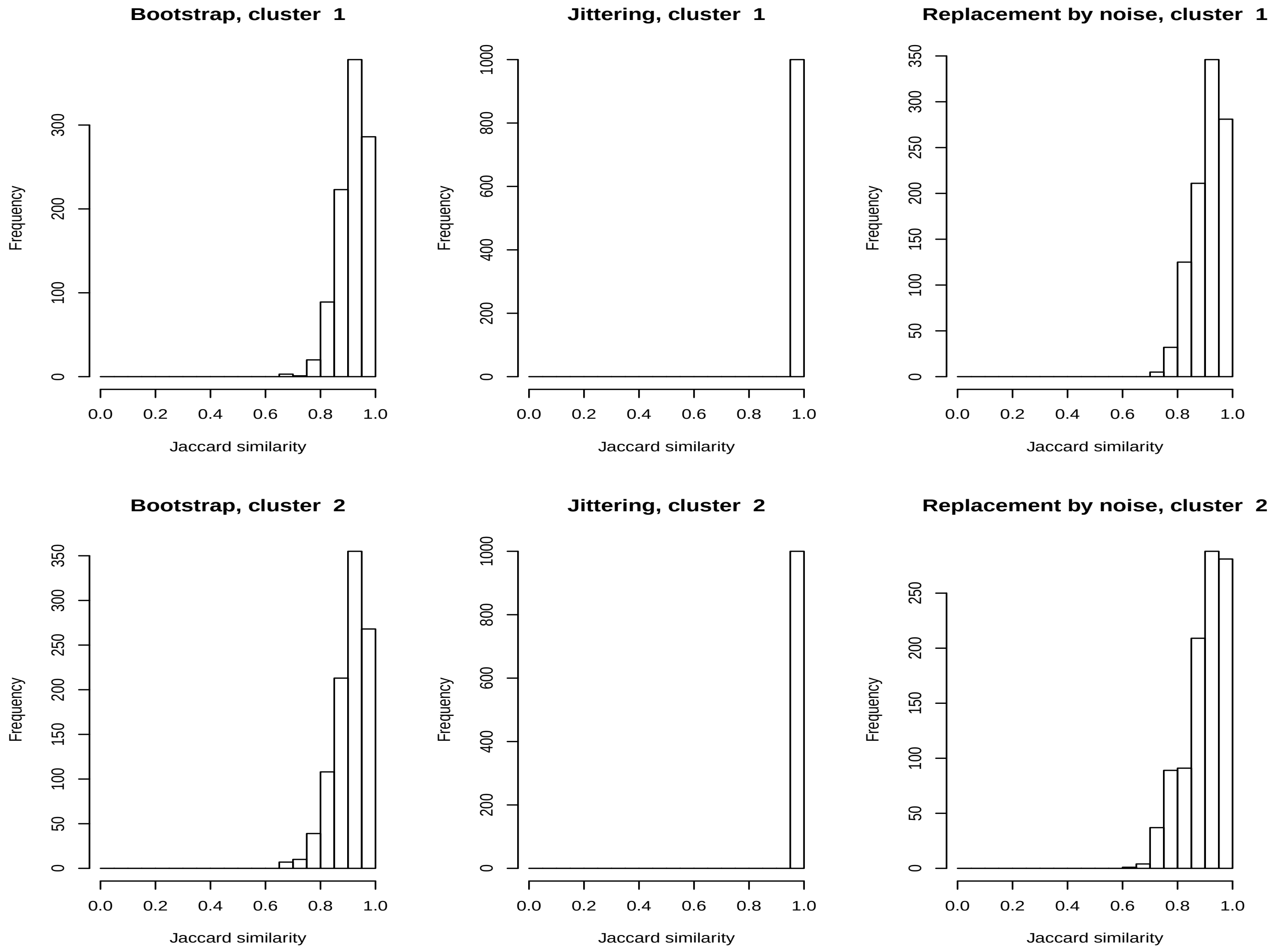


```
* Cluster stability assessment *
Cluster method:  kmeans
Full clustering results are given as parameter result
of the clusterboot object, which also provides further statistics
of the resampling results.
Number of resampling runs:  1000

Number of clusters found in data:  2

 Clusterwise Jaccard bootstrap mean:
[1] 0.9199726 0.9118352
dissolved:
[1] 0 0
recovered:
[1] 996 983
 Clusterwise Jaccard jittering mean:
[1] 0.9981930 0.9981273
dissolved:
[1] 0 0
recovered:
```

```
[1] 1000 1000
 Clusterwise Jaccard replacement by noise mean:
[1] 0.9133471 0.8996397
dissolved:
[1] 0 0
recovered:
[1] 995 958
```

Removing one sample

```
   Min. 1st Qu.  Median    Mean 3rd Qu.    Max.
 0.9048  1.0000  1.0000  0.9921  1.0000  1.0000
```

Removing one gene

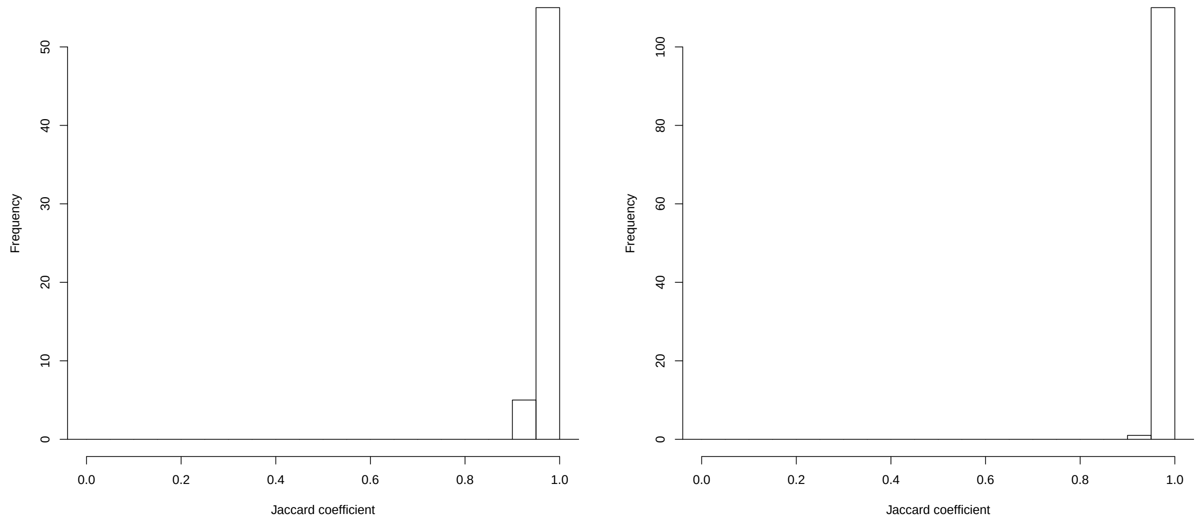

Figure 35: Removing one sample (left) and one gene (right) at a time: distributions of Jaccard coefficients

```
   Min. 1st Qu.  Median    Mean 3rd Qu.    Max.
 0.9091  1.0000  1.0000  0.9992  1.0000  1.0000
```

```
      APN                 AD              ADM               FOM
 Min.   :0.06349   Min.   :13.00   Min.   :0.8571   Min.   :0.6482
 1st Qu.:0.06349   1st Qu.:13.00   1st Qu.:0.8571   1st Qu.:0.8715
 Median :0.06349   Median :13.00   Median :0.8571   Median :0.9158
 Mean   :0.06401   Mean   :13.00   Mean   :0.8625   Mean   :0.9005
 3rd Qu.:0.06349   3rd Qu.:13.00   3rd Qu.:0.8571   3rd Qu.:0.9404
 Max.   :0.12063   Max.   :13.13   Max.   :1.4569   Max.   :0.9916
```

Removing sets of k genes

```
   Min. 1st Qu.  Median    Mean 3rd Qu.    Max.
 0.4762  0.7692  0.8333  0.8170  0.9091  1.0000
```

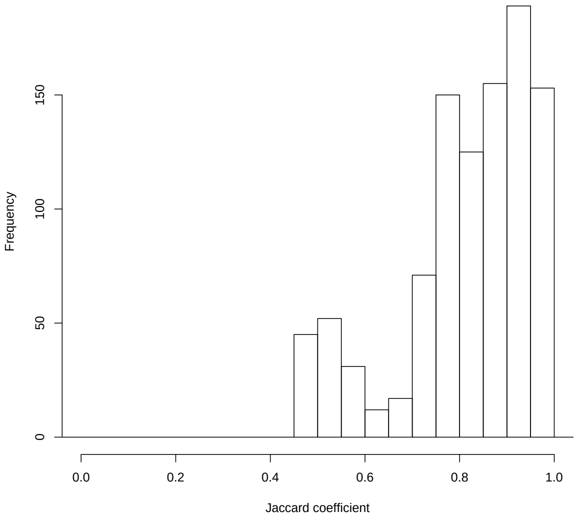

Figure 36: Removing two thirds of the genes: distribution of Jaccard coefficients

5.4.6 Validation measures

```
Clustering Methods:
 kmeans

Cluster sizes:
 2 3 4 5 6

Validation Measures:
                          2       3       4       5       6

kmeans APN           0.0638  0.0006  0.1486  0.1582  0.1054
       AD           13.0425 11.9173 11.8692 11.5951 11.1700
       ADM           0.9246  0.0079  1.6336  1.6651  1.2240
       FOM           0.9065  0.8428  0.8386  0.8315  0.8225
       Connectivity  9.0968 17.1563 36.6698 39.4595 55.3802
       Dunn          0.5243  0.6137  0.5997  0.5521  0.5964
       Silhouette    0.1796  0.1473  0.1246  0.1068  0.0851

Optimal Scores:

             Score   Method Clusters
APN           0.0006 kmeans 3
AD           11.1700 kmeans 6
ADM           0.0079 kmeans 3
FOM           0.8225 kmeans 6
Connectivity  9.0968 kmeans 2
Dunn          0.6137 kmeans 3
Silhouette    0.1796 kmeans 2
```

6 Desmedt et al (2007) dataset

6.1 LAS bicluster

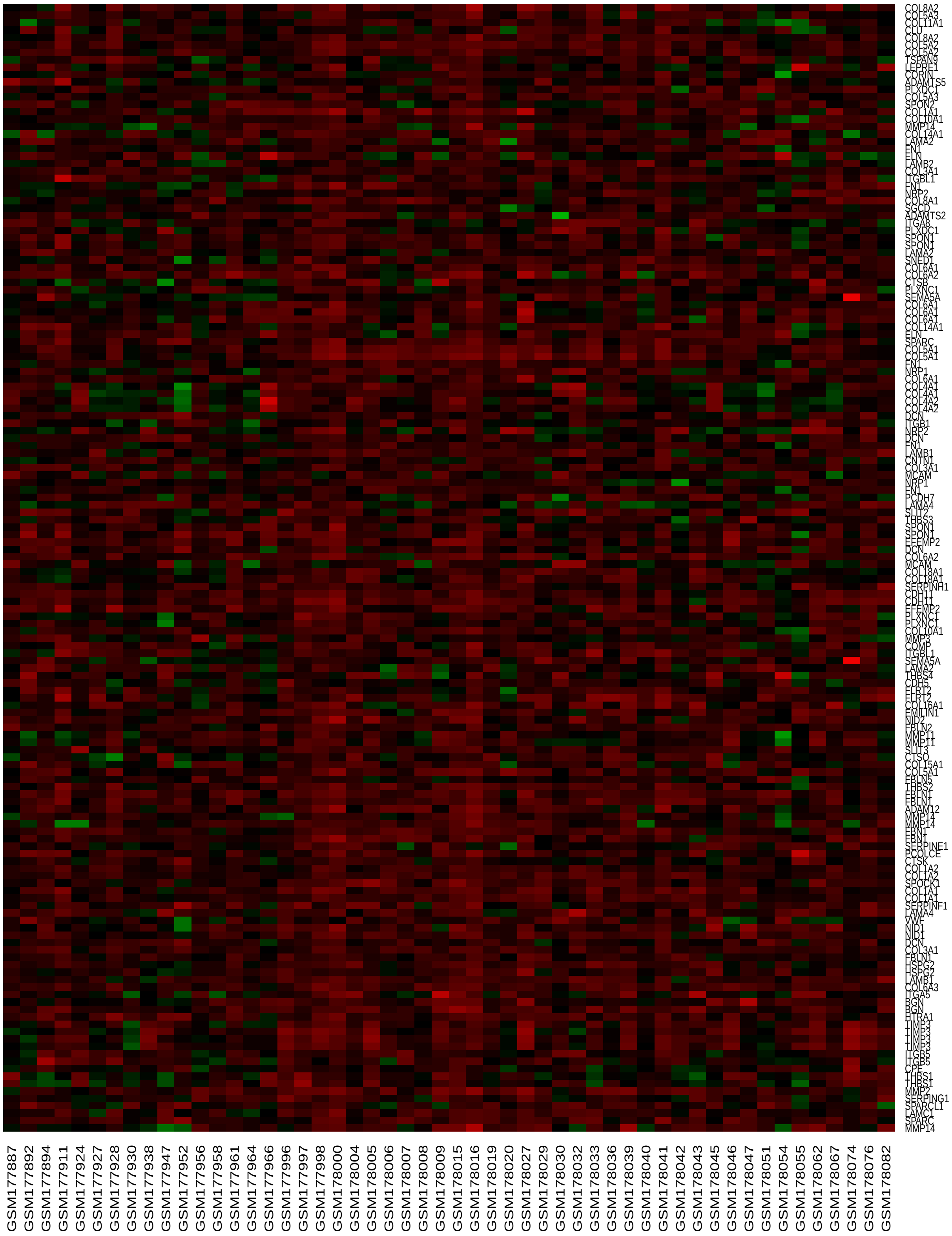

Figure 37: Heatmap of the LAS bicluster

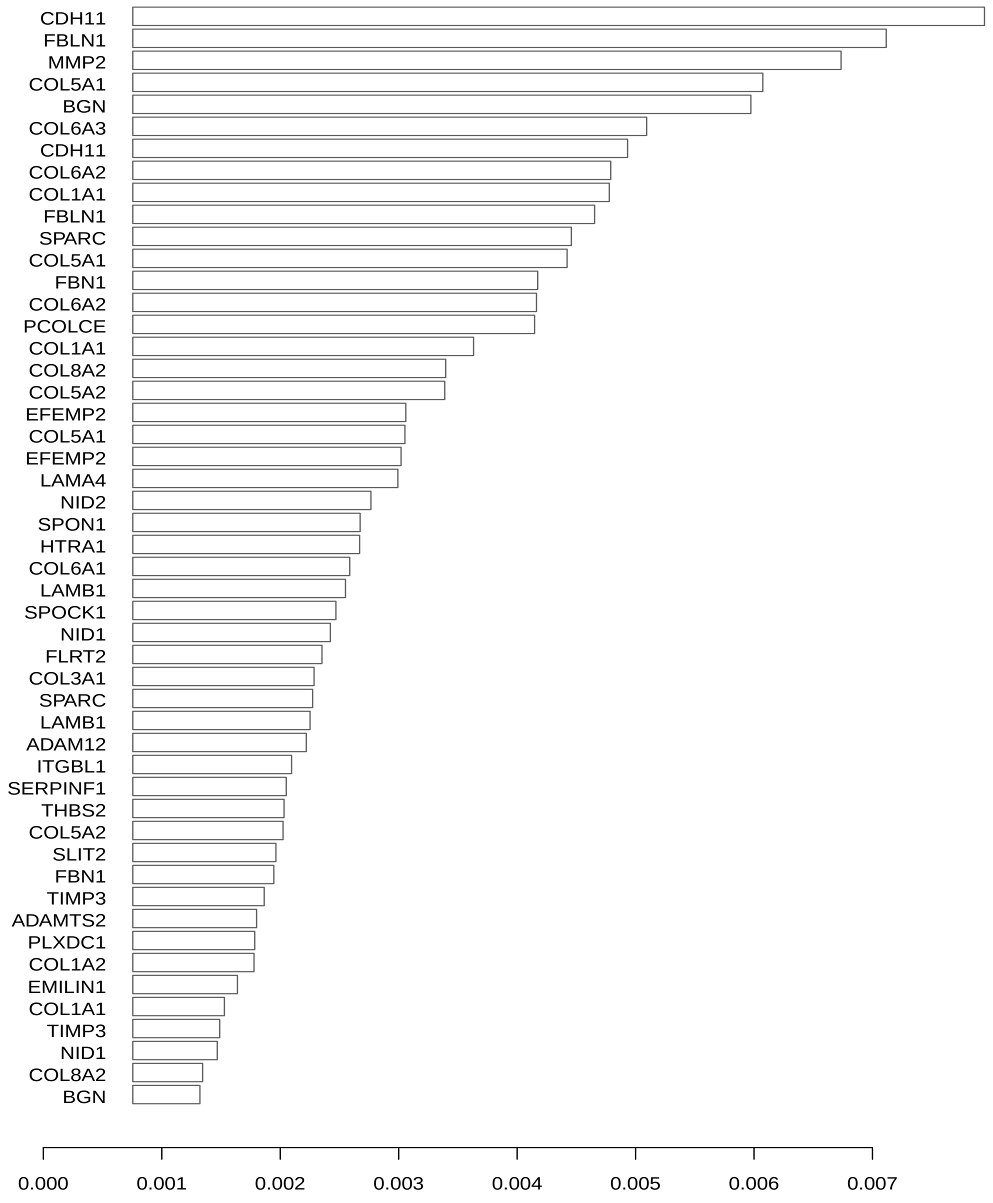

Figure 38: Most informative genes for the LAS bicluster

```
Number of informative genes of the LAS bicluster that also belong
to the ECM3 consensus list: 32 of 34 (94%)
```

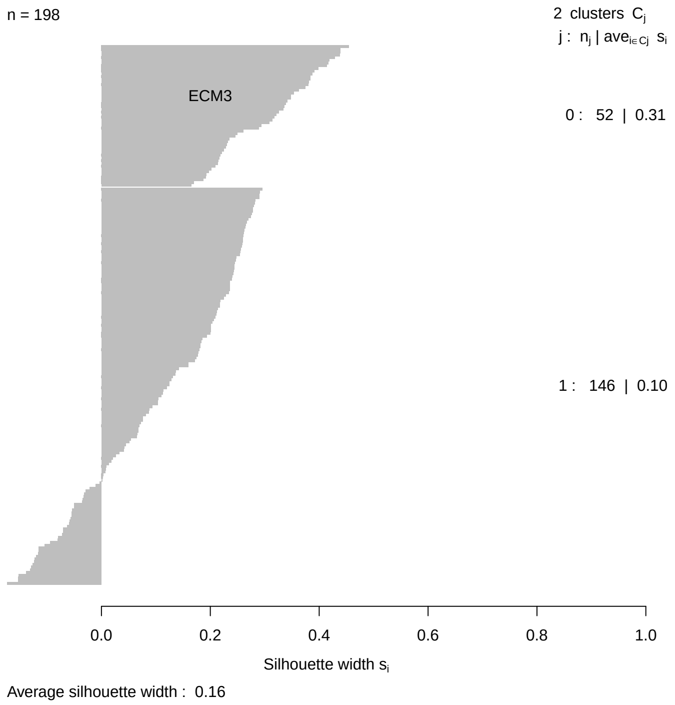

Figure 39: Silhoutte plot for the LAS bicluster

Connectivity	Dunn Index
53.52	0.35

Table 29: Connectivity validation measure and Dunn Index of LAS partitioning

6.2 IRCC-KM bicluster

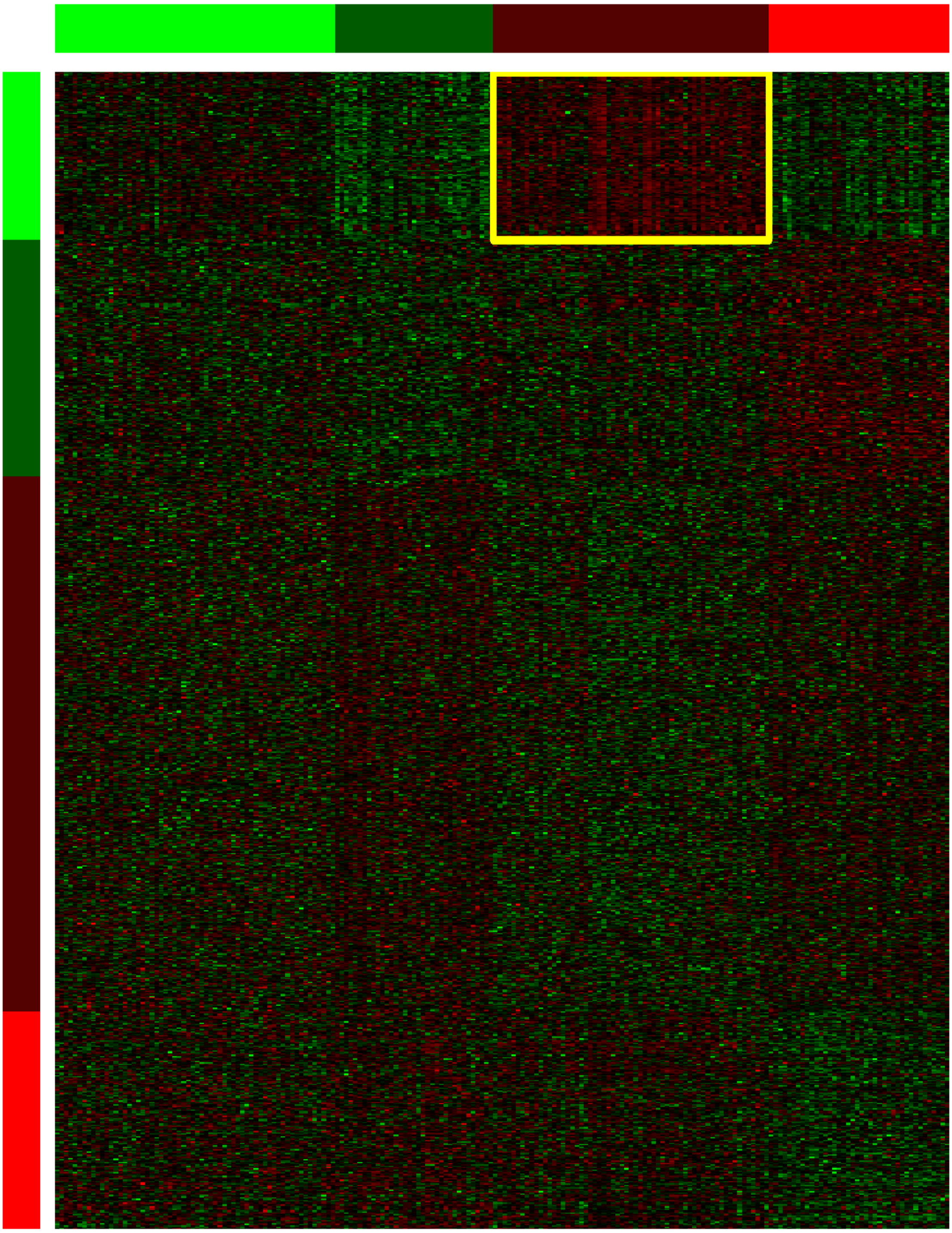

Figure 40: Heatmap of the IRCC-KM bicluster

IRCC-KM genes

MMP14	SPARC	LAMC1	LAMC1	MMP2	THBS1	THBS1
THBS1	TIMP3	TIMP3	TIMP3	TIMP3	HTRA1	BGN
BGN	ITGA5	COL6A3	LAMB1	TNC	HSPG2	HSPG2
FBLN1	COL3A1	DCN	NID1	NID1	LAMA4	SERPINF1
COL1A1	COL1A1	SPOCK1	COL1A2	COL1A2	CTSK	PCOLCE
SERPINE1	GPC1	FBN1	FBN1	MMP14	MMP14	ADAM12
FBLN1	FBLN1	THBS2	COL5A1	COL15A1	SLIT3	MMP11
MMP11	FBLN2	NID2	COL7A1	EMILIN1	COL11A1	COL16A1
FLRT2	FLRT2	CDH13	SEMA5A	ITGBL1	PCDH7	PCDH7
COMP	MMP3	COL10A1	MMP13	EFEMP2	CDH11	CDH11
SERPINH1	COL18A1	COL18A1	COL6A2	DCN	EFEMP2	SPON1
SPON1	THBS3	SLIT2	PCDH7	FN1	NRP1	COL3A1
CNTN1	LAMB1	FN1	DCN	NRP2	ITGB1	DCN
COL6A1	NRP1	FN1	COL5A1	COL5A1	SPARC	ELN
COL6A1	COL6A1	COL6A1	PLXNC1	COL6A2	COL6A1	SNED1
SPON1	SPON1	PLXDC1	ITGA8	ADAMTS2	SGCD	COL8A1
NRP2	FN1	FN1	ITGBL1	COL3A1	FN1	COL10A1
COL1A1	SPON2	COL5A3	PLXDC1	ADAMTS5	CORIN	LEPRE1
COL5A2	COL5A2	COL8A2	COL11A1	COL5A3	COL8A2	

IRCC-KM samples

GSM177887	GSM177892	GSM177894	GSM177911	GSM177921	GSM177924	GSM177927
GSM177928	GSM177930	GSM177938	GSM177944	GSM177947	GSM177958	GSM177961
GSM177964	GSM177966	GSM177976	GSM177989	GSM177992	GSM177993	GSM177994
GSM177996	GSM177997	GSM177998	GSM178000	GSM178002	GSM178004	GSM178005
GSM178006	GSM178007	GSM178008	GSM178009	GSM178014	GSM178015	GSM178016
GSM178019	GSM178027	GSM178028	GSM178029	GSM178030	GSM178032	GSM178033
GSM178035	GSM178036	GSM178039	GSM178040	GSM178041	GSM178042	GSM178043
GSM178045	GSM178046	GSM178047	GSM178051	GSM178054	GSM178055	GSM178062
GSM178067	GSM178073	GSM178074	GSM178076	GSM178082		

6.2.1 Comparing LAS and IRCC-KM biclusters

	No ECM	ECM3
No ECM3	744	31
ECM3	11	121
Jaccard similarity	0.74	

Table 30: Comparing gene lists: LAS (column) vs. IRCC-KM bicluster (row)

	No ECM	ECM3
No ECM3	134	3
ECM3	12	49
Jaccard similarity	0.77	

Table 31: Comparing sample lists: LAS (column) vs. IRCC-KM bicluster (row)

6.3 IRCC-HC bicluster

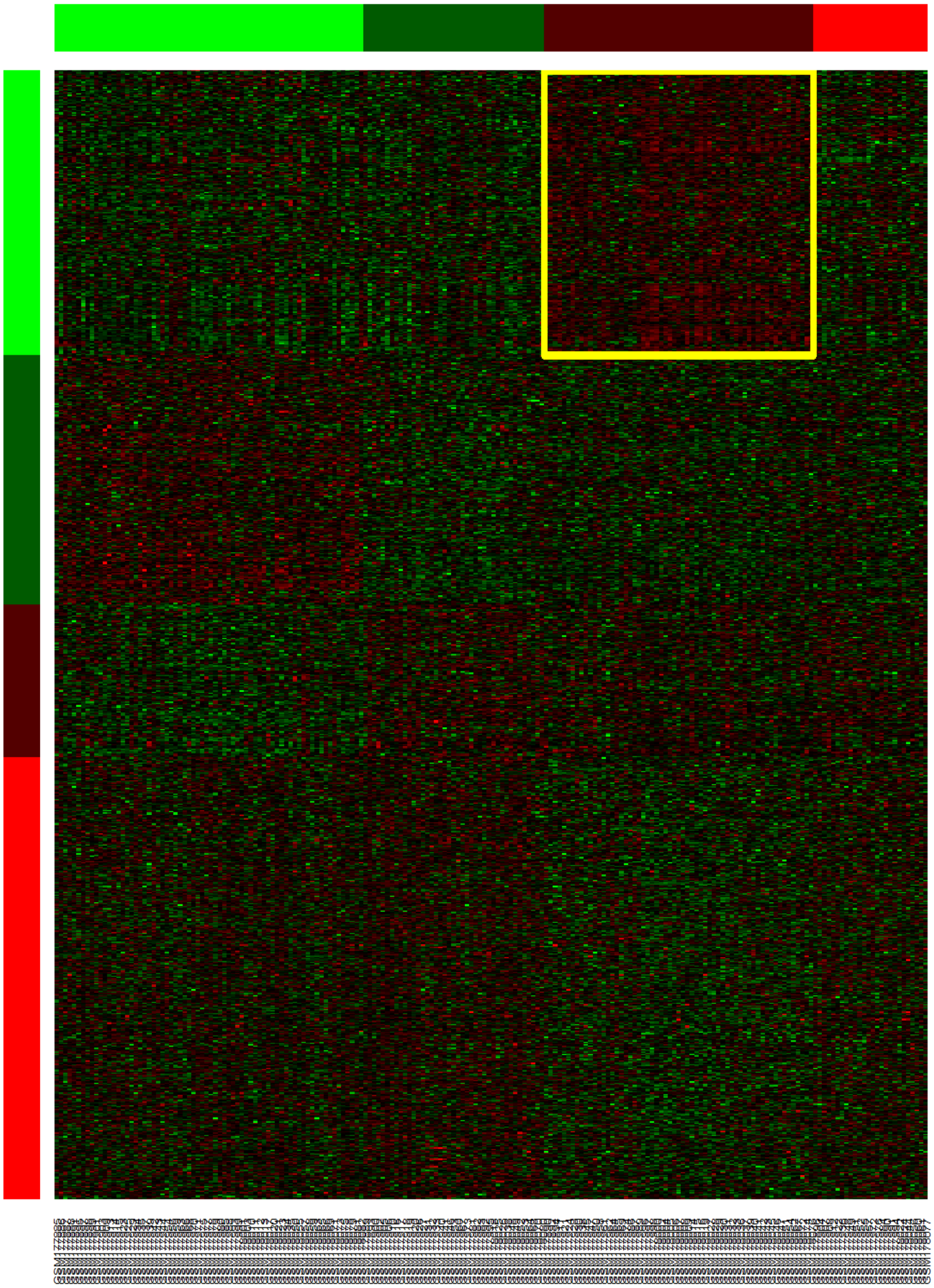

Figure 41: Heatmap of the IRCC-HC bicluster

IRCC-HC genes

ADAM12	ADAM8	ADAMTS1	ADAMTS2	ADAMTS2	ADAMTS5
ADIPOQ	BGN	BGN	CD36	CD36	CDH11
CDH11	CDH13	CDH2	CDH2	CDH5	CHL1
CHPF	CLU	CLU	CLU	CNTN1	CNTNAP1
COL10A1	COL10A1	COL11A1	COL11A1	COL13A1	COL13A1
COL13A1	COL14A1	COL14A1	COL14A1	COL15A1	COL16A1
COL17A1	COL18A1	COL18A1	COL1A1	COL1A1	COL1A1
COL1A2	COL1A2	COL3A1	COL3A1	COL3A1	COL4A1
COL4A1	COL4A2	COL4A2	COL5A1	COL5A1	COL5A1
COL5A2	COL5A2	COL5A3	COL5A3	COL6A1	COL6A1
COL6A1	COL6A1	COL6A1	COL6A2	COL6A2	COL6A3
COL7A1	COL7A1	COL8A1	COL8A2	COL8A2	COMP
CORIN	CPA3	CSGlcA_T	CSGlcA_T	CSPG4	CSPG4
CTSG	CTSK	DCN	DCN	DCN	DCN
DKFZP586H212	DPP4	DPP4	DPP4	EFEMP1	EFEMP1
EFEMP2	EFEMP2	ELN	ELN	EMILIN1	FBLN1
FBLN1	FBLN1	FBLN2	FBLN5	FBN1	FBN1
FLRT2	FLRT2	FN1	FN1	FN1	FN1
FN1	FN1	GPC1	GPC1	HSPG2	HSPG2
HTRA1	HYAL1	ITGA2	ITGA5	ITGA7	ITGA7
ITGB3	ITGB3	ITGB4	ITGB4	ITGB4	ITGBL1
ITGBL1	LAMA2	LAMA2	LAMA2	LAMA4	LAMA4
LAMA4	LAMA5	LAMB1	LAMB1	LAMC1	LAMC1
LEPRE1	MATN2	MCAM	MCAM	MCAM	MCAM
MME	MME	MMP11	MMP11	MMP13	MMP14
MMP14	MMP14	MMP14	MMP15	MMP17	MMP2
MMP3	NID1	NID1	NID2	NRP1	NRP1
NRP2	NRP2	NRXN2	PCDH12	PCDH17	PCDH7
PCDH7	PCDH7	PCOLCE	PCOLCE2	PLXDC1	PLXDC1
PLXNA1	PLXNA2	PLXNA3	PLXNB2	PLXNB3	PLXND1
PLXND1	PRSS22	SELE	SELP	SEMA3G	SEMA5A
SEMA5A	SERPINE1	SERPINE1	SERPINE2	SERPINF1	SERPING1
SERPINH1	SGCD	SGCD	SLIT2	SLIT3	SNED1
SNED1	SPARC	SPARC	SPARCL1	SPOCK1	SPON1
SPON1	SPON1	SPON1	SPON2	THBS1	THBS1
THBS1	THBS1	THBS2	THBS3	THBS4	TIMP1
TIMP3	TIMP3	TIMP3	TIMP3	TIMP4	TNN
TNXB	TNXB	TNXB	TSPAN7	TSPAN9	TSPAN9
VWF					

IRCC-HC samples

GSM177890 GSM177892 GSM177894 GSM177911 GSM177921 GSM177924 GSM177928
GSM177930 GSM177938 GSM177945 GSM177947 GSM177950 GSM177958 GSM177961
GSM177962 GSM177964 GSM177965 GSM177969 GSM177974 GSM177986 GSM177987
GSM177989 GSM177996 GSM177997 GSM177998 GSM178000 GSM178002 GSM178004
GSM178005 GSM178006 GSM178007 GSM178008 GSM178009 GSM178014 GSM178015

GSM178016 GSM178019 GSM178027 GSM178028 GSM178029 GSM178030 GSM178031
GSM178032 GSM178033 GSM178036 GSM178037 GSM178039 GSM178040 GSM178041
GSM178042 GSM178043 GSM178045 GSM178046 GSM178047 GSM178051 GSM178054
GSM178062 GSM178067 GSM178072 GSM178074 GSM178076

6.3.1 Comparing LAS and IRCC-HC biclusters

	No ECM	ECM3
No ECM3	667	11
ECM3	88	141
Jaccard similarity	0.59	

Table 32: Comparing gene lists: LAS (column) vs. IRCC-HC bicluster (row)

	No ECM	ECM3
No ECM3	129	8
ECM3	17	44
Jaccard similarity	0.64	

Table 33: Comparing sample lists: LAS (column) vs. IRCC-HC bicluster (row)

6.4 CCSS bicluster

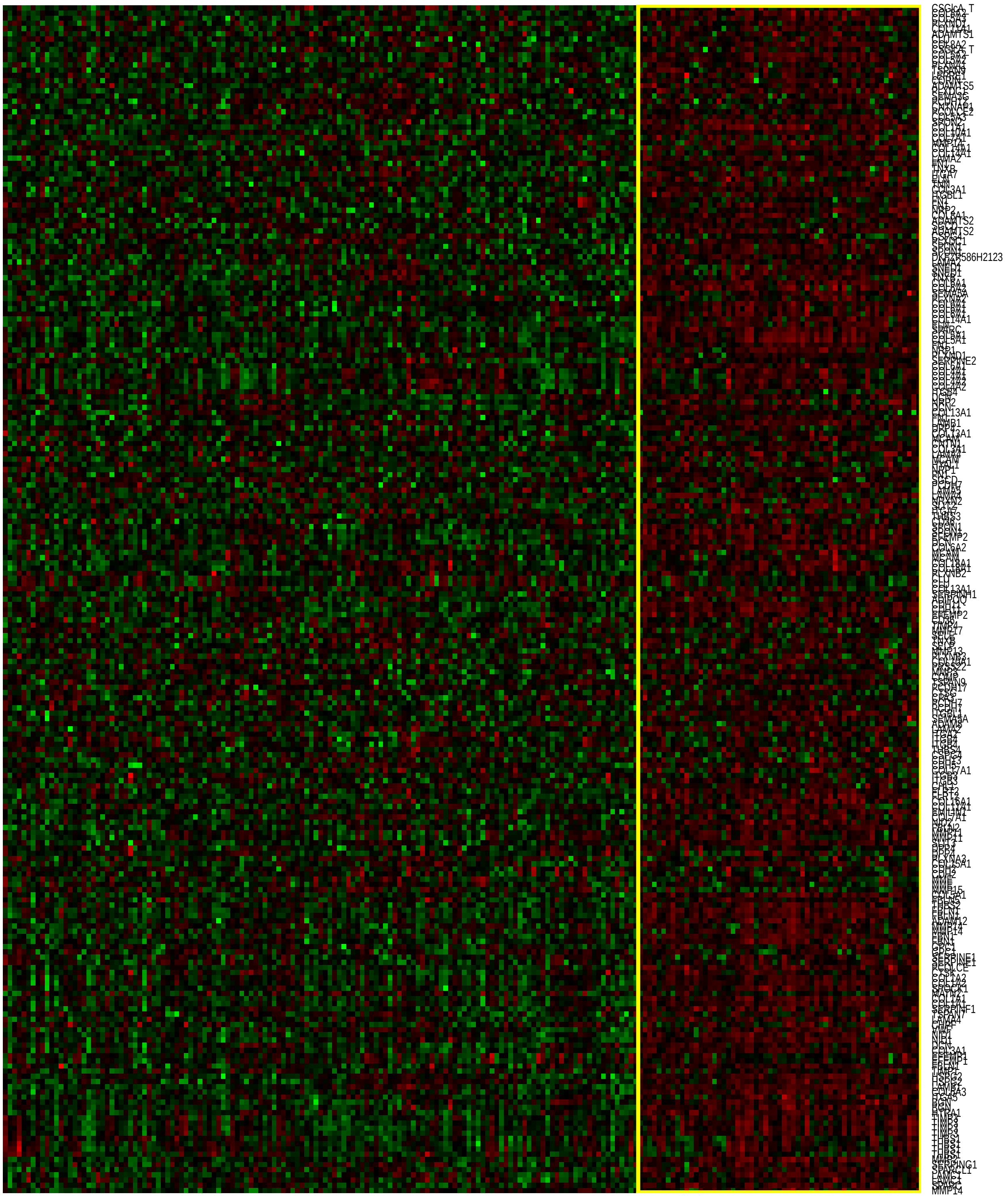

Figure 42: Heatmap of the CCSS bicluster

6.4.1 Comparing LAS and CCSS biclusters

	No ECM	ECM3
No ECM3	137	0
ECM3	9	52
Jaccard similarity	0.85	

Table 34: Comparing sample lists: LAS (column) vs. CCSS (row) biclusters

6.4.2 Prediction strength for CCSS

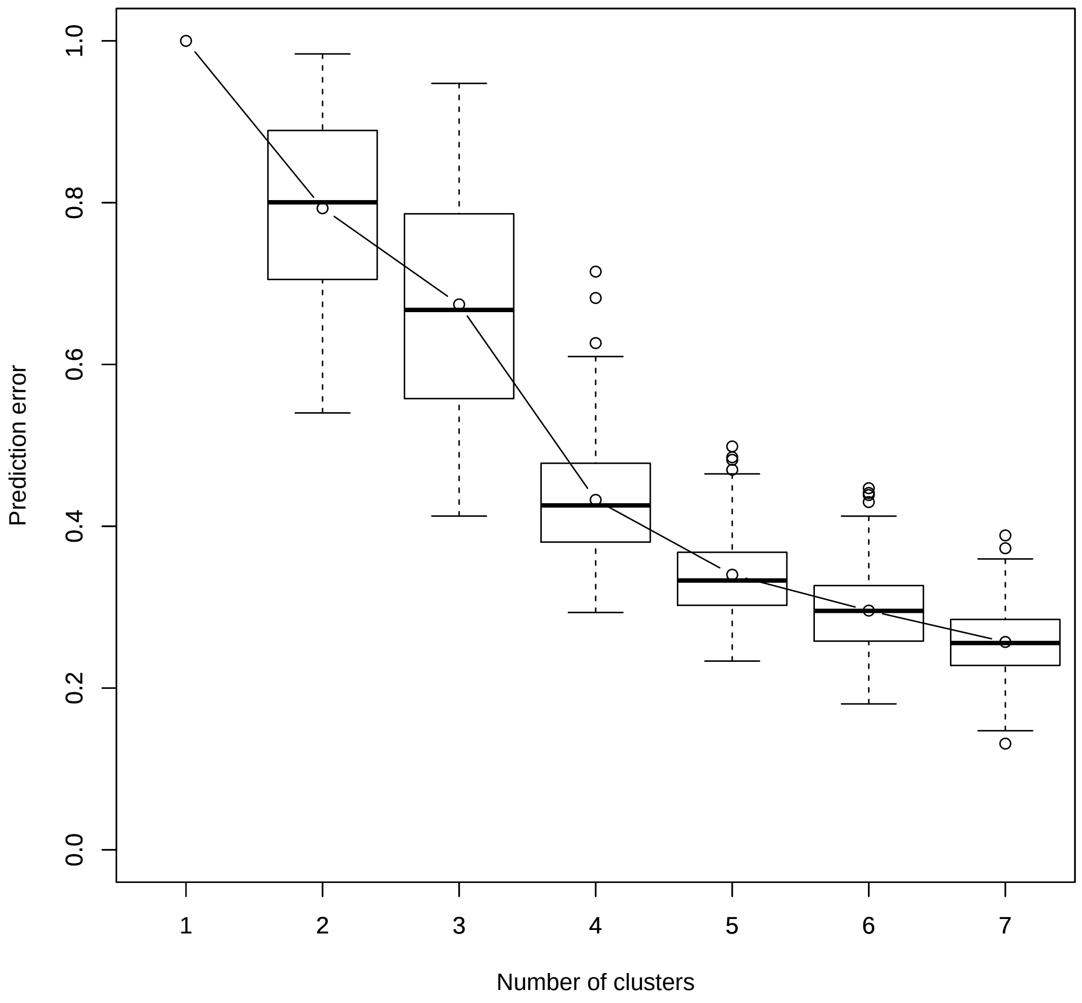

6.4.3 Consensus clustering

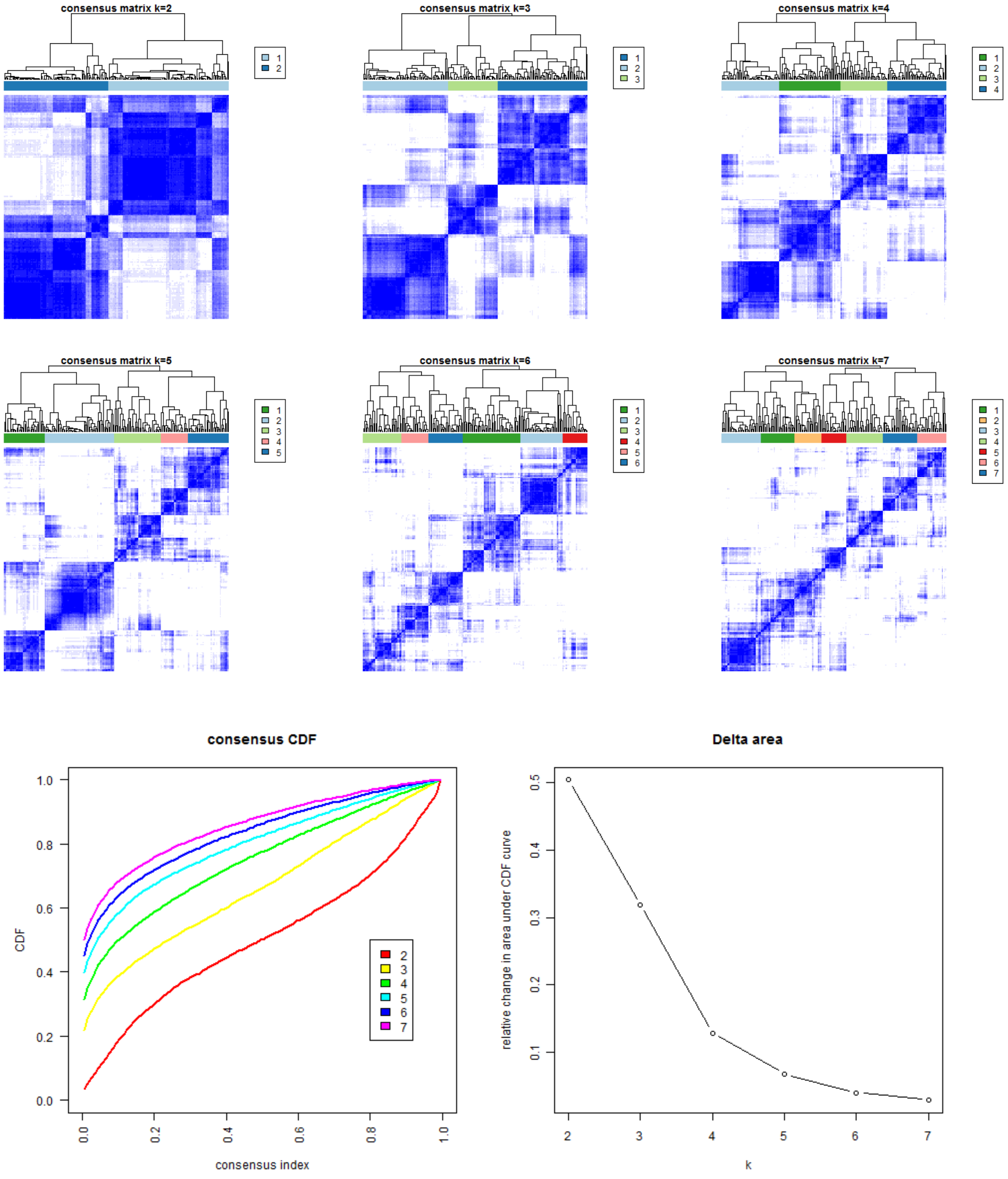

Figure 43: Statistical significance of CCSS clustering (Consensus clustering)

6.4.4 Statistical significance

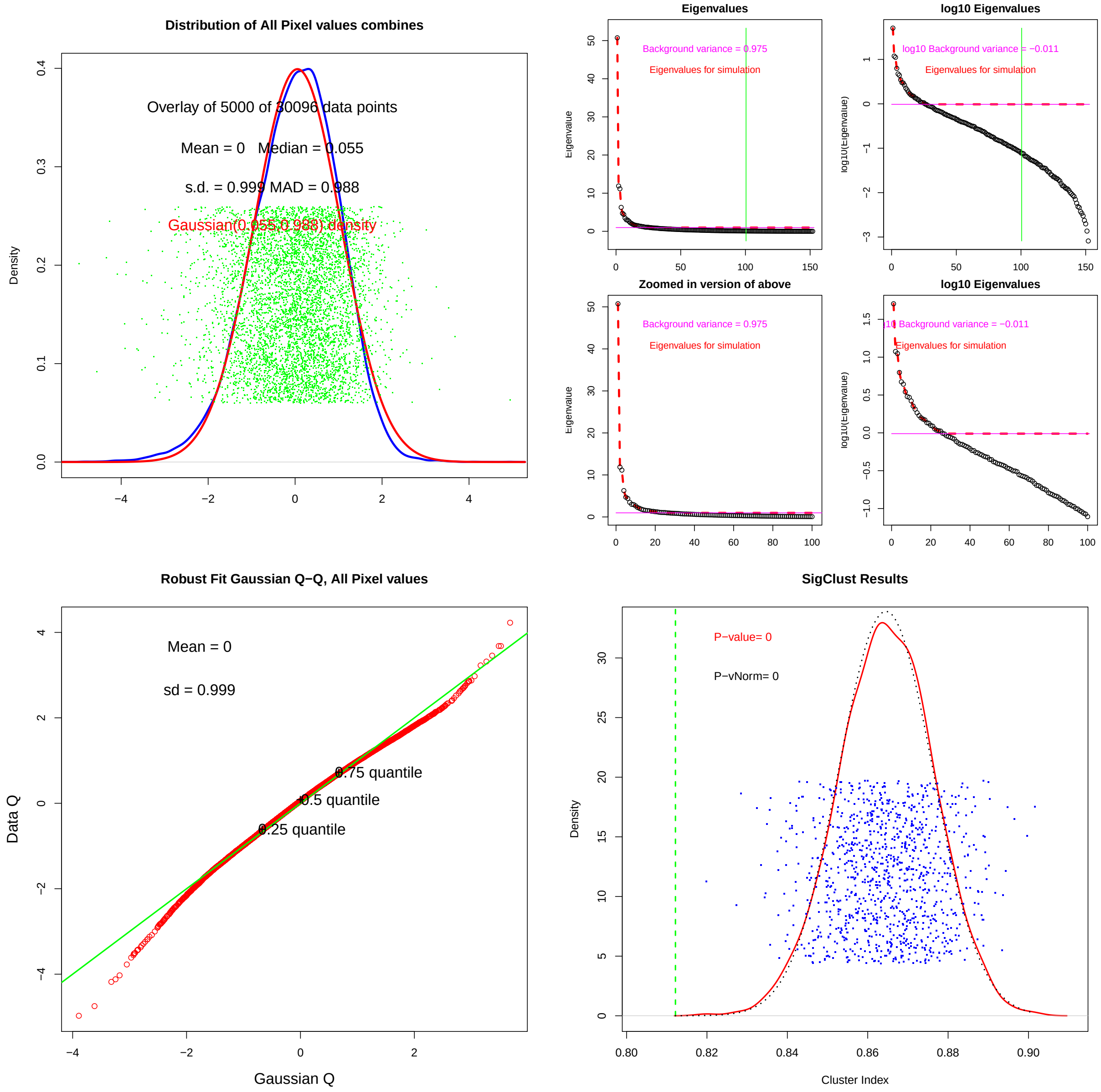

P-value	P-vNorm
0.0E+00	4.3E-06

Table 35: SigClust p-values

6.4.5 Cluster-wise assessment of cluster stability

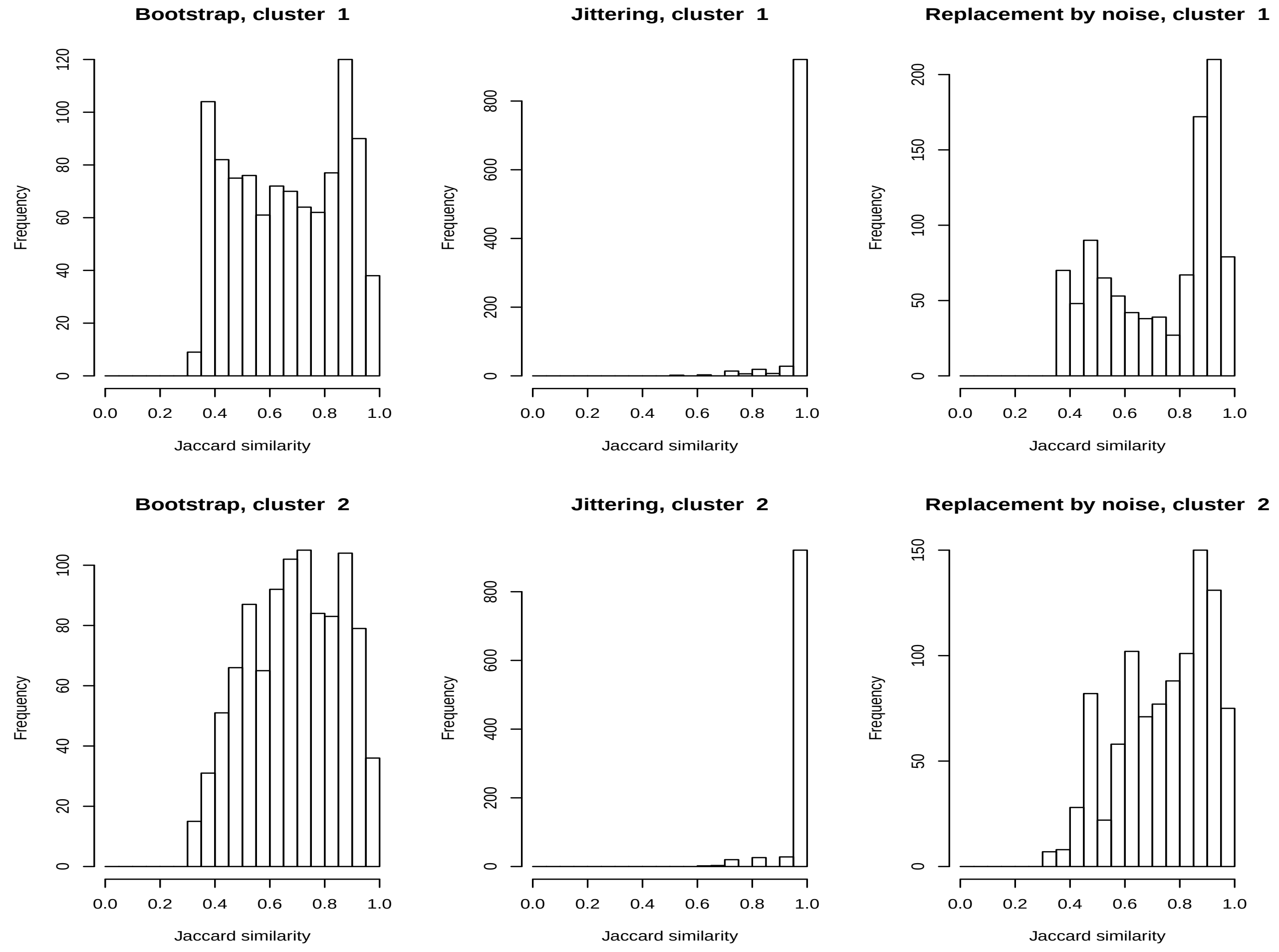


```
* Cluster stability assessment *
Cluster method:  kmeans
Full clustering results are given as parameter result
of the clusterboot object, which also provides further statistics
of the resampling results.
Number of resampling runs:  1000

Number of clusters found in data:  2

 Clusterwise Jaccard bootstrap mean:
[1] 0.6648033 0.6885693
dissolved:
[1] 270 163
recovered:
[1] 387 386
 Clusterwise Jaccard jittering mean:
[1] 0.9868980 0.9864249
dissolved:
[1] 0 0
recovered:
```

```
[1] 981 975
 Clusterwise Jaccard replacement by noise mean:
[1] 0.7334420 0.7464246
dissolved:
[1] 208 125
recovered:
[1] 555 545
```

Removing one sample

```
   Min. 1st Qu.  Median    Mean 3rd Qu.    Max. 
      1       1       1       1       1       1 
```

Removing one gene

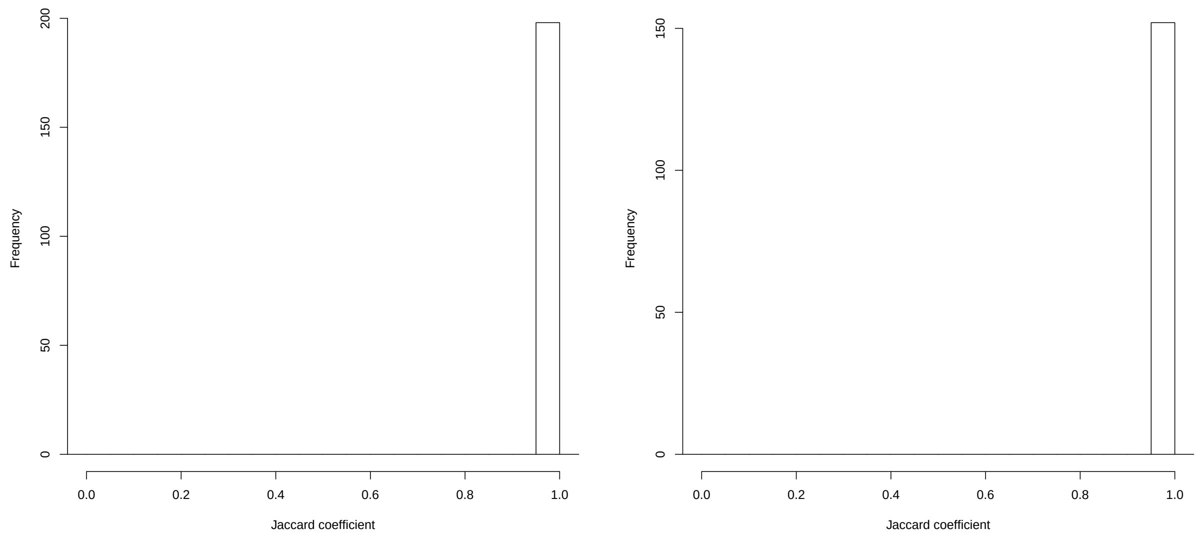

Figure 44: Removing one sample (left) and one gene (right) at a time: distributions of Jaccard coefficients

```
   Min. 1st Qu.  Median    Mean 3rd Qu.    Max. 
 0.9818  1.0000  1.0000  0.9990  1.0000  1.0000 
```

```
      APN                AD               ADM                FOM        
 Min.   :0.3603   Min.   :16.92   Min.   :5.044   Min.   :0.9655  
 1st Qu.:0.3631   1st Qu.:16.95   1st Qu.:5.072   1st Qu.:0.9942  
 Median :0.3631   Median :16.95   Median :5.072   Median :1.0005  
 Mean   :0.3633   Mean   :16.95   Mean   :5.076   Mean   :0.9971  
 3rd Qu.:0.3631   3rd Qu.:16.95   3rd Qu.:5.072   3rd Qu.:1.0037  
 Max.   :0.3653   Max.   :16.97   Max.   :5.114   Max.   :1.0045  
```

Removing sets of k genes

```
   Min. 1st Qu.  Median    Mean 3rd Qu.    Max. 
 0.5432  0.8000  0.8475  0.8401  0.8909  0.9821 
```

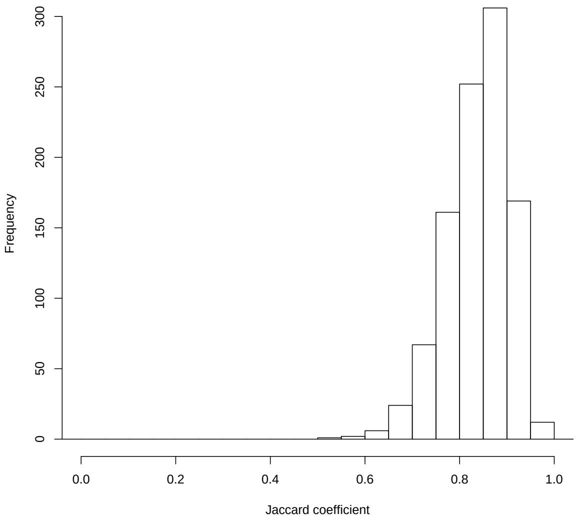

Figure 45: Removing two thirds of the genes: distribution of Jaccard coefficients

6.4.6 Validation measures

```
Clustering Methods:
 kmeans

Cluster sizes:
 2 3 4 5 6

Validation Measures:
                            2        3        4        5        6

kmeans APN             0.0187   0.0227   0.0755   0.1782   0.2505
       AD             15.1103  14.3905  14.1193  14.0215  13.8292
       ADM             0.2734   0.2917   0.7709   1.7650   2.3576
       FOM             0.8845   0.8452   0.8288   0.8166   0.8030
       Connectivity   51.4306 107.7202 131.1516 143.7472 155.8504
       Dunn            0.4221   0.3990   0.3982   0.4320   0.4480
       Silhouette      0.1893   0.1140   0.0852   0.0931   0.0880

Optimal Scores:

             Score   Method Clusters
APN           0.0187 kmeans 2
AD           13.8292 kmeans 6
ADM           0.2734 kmeans 2
FOM           0.8030 kmeans 6
Connectivity 51.4306 kmeans 2
Dunn          0.4480 kmeans 6
Silhouette    0.1893 kmeans 2
```

7 Sotiriou et al. (2006) dataset

7.1 LAS bicluster

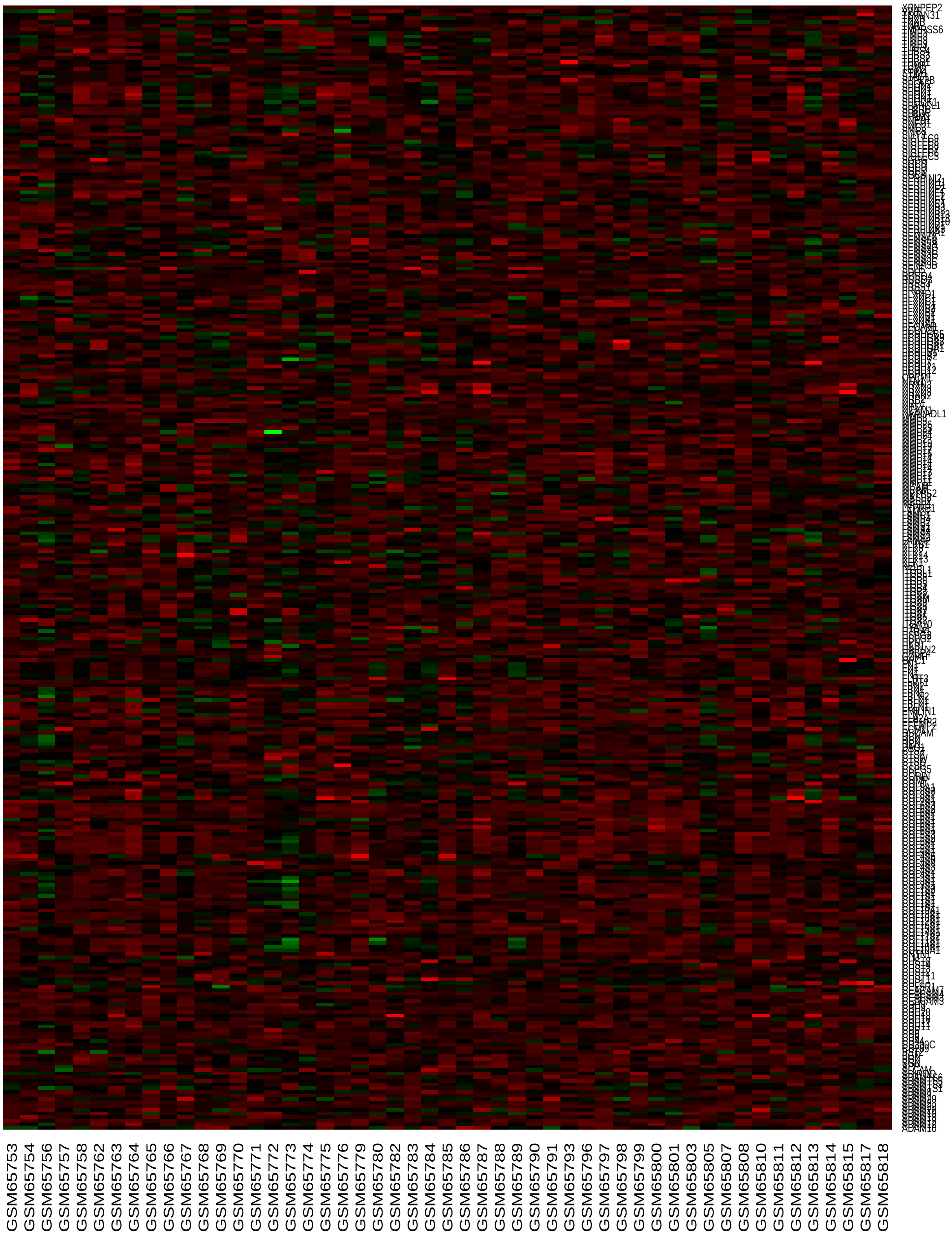

Figure 46: Heatmap of the LAS bicluster

LAS genes

ADAM10	ADAM12	ADAM12	ADAM18	ADAM19	ADAM22	ADAM22	ADAM23
ADAM29	ADAM7	ADAM8	ADAMTS1	ADAMTS3	ADAMTS5	ADAMTS6	ADIPOQ
ALCAM	APP	BGN	BGN	BGN	BST2	CD209	CD300C
CD44	CD6	CD6	CD6	CDH11	CDH11	CDH16	CDH18
CDH20	CDH4	CDH8	CEACAM3	CEACAM3	CEACAM4	CEACAM7	CELSR1
CHL1	CHST1	CHST11	CHST2	CHST3	CHST4	CHST8	CLU
CNTN1	COL10A1	COL10A1	COL11A1	COL11A1	COL11A2	COL14A1	COL15A1
COL16A1	COL17A1	COL18A1	COL18A1	COL19A1	COL1A1	COL1A1	COL1A1
COL1A1	COL1A2	COL1A2	COL3A1	COL3A1	COL3A1	COL4A1	COL4A2
COL4A3	COL4A4	COL4A6	COL4A6	COL5A1	COL5A1	COL5A1	COL5A2
COL5A2	COL5A3	COL6A1	COL6A1	COL6A1	COL6A1	COL6A1	COL6A2
COL6A2	COL6A3	COL7A1	COL8A1	COL8A2	COL8A2	COL9A1	COLQ
COMP	CORIN	CPE	CSPG5	CTSG	CTSO	CTSW	CTSZ
CTSZ	DAG1	DCN	DCN	DCN	DSCAM	ECM1	EFEMP2
EFEMP2	ELA2A	ELN	EMILIN1	FBLN1	FBLN1	FBLN1	FBLN2
FBN1	FBN1	FBN2	FLRT1	FLRT3	FN1	FN1	FN1
FN1	GPC1	GZMH	HABP4	HAPLN2	HAS1	HPN	HSPG2
HSPG2	HTRA1	HYAL2	ITGA10	ITGA3	ITGA5	ITGA7	ITGA7
ITGA8	ITGA9	ITGAM	ITGAX	ITGB3	ITGB4	ITGB5	ITGB5
ITGB8	ITGBL1	ITGBL1	KEL	KLK1	KLK13	KLK14	KLK2
KLK3	KLKB1	LAMA2	LAMA2	LAMA4	LAMA4	LAMB1	LAMB2
LAMB4	LAMC1	LAMC2	LEPRE1	MASP1	MASP2	MASP2	MBTPS2
MCAM	MCAM	MMP11	MMP11	MMP11	MMP13	MMP14	MMP14
MMP14	MMP14	MMP16	MMP17	MMP19	MMP19	MMP2	MMP24
MMP24	MMP25	MMP26	MMP3	MMP8	NAALADL1	NCAM1	NID2
NRP1	NRP2	NRXN2	NRXN3	NRXN3	NRXN3	NTN1	OPCML
PCDH1	PCDH12	PCDH21	PCDH7	PCDH7	PCDHA2	PCDHB1	PCDHGA1
PCDHGA1	PCDHGA3	PCDHGA9	PCDHGB5	PCOLCE	PECAM1	PLXNA1	PLXNA1
PLXNB1	PLXNB2	PLXNB2	PLXNC1	PLXNC1	PLXNC1	PLXND1	PRG2
PRSS1	PRSS3	ROBO3	ROBO4	SDC2	SELE	SEMA3B	SEMA3C
SEMA3F	SEMA3G	SEMA4C	SEMA4G	SEMA5A	SEMA5A	SEMA7A	SERPINA1
SERPINA3	SERPINB1	SERPINB10	SERPINB13	SERPINB13	SERPINB3	SERPINB3	SERPINC1
SERPINE1	SERPINE2	SERPINF1	SERPINF2	SERPING1	SERPINH1	SERPINI2	SGCA
SGCD	SGCD	SGCD	SGCG	SIGLEC5	SIGLEC6	SIGLEC7	SIGLEC8
SIGLEC8	SIGLEC9	SLIT3	SMC3	SMC3	SNED1	SNED1	SPAM1
SPARC	SPARC	SPARCL1	SPOCK1	SPON1	SPON1	SPON1	SPON1
SPON2	SPPL2B	STAG1	STIM1	TGM2	TGM5	THBS1	THBS2
THBS3	THBS4	TIMP1	TIMP3	TIMP3	TIMP3	TIMP3	TMPRSS6
TNR	TNXB	TNXB	TSPAN31	VWF	XPNPEP2		

LAS samples

GSM65753 GSM65754 GSM65756 GSM65757 GSM65758 GSM65762 GSM65763 GSM65764
GSM65765 GSM65766 GSM65767 GSM65768 GSM65769 GSM65770 GSM65771 GSM65772
GSM65773 GSM65774 GSM65775 GSM65776 GSM65779 GSM65780 GSM65782 GSM65783
GSM65784 GSM65785 GSM65786 GSM65787 GSM65788 GSM65789 GSM65790 GSM65791
GSM65793 GSM65796 GSM65797 GSM65798 GSM65799 GSM65800 GSM65801 GSM65803
GSM65805 GSM65807 GSM65808 GSM65810 GSM65811 GSM65812 GSM65813 GSM65814
GSM65815 GSM65817 GSM65818

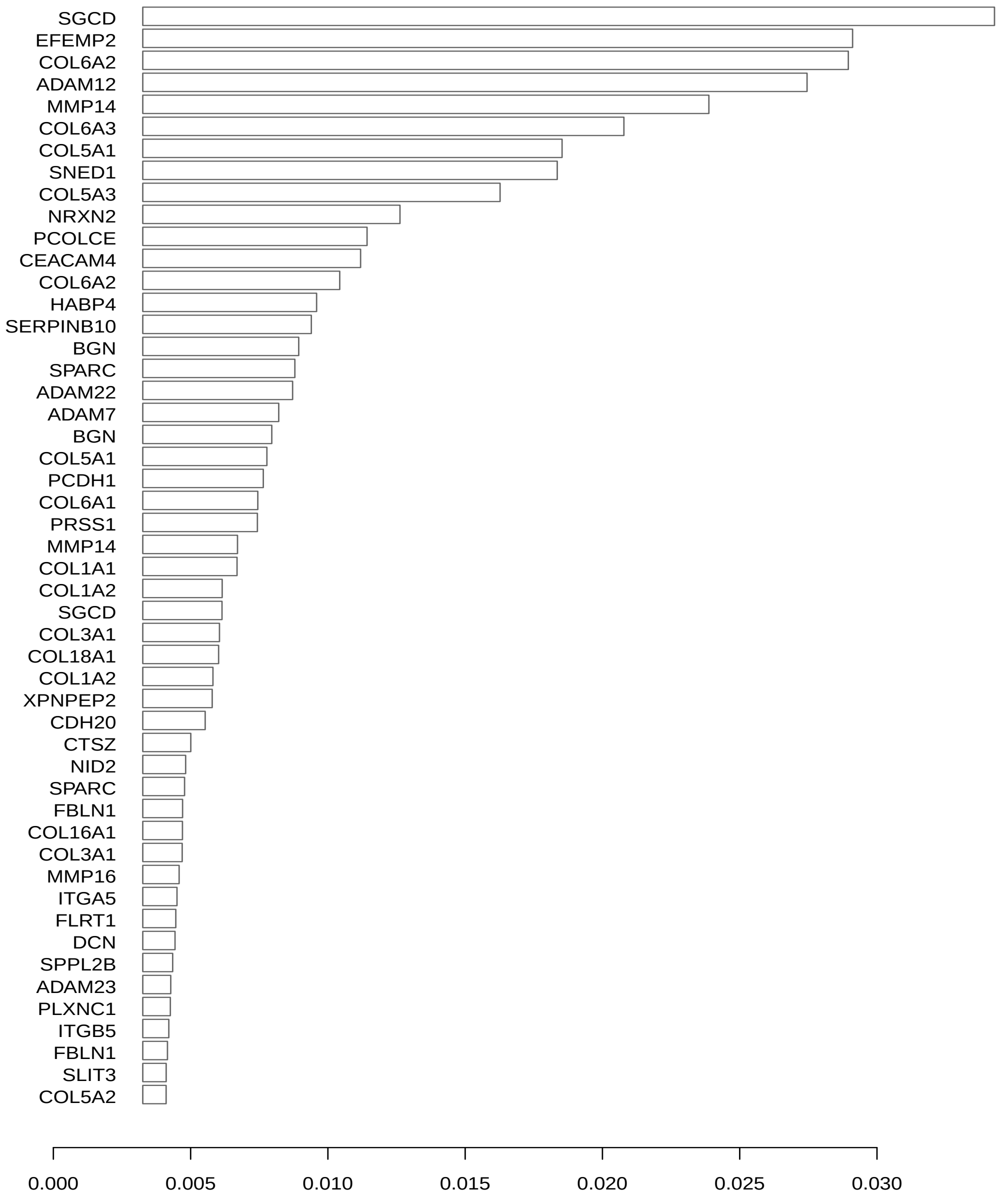

Figure 47: Most informative genes for the LAS bicluster

`Number of informative genes of the LAS bicluster that also belong to the ECM3 consensus list: 29 of 34 (85%)`

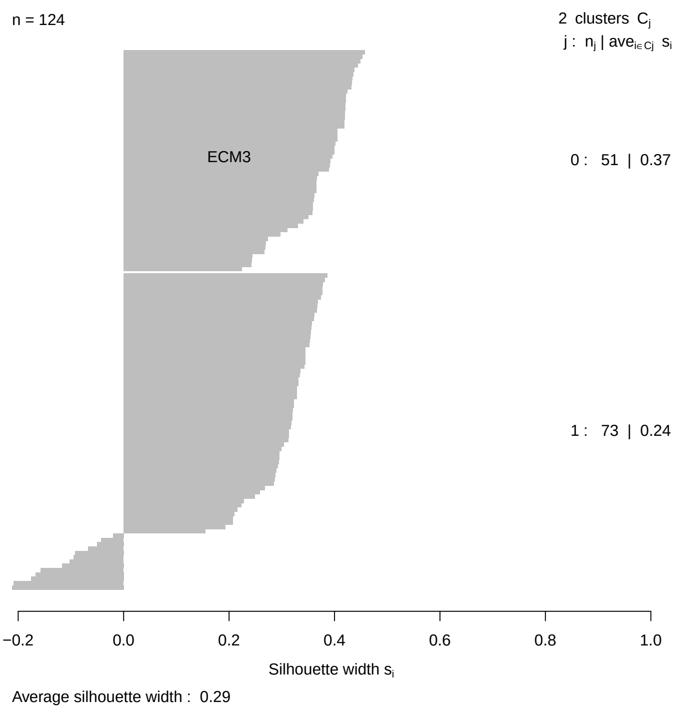

Figure 48: Silhoutte plot for the LAS bicluster

Connectivity	Dunn Index
12.88	0.38

Table 36: Connectivity validation measure and Dunn Index of LAS partitioning

7.2 IRCC-KM bicluster

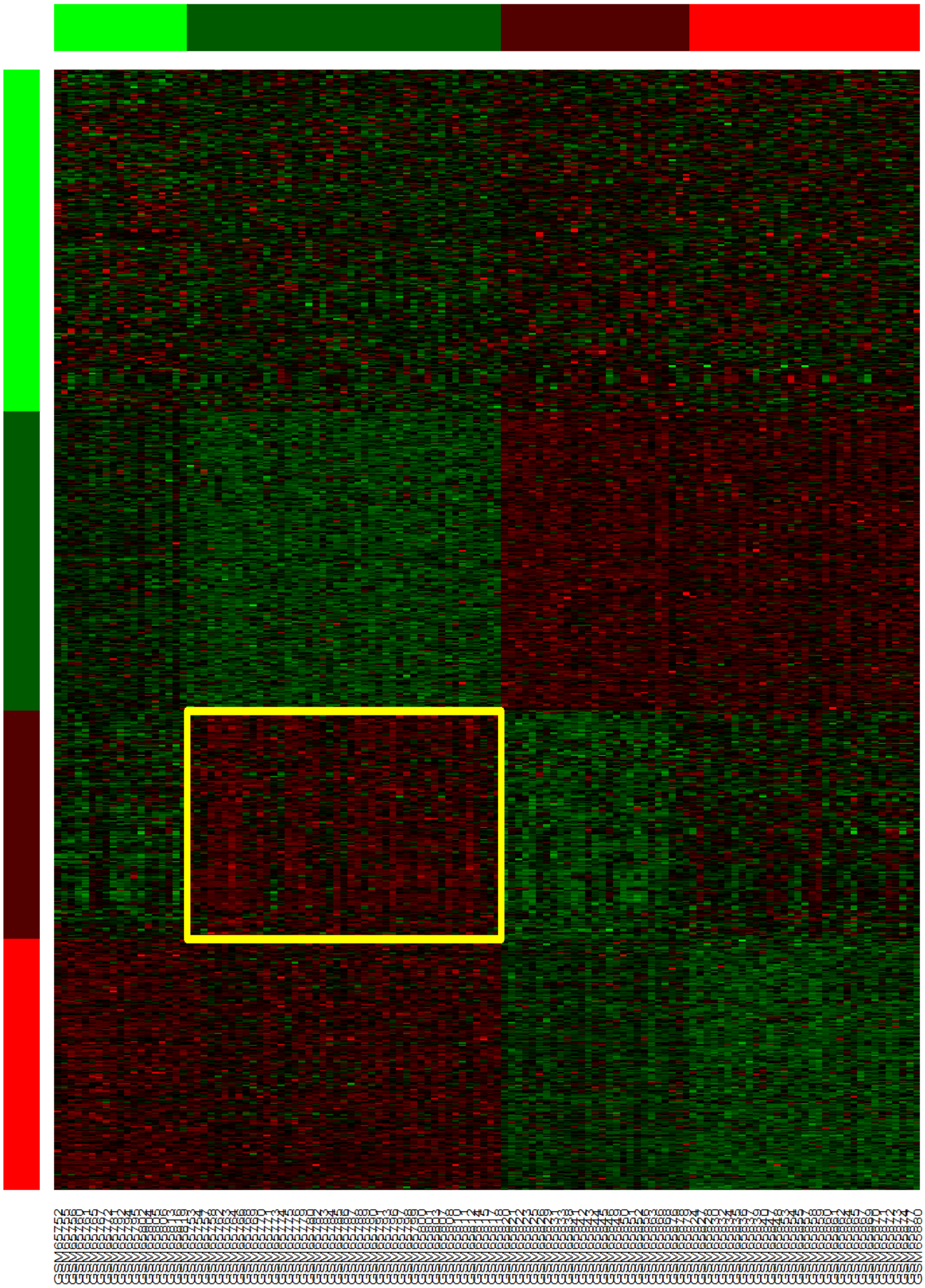

Figure 49: Heatmap of the IRCC-KM bicluster

IRCC-KM genes

ADAM12	ADAM12	ADAMTS1	ADAMTS5	ADAMTSL2	ADIPOQ	ALCAM	ALCAM
APP	APP	BGN	BGN	BGN	CD36	CDH11	CDH11
CDH5	CELSR1	CHL1	CHST10	CHSY1	CLU	CLU	COL10A1
COL10A1	COL11A1	COL11A1	COL14A1	COL15A1	COL16A1	COL18A1	COL1A1
COL1A1	COL1A1	COL1A2	COL1A2	COL3A1	COL3A1	COL3A1	COL4A1
COL4A5	COL4A6	COL5A1	COL5A1	COL5A1	COL5A2	COL5A2	COL5A3
COL5A3	COL6A1	COL6A1	COL6A1	COL6A1	COL6A2	COL6A2	COL6A3
COL8A1	COL8A2	COL8A2	COMP	CORIN	CPE	CTSK	CTSO
DAG1	DCN	DCN	DCN	DCN	ECM1	EFEMP1	EFEMP2
EFEMP2	ELN	EMILIN1	ENPEP	FBLN1	FBLN1	FBLN1	FBLN2
FBLN5	FBN1	FBN1	FLRT2	FLRT2	FN1	FN1	FN1
FN1	FN1	GPC1	HSPG2	HSPG2	HTRA1	HYAL2	ITGB1
ITGB5	ITGB5	ITGBL1	ITGBL1	LAMA2	LAMA2	LAMA2	LAMA4
LAMA4	LAMB1	LAMB1	LAMB2	LAMC1	LAMC1	LEPRE1	MATN3
MCAM	MCAM	MCAM	MGEA5	MMP11	MMP11	MMP13	MMP14
MMP14	MMP16	MMP19	MMP2	MMP3	NID1	NID1	NID2
NRP1	PCDH1	PCDH7	PCDH7	PCDHGA1	PCDHGA1	PCDHGA1	PCDHGA1
PCOLCE	PECAM1	PLXNA1	PLXNB1	PLXNB2	PLXNC1	PLXND1	PLXND1
PLXDC1	PRSS16	ROBO1	SDC4	SEMA3C	SEMA3G	SEMA5A	SEMA5A
SERPINA3	SERPINB1	SERPINE1	SERPINE2	SERPINF1	SERPING1	SERPINH1	SGCB
SGCE	SLIT2	SLIT3	SNED1	SPARC	SPARC	SPARCL1	SPG20
SPOCK1	SPON1	SPON1	SPON1	SPON1	SPON2	STAG1	THBS2
THBS3	THBS4	TIMP3	TIMP3	TIMP3	TIMP3	TNXB	TSPAN15
VWF							

IRCC-KM samples

GSM65753	GSM65754	GSM65757	GSM65758	GSM65762	GSM65763	GSM65764	GSM65766
GSM65768	GSM65769	GSM65770	GSM65771	GSM65773	GSM65774	GSM65775	GSM65776
GSM65779	GSM65780	GSM65782	GSM65783	GSM65784	GSM65785	GSM65786	GSM65787
GSM65788	GSM65789	GSM65790	GSM65791	GSM65793	GSM65796	GSM65797	GSM65798
GSM65799	GSM65800	GSM65801	GSM65803	GSM65807	GSM65808	GSM65810	GSM65811
GSM65812	GSM65814	GSM65815	GSM65817	GSM65818			

7.2.1 Comparing LAS and IRCC-KM biclusters

	No ECM	ECM3
No ECM3	553	171
ECM3	46	139
Jaccard similarity	0.39	

Table 37: Comparing gene lists: LAS (column) vs. IRCC-KM bicluster (row)

	No ECM	ECM3
No ECM3	73	6
ECM3	0	45
Jaccard similarity	0.88	

Table 38: Comparing sample lists: LAS (column) vs. IRCC-KM bicluster (row)

7.3 IRCC-HC bicluster

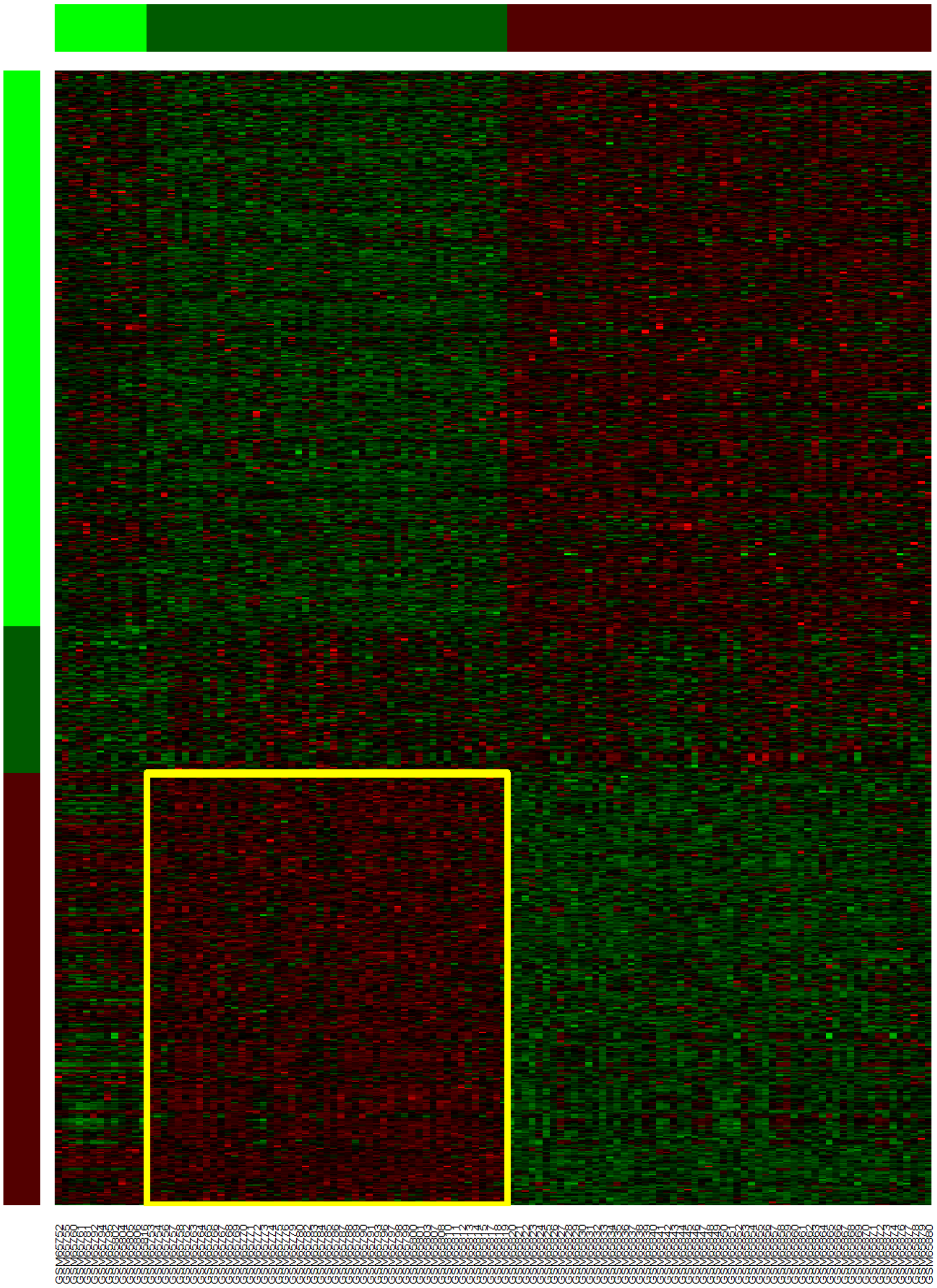

Figure 50: Heatmap of the IRCC-HC bicluster

IRCC-HC genes

ADAM12	ADAM12	ADAM18	ADAM19	ADAM20	ADAM22	ADAM22	ADAM23
ADAM29	ADAM7	ADAM8	ADAMTS1	ADAMTS3	ADAMTS5	ADAMTS6	ADIPOQ
APP	APP	APP	BGN	BGN	BGN	CD209	CD300C
CD44	CD6	CD6	CD6	CDH11	CDH11	CDH16	CDH18
CDH20	CDH4	CDH8	CEACAM3	CEACAM3	CEACAM4	CEACAM7	CELSR1
CHL1	CHST11	CHST2	CHST3	CHST4	CHST8	CHSY1	CNTN1
CNTN6	COL10A1	COL10A1	COL11A1	COL11A1	COL11A2	COL14A1	COL15A1
COL16A1	COL17A1	COL18A1	COL18A1	COL19A1	COL1A1	COL1A1	COL1A1
COL1A1	COL1A2	COL1A2	COL3A1	COL3A1	COL3A1	COL4A1	COL4A1
COL4A2	COL4A2	COL4A3	COL4A4	COL4A6	COL5A1	COL5A1	COL5A1
COL5A2	COL5A2	COL5A3	COL5A3	COL6A1	COL6A1	COL6A1	COL6A1
COL6A1	COL6A1	COL6A2	COL6A2	COL6A3	COL7A1	COL8A1	COL8A2
COL8A2	COL9A1	COL9A3	COLQ	COMP	CORIN	CSPG4	CSPG4
CSPG5	CTSB	CTSG	CTSK	CTSO	CTSZ	CTSZ	DCN
DCN	DCN	DCN	DSCAM	EFEMP2	EFEMP2	ELA2A	ELN
EMILIN1	FBLN1	FBLN1	FBLN1	FBLN2	FBLN5	FBN1	FBN1
FBN2	FBN2	FLRT1	FLRT2	FLRT2	FLRT3	FN1	FN1
FN1	FN1	GPC1	HABP4	HAPLN2	HAS1	HPN	HSPG2
HSPG2	HTRA1	ITGA10	ITGA3	ITGA5	ITGA7	ITGA7	ITGA8
ITGA9	ITGAM	ITGAX	ITGB1	ITGB3	ITGB3	ITGB3	ITGB4
ITGB4	ITGB5	ITGB5	ITGB8	ITGBL1	ITGBL1	KEL	KLK1
KLK13	KLK2	KLK3	KLKB1	LAMA2	LAMA2	LAMA2	LAMA4
LAMA4	LAMA4	LAMB1	LAMB1	LAMB4	LAMC1	LAMC1	LAMC2
LEPRE1	MASP1	MASP2	MASP2	MATN3	MBTPS2	MCAM	MCAM
MCAM	MGEA5	MMP11	MMP11	MMP11	MMP13	MMP14	MMP14
MMP14	MMP14	MMP16	MMP17	MMP19	MMP19	MMP2	MMP24
MMP24	MMP25	MMP26	MMP3	MMP8	NAALADL1	NCAM1	NCAM1
NID1	NID2	NRP1	NRP2	NRP2	NRXN2	NRXN3	NRXN3
NRXN3	NTN1	OPCML	P11	PCDH1	PCDH12	PCDH21	PCDH7
PCDH7	PCDHA2	PCDHB1	PCDHGA1	PCDHGA1	PCDHGA1	PCDHGA1	PCDHGA3
PCDHGA9	PCDHGB5	PCOLCE	PECAM1	PLXNA1	PLXNA1	PLXNB1	PLXNB2
PLXNB2	PLXNC1	PLXNC1	PLXNC1	PLXND1	PLXND1	PRG2	PRSS1
PRSS16	PRSS3	ROBO1	ROBO3	ROBO4	SDC2	SELE	SEMA3B
SEMA3F	SEMA3G	SEMA4G	SEMA5A	SEMA5A	SEMA7A	SERBP1	SERPINA1
SERPINB10	SERPINB13	SERPINB13	SERPINB3	SERPINB3	SERPINB4	SERPINB7	SERPINB8
SERPINC1	SERPIND1	SERPINE1	SERPINE1	SERPINE2	SERPINF1	SERPINF2	SERPING1
SERPINH1	SERPINI2	SGCA	SGCB	SGCB	SGCD	SGCD	SGCD
SGCE	SGCG	SIGLEC5	SIGLEC6	SIGLEC7	SIGLEC7	SIGLEC8	SIGLEC8
SIGLEC9	SLIT2	SLIT3	SMC3	SMC3	SNED1	SNED1	SPAM1
SPAM1	SPARC	SPARC	SPARCL1	SPG20	SPOCK1	SPON1	SPON1
SPON1	SPON1	SPON2	SPPL2B	STAG1	STIM1	TGM5	THBS1
THBS2	THBS3	THBS4	TIMP1	TIMP2	TIMP3	TIMP3	TIMP3
TIMP3	TMPRSS6	TNR	TNXB	TNXB	TSPAN12	TSPAN31	TSPAN32
VWF	XPNPEP2						

IRCC-HC samples

GSM65753 GSM65754 GSM65756 GSM65757 GSM65758 GSM65762 GSM65763 GSM65764
GSM65765 GSM65766 GSM65767 GSM65768 GSM65769 GSM65770 GSM65771 GSM65772
GSM65773 GSM65774 GSM65775 GSM65776 GSM65779 GSM65780 GSM65782 GSM65783
GSM65784 GSM65785 GSM65786 GSM65787 GSM65788 GSM65789 GSM65790 GSM65791
GSM65793 GSM65796 GSM65797 GSM65798 GSM65799 GSM65800 GSM65801 GSM65803
GSM65807 GSM65808 GSM65810 GSM65811 GSM65812 GSM65813 GSM65814 GSM65815
GSM65817 GSM65818 GSM65819

7.3.1 Comparing LAS and IRCC-HC biclusters

	No ECM	ECM3
No ECM3	544	19
ECM3	55	291
Jaccard similarity	0.80	

Table 39: Comparing gene lists: LAS (column) vs. IRCC-HC bicluster (row)

	No ECM	ECM3
No ECM3	72	1
ECM3	1	50
Jaccard similarity	0.96	

Table 40: Comparing sample lists: LAS (column) vs. IRCC-HC bicluster (row)

7.4 CCSS bicluster

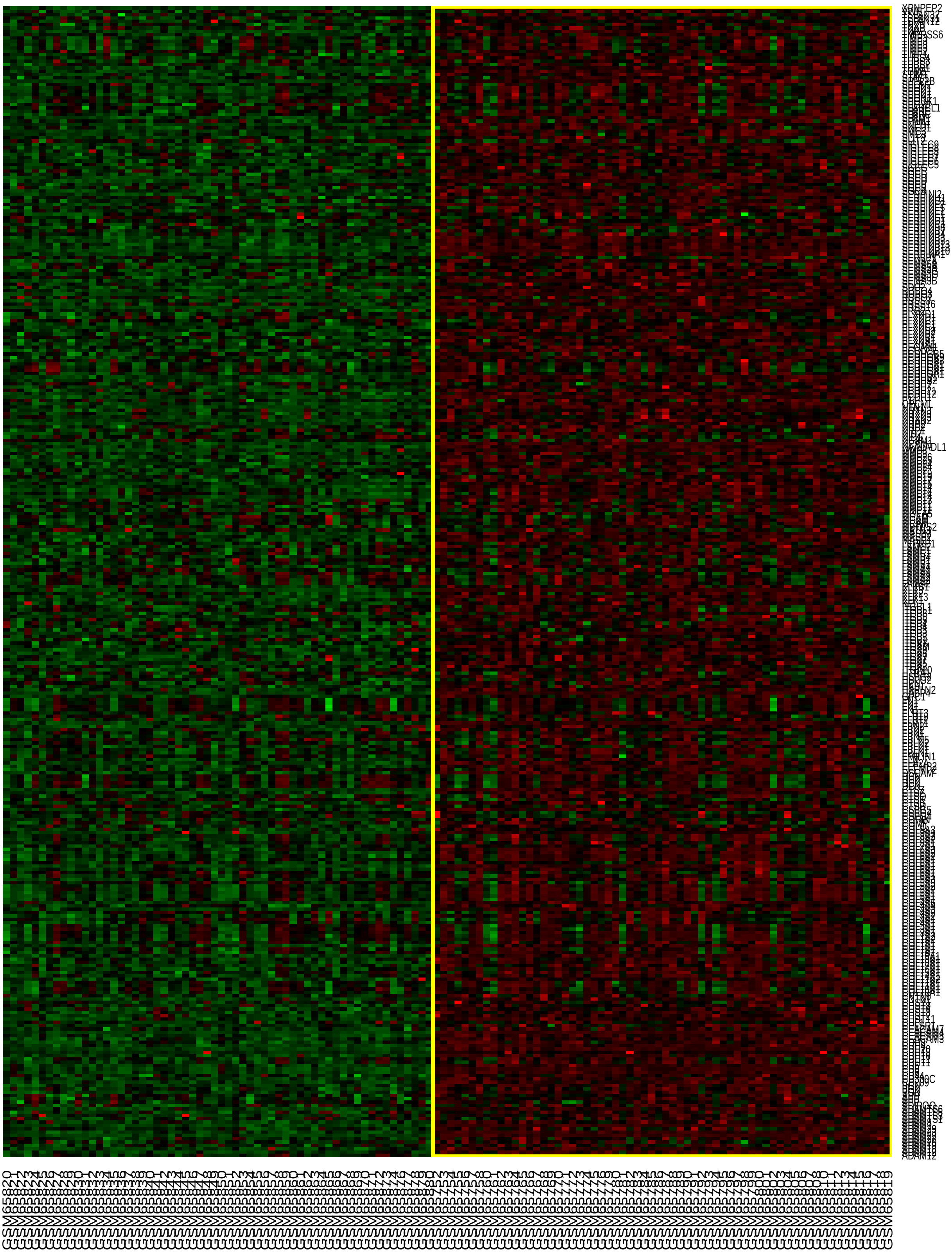

Figure 51: Heatmap of the CCSS bicluster

7.4.1 Comparing LAS and CCSS biclusters

	No ECM	ECM3
No ECM3	60	0
ECM3	13	51
Jaccard similarity	0.80	

Table 41: Comparing sample lists: LAS (column) vs. CCSS (row) biclusters

7.4.2 Prediction strength for CCSS

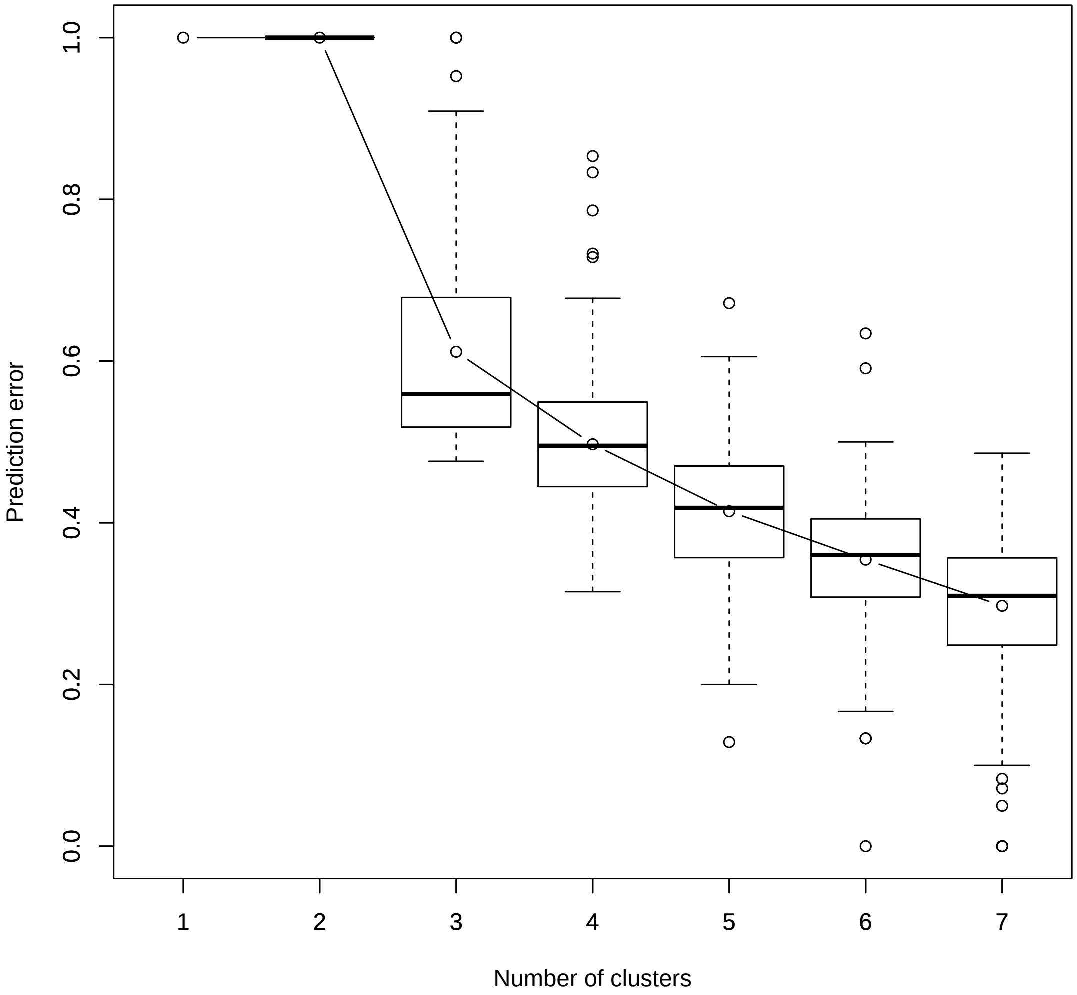

7.4.3 Consensus clustering

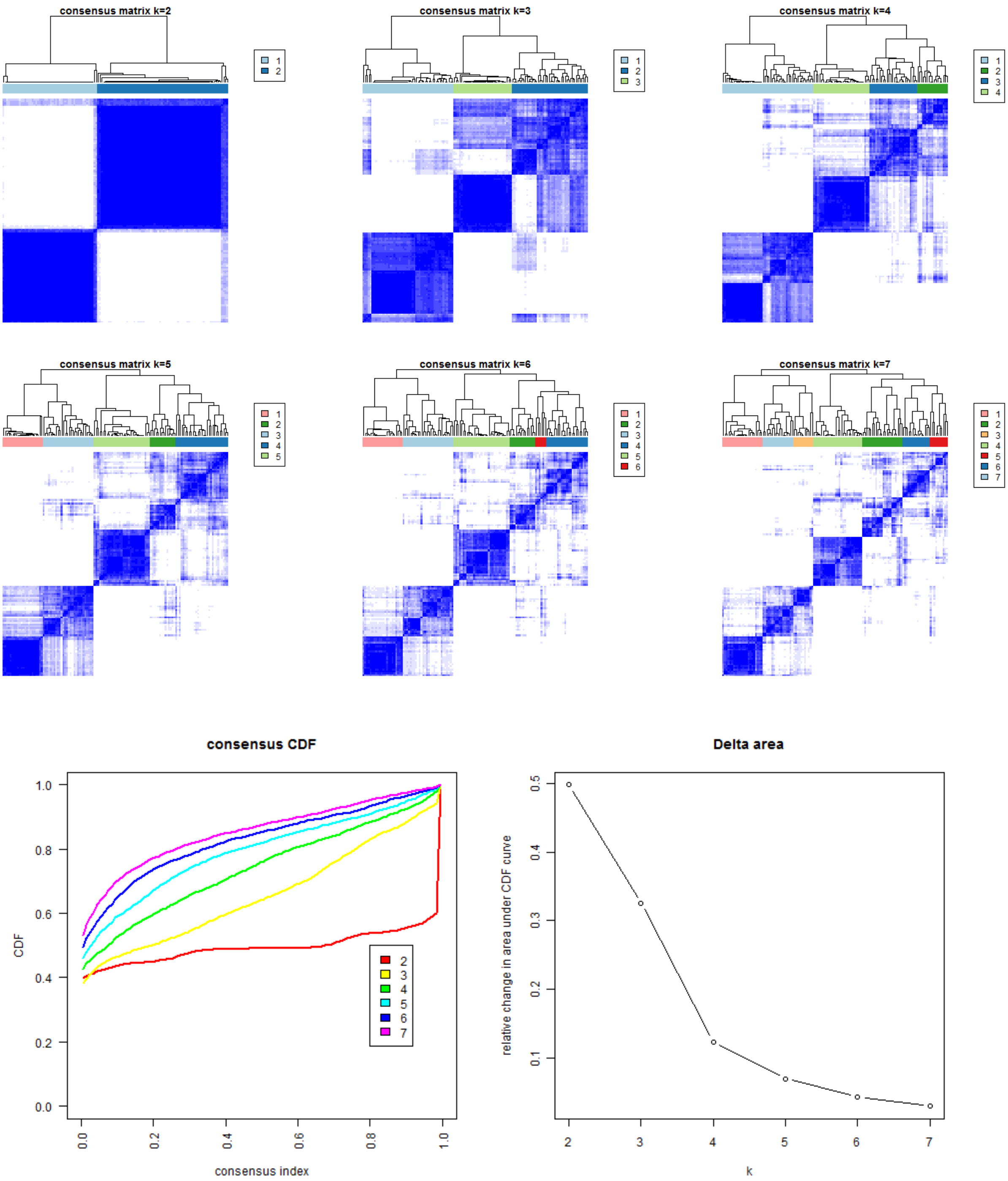

Figure 52: Statistical significance of CCSS clustering (Consensus clustering)

7.4.4 Statistical significance

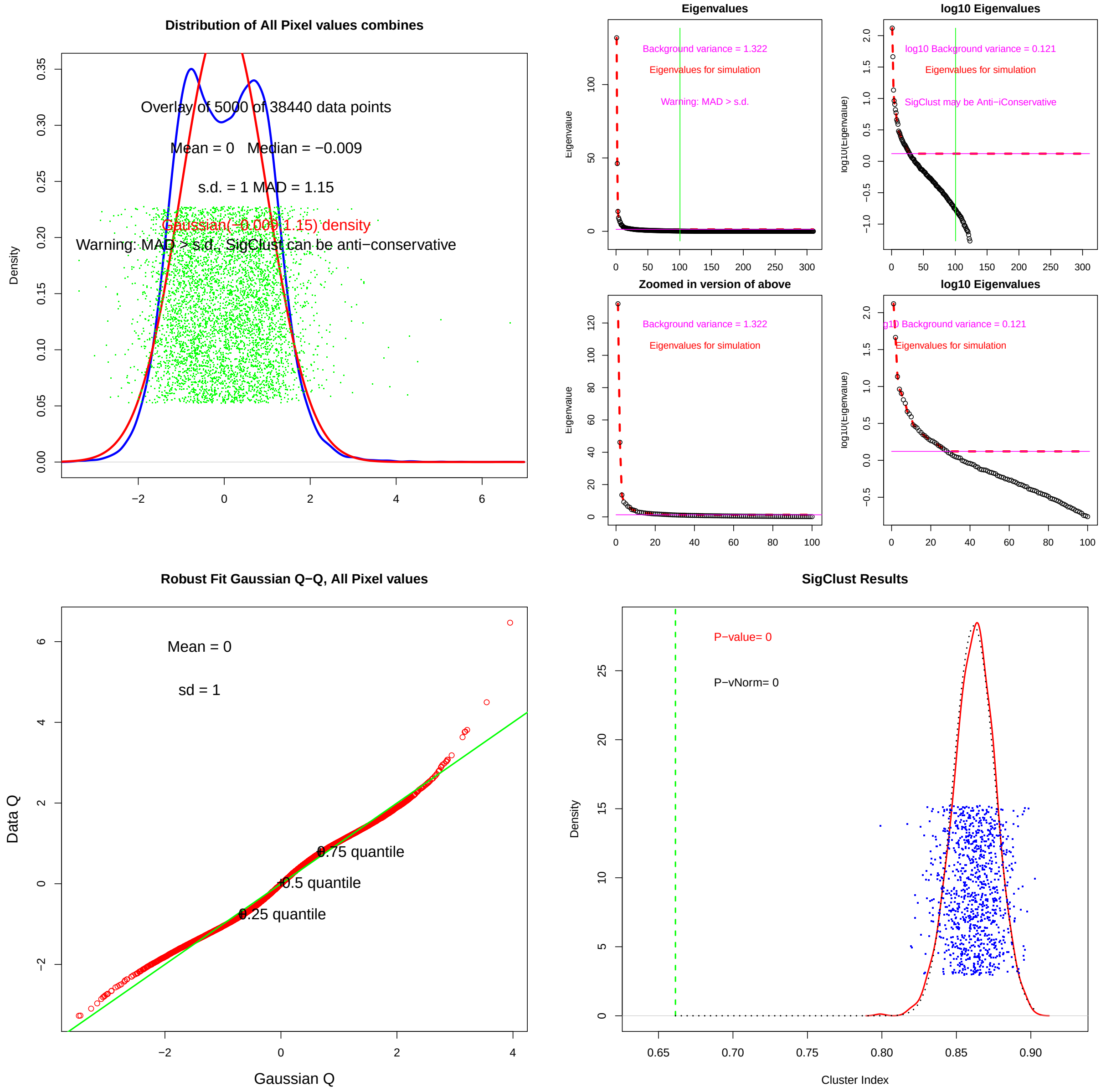

P-value	P-vNorm
0.0E+00	4.7E-46

Table 42: SigClust p-values

7.4.5 Cluster-wise assessment of cluster stability

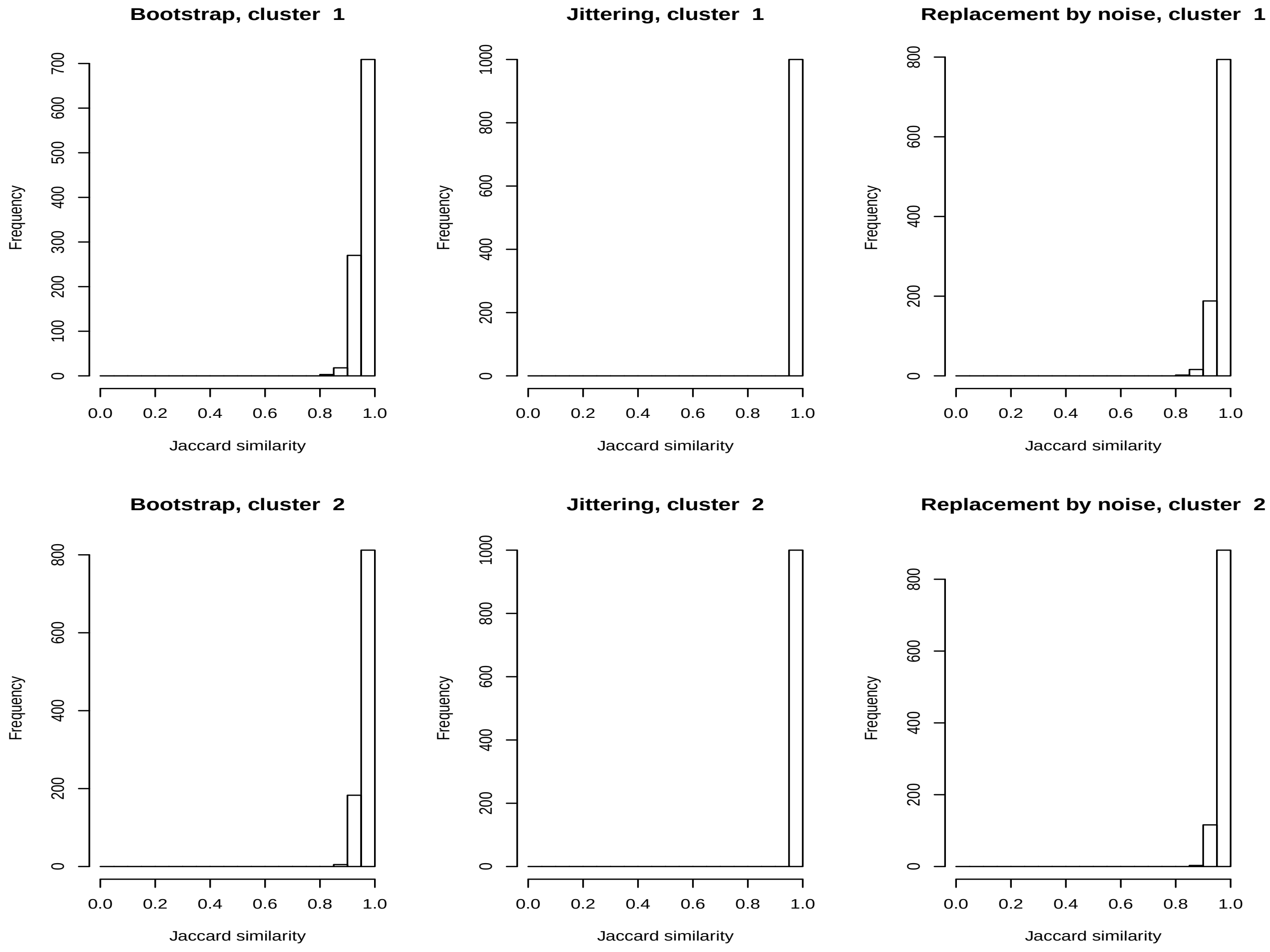


```
* Cluster stability assessment *
Cluster method:  kmeans
Full clustering results are given as parameter result
of the clusterboot object, which also provides further statistics
of the resampling results.
Number of resampling runs:  1000

Number of clusters found in data:  2

 Clusterwise Jaccard bootstrap mean:
[1] 0.9632207 0.9702989
dissolved:
[1] 0 0
recovered:
[1] 1000 1000
 Clusterwise Jaccard jittering mean:
[1] 1 1
dissolved:
[1] 0 0
recovered:
```

```
[1] 1000 1000
 Clusterwise Jaccard replacement by noise mean:
[1] 0.9684621 0.9744983
dissolved:
[1] 0 0
recovered:
[1] 1000 1000
```

Removing one sample

```
   Min. 1st Qu.  Median    Mean 3rd Qu.    Max.
      1       1       1       1       1       1
```

Removing one gene

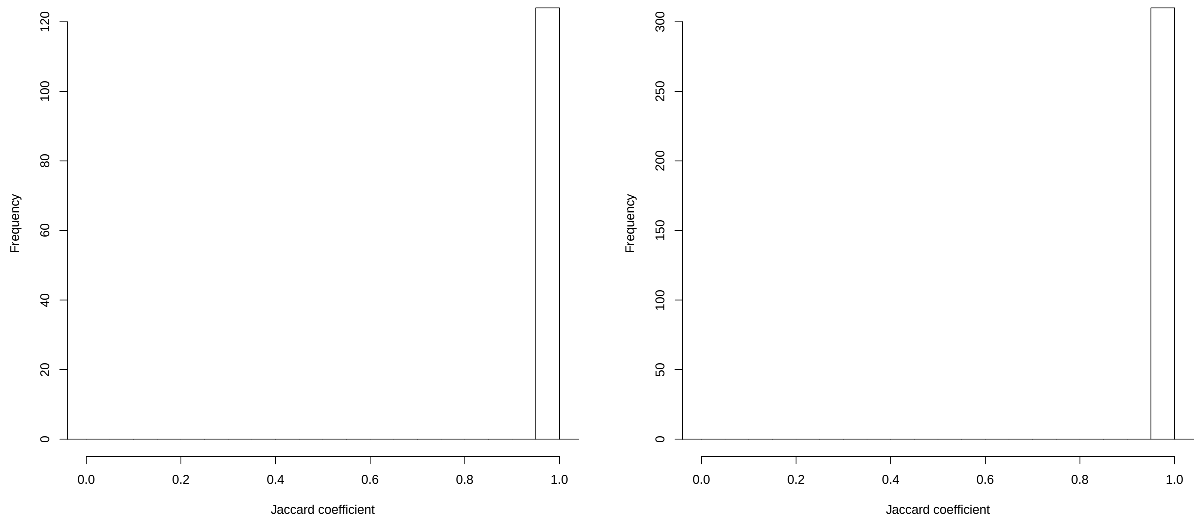

Figure 53: Removing one sample (left) and one gene (right) at a time: distributions of Jaccard coefficients

```
   Min. 1st Qu.  Median    Mean 3rd Qu.    Max.
      1       1       1       1       1       1

      APN                AD               ADM               FOM
 Min.   :0.1723   Min.   :20.32   Min.   :5.148   Min.   :0.2008
 1st Qu.:0.1723   1st Qu.:20.32   1st Qu.:5.148   1st Qu.:0.6111
 Median :0.1723   Median :20.32   Median :5.148   Median :0.8099
 Mean   :0.1723   Mean   :20.32   Mean   :5.148   Mean   :0.7572
 3rd Qu.:0.1723   3rd Qu.:20.32   3rd Qu.:5.148   3rd Qu.:0.9171
 Max.   :0.1723   Max.   :20.32   Max.   :5.148   Max.   :1.0077
```

Removing sets of k genes

```
   Min. 1st Qu.  Median    Mean 3rd Qu.    Max.
 0.8750  1.0000  1.0000  0.9999  1.0000  1.0000
```

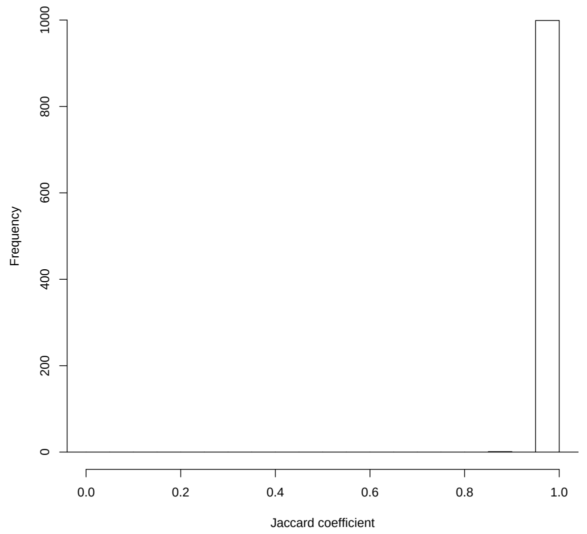

Figure 54: Removing two thirds of the genes: distribution of Jaccard coefficients

7.4.6 Validation measures

```
Clustering Methods:
 kmeans

Cluster sizes:
 2 3 4 5 6

Validation Measures:
                             2       3       4       5       6

kmeans APN            0.0000  0.0019  0.0016  0.0065  0.0054
       AD            18.8528 17.6381 16.9014 16.4625 16.0267
       ADM            0.0000  0.0433  0.0312  0.0979  0.0851
       FOM            0.7572  0.7133  0.6855  0.6715  0.6614
       Connectivity   0.0000 16.6972 45.2095 55.7548 61.4861
       Dunn           0.5753  0.4334  0.5102  0.5065  0.5062
       Silhouette     0.3458  0.2590  0.1643  0.1208  0.1130

Optimal Scores:

             Score   Method Clusters
APN           0.0000 kmeans 2
AD           16.0267 kmeans 6
ADM           0.0000 kmeans 2
FOM           0.6614 kmeans 6
Connectivity  0.0000 kmeans 2
Dunn          0.5753 kmeans 2
Silhouette    0.3458 kmeans 2
```

8 Ma et al. (2004) dataset (GDS807)

8.1 LAS bicluster

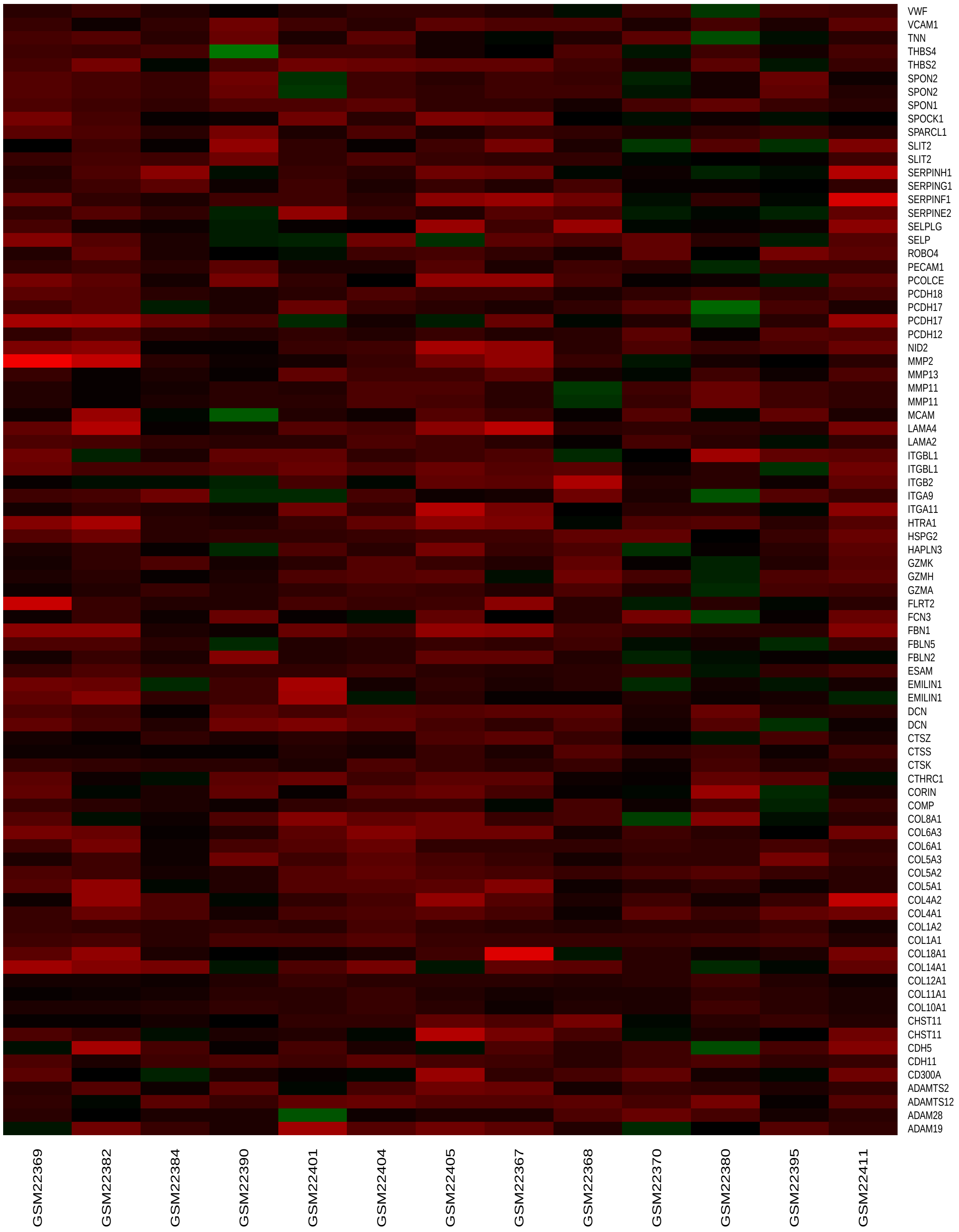

Figure 55: Heatmap of the LAS bicluster

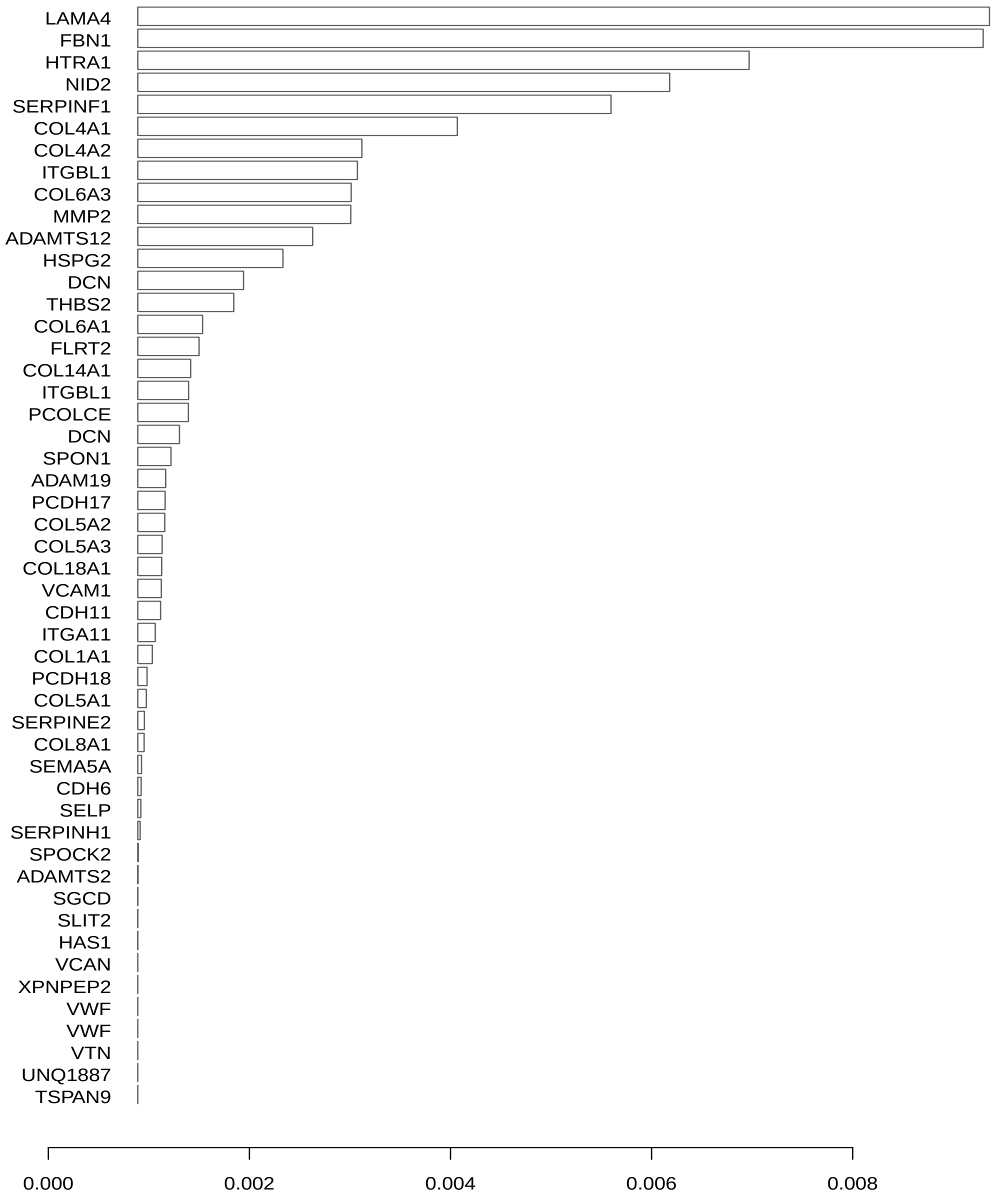

Figure 56: Most informative genes for the LAS bicluster

```
Number of informative genes of the LAS bicluster that also belong
to the ECM3 consensus list: 20 of 34 (59%)
```

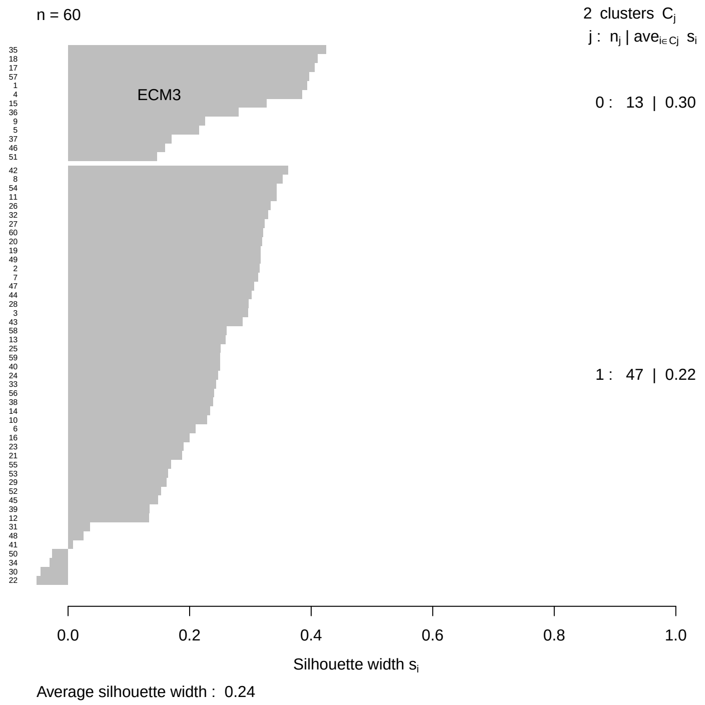

Figure 57: Silhoutte plot for the LAS bicluster

Connectivity	Dunn Index
12.97	0.39

Table 43: Connectivity validation measure and Dunn Index of LAS partitioning

8.2 IRCC-KM bicluster

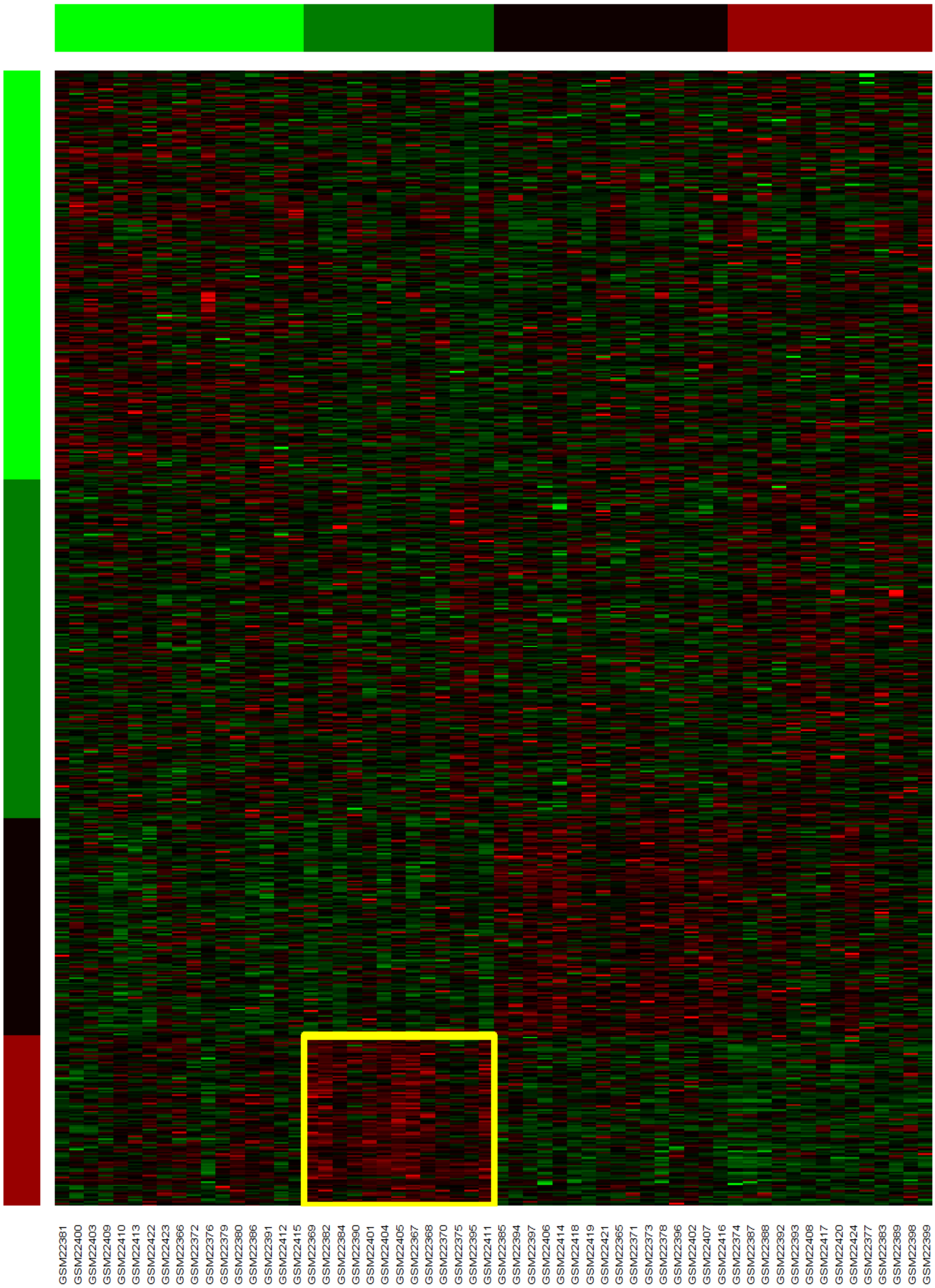

Figure 58: Heatmap of the IRCC-KM bicluster

IRCC-KM genes

ADAM19	ADAMTS1	ADAMTS12	ADAMTS2	ADAMTS6	CD300A
CD36	CD36	CDH11	CDH6	CHST11	CHST11
CHST2	COL10A1	COL11A1	COL12A1	COL14A1	COL15A1
COL18A1	COL1A1	COL1A2	COL4A1	COL4A2	COL5A1
COL5A2	COL5A3	COL6A1	COL6A3	COL8A1	COMP
CORIN	CPE	CTHRC1	CTSK	CTSS	CTSZ
DCN	DCN	EMILIN1	EMILIN1	ESAM	FBLN2
FBLN5	FBN1	FLRT2	GZMA	GZMH	GZMK
HAPLN3	HSPG2	HTRA1	ITGA11	ITGA5	ITGA7
ITGA8	ITGB2	ITGBL1	ITGBL1	LAMA2	LAMA4
MCAM	MME	MMP1	MMP11	MMP11	MMP13
MMP2	MMP3	NID2	PCDH12	PCDH17	PCDH17
PCDH18	PCDH7	PCOLCE	PCOLCE2	PLXNB3	ROBO4
SELE	SELP	SELPLG	SEMA4A	SERPINE2	SERPINF1
SERPING1	SERPINH1	SGCD	SLIT2	SLIT2	SPARC
SPARCL1	SPOCK1	SPOCK2	SPON1	SPON2	SPON2
THBS2	THBS4	TIMP3	TNN	TSPAN7	VCAM1
VWF	VCAN				

IRCC-KM samples

GSM22369	GSM22382	GSM22384	GSM22390	GSM22401	GSM22404
GSM22405	GSM22367	GSM22368	GSM22370	GSM22375	GSM22395
GSM22411					

8.2.1 Comparing LAS and IRCC-KM biclusters

	No ECM	ECM3
No ECM3	582	5
ECM3	25	79
Jaccard similarity	0.72	

Table 44: Comparing gene lists: LAS (column) vs. IRCC-KM bicluster (row)

	No ECM	ECM3
No ECM3	46	1
ECM3	1	12
Jaccard similarity	0.86	

Table 45: Comparing sample lists: LAS (column) vs. IRCC-KM bicluster (row)

8.3 IRCC-HC bicluster

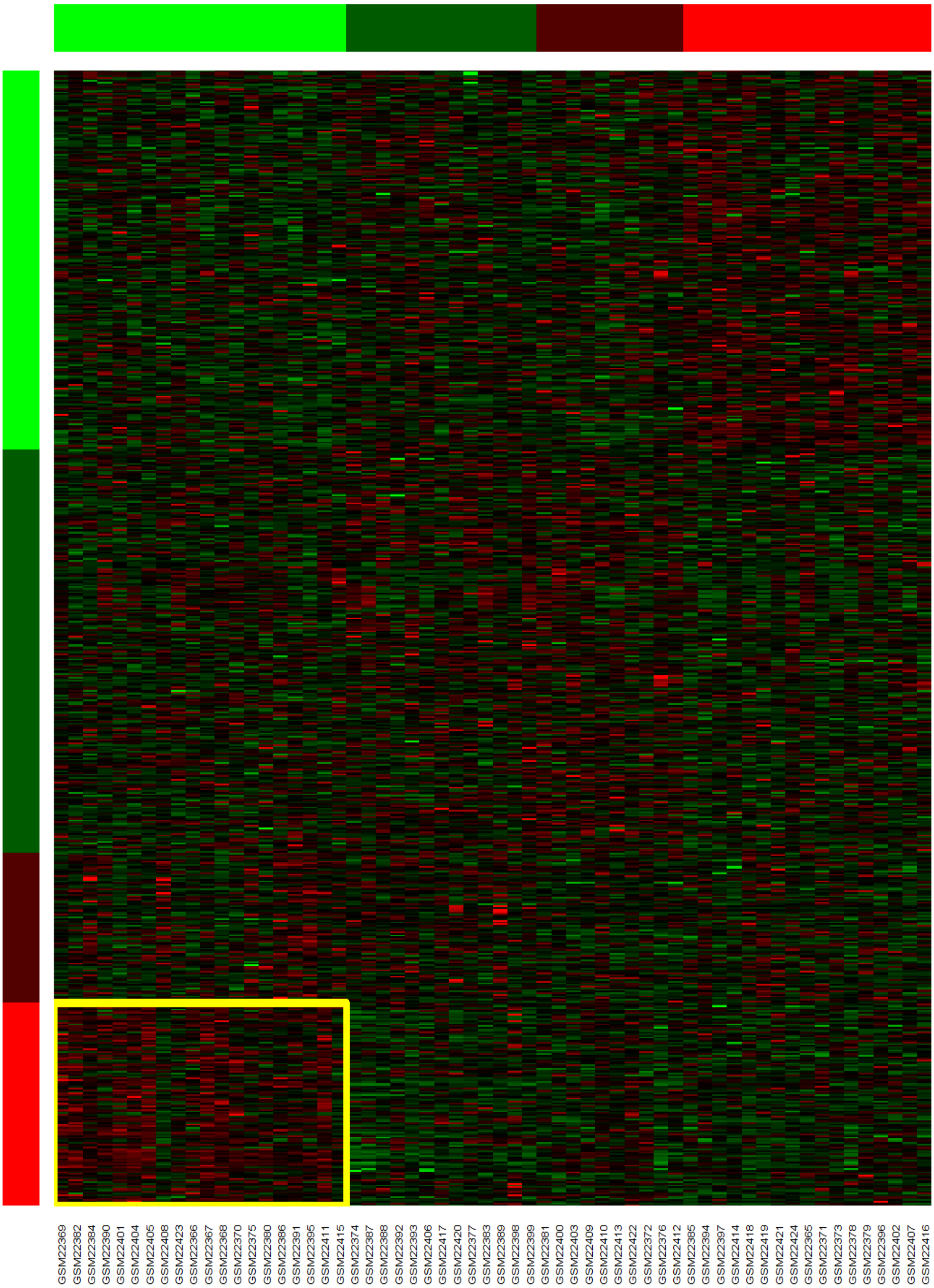

Figure 59: Heatmap of the IRCC-HC bicluster

IRCC-HC genes

ADAM19	ADAMDEC1	ADAMTS1	ADAMTS12	ADAMTS2	ADAMTS4
ADAMTSL1	ADIPOQ	CD300A	CD36	CD36	CD6
CDH11	CDH17	CDH26	CDH5	CHL1	CHST11
CHST11	CHST2	CNTN1	COL10A1	COL11A1	COL14A1
COL18A1	COL1A1	COL1A2	COL4A1	COL4A2	COL5A1
COL5A2	COL5A3	COL6A1	COL6A3	COL8A1	COL9A2
COMP	CTHRC1	CTSC	CTSH	CTSH	CTSK
CTSZ	DCN	DCN	EMILIN1	EMILIN1	ESAM
FBN1	FLRT2	GPC6	GZMA	GZMH	GZMK
HAPLN3	HAS1	HAS2	HSPG2	HTRA1	ICAM2
ITGA11	ITGA7	ITGA8	ITGB2	ITGBL1	ITGBL1
KLK15	LAMA2	LAMA4	MARCO	MATN3	MCAM
MMP1	MMP11	MMP11	MMP13	MMP2	MMP3
MMP9	NAALAD2	NAALADL1	NAPSB	NID2	PCDH12
PCDH17	PCDH17	PCDH18	PCDHB13	PCDHB18	PCOLCE
PCOLCE2	PECAM1	PLXDC1	RNPEP	ROBO4	SELE
SELP	SELPLG	SEMA4D	SEMA7A	SERPINE2	SERPINF1
SERPING1	SERPINH1	SGCD	SIGLEC7	SLIT2	SLIT2
SPARC	SPARCL1	SPG21	SPOCK1	SPOCK2	SPON1
SPON2	SPON2	STAG3	THBS2	THBS4	TSPAN32
VCAM1	VCAN	VWF	XPNPEP2		

IRCC-HC samples

GSM22369	GSM22382	GSM22384	GSM22390	GSM22401	GSM22404
GSM22405	GSM22408	GSM22423	GSM22366	GSM22367	GSM22368
GSM22370	GSM22375	GSM22380	GSM22386	GSM22391	GSM22395
GSM22411	GSM22415				

8.3.1 Comparing LAS and IRCC-HC biclusters

	No ECM	ECM3
No ECM3	558	9
ECM3	49	75
Jaccard similarity	0.56	

Table 46: Comparing gene lists: LAS (column) vs. IRCC-HC bicluster (row)

	No ECM	ECM3
No ECM3	40	0
ECM3	7	13
Jaccard similarity	0.65	

Table 47: Comparing sample lists: LAS (column) vs. IRCC-HC bicluster (row)

8.4 CCSS bicluster

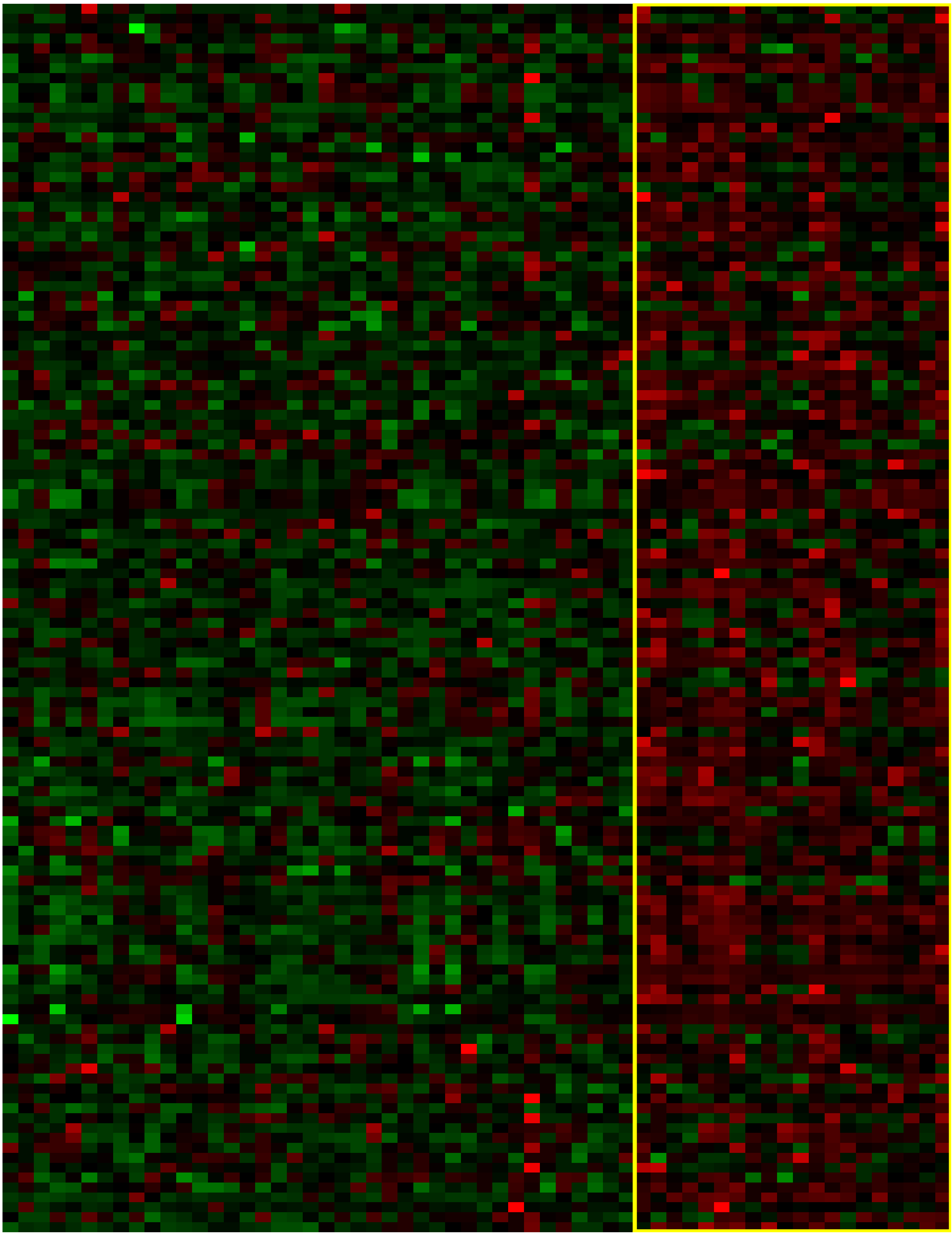

Figure 60: Heatmap of the CCSS bicluster

8.4.1 Comparing LAS and CCSS biclusters

	No ECM	ECM3
No ECM3	40	0
ECM3	7	13
Jaccard similarity	0.65	

Table 48: Comparing sample lists: LAS (column) vs. CCSS (row) biclusters

8.4.2 Prediction strength for CCSS

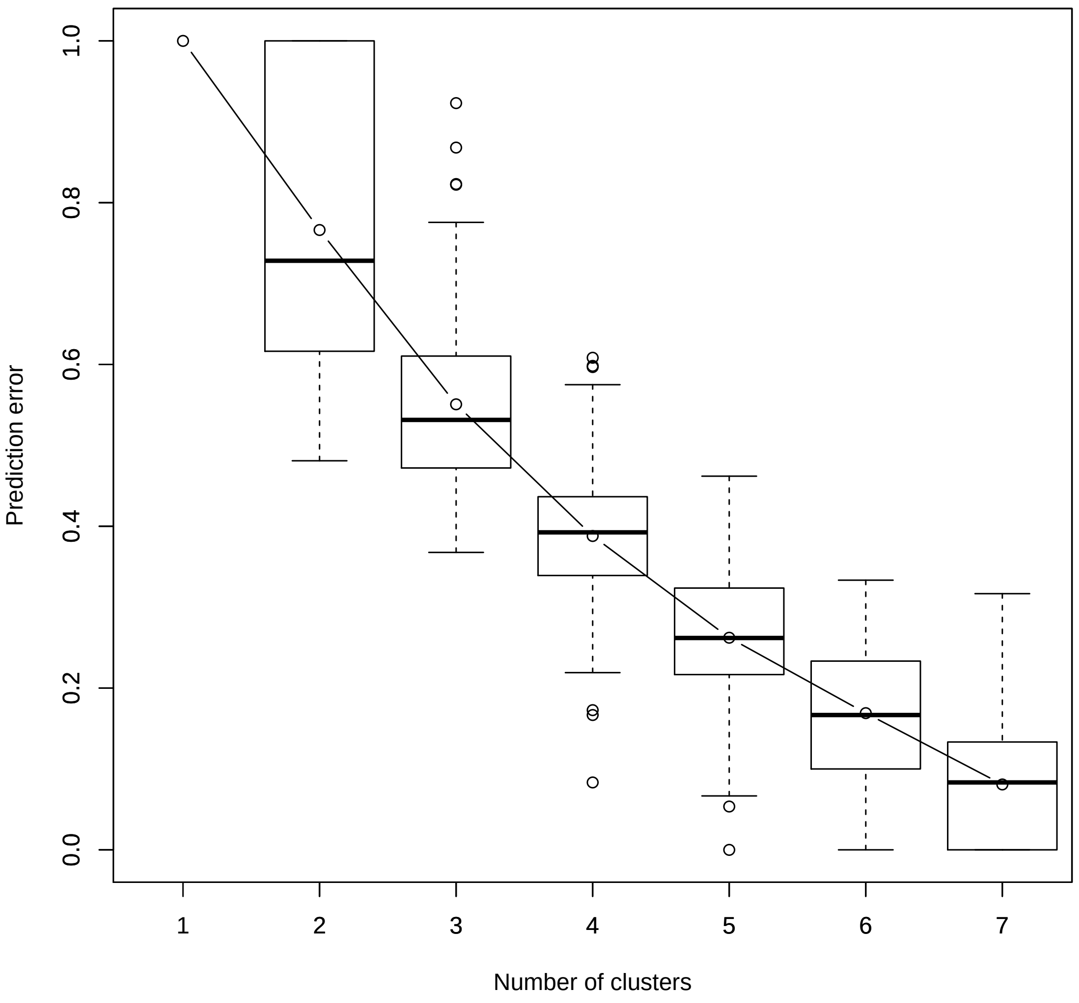

8.4.3 Consensus clustering

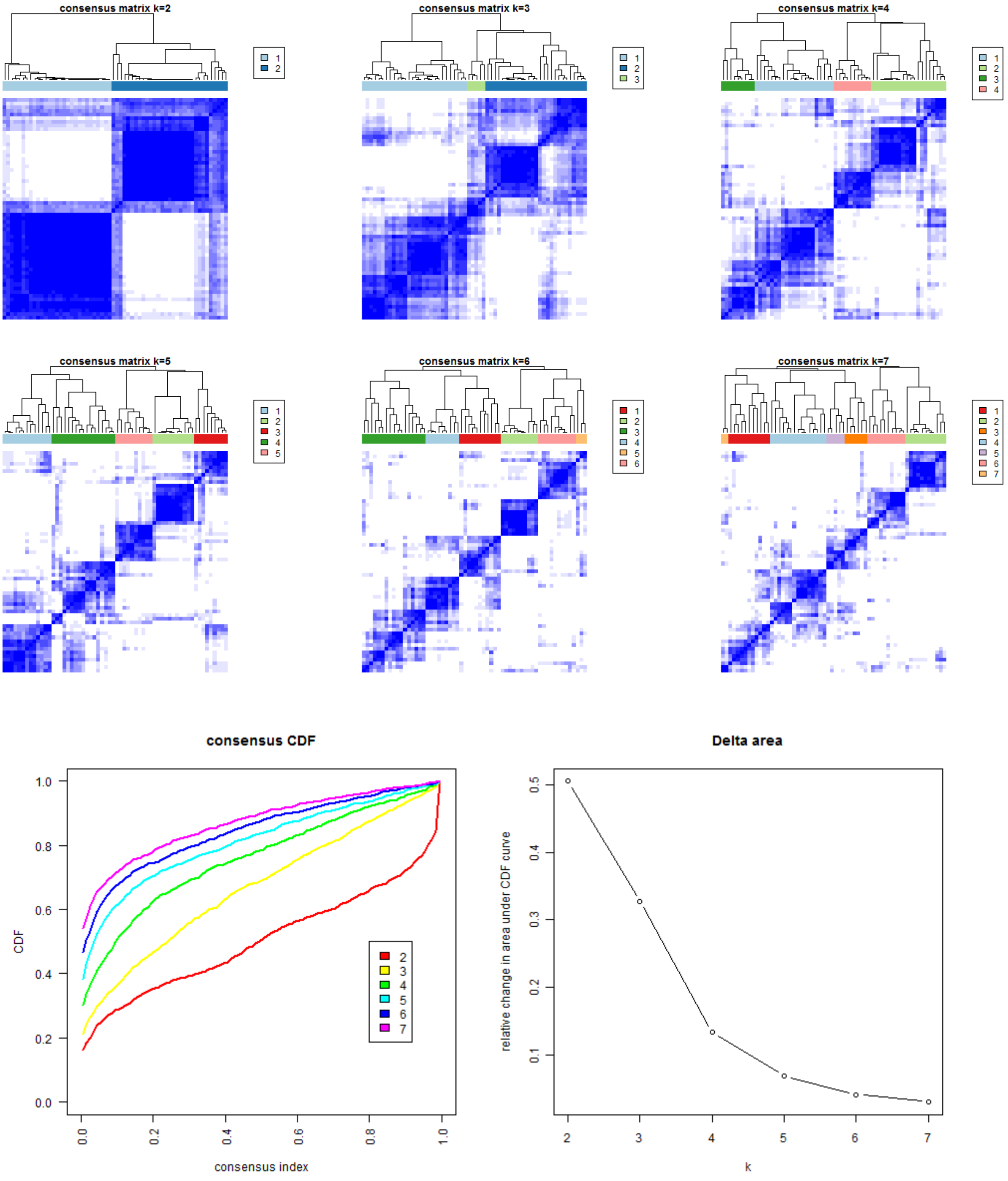

Figure 61: Statistical significance of CCSS clustering (Consensus clustering)

8.4.4 Statistical significance

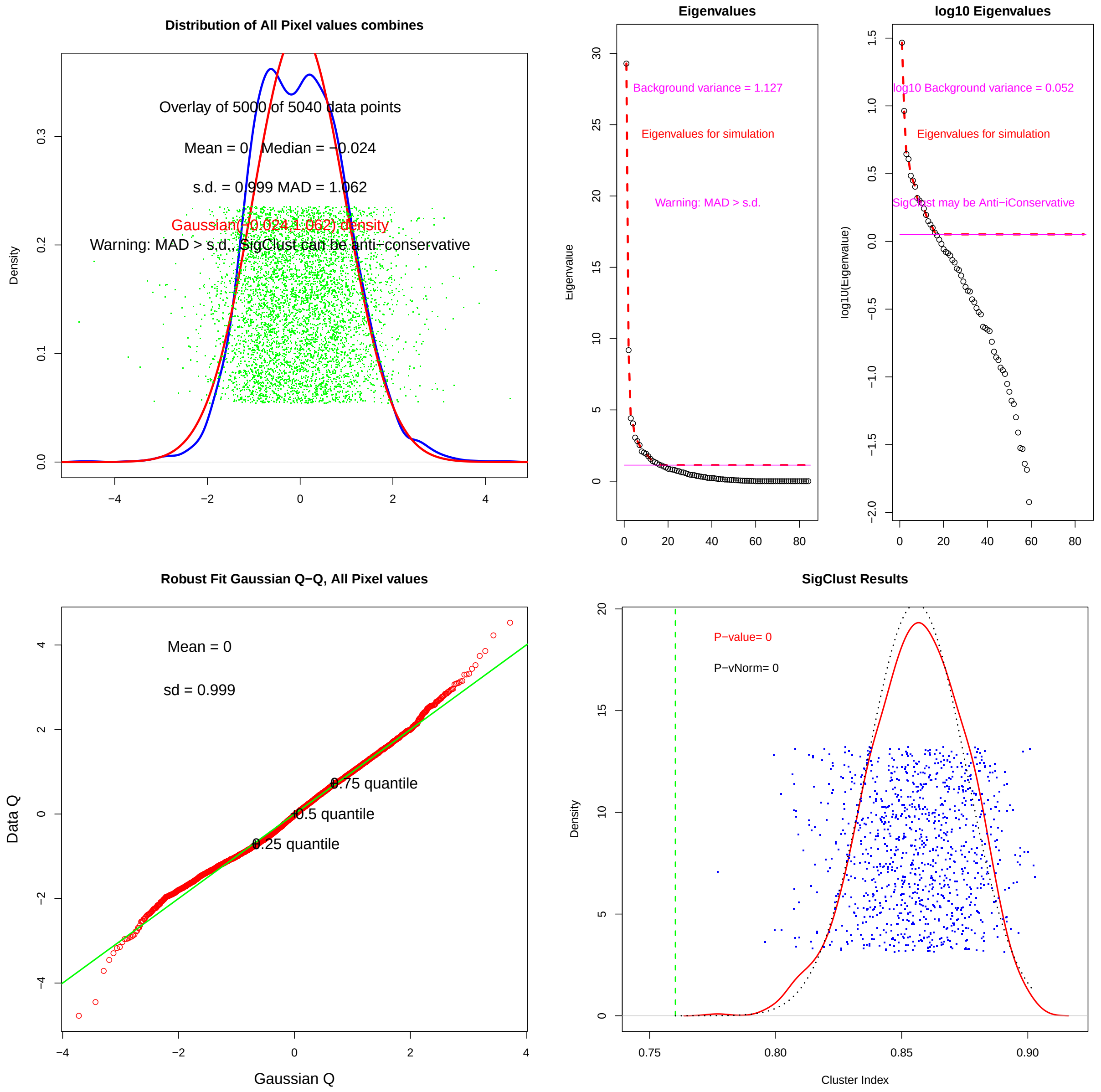

P-value	P-vNorm
0.0E+00	6.1E-07

Table 49: SigClust p-values

8.4.5 Cluster-wise assessment of cluster stability

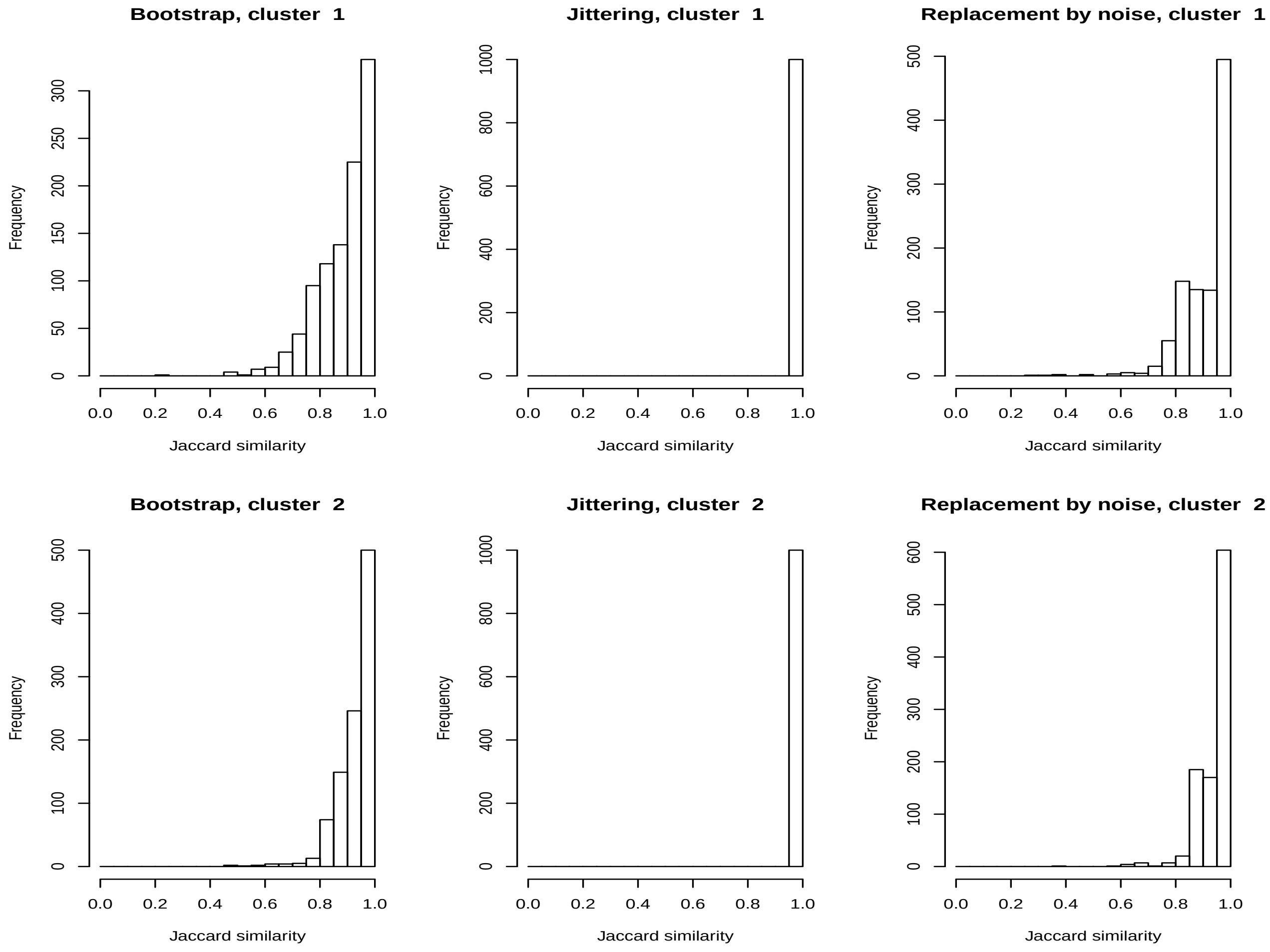


```
* Cluster stability assessment *
Cluster method:  kmeans
Full clustering results are given as parameter result
of the clusterboot object, which also provides further statistics
of the resampling results.
Number of resampling runs:  1000

Number of clusters found in data:  2

 Clusterwise Jaccard bootstrap mean:
[1] 0.8940155 0.9342179
dissolved:
[1] 5 2
recovered:
[1] 909 982
 Clusterwise Jaccard jittering mean:
[1] 0.9997000 0.9998364
dissolved:
[1] 0 0
recovered:
```

```
[1] 1000 1000
 Clusterwise Jaccard replacement by noise mean:
[1] 0.9112561 0.9445279
dissolved:
[1] 6 1
recovered:
[1] 967 986
```

Removing one sample

```
   Min. 1st Qu.  Median    Mean 3rd Qu.    Max.
 0.3220  0.3220  0.3390  0.3333  0.3390  0.3390
```

Removing one gene

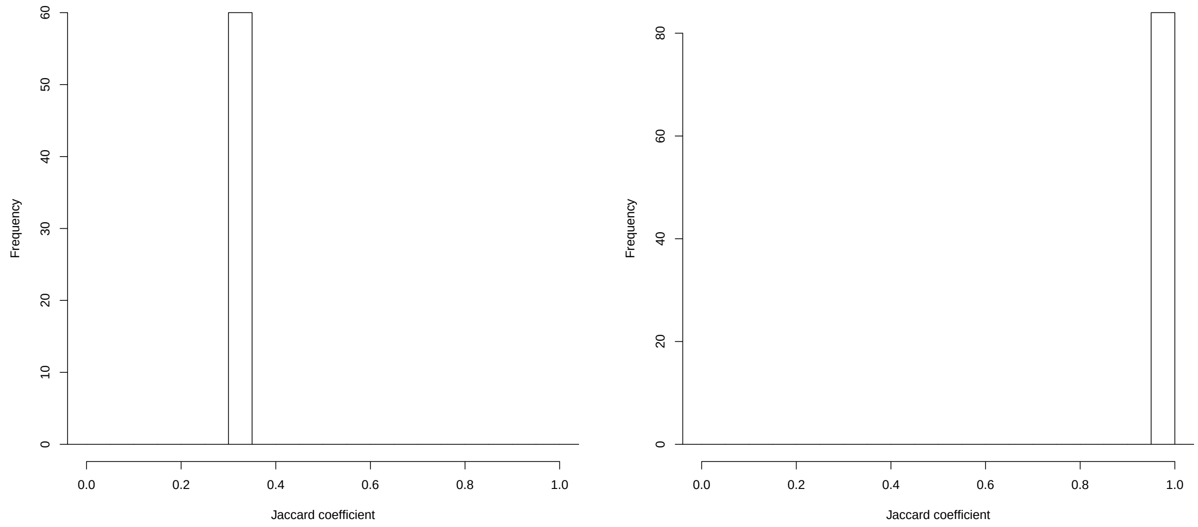

Figure 62: Removing one sample (left) and one gene (right) at a time: distributions of Jaccard coefficients

```
   Min. 1st Qu.  Median    Mean 3rd Qu.    Max.
      1       1       1       1       1       1

      APN                 AD                ADM                FOM
 Min.   :0.1986   Min.   :11.39   Min.   :2.22   Min.   :0.6757
 1st Qu.:0.1986   1st Qu.:11.39   1st Qu.:2.22   1st Qu.:0.8271
 Median :0.1986   Median :11.39   Median :2.22   Median :0.8893
 Mean   :0.1986   Mean   :11.39   Mean   :2.22   Mean   :0.8765
 3rd Qu.:0.1986   3rd Qu.:11.39   3rd Qu.:2.22   3rd Qu.:0.9376
 Max.   :0.1986   Max.   :11.39   Max.   :2.22   Max.   :1.0003
```

Removing sets of k genes

```
   Min. 1st Qu.  Median    Mean 3rd Qu.    Max.
 0.3824  0.6000  0.6500  0.6647  0.7500  1.0000
```

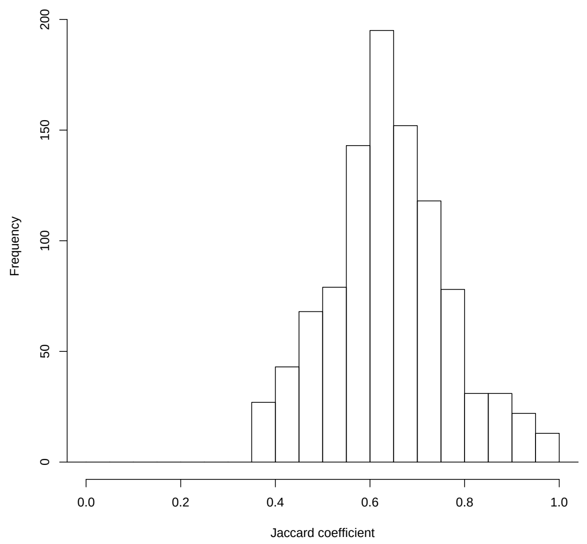

Figure 63: Removing two thirds of the genes: distribution of Jaccard coefficients

8.4.6 Validation measures

```
Clustering Methods:
 kmeans

Cluster sizes:
 2 3 4 5 6

Validation Measures:
                              2       3       4       5       6

kmeans APN            0.0000  0.0148  0.1365  0.3117  0.2067
       AD             0.9662  0.8833  0.8672  0.8641  0.8125
       ADM            0.0000  0.1591  1.2190  2.4579  1.6711
       FOM            0.8765  0.8270  0.8155  0.7987  0.7874
       Connectivity  56.7460 69.4218 86.0329 80.4929 94.1976
       Dunn           0.3129  0.3448  0.3436  0.3651  0.3436
       Silhouette     0.0302  0.0689  0.0478  0.0589  0.0533

Optimal Scores:

             Score   Method Clusters
APN           0.0000 kmeans 2
AD            0.8125 kmeans 6
ADM           0.0000 kmeans 2
FOM           0.7874 kmeans 6
Connectivity 56.7460 kmeans 2
Dunn          0.3651 kmeans 5
Silhouette    0.0689 kmeans 3
```

9 Boersma et al. (2008) (microdissected stromal samples)

9.1 LAS bicluster

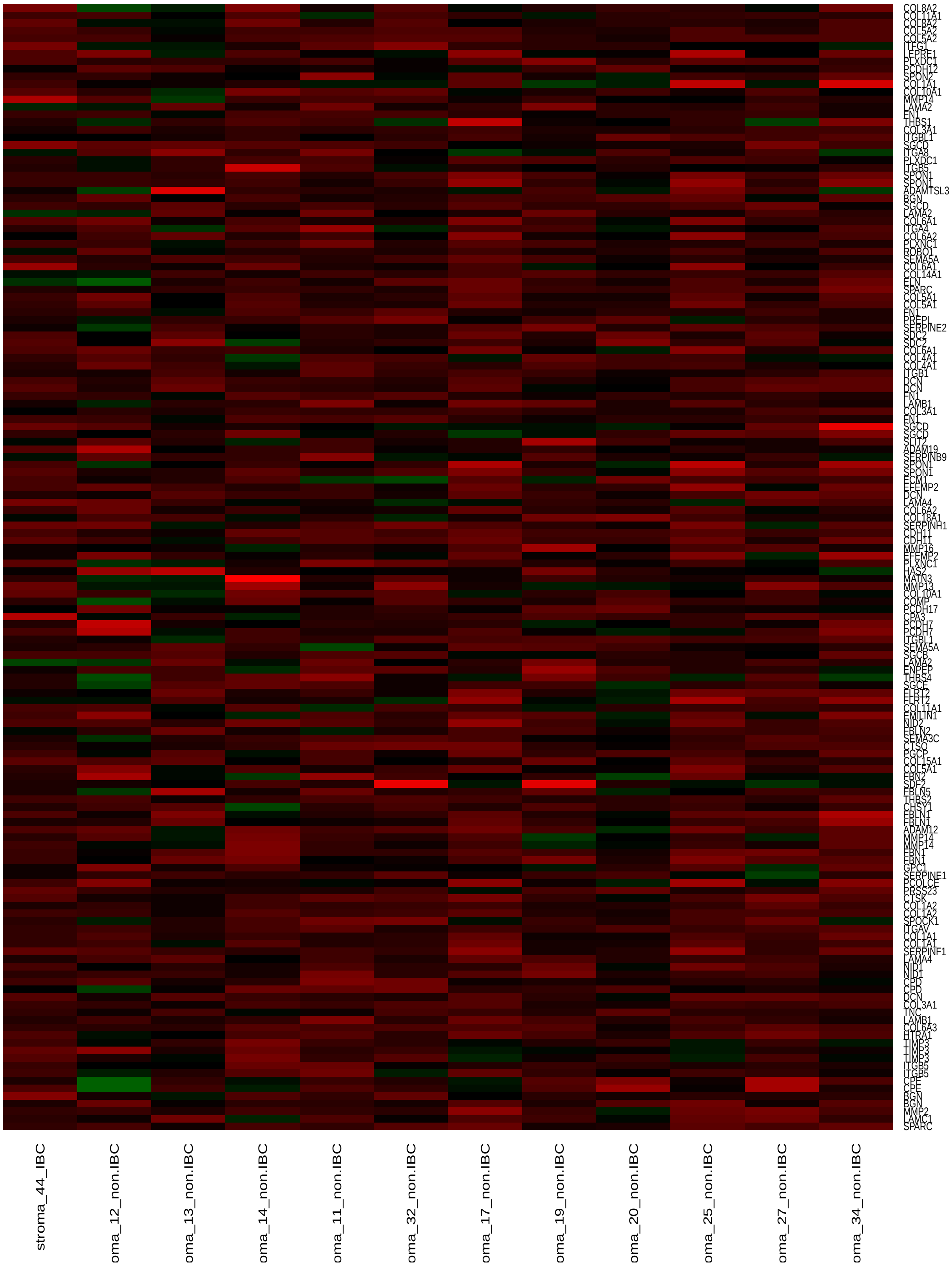

Figure 64: Heatmap of the LAS bicluster

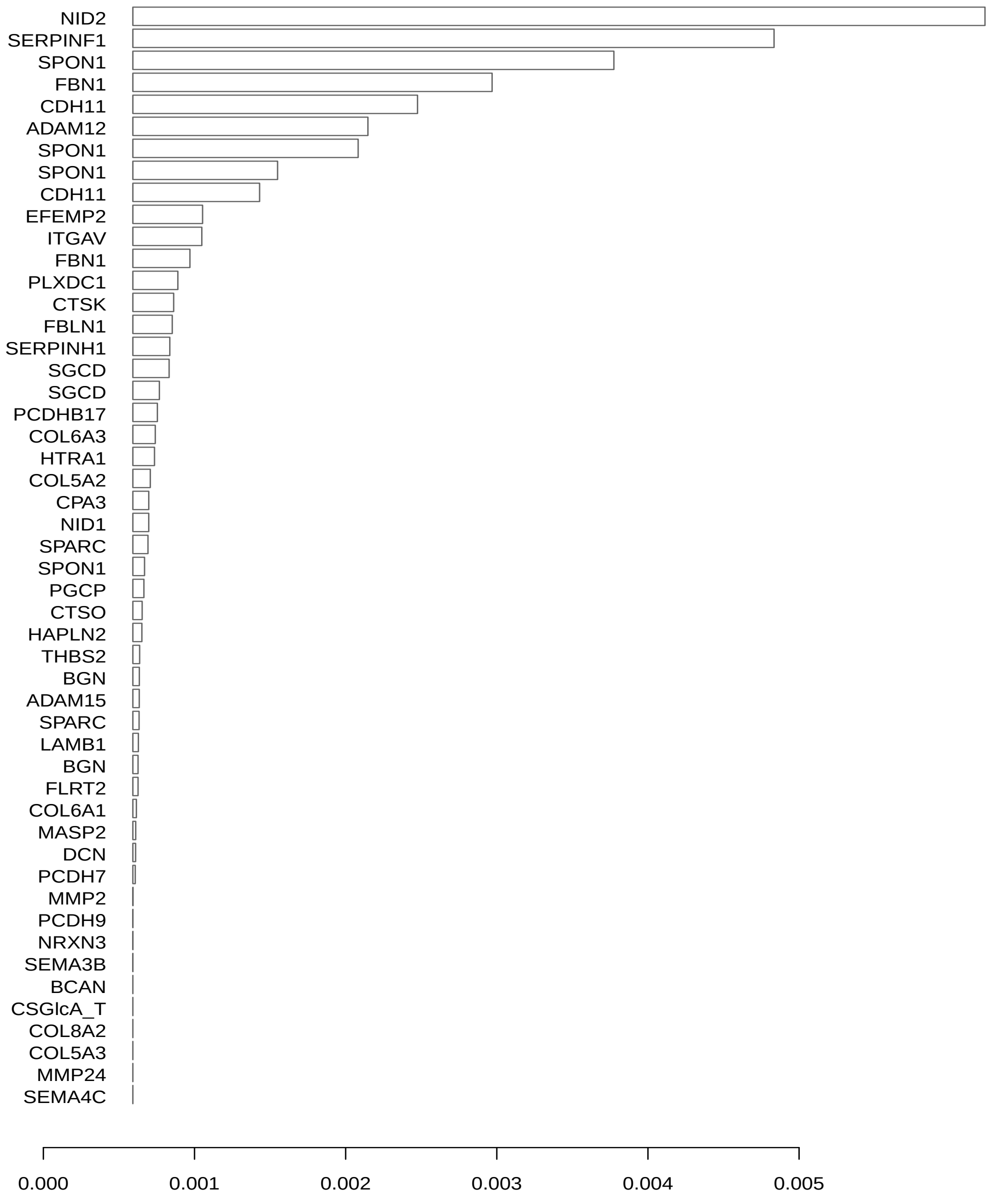

Figure 65: Most informative genes for the LAS bicluster

```
Number of informative genes of the LAS bicluster that also belong
to the ECM3 consensus list: 32 of 34 (94%)
```

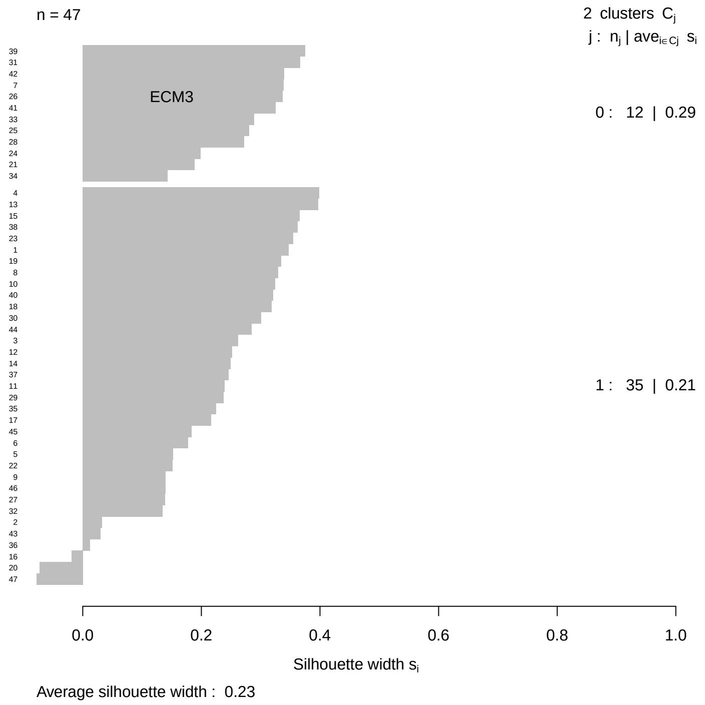

Figure 66: Silhoutte plot for the LAS bicluster

Connectivity	Dunn Index
8.98	0.48

Table 50: Connectivity validation measure and Dunn Index of LAS partitioning

9.2 IRCC-KM bicluster

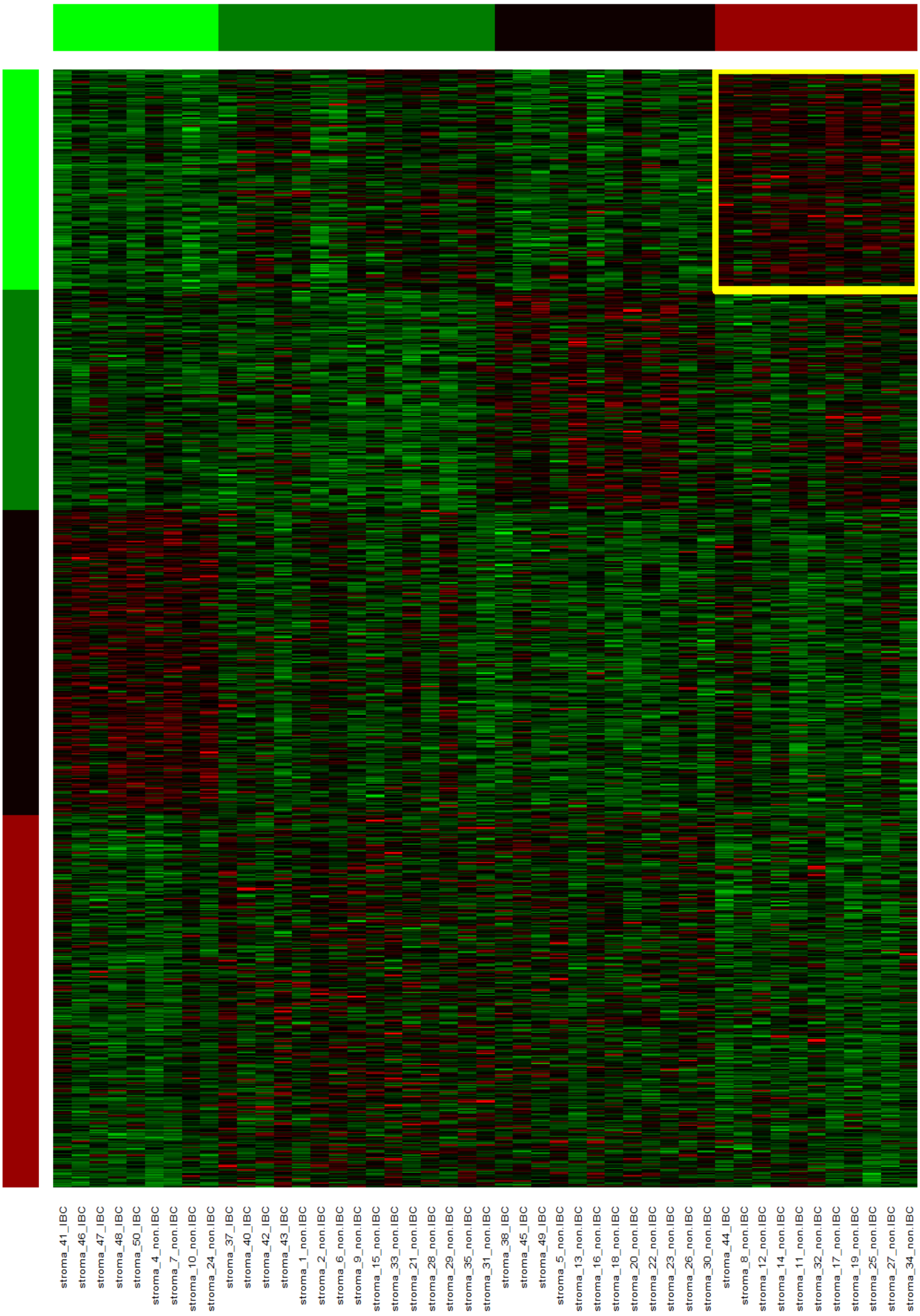

Figure 67: Heatmap of the IRCC-KM bicluster

IRCC-KM genes

MMP14	SPARC	CTSD	CTSB	CTSB	CD9
MMP2	BGN	BGN	THBS1	THBS1	THBS1
THBS1	ITGB5	ITGB5	TIMP3	TIMP3	TIMP3
TIMP3	HTRA1	COL6A3	CTSC	LAMB1	TNC
TIMP1	COL3A1	DCN	CPD	CPD	ALCAM
ALCAM	NID1	NID1	CHPF	SERPINF1	COL1A1
COL1A1	ITGAV	SPOCK1	ADAM9	COL1A2	COL1A2
CTSK	PRSS23	PCOLCE	ADAM10	SERPINE1	SERPINE1
GPC1	FBN1	FBN1	ITGB2	MMP14	MMP14
CTSS	CTSS	ADAM12	FBLN1	THBS2	SDF2
TIMP2	FBN2	TSPAN31	COL5A1	PGCP	CTSO
SEMA3C	SEMA3C	MMP11	MMP11	MMP9	NID2
EMILIN1	COL11A1	MMP1	MMP12	ITGB3	GPC4
GPC4	ITGAE	SGCB	ITGBL1	PCDH7	PCDH7
CPA3	COMP	ITGAM	MMP3	ITGA4	COL10A1
MMP13	MATN3	PLXNC1	EFEMP2	CDH11	CDH11
SIGLEC7	SERPINH1	CD164	PGCP	CD164	COL6A2
CTSZ	DCN	EFEMP2	ECM1	SPON1	SPON1
SERPINB9	ADAM19	CD300A	ITGAX	SGCD	SGCD
FN1	COL3A1	COL13A1	DPP4	FN1	DCN
NRP2	DCN	ITGB1	COL6A1	SDC2	SDC2
SDC2	FN1	COL5A1	COL5A1	SPARC	COL6A1
ROBO1	PLXNC1	CTSB	CTSB	COL6A2	ITGA4
COL6A1	SGCD	SERPINB1	BGN	SPON1	SPON1
ITGB5	ITGB5	PLXDC1	ADAMTS2	SGCD	NRP2
FN1	FN1	ITGBL1	APP	COL3A1	ADAM12
FBN2	THBS1	FN1	MMP14	COL10A1	COL1A1
SPG21	SPON2	CHST12	CHST11	PLXDC1	CORIN
KLK14	LEPRE1	ITFG1	COL5A2	COL5A2	CSGlcA_T
COL8A2	COL11A1	COL8A2	CSGlcA_T		

IRCC-KM samples

stroma_44_IB stroma_8_non stroma_12_no stroma_14_no stroma_11_no stroma_32_no stroma_17_no stroma_19_no stroma_25_no stroma_27_no stroma_34_no

9.2.1 Comparing LAS and IRCC-KM biclusters

	No ECM	ECM3
No ECM3	689	36
ECM3	66	112
Jaccard similarity	0.52	

Table 51: Comparing gene lists: LAS (column) vs. IRCC-KM bicluster (row)

	No ECM	ECM3
No ECM3	34	2
ECM3	1	10
Jaccard similarity	0.77	

Table 52: Comparing sample lists: LAS (column) vs. IRCC-KM bicluster (row)

9.3 IRCC-HC bicluster

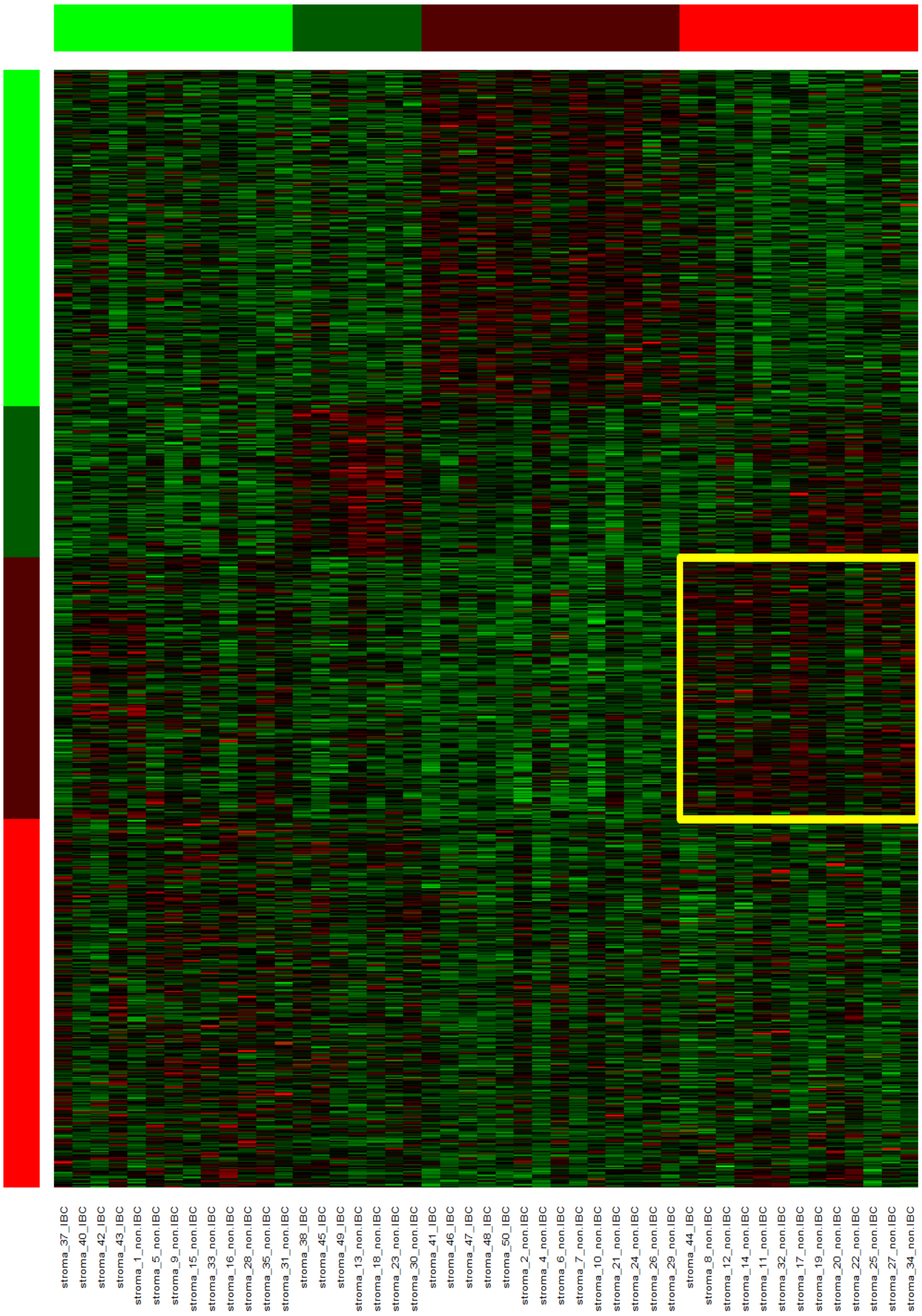

Figure 68: Heatmap of the IRCC-HC bicluster

IRCC-HC genes

ADAM10	ADAM10	ADAM12	ADAM8	ADAM9	ADAMDEC1
ADAMTS2	ADRM1	BGN	BGN	BGN	BST1
CD164	CD164	CD164	CD300A	CD300A	CD44
CD44	CD44	CD44	CD44	CD44	CD44
CDH11	CDH11	CHPF	CHST11	CHST7	COL10A1
COL10A1	COL11A1	COL11A1	COL1A1	COL1A1	COL1A1
COL1A2	COL1A2	COL3A1	COL3A1	COL3A1	COL4A3BP
COL5A1	COL5A1	COL5A1	COL5A2	COL5A2	COL6A1
COL6A1	COL6A1	COL6A2	COL6A2	COL6A3	COL7A1
COL8A1	COL8A2	COL8A2	COMP	CORIN	CPA3
CPD	CPM	CSGlcA_T	CSGlcA_T	CTSB	CTSB
CTSB	CTSB	CTSC	CTSD	CTSF	CTSH
CTSK	CTSS	CTSS	CTSZ	DCN	DCN
DCN	DCN	DPP4	DPP4	DPP4	ECM1
EFEMP2	EFEMP2	EMILIN1	FBLN1	FBLN1	FBN1
FBN1	FCN1	FN1	FN1	FN1	FN1
FN1	FN1	GPC4	GPC4	HTRA1	ITGA3
ITGA5	ITGAE	ITGAL	ITGAM	ITGAV	ITGAX
ITGB1	ITGB1BP1	ITGB2	ITGB3	ITGB3	ITGB5
ITGB5	ITGB5	ITGB5	ITGB7	ITGBL1	ITGBL1
LAMA4	LAMB1	LAMB1	LAMB2	LEPRE1	MATN3
MME	MME	MMP1	MMP11	MMP12	MMP13
MMP14	MMP14	MMP14	MMP19	MMP19	MMP2
MMP9	NID1	NID1	NID2	NRP1	NRP2
NRP2	PCDH7	PCDH7	PCOLCE	PGCP	PGCP
PLXDC1	PLXDC1	PLXNA1	PLXNA2	PLXNB2	PLXNC1
PLXNC1	PLXNC1	PLXND1	PLXND1	PREP	PREP
PRG2	PRSS23	PSMC4	SDC2	SDC2	SDC2
SELPLG	SEMA3C	SERPINB1	SERPINB1	SERPINE1	SERPINE1
SERPINF1	SERPINH1	SGCD	SGCD	SGCD	SGCD
SIGLEC7	SPARC	SPARC	SPOCK1	SPON1	SPON1
SPON1	SPON1	SPON2	TGM2	THBS1	THBS1
THBS1	THBS1	THBS1	THBS2	THBS3	TIMP1
TIMP2	TIMP3	TIMP3	TIMP3	TIMP3	TSPAN2
TSPAN3	TSPAN3	TSPAN31	TSPAN31	TSPAN4	TSPAN4
XPNPEP2					

IRCC-HC samples

stroma_44_IB stroma_8_non stroma_12_no stroma_14_no stroma_11_no stroma_32_no stroma_17_no stroma_19_no stroma_20_no stroma_22_no stroma_25_no stroma_27_no stroma_34_no

9.3.1 Comparing LAS and IRCC-HC biclusters

	No ECM	ECM3
No ECM3	647	45
ECM3	108	103
Jaccard similarity	0.40	

Table 53: Comparing gene lists: LAS (column) vs. IRCC-HC bicluster (row)

	No ECM	ECM3
No ECM3	33	1
ECM3	2	11
Jaccard similarity	0.79	

Table 54: Comparing sample lists: LAS (column) vs. IRCC-HC bicluster (row)

9.4 CCSS bicluster

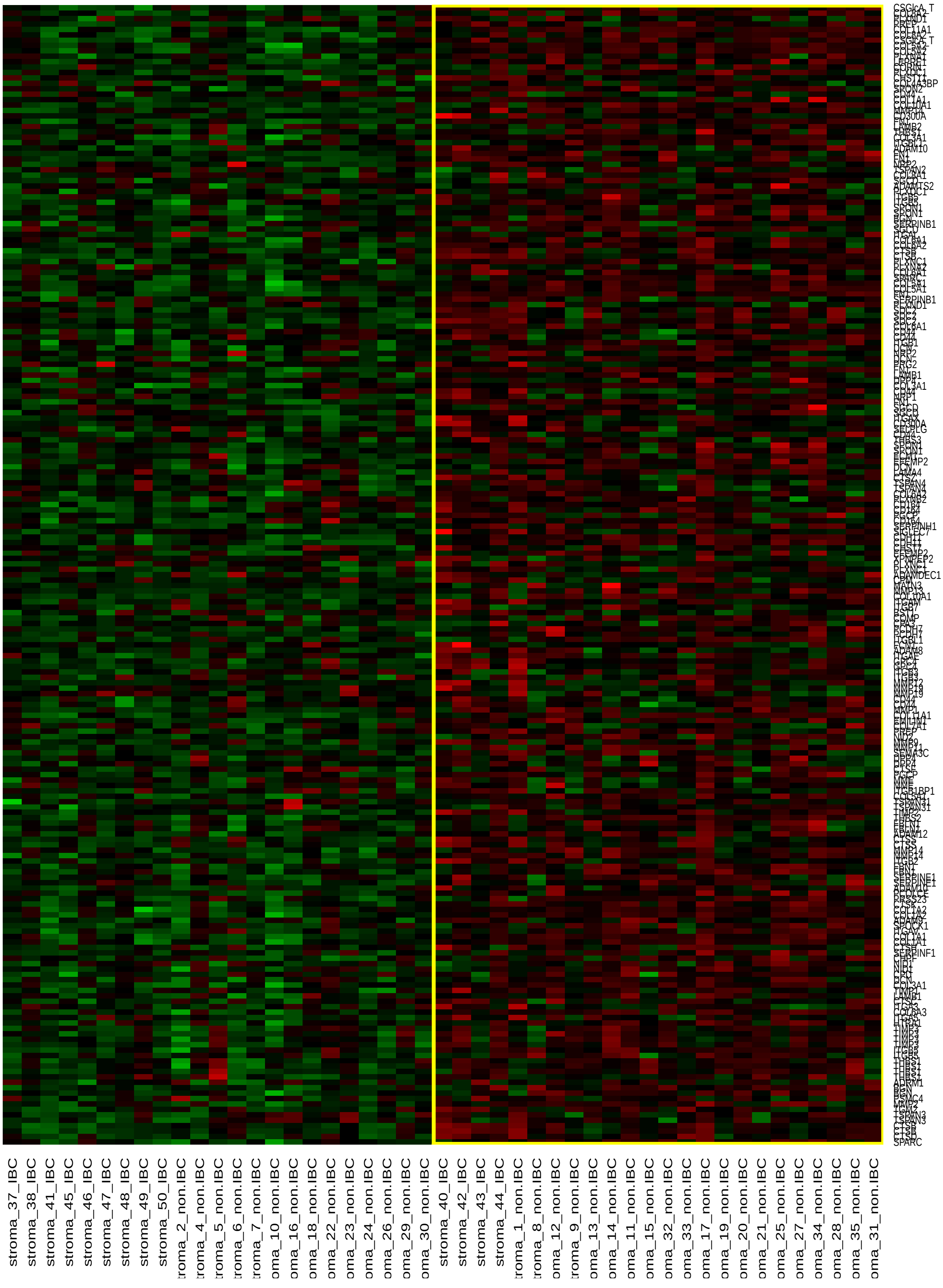

Figure 69: Heatmap of the CCSS bicluster

9.4.1 Comparing LAS and CCSS biclusters

	No ECM	ECM3
No ECM3	23	0
ECM3	12	12
Jaccard similarity	0.50	

Table 55: Comparing sample lists: LAS (column) vs. CCSS (row) biclusters

9.4.2 Prediction strength for CCSS

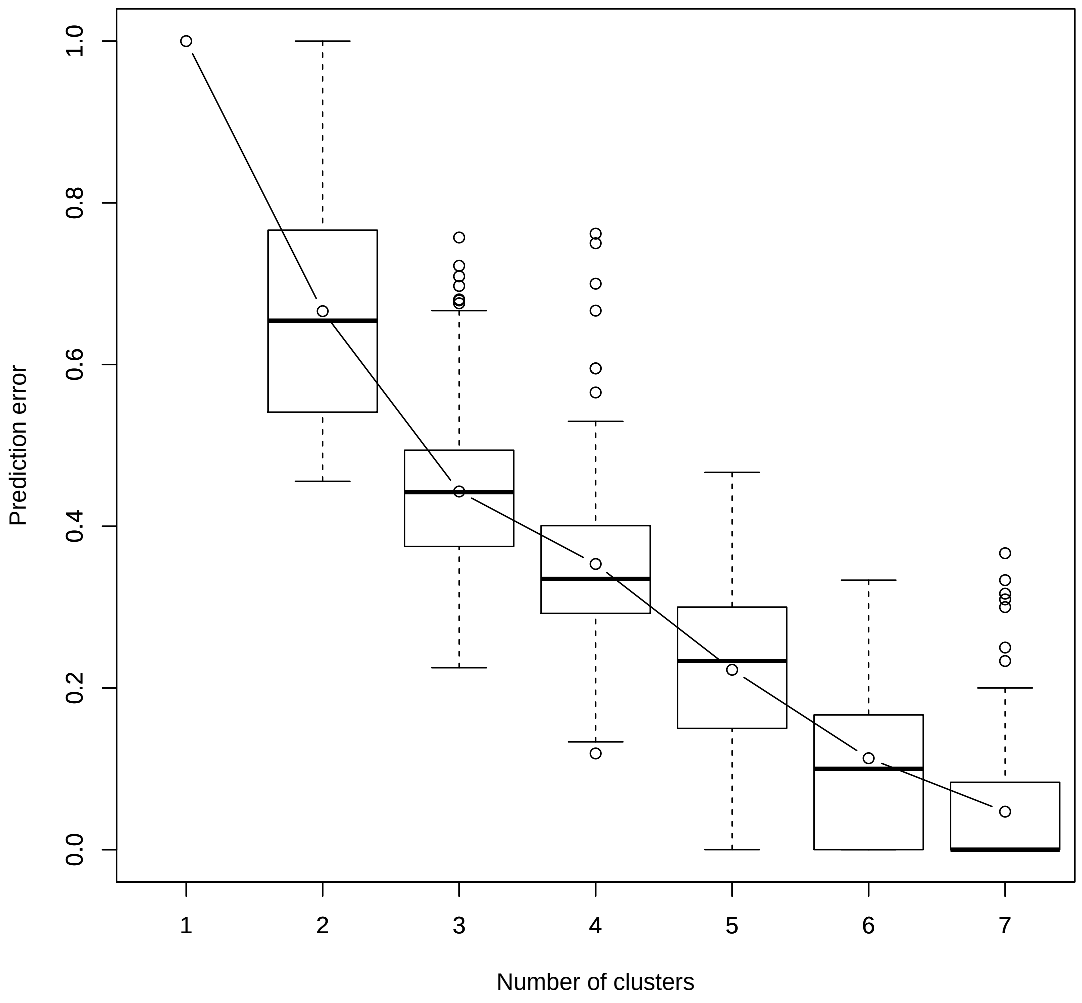

9.4.3 Consensus clustering

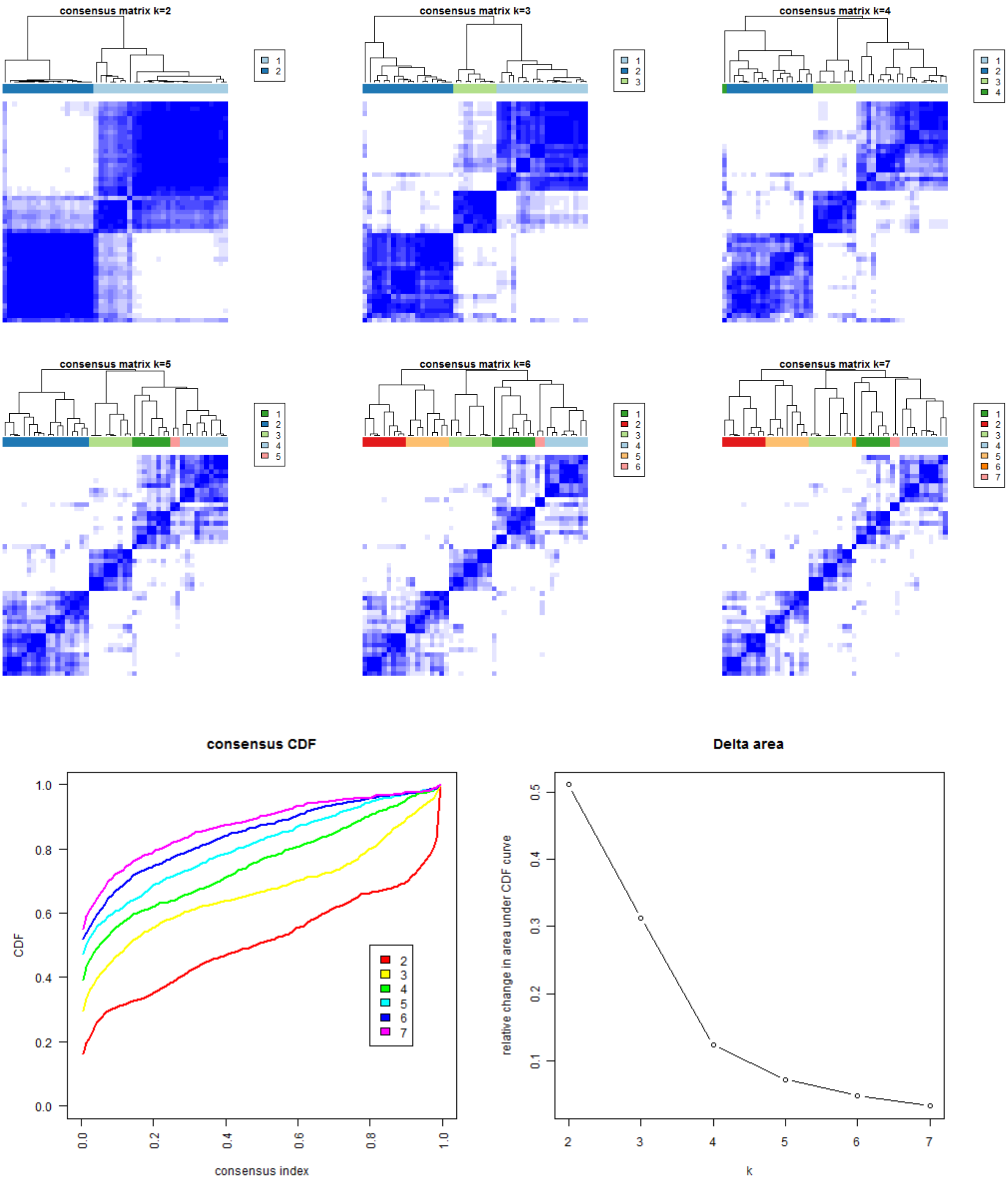

Figure 70: Statistical significance of CCSS clustering (Consensus clustering)

9.4.4 Statistical significance

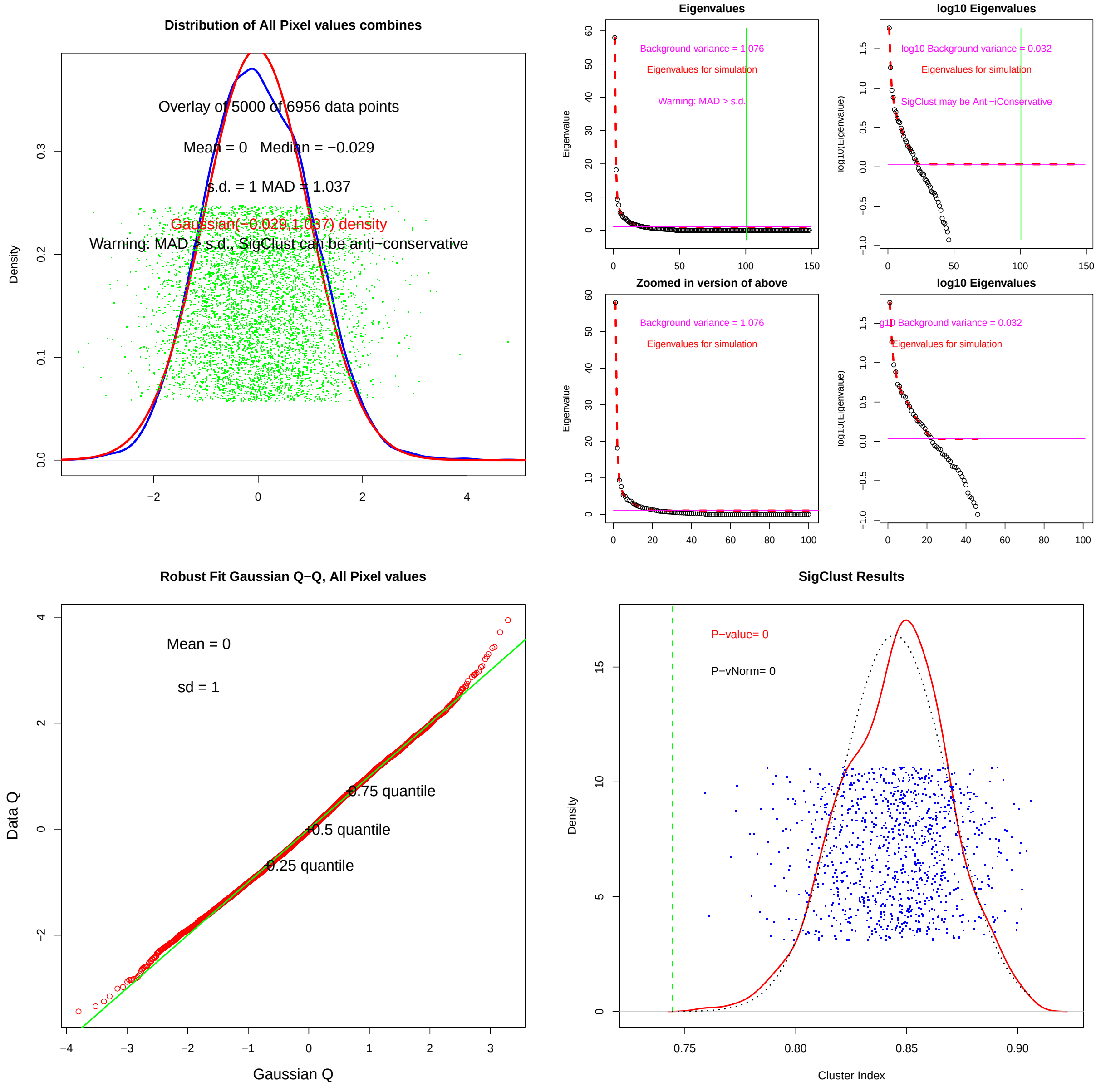

P-value	P-vNorm
0.0E+00	2.0E-05

Table 56: SigClust p-values

9.4.5 Cluster-wise assessment of cluster stability

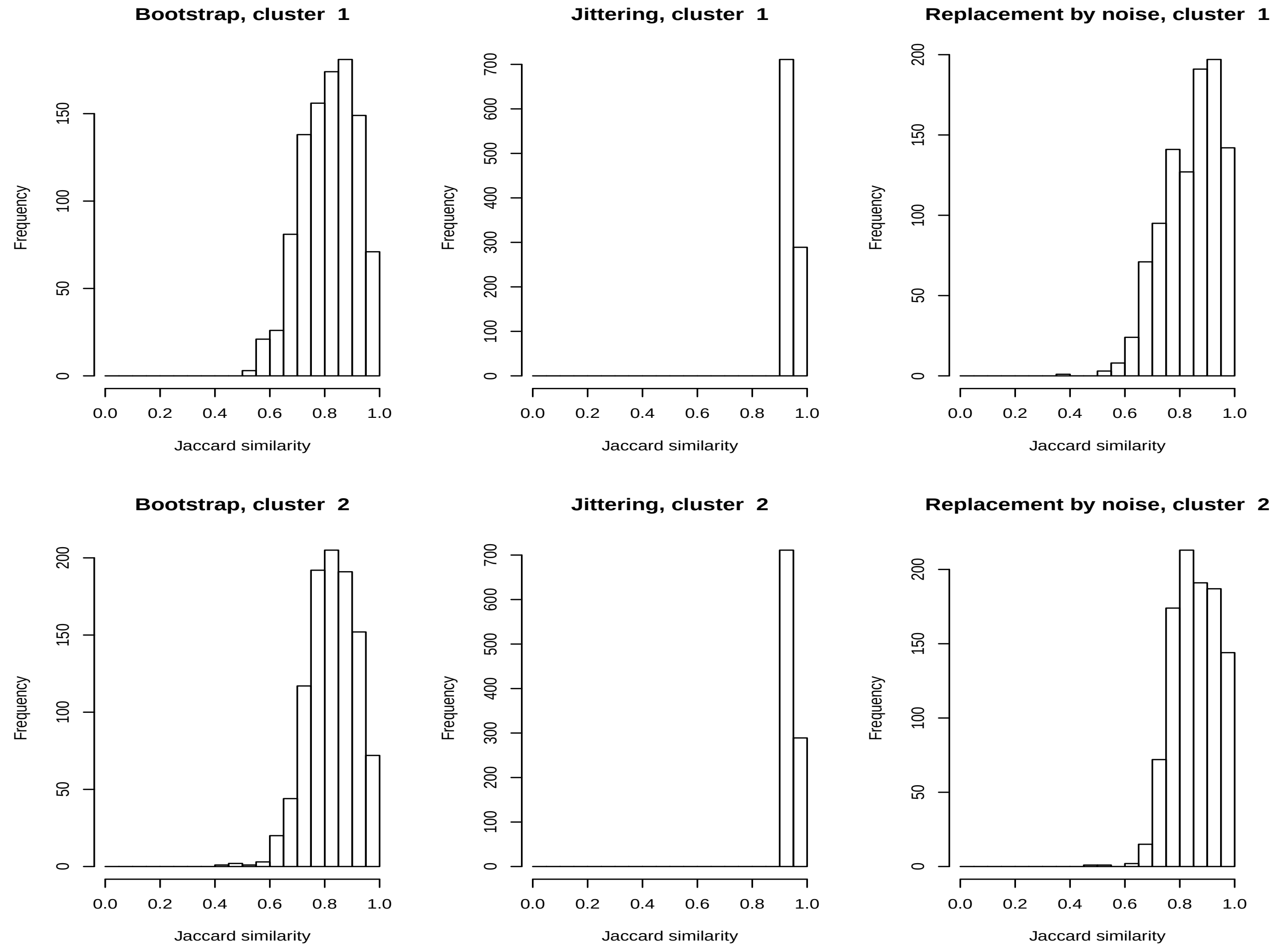


```
* Cluster stability assessment *
Cluster method:  kmeans
Full clustering results are given as parameter result
of the clusterboot object, which also provides further statistics
of the resampling results.
Number of resampling runs:  1000

Number of clusters found in data:  2

 Clusterwise Jaccard bootstrap mean:
[1] 0.8155460 0.8279897
dissolved:
[1] 0 3
recovered:
[1] 731 812
 Clusterwise Jaccard jittering mean:
[1] 0.9613784 0.9633618
dissolved:
[1] 0 0
recovered:
```

```
[1] 1000 1000
 Clusterwise Jaccard replacement by noise mean:
[1] 0.8409090 0.8565614
dissolved:
[1] 1 1
recovered:
[1] 798 909
```

Removing one sample

```
   Min. 1st Qu.  Median    Mean 3rd Qu.    Max.
 0.9412  0.9444  1.0000  0.9795  1.0000  1.0000
```

Removing one gene

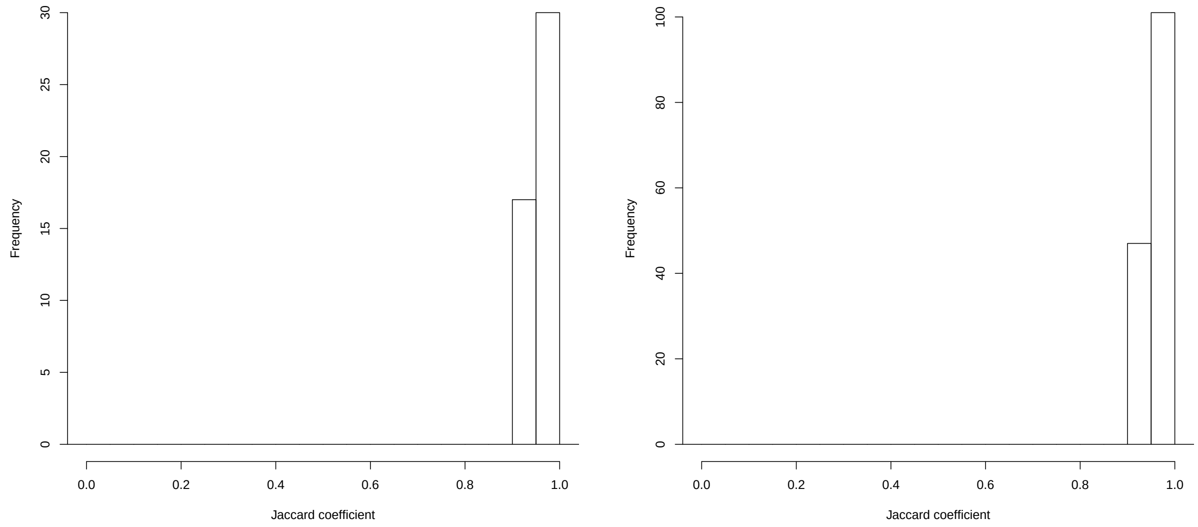

Figure 71: Removing one sample (left) and one gene (right) at a time: distributions of Jaccard coefficients

```
   Min. 1st Qu.  Median    Mean 3rd Qu.    Max.
 0.9444  0.9444  1.0000  0.9824  1.0000  1.0000
```

```
      APN                AD              ADM               FOM
 Min.   :0.1824   Min.   :14.82   Min.   :2.759   Min.   :0.6602
 1st Qu.:0.1824   1st Qu.:14.82   1st Qu.:2.759   1st Qu.:0.8317
 Median :0.2116   Median :14.90   Median :3.116   Median :0.8855
 Mean   :0.2023   Mean   :14.87   Mean   :3.003   Mean   :0.8784
 3rd Qu.:0.2116   3rd Qu.:14.90   3rd Qu.:3.116   3rd Qu.:0.9403
 Max.   :0.2116   Max.   :14.90   Max.   :3.116   Max.   :1.0012
```

Removing sets of k genes

```
   Min. 1st Qu.  Median    Mean 3rd Qu.    Max.
 0.5769  0.8333  0.8889  0.8788  0.9444  1.0000
```

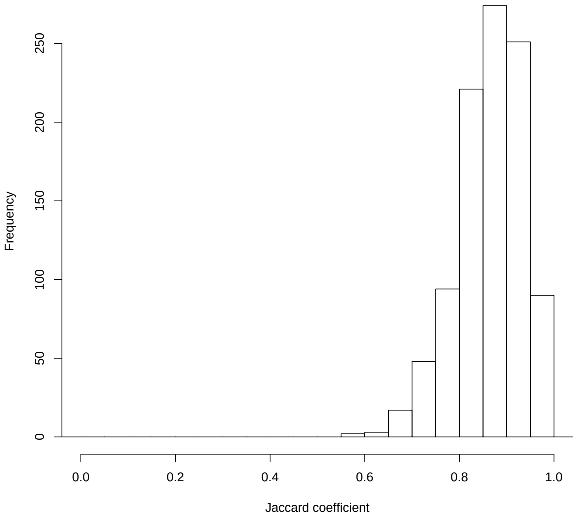

Figure 72: Removing two thirds of the genes: distribution of Jaccard coefficients

9.4.6 Validation measures

```
Clustering Methods:
 kmeans

Cluster sizes:
 2 3 4 5 6

Validation Measures:
                                2       3       4       5       6

kmeans APN             0.0128  0.0110  0.0357  0.0391  0.0821
       AD              0.9731  0.8851  0.8341  0.7806  0.7606
       ADM             0.2520  0.1921  0.4987  0.5343  1.0879
       FOM             0.8784  0.8298  0.7906  0.7779  0.7580
       Connectivity   57.8988 65.3385 68.7929 71.1440 74.5341
       Dunn            0.2775  0.2785  0.3473  0.3473  0.3438
       Silhouette      0.0093  0.0402  0.0361  0.0498  0.0381

Optimal Scores:

             Score   Method Clusters
APN           0.0110 kmeans 3
AD            0.7606 kmeans 6
ADM           0.1921 kmeans 3
FOM           0.7580 kmeans 6
Connectivity 57.8988 kmeans 2
Dunn          0.3473 kmeans 4
Silhouette    0.0498 kmeans 5
```

10 Boersma et al. (2008) (microdissected tumor cell samples)

10.1 LAS bicluster

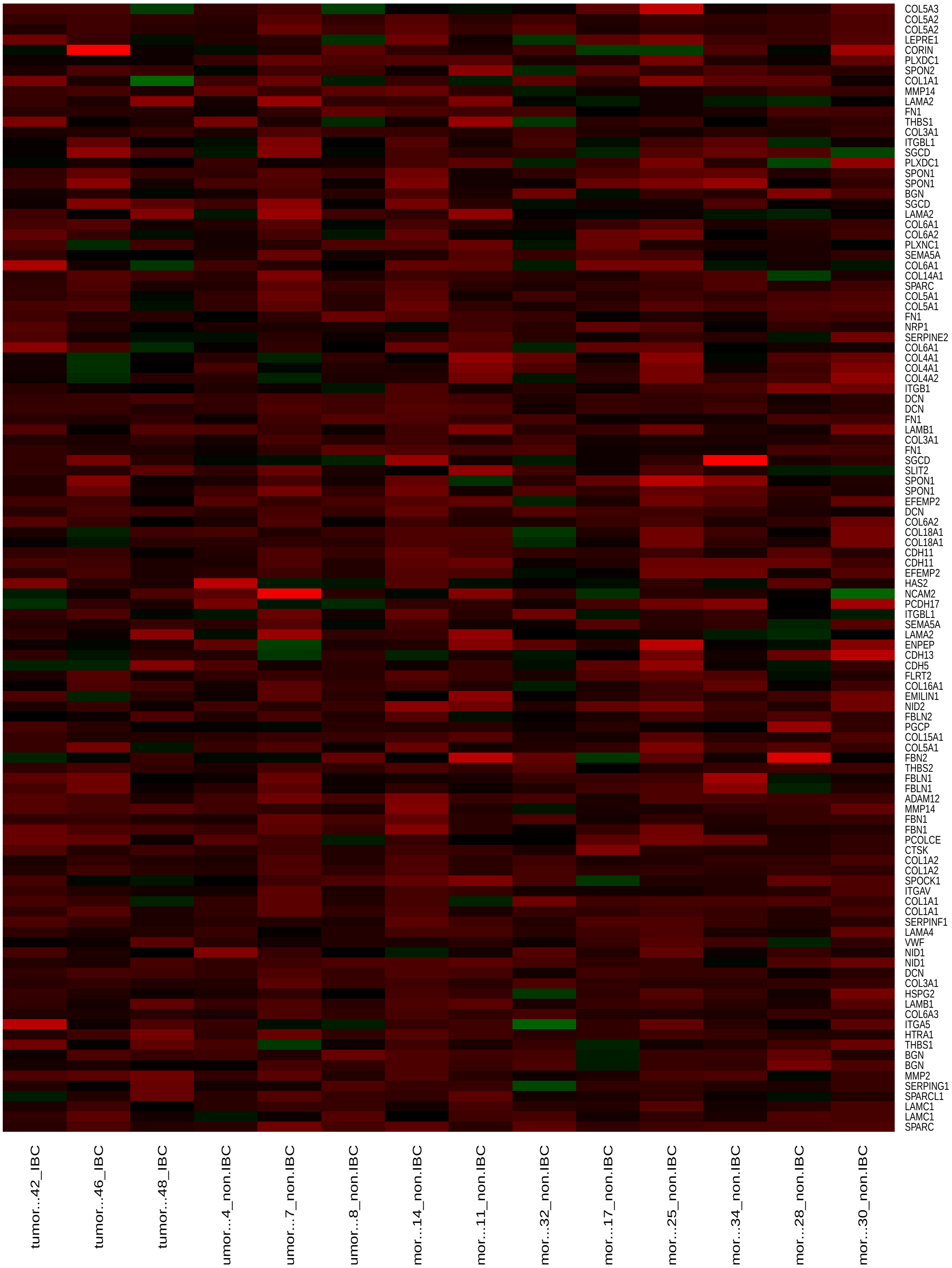

Figure 73: Heatmap of the LAS bicluster

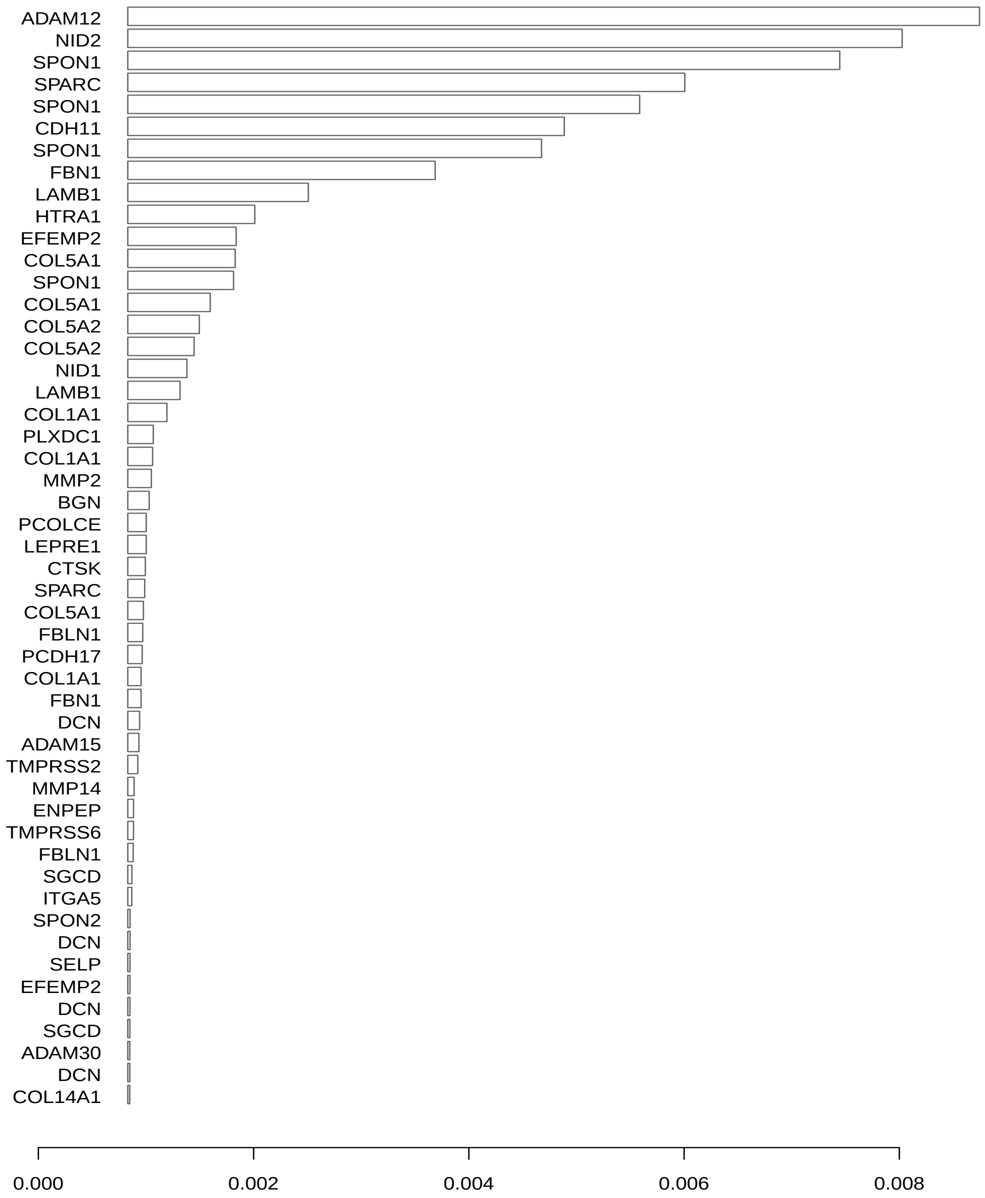

Figure 74: Most informative genes for the LAS bicluster

```
Number of informative genes of the LAS bicluster that also belong
to the ECM3 consensus list: 28 of 34 (82%)
```

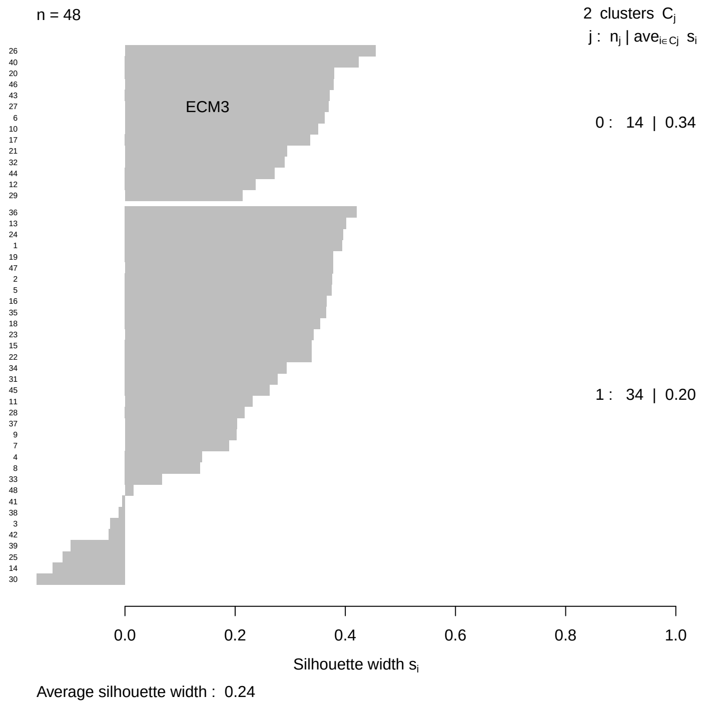

Figure 75: Silhoutte plot for the LAS bicluster

Connectivity	Dunn Index
11.62	0.34

Table 57: Connectivity validation measure and Dunn Index of LAS partitioning

10.2 IRCC-KM bicluster

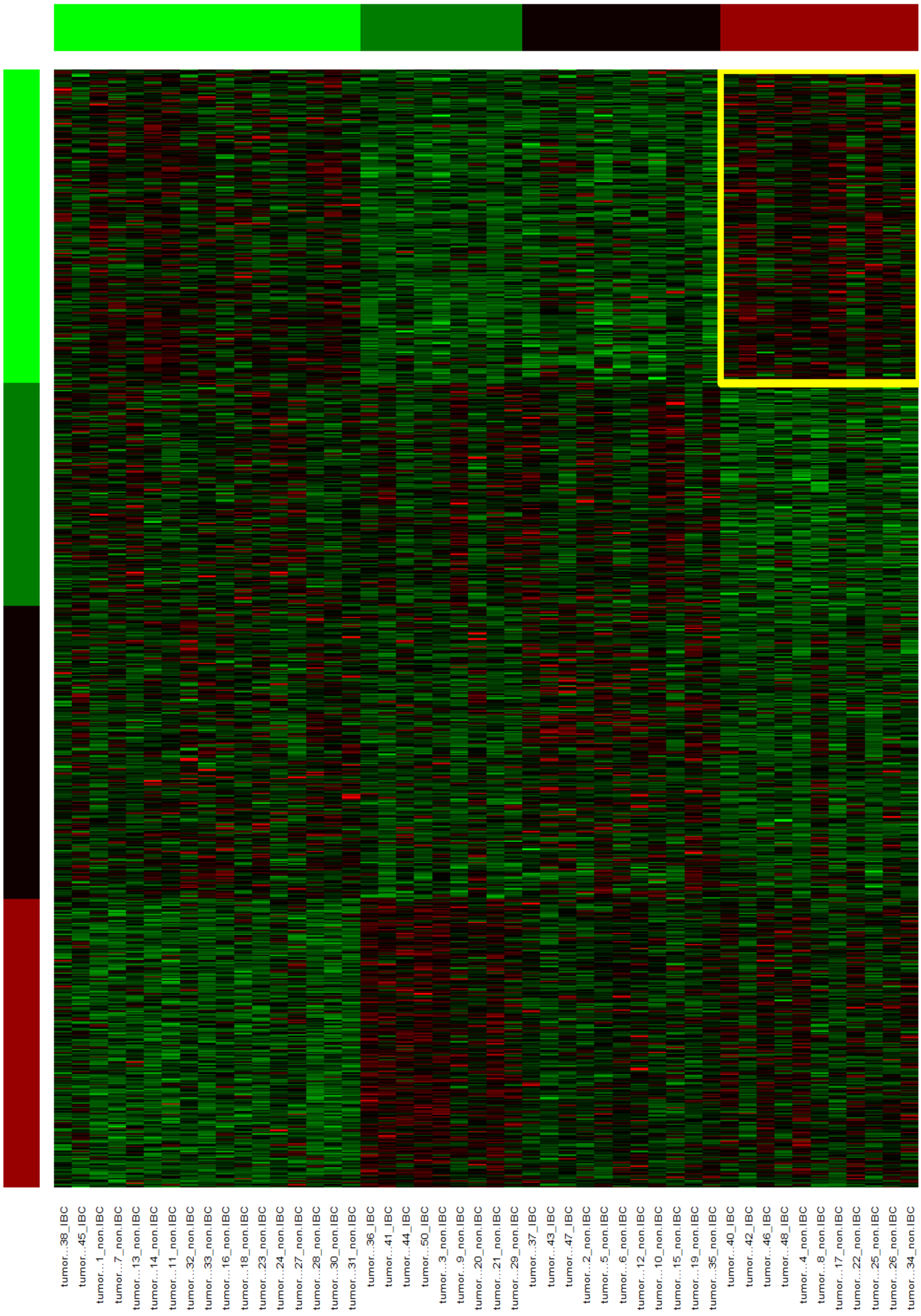

Figure 76: Heatmap of the IRCC-KM bicluster

IRCC-KM genes

SPARC	CTSD	LAMC1	LAMC1	SPARCL1	CTSB
CTSB	SERPING1	TGM2	MMP2	BGN	BGN
THBS1	THBS1	CPE	ITGB5	ITGB5	TIMP3
TIMP3	TIMP3	TIMP3	HTRA1	ITGA5	COL6A3
CTSC	LAMB1	TNC	HSPG2	HSPG2	TIMP1
EFEMP1	COL3A1	DCN	CPD	NID1	NID1
VWF	LAMA4	TSPAN7	SERPINF1	STAG1	CTSH
COL1A1	COL1A1	MATN2	ITGAV	SPOCK1	COL1A2
COL1A2	CTSK	PCOLCE	SERPINE1	SERPINE1	FBN1
FBN1	ITGB2	MMP14	CTSS	CTSS	ADAM12
FBLN1	FBLN1	CHSY1	THBS2	FBLN5	TIMP2
COL5A1	ITGB1BP1	MME	MME	COL15A1	PGCP
DPP4	DPP4	CTSO	SEMA3C	SLIT3	VCAM1
FBLN2	MMP9	NID2	EMILIN1	COL16A1	FLRT2
FLRT2	SELL	MMP19	MMP12	CHL1	ITGB3
ITGB3	CDH5	ICAM2	CDH13	ENPEP	ENPEP
GPC4	GPC4	LAMA2	ADAM8	FCN1	SEMA5A
ITGBL1	GZMA	CDH6	CPA3	PCDH17	NCAM2
COMP	BST1	ADAM17	ITGAM	ITGA4	SELP
CPM	ADAMDEC1	SELE	HAS2	PLXNC1	CD36
EFEMP2	CHST7	CDH11	CDH11	ADIPOQ	SIGLEC7
CD209	HAS1	ADAM28	PGCP	TNXB	PECAM1
PECAM1	PECAM1	COL18A1	COL18A1	MCAM	COL6A2
GPC3	SMC3	TSPAN4	TSPAN4	CTSZ	LAMA4
DCN	EFEMP2	ECM1	SPON1	SPON1	CD36
SERPINB9	ADAM19	SELPLG	SLIT2	CD300A	ITGAX
SGCD	FN1	NRP1	MCAM	CD44	TGM2
COL3A1	MCAM	DPP4	LAMB1	FN1	DCN
DCN	ITGB1	COL4A2	COL4A2	COL4A1	COL4A1
COL6A1	SDC2	SDC2	SDC2	SERPINE2	PLXND1
NRP1	FN1	COL5A1	COL5A1	SPG20	SPARC
ELN	COL14A1	COL6A1	SEMA5A	PLXNC1	CTSB
CTSB	COL6A2	ITGA4	COL6A1	ITGAL	SNED1
SNED1	LAMA2	ADAM17	SGCD	ICAM2	BGN
SPON1	SPON1	ITGB5	ITGB5	PLXDC1	OPCML
SGCD	SPG7	FN1	ADAMTS3	ITGBL1	COL3A1
ITGB3	TNN	THBS1	TNC	LAMB2	ITGA7
FN1	LAMA2	CD300A	MMP14	COL1A1	SPON2
CHST12	COL5A3	SIGLEC1	COL4A3BP	CHST11	PCDH12
SEMA3G	PLXDC1	MMP28	ADAMTS5	ADAMTS9	CORIN
LEPRE1	ADAMTS6	TMPRSS5	PLXNA1	COL5A2	COL5A2
CSGlcA_T	CLU	NAALAD2	ADAMTS1	PLXND1	COL5A3
CSGlcA_T					

IRCC-KM samples

tumor...40_I tumor...42_I tumor...46_I tumor...48_I tumor...4_no tumor...8_no
tumor...17_n tumor...22_n tumor...25_n tumor...26_n tumor...34_n

10.2.1 Comparing LAS and IRCC-KM biclusters

	No ECM	ECM3
No ECM3	649	1
ECM3	144	109
Jaccard similarity	0.43	

Table 58: Comparing gene lists: LAS (column) vs. IRCC-KM bicluster (row)

	No ECM	ECM3
No ECM3	31	6
ECM3	3	8
Jaccard similarity	0.47	

Table 59: Comparing sample lists: LAS (column) vs. IRCC-KM bicluster (row)

10.3 IRCC-HC bicluster

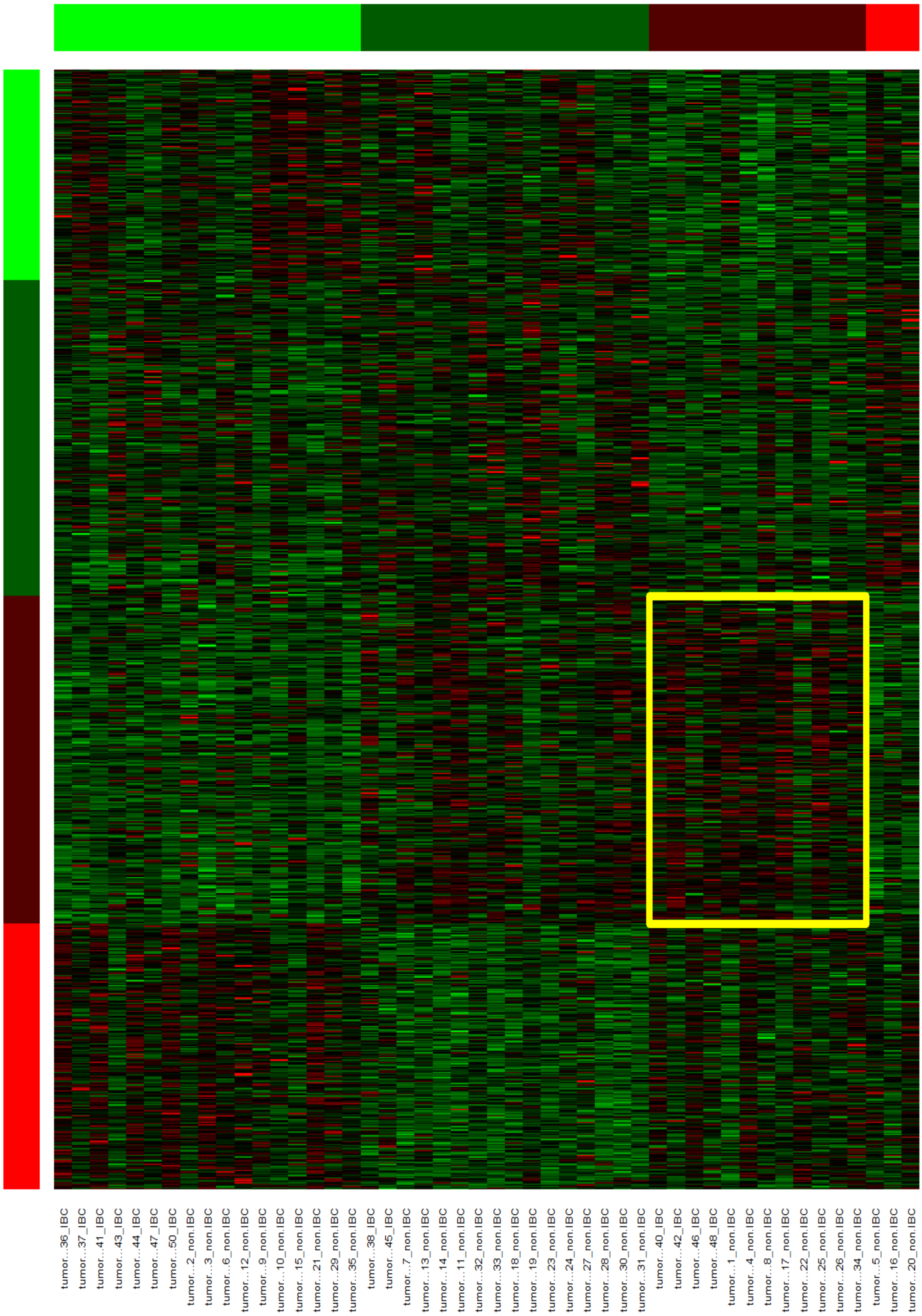

Figure 77: Heatmap of the IRCC-HC bicluster

IRCC-HC genes

ADAM12	ADAM12	ADAM17	ADAM17	ADAM19	ADAM28
ADAM28	ADAM3A	ADAM8	ADAMDEC1	ADAMTS1	ADAMTS2
ADAMTS5	ADAMTS9	ADIPOQ	BGN	BGN	BGN
BST1	CD209	CD300A	CD300A	CD36	CD36
CD6	CDH11	CDH11	CDH13	CDH5	CDH6
CHL1	CHST11	CHST12	CHST2	CHST7	COL10A1
COL10A1	COL11A1	COL11A1	COL14A1	COL15A1	COL16A1
COL18A1	COL18A1	COL1A1	COL1A1	COL1A1	COL1A2
COL1A2	COL3A1	COL3A1	COL3A1	COL4A1	COL4A1
COL4A2	COL4A2	COL4A3BP	COL5A1	COL5A1	COL5A1
COL5A2	COL5A2	COL5A3	COL5A3	COL6A1	COL6A1
COL6A1	COL6A2	COL6A2	COL6A3	COL8A2	COL8A2
COMP	CORIN	CPA3	CPM	CPM	CTSB
CTSB	CTSB	CTSB	CTSC	CTSD	CTSG
CTSH	CTSK	CTSS	CTSS	CTSW	CTSZ
DCN	DCN	DCN	DCN	DKFZP586H212	DPP4
DPP4	DPP4	EFEMP2	EFEMP2	EMILIN1	ENPEP
ENPEP	FBLN1	FBLN1	FBLN2	FBN1	FBN1
FCN1	FLRT2	FLRT2	FN1	FN1	FN1
FN1	GZMA	GZMB	GZMH	GZMK	HAS1
HAS2	HSPG2	HTRA1	HYAL2	ICAM1	ICAM1
ICAM2	ICAM2	ITGA10	ITGA3	ITGA4	ITGA4
ITGA5	ITGA7	ITGAE	ITGAL	ITGAM	ITGAX
ITGB1	ITGB2	ITGB3	ITGB3	ITGB3	ITGB7
ITGBL1	ITGBL1	LAMA2	LAMA2	LAMA2	LAMA4
LAMB1	LAMB1	LEPRE1	MARCO	MATN1	MATN2
MATN3	MCAM	MCAM	MCAM	MCAM	MME
MME	MMP11	MMP11	MMP12	MMP13	MMP14
MMP14	MMP16	MMP19	MMP2	MMP28	MMP7
MMP9	NAALAD2	NCAM2	NID1	NID1	NID2
NRP1	NRP1	NRP2	OPCML	PCDH12	PCDH17
PCOLCE	PECAM1	PECAM1	PECAM1	PLXDC1	PLXDC1
PLXNA1	PLXNA1	PLXNC1	PLXNC1	PLXND1	PLXND1
ROBO1	SELE	SELL	SELP	SELPLG	SEMA3C
SEMA3C	SEMA3G	SEMA4D	SEMA5A	SEMA5A	SERPINB9
SERPINE1	SERPINE1	SERPINF1	SERPING1	SERPINH1	SGCD
SGCD	SGCD	SGCD	SIGLEC1	SIGLEC1	SIGLEC7
SIGLEC9	SLIT2	SLIT3	SNED1	SNED1	SPARC
SPARC	SPARCL1	SPOCK1	SPOCK2	SPOCK2	SPOCK3
SPON1	SPON1	SPON1	SPON1	SPON2	TGM2
TGM2	THBS1	THBS1	THBS1	THBS1	THBS1
THBS2	THBS4	TIMP1	TIMP2	TIMP3	TIMP3
TIMP3	TIMP3	TNC	TNC	TNXB	TSPAN3
TSPAN3	TSPAN4	TSPAN4	TSPAN7	VCAM1	VWF

IRCC-HC samples

```
tumor...40_I tumor...42_I tumor...46_I tumor...48_I tumor...1_no tumor...4_no
tumor...8_no tumor...17_n tumor...22_n tumor...25_n tumor...26_n tumor...34_n
```

10.3.1 Comparing LAS and IRCC-HC biclusters

	No ECM	ECM3
No ECM3	632	7
ECM3	161	103
Jaccard similarity	0.38	

Table 60: Comparing gene lists: LAS (column) vs. IRCC-HC bicluster (row)

	No ECM	ECM3
No ECM3	30	6
ECM3	4	8
Jaccard similarity	0.44	

Table 61: Comparing sample lists: LAS (column) vs. IRCC-HC bicluster (row)

10.4 CCSS bicluster

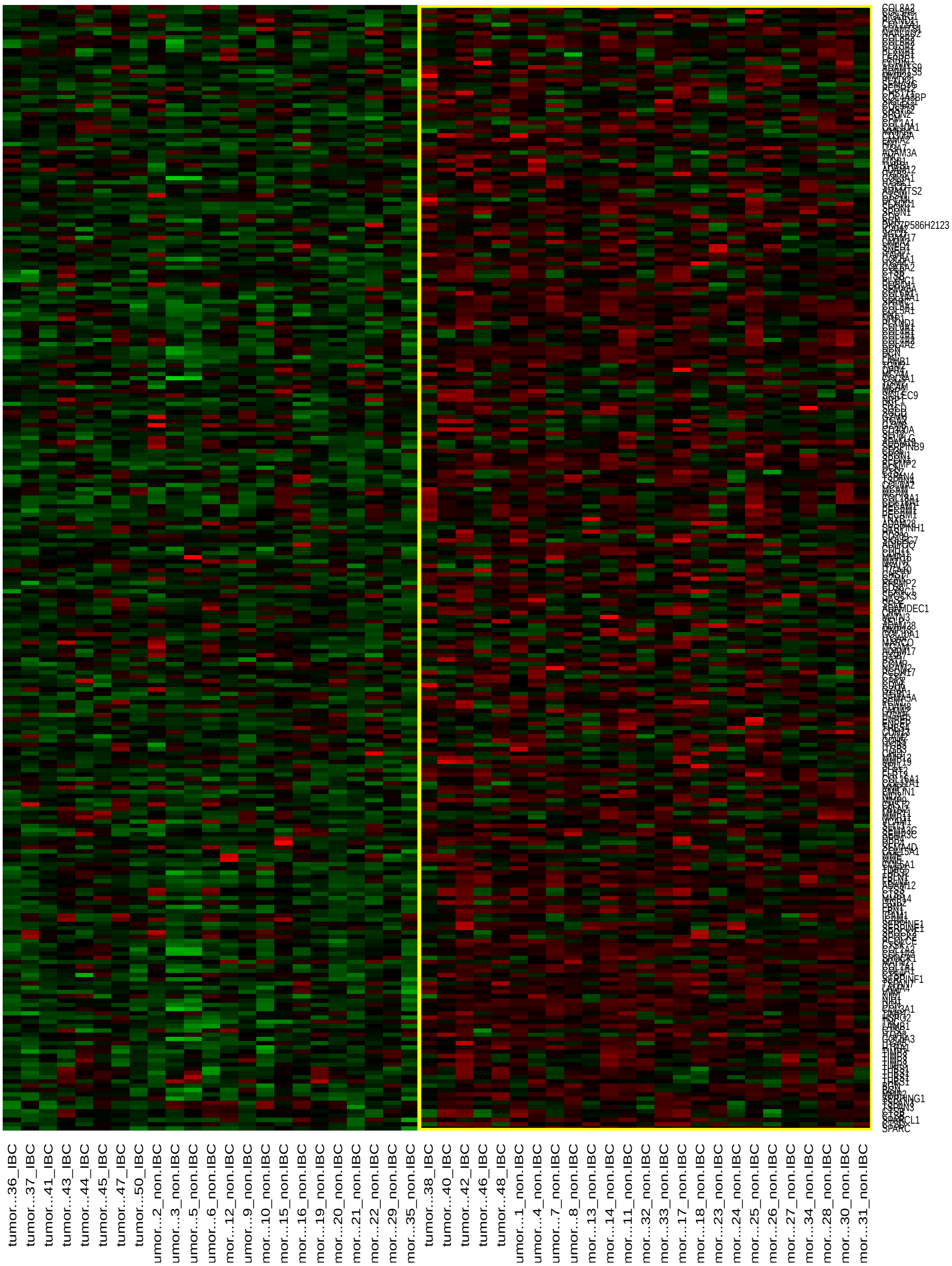

Figure 78: Heatmap of the CCSS bicluster

10.4.1 Comparing LAS and CCSS biclusters

	No ECM	ECM3
No ECM3	23	0
ECM3	11	14
Jaccard similarity	0.56	

Table 62: Comparing sample lists: LAS (column) vs. CCSS (row) biclusters

10.4.2 Prediction strength for CCSS

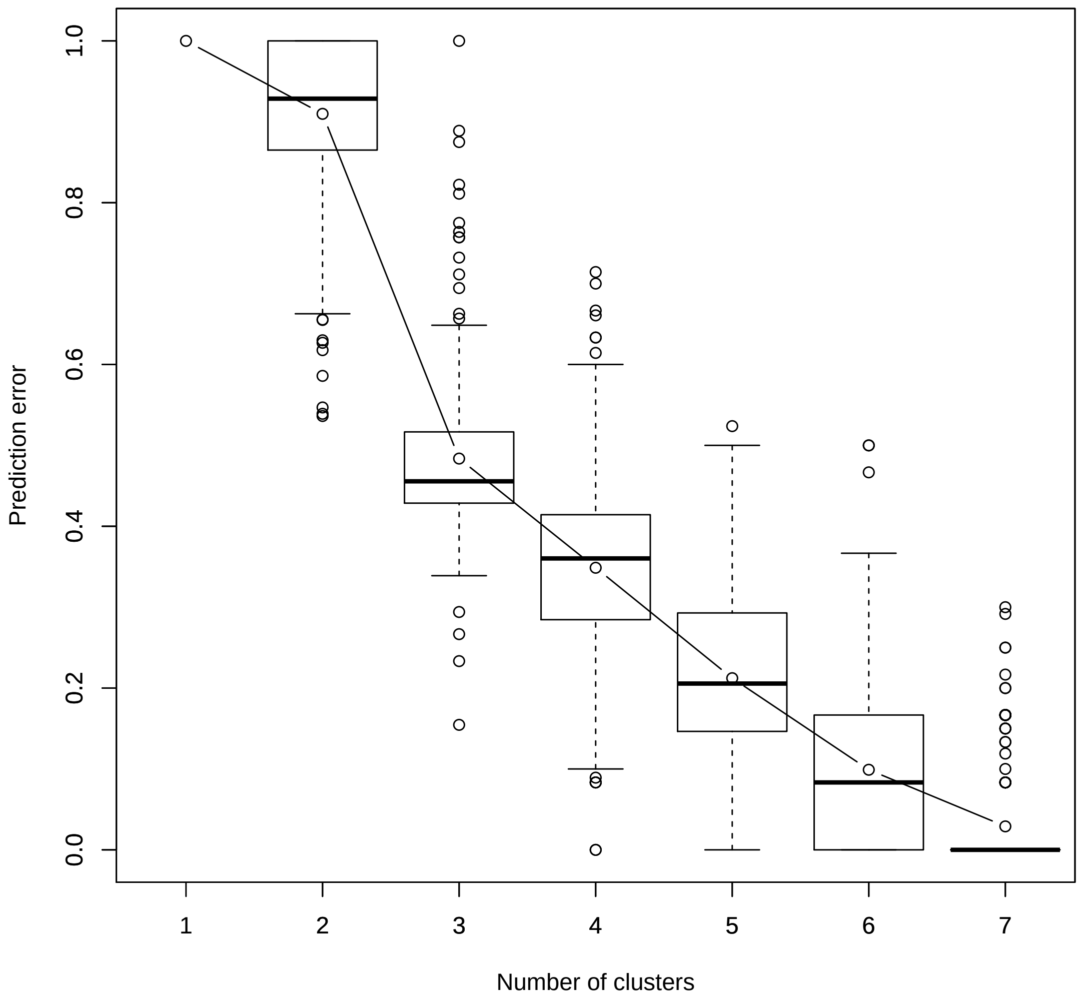

10.4.3 Consensus clustering

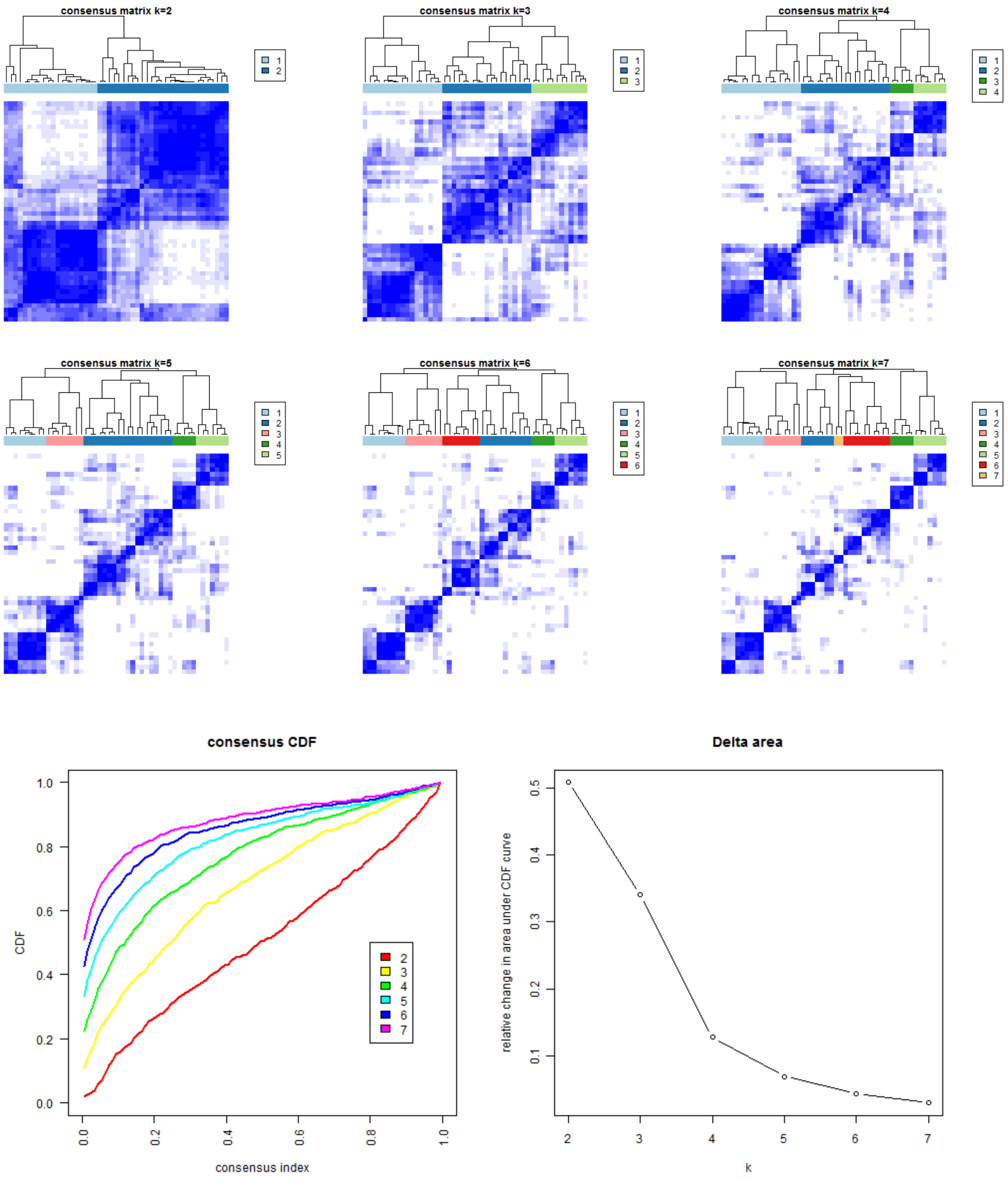

Figure 79: Statistical significance of CCSS clustering (Consensus clustering)

10.4.4 Statistical significance

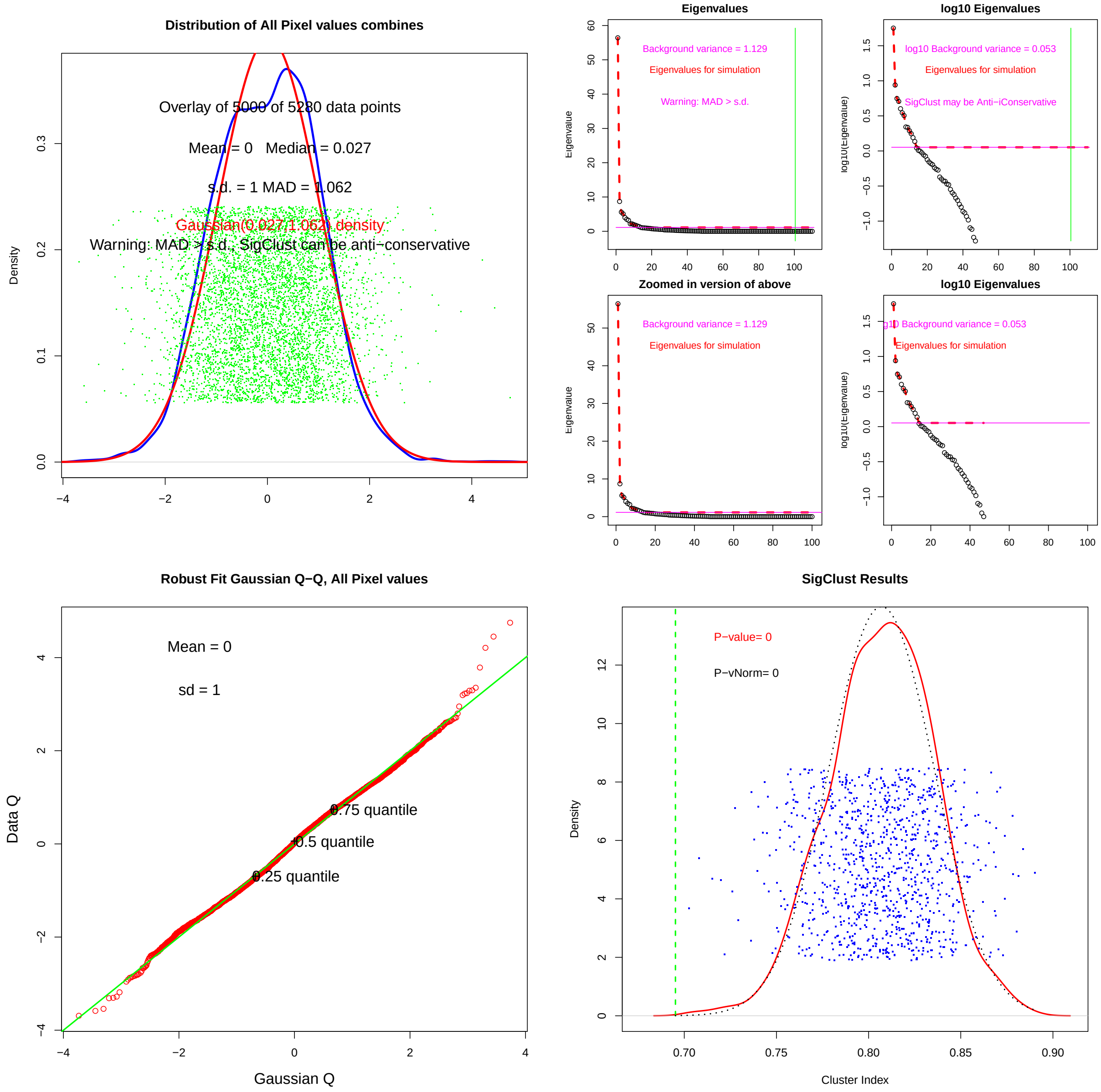

P-value	P-vNorm
0.0E+00	4.4E-05

Table 63: SigClust p-values

10.4.5 Cluster-wise assessment of cluster stability

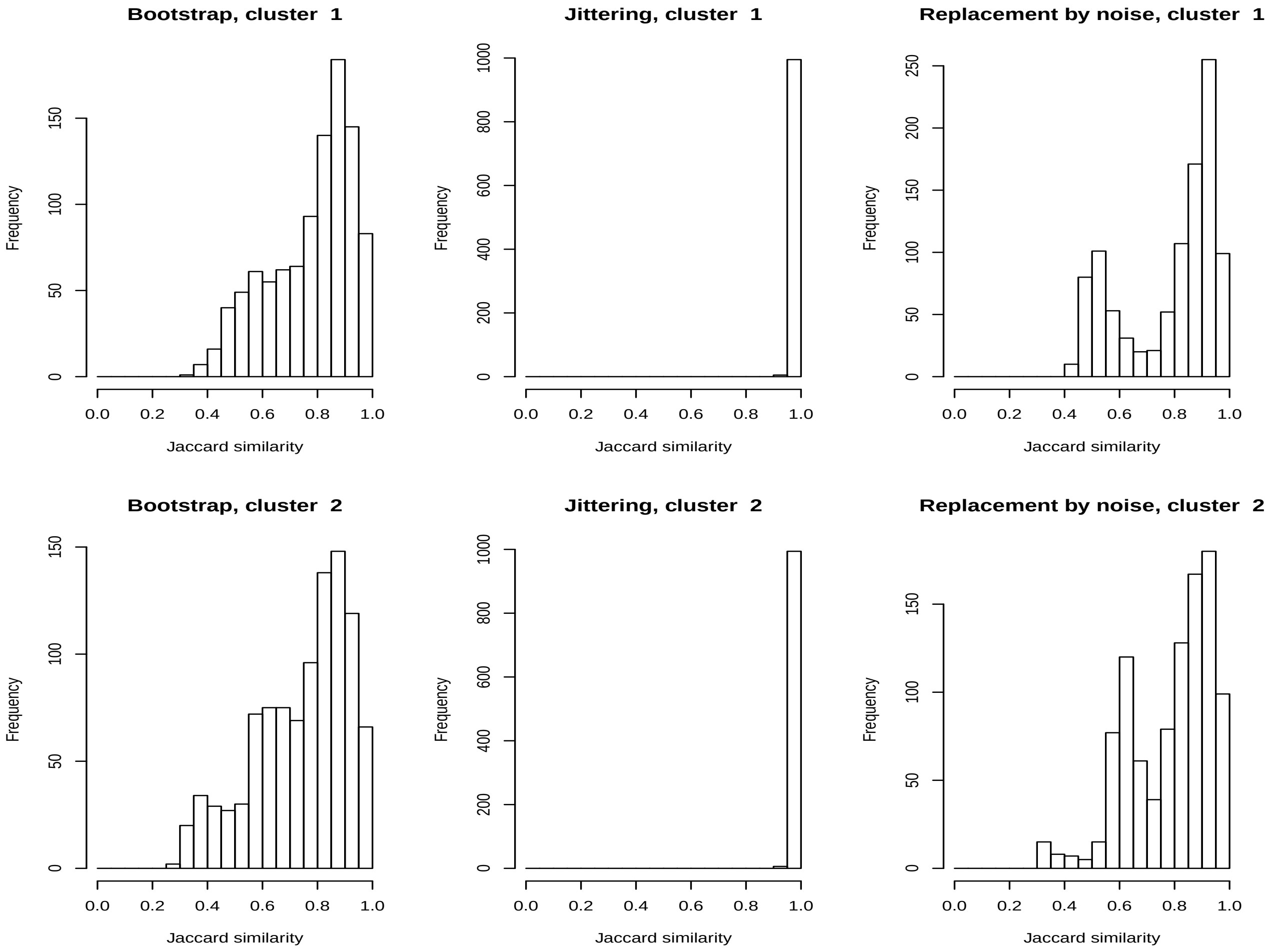


```
* Cluster stability assessment *
Cluster method:  kmeans
Full clustering results are given as parameter result
of the clusterboot object, which also provides further statistics
of the resampling results.
Number of resampling runs:  1000

Number of clusters found in data:  2

 Clusterwise Jaccard bootstrap mean:
[1] 0.7789089 0.7438281
dissolved:
[1]  64 112
recovered:
[1] 645 567
 Clusterwise Jaccard jittering mean:
[1] 0.9990581 0.9989084
dissolved:
[1] 0 0
recovered:
```

```
[1] 1000 1000
 Clusterwise Jaccard replacement by noise mean:
[1] 0.7869988 0.7863675
dissolved:
[1] 90 35
recovered:
[1] 684 653
```

Removing one sample

```
   Min. 1st Qu.  Median    Mean 3rd Qu.    Max.
 0.5106  0.5106  0.5106  0.5208  0.5319  0.5319
```

Removing one gene

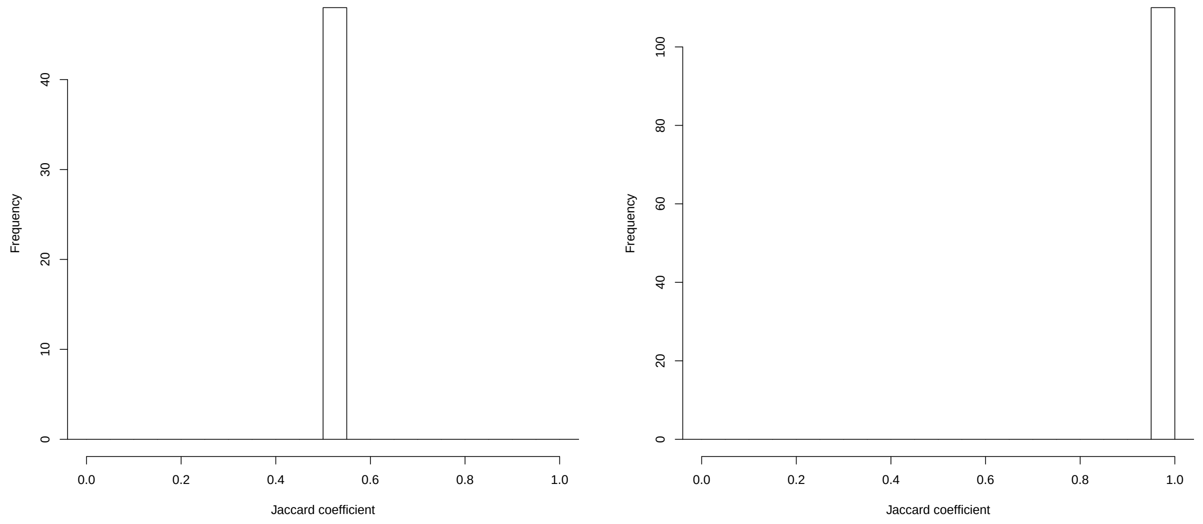

Figure 80: Removing one sample (left) and one gene (right) at a time: distributions of Jaccard coefficients

```
   Min. 1st Qu.  Median    Mean 3rd Qu.    Max.
      1       1       1       1       1       1

      APN             AD               ADM               FOM
 Min.   :0.31   Min.   :12.66   Min.   :4.739   Min.   :0.5776
 1st Qu.:0.31   1st Qu.:12.66   1st Qu.:4.739   1st Qu.:0.7176
 Median :0.31   Median :12.66   Median :4.739   Median :0.7984
 Mean   :0.31   Mean   :12.66   Mean   :4.739   Mean   :0.8010
 3rd Qu.:0.31   3rd Qu.:12.66   3rd Qu.:4.739   3rd Qu.:0.8822
 Max.   :0.31   Max.   :12.66   Max.   :4.739   Max.   :1.0121
```

Removing sets of k genes

```
   Min. 1st Qu.  Median    Mean 3rd Qu.    Max.
 0.7812  0.9231  0.9615  0.9517  1.0000  1.0000
```

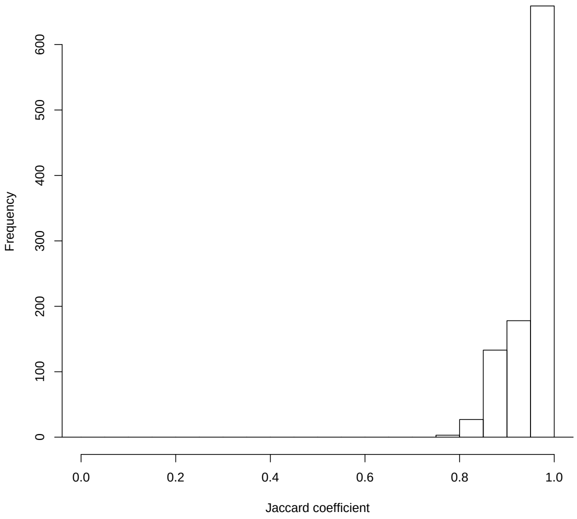

Figure 81: Removing two thirds of the genes: distribution of Jaccard coefficients

10.4.6 Validation measures

```
Clustering Methods:
 kmeans

Cluster sizes:
 2 3 4 5 6

Validation Measures:
                           2       3       4       5       6

kmeans APN            0.0000  0.0722  0.0548  0.0730  0.1391
       AD             0.9436  0.9306  0.8485  0.8202  0.7896
       ADM            0.0000  0.9645  0.6306  0.7693  1.3773
       FOM            0.8010  0.7575  0.7290  0.7062  0.6943
       Connectivity  43.6552 71.5976 67.5746 77.8921 77.0595
       Dunn           0.2697  0.2286  0.2697  0.2697  0.2697
       Silhouette     0.0641  0.0131  0.0532  0.0213  0.0236

Optimal Scores:

             Score   Method Clusters
APN           0.0000 kmeans 2
AD            0.7896 kmeans 6
ADM           0.0000 kmeans 2
FOM           0.6943 kmeans 6
Connectivity 43.6552 kmeans 2
Dunn          0.2697 kmeans 2
Silhouette    0.0641 kmeans 2
```
